# MacSyFinder: A Program to Mine Genomes for Molecular Systems with an Application to CRISPR-Cas Systems

**DOI:** 10.1371/journal.pone.0110726

**Published:** 2014-10-17

**Authors:** Sophie S. Abby, Bertrand Néron, Hervé Ménager, Marie Touchon, Eduardo P. C. Rocha

**Affiliations:** 1 Microbial Evolutionary Genomics, Institut Pasteur, Paris, France; 2 UMR3525, CNRS, Paris, France; 3 Centre d’Informatique pour la Biologie, Institut Pasteur, Paris, France; Universidad de La Laguna, Spain

## Abstract

**Motivation:**

Biologists often wish to use their knowledge on a few experimental models of a given molecular system to identify homologs in genomic data. We developed a generic tool for this purpose.

**Results:**

**Mac**romolecular **Sy**stem **Finder** (MacSyFinder) provides a flexible framework to model the properties of molecular systems (cellular machinery or pathway) including their components, evolutionary associations with other systems and genetic architecture. Modelled features also include functional analogs, and the multiple uses of a same component by different systems. Models are used to search for molecular systems in complete genomes or in unstructured data like metagenomes. The components of the systems are searched by sequence similarity using Hidden Markov model (HMM) protein profiles. The assignment of hits to a given system is decided based on compliance with the content and organization of the system model. A graphical interface, MacSyView, facilitates the analysis of the results by showing overviews of component content and genomic context. To exemplify the use of MacSyFinder we built models to detect and class CRISPR-Cas systems following a previously established classification. We show that MacSyFinder allows to easily define an accurate “Cas-finder” using publicly available protein profiles.

**Availability and Implementation:**

MacSyFinder is a standalone application implemented in Python. It requires Python 2.7, Hmmer and makeblastdb (version 2.2.28 or higher). It is freely available with its source code under a GPLv3 license at https://github.com/gem-pasteur/macsyfinder. It is compatible with all platforms supporting Python and Hmmer/makeblastdb. The “Cas-finder” (models and HMM profiles) is distributed as a compressed tarball archive as Supporting Information.

## Introduction

Macromolecular systems are involved in key aspects of cell biology [1], [2]. They can be constituted of nanomachines, like the ribosome or the flagellum, or molecular pathways, like the ones allowing the degradation of foreign genetic elements by CRISPR-Cas systems. The identification and classification of macromolecular systems is important to characterize biological traits, and is routinely done in many laboratories. However, it is difficult to do on a systematic basis by a number of reasons. Firstly, systems are made of many different components with different levels of dispensability, some being essential and others accessory. For example, homologous recombination in bacteria involves some key essential components (like RecA), and several associated alternative pathways (like RecBCD and RecFOR) [3]. Secondly, key components may have homologs in other systems, complicating their unambiguous assignment to a given system. This is for instance the case of the non-flagellar type III secretion system for which eight of the nine core genes have homologs in the bacterial flagellum [4]. Thirdly, the components of the systems evolve at very diverse rates, complicating the identification of homology by sequence similarity. For example, many proteins involved in reproduction are highly conserved, whereas others endure selection for fast evolution [5]. These difficulties can be partly circumvented by searching for the whole set of components of the system because the integration of all the information leads to more accurate inference. This is especially relevant if the genes encoding these components are organized in highly conserved ways. In Prokaryotes, organelles and viruses, macromolecular systems are often encoded in one or a few conserved neighbouring operons ensuring tight regulation and correct assembly/functioning. This facilitates the assignment of certain components to a system [6]–[9].

We have developed a program named **Mac**romolecular **Sy**stem **Finder** (MacSyFinder) to detect molecular systems in genome data from user-defined biological models. The components of the systems are searched using protein profiles encoded as hidden Markov models (HMM), such as those available in databases like PFAM, TIGRFAM or PRODOM [10]–[12]. Protein profiles provide a compressed way to represent a database of homologous sequences, giving increased sensitivity and specificity [13]. MacSyFinder identifies the presence of a given system according to the specifications of the input model, which includes customizable information on the type and number of components, on their genetic organization, and other relevant discriminating traits. We implemented MacSyFinder as a generic portable tool that can be installed in-house for large genomic or metagenomic projects. The companion program, MacSyView, allows the visualization of the results of MacSyFinder. To show a typical situation where MacSyFinder can be useful, we built a set of models to identify Cas proteins. Clustered regularly interspaced short palindromic repeats (CRISPR) arrays and their associated Cas (CRISPR-associated) proteins form the CRISPR-Cas system. CRISPR-Cas are sophisticated adaptive immune systems that rely on small RNAs for sequence-specific targeting of foreign nucleic acids such as viruses and plasmids [14]. Cas proteins have been intensively studied in the recent years for their role in the interaction between Prokaryotes and their mobile genetic elements and for their biotechnological interest [15], [16]. Tools are available to detect and analyse CRISPR arrays [17]–[19], however, no program is available to detect and class *cas* operons themselves. This example shows that using information from the literature and available protein profiles, one can easily build an accurate and efficient “Cas-finder” with MacSyFinder.

## MacSyFinder's Rationale

### Definition of the models

**Figure 1:**
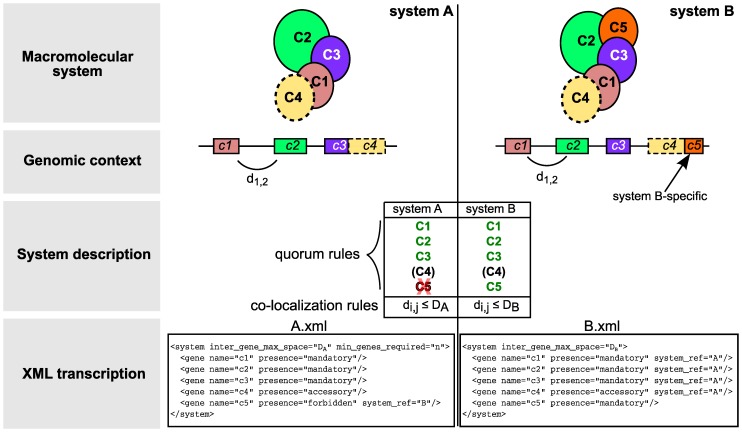
Modelling systems with MacSyFinder. The components of a system assemble into macromolecular systems or correspond to a biological pathway. They are typically encoded in genomes in one or a few different loci (“Genomic context”). We illustrate how systems can be modelled and distinguished with two imaginary systems “A” and “B” that have four homologous components (C1–C4, similar colours for the two systems). The system “B” has one component that is not found in “A”(C5). The parameter *inter_gene_max_space* (D) defines the maximal number of genes between two consecutive components (d_i,j_). The two systems are defined by a set of *mandatory* (green), *accessory* (black) and *forbidden* (red) components. The quorum rules allow relaxing the definition of the system without altering the list of its components (*min_genes_required* and *min_mandatory_genes_required* parameters in XML files). If they are not specified, a default value is computed from the number of components described in the XML files. The bottom part of the figure shows the description of the systems in the XML grammar (see the documentation in File S1). Components listed here refer to protein profiles (Fig. 3). When a component is found in several systems, it is defined only once, and can be reused in another system with the *system_ref* keyword. Much more complex features can be defined, including exchangeable genes, distant genes and component-specific parameters (File S1).

MacSyFinder models, written using an XML grammar, describe the components and genetic organization of a given macromolecular system (see the documentation in File S1 for a full description of the grammar). Each model is defined in a dedicated file named after the type of system (*e.g.,* CAS-TypeI.xml'), which contains system-wise and component-wise features (Figure 1). MacSyFinder considers three classes of components: *mandatory*, *accessory*, and *forbidden*. Components that are ubiquitous and identifiable in all systems are defined as *mandatory*. Other components of the system are defined as *accessory*. These *accessory* components can be essential for the assembly/functioning of the system, while not being identifiable by sequence similarity because of rapid evolution or because they are non-homologous among variants of the system. Discrimination between partly homologous systems is easier when some specific components are defined as *forbidden* in the models of the systems lacking them (Fig. 1).

Systems that respect a pre-defined minimal quorum of components are identified as complete. The quorum is either the number of *mandatory* components and/or the sum of *mandatory* and *accessory* components (see the documentation on attributes *min_mandatory_genes_required* and *min_genes_required* in File S1). Components defined as functionally *exchangeable* are only counted once in the quorum. These components can be part of systems defined in other models using the *system_ref* keyword. Genes encoding components that participate in multiple systems of the same type, such as proteins interacting with different instances of a system, are labelled *multi_system*.

The genetic architecture of the components is defined using several attributes. Two components are co-localized when their genes are closer than a given number of genes (system-wise parameter *inter_gene_max_space*, Fig. 1). A component defined with the *loner* attribute does not need to be co-localized with other components to be part of a system. One can also specify component-specific values of *inter_gene_max_space*. The system-wise parameter *multi_loci* allows MacSyFinder to detect systems encoded by several distant clusters of genes.

### Implementation, system requirements and availability

MacSyFinder was coded in Python, and details on its object-oriented implementation are available in Text S1 and in File S1. MacSyFinder requires Python version 2.7, the formatdb or makeblastdb tools (version 2.2.28 or better for the latter) [20], [21] and the program Hmmer [13], [22]. MacSyFinder is freely available. Its source code is distributed under a GPLv3 license at https://github.com/gem-pasteur/macsyfinder and updated versions will be accessible there. MacSyFinder is compatible with all platforms supporting Python, Hmmer, and makeblastdb. The MacSyFinder release used in this paper is provided in Data S1. MacSyView's source code is freely available at https://github.com/gem-pasteur/macsyview but it is also distributed in the MacSyFinder's package. MacSyView was coded in Javascript and uses third-party libraries that are included in the package, and accredited in the COPYRIGHT file (See Text S1). It was tested on Chromium and Firefox for Linux, and on Chrome, Firefox and Safari for Mac OS X. A documentation file including installation and users' instructions, details on modelling procedures and examples for MacSyView and MacSyFinder is available in File S1.

### Input and output

The MacSyFinder program (Data S1) receives as input a list of systems defined in XML files (see above), protein profiles, command-line parameters and a file with protein sequences in fasta format (see the documentation in File S1). The parameters can be specified in the command-line or in a configuration file. System and component parameters specified in the command-line override model specifications in the XML files.

MacSyFinder manages three different types of protein datasets. The **unordered** dataset lacks information on gene order and genome origin. This mode is useful to study large sequence databanks or metagenomic data. Naturally, in these datasets the notions of co-localization and quorum are not relevant. The **unordered replicon** dataset includes protein sequences from one single genome. This is useful to analyse unassembled genomes with large numbers of contigs. In this case the notion of quorum is relevant (albeit with certain limitations), but co-localization is not. The **ordered replicon** dataset includes proteins from one single replicon that are ordered according to the position of the corresponding genes in the genome. This is the most powerful mode and can be used to analyse complete or nearly complete genomes. Another related mode (*gembase*) requires a specific input file format and allows the analysis of multiple ordered replicons in a single step (see File S1).

**Figure 2:**
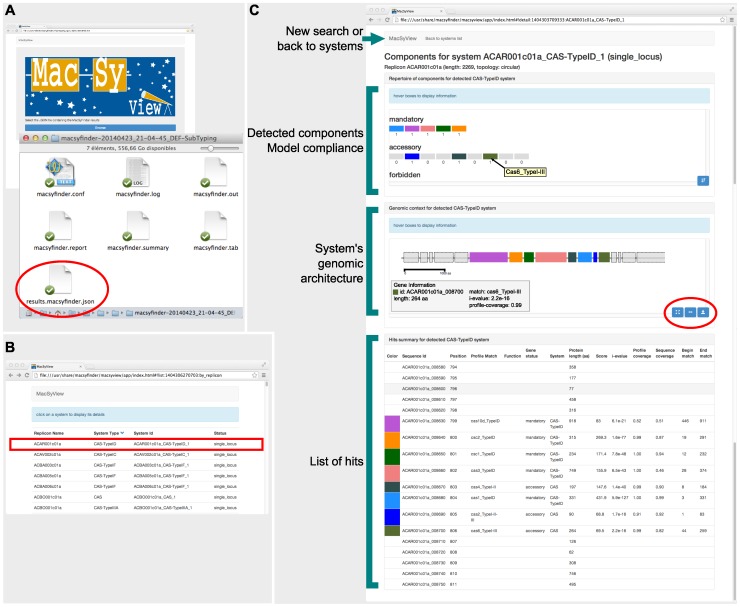
Snapshot of MacSyFinder's results as viewed with MacSyView. A. The MacSyView web-browser based application allows the visualization of MacSyFinder's output file “results.macsyfinder.json”. B. MacSyView displays the list of systems available in the results file. The user picks a system to visualize by clicking on it in the list. C. The page displaying the system is made of a header, and three panels. The header allows to select another input file, or to go back to the list of systems. It displays information on the system that is being visualized. The first panel shows how the detected system fits the model compliance in terms of its components. Boxes represent the number of each *mandatory*, *accessory*, and *forbidden* components. A tooltip gives the name of the component when the mouse hovers a box. Component boxes can be sorted by decreasing number of components. The second panel shows the genetic context of the system (as transcribed from the input fasta file), with components drawn to scale. When the mouse hovers a box, a tooltip displays information on the corresponding component, including scores of the Hmmer hit. This view can be exported as a SVG file for drawing purposes (tools circled in red). The third panel gives detailed information on the components of the system.

The output of MacSyFinder includes log files, intermediate results, the number of detected systems, and the information on each detected component from each instance of the system. This information is made available in the form of text tables and JSON files. We have built MacSyView, a standalone web-browser application that uses output JSON files to visualise the systems and their genomic context. MacSyView generates exportable SVG files containing views of the detected systems (Fig. 2).

### Functioning

**Figure 3:**
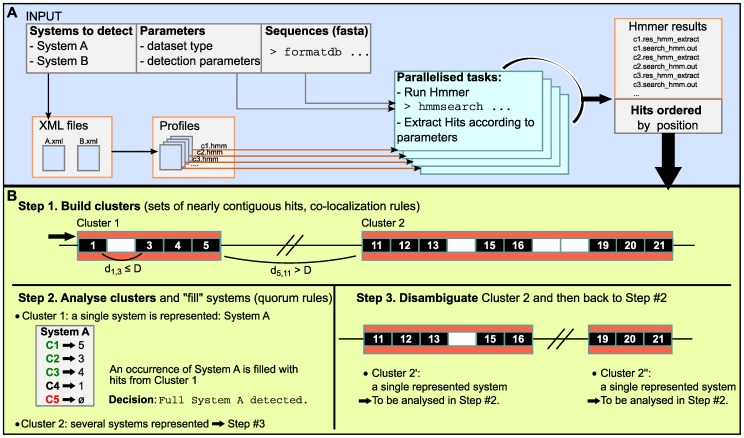
Functioning of MacSyFinder. A. The user launches MacSyFinder to detect macromolecular systems A and B (example of Fig. 1). System-specific parameters are read from the corresponding XML definition files. This includes the list of the components of the systems and the corresponding HMM profiles. Other detection parameters are picked by order of priority: on the command-line, in the configuration file, and in the XML files. Sequences are indexed with the “formatdb” or “makeblastdb” tools for similarity search with the Hmmer program. MacSyFinder runs (optionally in parallel) the Hmmer searches on a non-redundant list of components' profiles. If the sequence dataset is “unordered” MacSyFinder only outputs the hits and the components detected for each type of system. B. Step #1: the co-localization criterion can be used in the ordered datasets. It involves clustering the hits separated by less than D protein-coding genes. The components described as “loner” in the XML definition files can be at any distance from other components. Step #2: the components of each cluster are used to fill the occurrences of the systems. Depending on the quorum, a cluster can describe a “full” system, or a “scattered” system. Step #3: clusters with components belonging to more than one system are split in unique systems and then re-directed separately to step #2.

The user runs MacSyFinder from the command-line on a protein sequence dataset for a number of systems of interest. The non-redundant list of components to search is extracted from the XML files. The presence of a given component is determined by similarity search with HMM protein profiles using the program Hmmer [13]. The hits are filtered according to user-defined i-evalue (for statistical significance) and to the minimal coverage of the profile in the alignment (to control for the minimal size of the profile that must be matched to obtain biologically relevant hits). The components defined in the models are searched in parallel for rapidity (Fig. 3A). If multiple profiles match the same protein, MacSyFinder selects the hit with the highest score. The subsequent steps depend on whether the input dataset is an ordered replicon, an unordered genome or an unordered genomic dataset (*e.g*., a metagenome).

If the dataset is an **ordered replicon**, the hits are clustered according to the genetic organization specified in the model. Clusters including the components of a single type of system are used to fill inventories of “compatible” systems (Fig. 3B). If multiple systems are compatible with the set of components in the clusters, then the different candidate systems are examined. The order of exam is given by decreasing number of components shared between the cluster and the compatible systems. The cluster will be assigned to the first system in the list that fits its content. A system is regarded as complete if the quorum is respected. When a complete instance of the system has components from a single locus, further new occurrences of the same components in the cluster are used to produce a novel instance. When a single cluster is not enough to make a complete instance and the *multi_loci* parameter is turned on, the hits are stored to fill up an instance of the system encoded by multiple distant loci. Clusters with components from multiple systems are split in sub-clusters containing components from a single system. These sub-clusters are then re-analysed in terms of their components (Fig. 3B). MacSyFinder can only resolve these complex cases if the components of each system are contiguous, instead of scattered on the cluster.

**Unordered** sequence datasets cannot be analysed with the co-localization criteria. Therefore, hits from the similarity searches are directly used to fill inventories of each system. Systems are complete if the required quorum is respected. The presence of *forbidden* components is ignored in this mode, even if such occurrences are stored to inform the user. A single system instance will be filled per system and dataset, independently of the number of component occurrences found. This is because components cannot be individually assigned to particular instances in the absence of the genomic context. Nevertheless, the analysis of the number of identified components can be used to estimate the number of instances in the dataset.

## Application

### Data

The complete genomes of bacteria (2484) and archaea (159) were downloaded from NCBI RefSeq (ftp://ftp.ncbi.nih.gov/genomes/, November 2013). Profiles for the Cas protein families were obtained from the TIGRFAM database, version 13.0 (http://www.jcvi.org/cgi-bin/tigrfams/index.cgi, August 15 2012) [11], [23]. Among the 89 profiles available, 53 were constructed by Haft and colleagues [24] and correspond to 45 Cas protein families that were used to propose a CRISPR-Cas systems classification [25]. Subsequently, these authors constructed 36 additional protein profiles more specific to given subtypes that are available in TIGRFAM [23]. We renamed these profiles to make them more informative for the user (see Table S1 for correspondence with established classification).

### Developing a new system's model

The goal of MacSyFinder is to query genomic data using biologically meaningful models of a system. The first step of the model building procedure is therefore to use the available knowledge to identify the system components, their frequency and their genetic architecture. The second step is to obtain protein profiles for the components either by building them specifically for this purpose or by retrieving them from public databases. Protein profiles can be built easily from multiple sequence alignments of homologous proteins using Hmmer [13]. The third step is to write the model in the simple MacSyFinder's XML grammar (see above and Fig. 1 for an example). The final step is to include information about homologous systems in the model. The use of system-specific profiles and *forbidden* attributes facilitates the discrimination between systems (Fig. 1). Our experience is that complex models should be built by iterating several times on these steps from simpler models. Indeed, the fine-tuning of the quorum definitions and genetic architectures can vastly increase the quality of identification of a system. Often, one is confronted with systems for which very few instances have been experimentally studied. In this case, iteration of the modelling steps provides both more reliable models and a better knowledge of the systems diversity. To exemplify the use of MacSyFinder we built models to identify Cas proteins and classify CRISPR-Cas systems. This is a very typical example of systems that are intensively studied, for which there are many protein profiles in the databases, but no software dedicated to their detection.

### Detection and classification of CRISPR-Cas systems

**Figure 4:**
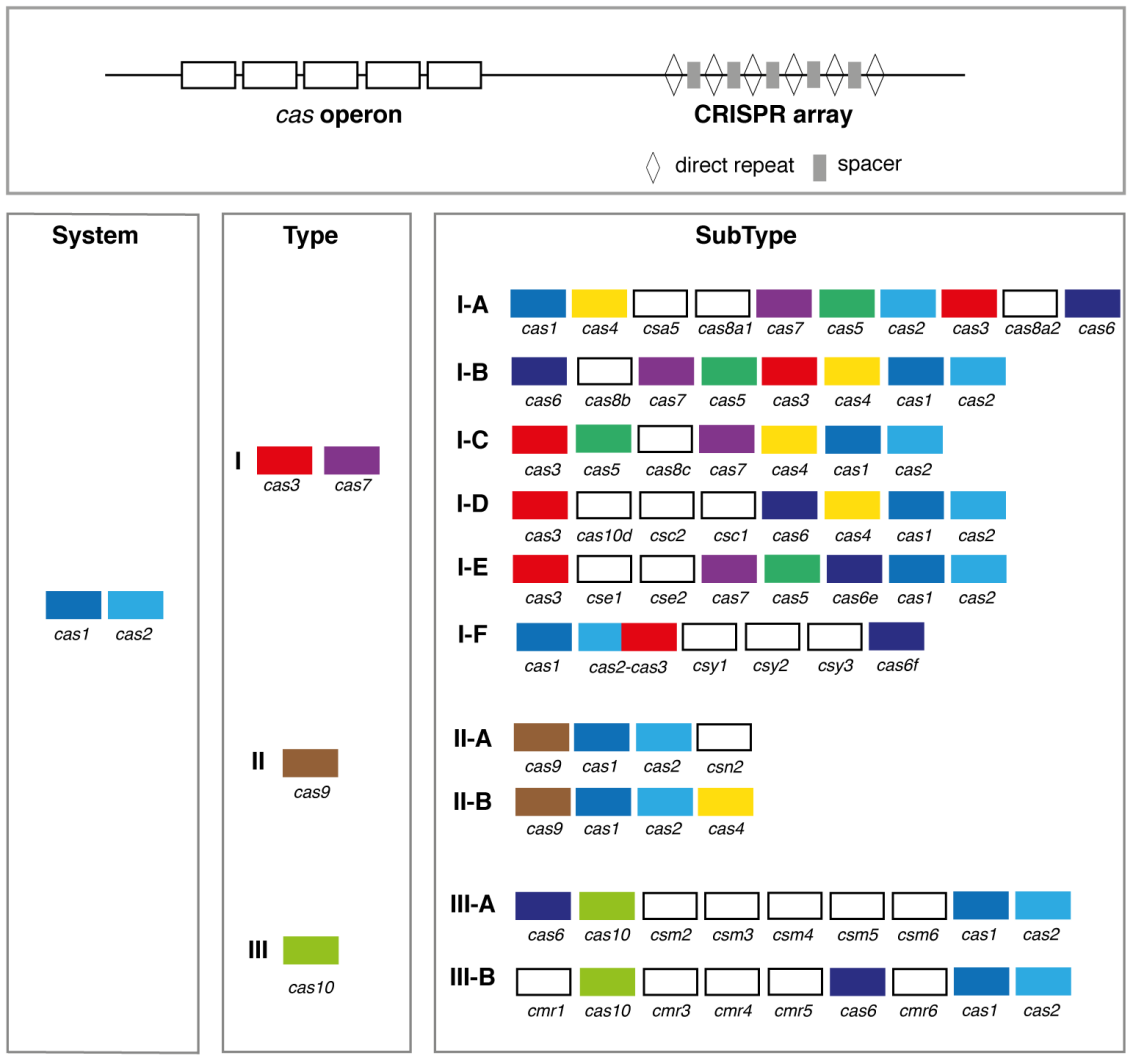
Simplified operon organization of the three major types and ten subtypes of CRISPR-Cas systems. Each *cas* gene family is indicated with a distinct colour, those specific to a subtype are in white. Only the main *cas* gene families are represented.

The known *cas* operons have from 3 to 13 genes encoding very diverse proteins, among which several nucleases and helicases with DNA and/or RNA binding domains [24], [25]. A unified classification of CRISPR-Cas systems has been recently established based on the presence or absence of peculiar Cas protein families, and on the genetic architecture of the *cas* operon [25]. Three major types and several subtypes of CRISPR-Cas systems have been described. *cas1* and *cas2* universally occur across types and subtypes, whereas *cas3*/*cas7*, *cas9*, and *cas10* have been defined as the signature genes for type I, type II, and type III, respectively (Fig. 4). Protein profiles matching most of these Cas protein families are publicly available in the TIGRFAM database [11], [24]. We used this information to exemplify how MacSyFinder can be used to identify and classify these systems.

#### General model and choice of parameters

In a first round of analysis, we defined a **general simple model** to identify all possible clusters of Cas proteins in 2643 prokaryotic genomes. In this general definition, all the CRISPR-Cas-HMM profiles available in TIGRFAM database were used whatever their type or subtype specificity (Table S1). At this stage, we used relatively relaxed criteria: all the components were defined as *accessory* and all clusters with at least 3 different components (*min_genes_required* = 3) distant from at most 5 genes (*inter_gene_max_space* “D” = 5) were retained. With this procedure, we identified 1628 clusters of Cas proteins and could annotate 10663 Cas proteins (*i.e.*, with significant matches to protein profiles). The total number of genes in the detected clusters ranged from 3 to 36 with an average of 7.7±3.5 genes (Fig. S1A). In these clusters, most of the genes (86%) encode known Cas proteins (*i.e.*, described in the general definition) and 56% of clusters have components strictly contiguous (Fig. S1B). While these preliminary results suggest that most clusters are Cas systems, a small fraction of them (7%) is larger than the larger described systems (>13 genes, Fig. S1A), suggesting that the above-mentioned parameters might be too permissive (Fig. S1C). These large clusters might correspond to contiguous or intertwined systems (*i.e.*, chimeric variants). To test this hypothesis, we explored the effect of changing D on the identification of clusters (*i.e.*, D = 4, 5, and 6, see Table S2). A more stringent co-localization criterion (D = 4), resulted in a decrease of the overall number of Cas proteins assigned to systems, the subdivision of several previously detected clusters, and the persistence of large clusters (Table S2). A less stringent criterion (D = 6) led to the fusion of several clusters with a small gain of Cas proteins assigned to systems (Table S2). We therefore set the final co-localization criterion to 5, and the minimal number of genes to 3. Doubts about multiple closely co-occurring systems can often be removed using more specific “typing” and “subtyping” models, because in this case contiguous systems of different types will be set apart (see below). While the general definition of the system is very simple, it fetches systems with Cas1 or a Cas2 protein in respectively 88% and 73% of the clusters, even if weak constraints were imposed on their presence (*accessory* proteins in the general definition of the system). We identified Cas clusters in 78% of archaeal genomes and 39% of bacterial genomes. This is very similar to previous observations and therefore suggests that even the general model accurately identifies Cas systems [26] (Fig. S1D, and see the paragraph on the validation of our models).

#### Typing and Subtyping CRISPR-Cas systems

**Figure 5:**
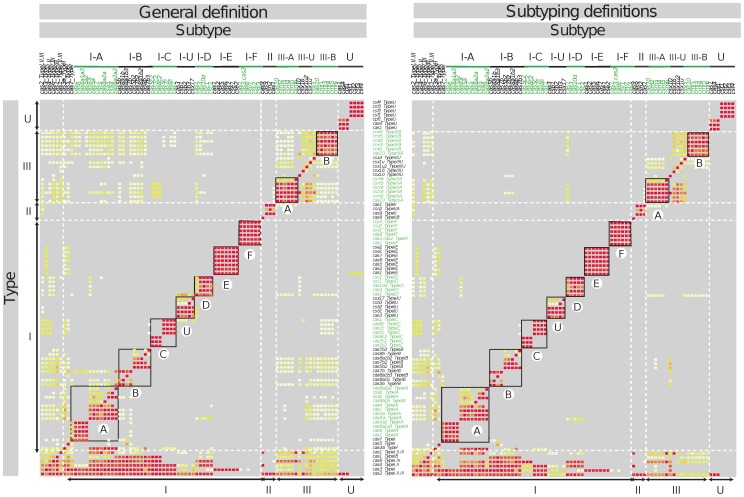
Frequency of co-occurrence between Cas proteins present in clusters detected with the general model (left) and the subtyping models (right). Each matrix was normalized by the maximum of each column. The higher the frequency is, the warmer the colour is: the red diagonal corresponds to a 100% co-occurrence. Only frequencies above 1% were represented, others are in grey.

To exemplify the ability of MacSyFinder to characterise sub-systems we built models for each type and subtype of Cas systems from the pre-existing classification [25] (Fig. 4). We first tested the specificity of the 89 available protein profiles for a given type and subtype by analysing the co-occurrence of pairs of Cas proteins in clusters detected with the general model (Fig. 5 and Text S1). Then we designed the corresponding models accordingly. In the final models (Fig. S2 and Data S2), all profiles specific to a system were defined as *mandatory* (signature gene) while all the others were defined as *accessory*. Because some systems have very similar content and organization (*e.g.*, Type II-A and II-B), profiles distinguishing them are *accessory* or *mandatory* in a system, and *forbidden* in the other (see Fig. 1 and Fig. S2 for examples). Although the types and subtypes have different numbers of genes, we set the *min_genes_required* parameter to 3, and the *inter_gene_max_space* parameter to 5 for all models to make the detection as large as possible and comparable with that resulting from the general model. We defined 5”typing” models and 15”subtyping” models for *cas* loci detection and classification (see Fig. S2 and Data S2).

Using the **subtyping models** we classed the previously detected Cas clusters, but were also able to split different contiguous systems (Fig. 5). Thus, among the 1628 Cas clusters, 95% correspond to a single system, 3% to contiguous distinct systems (including the type III-B well known to be associated with other systems-type [26]), the remaining 2% correspond to chimeric variants. Most of the Cas clusters could readily be assigned to proposed types (97%) and subtypes (94%) with our models. The remaining corresponded to *cas* locus with no gene signature, or to chimeric variants (Table S3).

#### Validation of detected systems

We made two analyses to obtain a more precise assessment of the accuracy of the method. Firstly, we quantified how often Cas systems detected with the General definition (command-lines available in Text S1) co-occurred with CRISPR arrays as is the case in all fully functional described systems. We searched for CRISPR-arrays with [17] as described in [27] and found that 88% of the detected Cas systems are close (<1kb, same result for <5kb) to a CRISPR-array and that 98% are present in a replicon containing at least one CRISPR-array. The absence of CRISPR in so few Cas-containing genomes suggests the method has a low rate of false positives. Secondly, we took from the literature the list of CRISPR-Cas with experimentally characterized *in vivo* effects [28]. In this list we could detect 100% of the 25 known Cas systems of genomes included in our dataset (Table S4) with our “general”, “typing” and “subtyping” models (see command-lines in Text S1). Furthermore, we could assign the correct subtype to 23 of them, and we propose a subtype for the system of *Mycoplasma gallisepticum*. This suggests a low rate of false negatives. Altogether these results suggest the method is very accurate and that most clusters correspond to CRISPR-Cas systems. Type I systems are more abundant in both bacteria (in ∼31% of the bacterial genomes) and archaea (∼71%), Type II are only found in bacteria, while Type III are more prevalent in archaea (∼38%) (Table 1 and Fig. S3). These results are consistent with previous analyses [25]. Subtypes I-C, I-E and I-F are more commonly found in bacteria, while subtypes I-A, I-B and I-D are frequent in archaea, as previously noted [26]. Overall, these results suggest that our models are able to accurately identify and type Cas systems. Profiles and models for the “Cas-Finder” are provided in Data S2. Users can easily add or remove components and change the genetic organization specifications.

**Table 1:** Taxonomic distribution of CRISPR-Cas types and sub-types in prokaryotes expressed in number and percentage of the genomes harboring the systems.

Typing	Type I							Type II			Type III			Type U
Bacteria	769 (31%)							177 (7%)			222 (9%)			7
Archaea	113 (71%)							0			60 (38%)			0
**Subtyping**	**I-A**	**I-B**	**I-C**	**I-D**	**I-E**	**I-F**	**I-U**	**II-A**	**II-B**	**II-U**	**III-A**	**III-B**	**III-U**	**U**
Bacteria	16 (<1%)	173 (7%)	196 (8%)	31 (1%)	282 (11%)	111 (5%)	47 (2%)	81 (3%)	5 (<1%)	93 (4%)	123 (5%)	112 (5%)	3 (<1%)	7 (<1%)
Archaea	55 (35%)	48 (30%)	2 (<1%)	16 (10%)	5 (3%)	0	2 (1%)	0	0	0	28 (18%)	40 (25%)	0	0

## Discussion

The use of MacSyFinder will often involve preliminary steps to model the biological systems of interest. This allows the researcher to produce structured knowledge and is particularly useful when these systems have distinguishable traits, such as a specific genetic architecture. Often there are few studies suggesting the parameters to use in the models. Under these circumstances, one should start with very simple models, *e.g.*, noting all components as *accessory* and using low quorums. The analysis of the results of these preliminary models often provides important clues on how to produce more complex and accurate models. For example, by relaxing the criteria of the requirements to identify type III secretion systems (T3SS) we were recently able to identify a new homologous system in *Myxobacteria* [4]. Modelling itself can thus lead to new biological findings.

MacSyFinder ignores phylogenetic information when putting together components of systems scattered in a replicon or in unordered datasets. In contrast, the preliminary distinction between homologous proteins can often be done using MacSyFinder without the need for lengthy phylogenetic analyses. This works in two steps. First, one must produce a multiple alignment gathering the different families of homologous proteins. This alignment must be divided into sub-alignments according to the different systems, leading to the production of different profiles for the different sub-families of homologs. Finally, and as a rule, for a given protein, the best-scoring profile corresponds to the relevant homologous family (see Fig. 5, and Fig. 2 in [4]).

It is difficult to estimate *a priori* how accurate MacSyFinder will be for any given biological system because this will depend on several system-specific variables. First, it will depend on the number of components of the system, their frequency in the system and their degree of sequence conservation. Systems with many highly conserved and frequent components will be much easier to identify than systems with many infrequent and fast-evolving components. Second, it will depend on the existence of other systems sharing homologous components. Systems including many components with homologs in other systems will be harder to identify. We have shown MacSyFinder can type CRISPR-Cas systems, even if they share homologs. Hence, even in these difficult situations MacSyFinder provides accurate models. The situation is necessarily more complicated when identifying systems with many homologs encoded by genes scattered in the genomes. In this case, phylogenetic methods may help in the reconstruction of the different systems.

Considering MacSyFinder's running time, the limiting step is usually the identification of hits by Hmmer, which is currently very efficient [13]. To speed up this step, MacSyFinder is able to compute and analyse Hmmer hits in parallel. MacSyFinder and its companion MacSyView are easy to install standalone tools. This is an advantage when it is necessary to keep the data private or when projects are so large that network transfer time is prohibitive. MacSyFinder was built to be simple to use. It is thus ideal for biologists without extensive knowledge of programming or scripting wishing to unravel the diversity of certain systems or to annotate genetic data. Often, bioinformaticians produce methods to identify machineries and would like to easily package them for reproducibility and distribution among biologists. This can be easily done with MacSyFinder *via* the distribution of XML files and the relevant protein profiles. The “Cas-finder” we present here is a particularly relevant case. At the time we started the project, there was public information available on the protein profiles and on the genetic organization of the systems. We only had to define the models and use them in such a way that we could identify the systems and class them. The result is a highly accurate application to identify *cas* operons that can be easily distributed (Data S2).

## Supporting Information

Figure S1 **Genomic architecture and taxonomic distribution of detected** ***cas*** **genes clusters (general model).** A. Distribution of the number of different genes in detected clusters. B. Distribution of the maximal distance between two components observed in each detected cluster. C. Boxplot of the number of different genes in each cluster vs. the maximal distance between two components observed in each cluster. D. Proportion of bacterial and archaeal genomes without (cluster−) and with at least one cluster of *cas* genes (cluster+).
(EPS)

Figure S2 **Schematic and simplified representation of the subtype models.** Each box corresponds to a *cas* gene family and the name of the corresponding HMM protein profiles are listed below. Some *cas* gene families have multiple HMM profiles available in the TIGRFAM database. Each *cas* gene family has its boxes filled (subtype non-specific) or surrounded (subtype-specific) by a distinct colour. Only the main *cas* gene families are represented. For full subtype models, see the XML files in Data S2.
(EPS)

Figure S3 **Taxonomic distribution of the three CRISPR-Cas systems types.** For each clade, the number of representative genomes is given, along with bar plots showing the percentage of these genomes containing the three types of CRISPR-Cas systems.
(EPS)

Table S1 **List of HMM profiles used for the “Cas-Finder”.**
(XLS)

Table S2 **Impact of the co-localization parameter on the detection.**
(PDF)

Table S3 **Detection results.**
(PDF)

Table S4 **Validation of the CRISPR-Cas systems detection on systems with** ***in vivo*** **effects listed in the review by Bondy-Denomy et. al 2014** [28] **.**
(PDF)

Text S1 **Supporting text (PDF file).**
(PDF)

File S1 **MacSyFinder's documentation file (PDF file).**
(PDF)

Data S1 **The MacSyFinder/MacSyView package (compressed tarball archive).**
(GZ) Click here for additional data file.

Data S2 **The Cas-Finder: models and profiles (compressed tarball archive).**
(ZIP)
