# Supplementary material for: MacSyFinder: A Program to Mine Genomes for Molecular Systems with an Application to CRISPR-Cas Systems
Source: PLoS One. 2014 Oct 17;9(10):e110726. doi: 10.1371/journal.pone.0110726 (PMC4201578; doi:10.1371/journal.pone.0110726)
Supplement: Table S2 — Impact of the co-localization parameter on the detection. (PDF) [file pone.0110726.s005.pdf]

Table S2. Impact of the co-localization parameter on the detection.

inter_gene_max_space (D)	N_components detected (known Cas proteins)	N_clusters detected	% large clusters > 13 genes	Maximal cluster size (N_total_genes)
**4**	10615	1638	6%	36
**5**	10663	1628	7%	36
**6**	10683	1613	8%	43
