# Supplementary material for: MacSyFinder: A Program to Mine Genomes for Molecular Systems with an Application to CRISPR-Cas Systems
Source: PLoS One. 2014 Oct 17;9(10):e110726. doi: 10.1371/journal.pone.0110726 (PMC4201578; doi:10.1371/journal.pone.0110726)
Supplement: Table S3 — Detection results. (PDF) [file pone.0110726.s006.pdf]

Table S3. Detection results.

	N_Definitions		N_Components	N_Systems
General	1	General	10663	1628
Typing	5	Type I	7579	1100
		Type II	624	181
		Type III	2047	340
		Type U	27	7
		CAS*	66	20
	TOTAL	Typing	10343	1648
SubTyping	7	Type I-A	686	80
		Type I-B	1837	264
		Type I-C	1320	202
		Type I-D	340	50
		Type I-E	2227	298
		Type I-F	631	113
		Type I-U	203	54
	TOTAL		7244	1061
	3	Type II-A	320	81
		Type II-B	18	5
		Type II-U*	283	94
	TOTAL		621	180
	3	Type III-A	1057	155
		Type III-B	943	171
		Type III-U	9	3
	TOTAL		2009	329
	1	Type U	27	7
	1	CAS*	80	24
	TOTAL	SubTyping	9981	1601

CAS*: detected clusters containing only 3 of the Cas1, 2, 4 and 6 proteins, and no gene signature.

Type II-U*: detected clusters containing only Cas1, Cas2 and Cas9 recently proposed as Type II-C [1]. Because they are difficult to distinguish with degraded Type II-A or Type II-B systems, we preferred classify them as Type II-U (U for unclassified).

References

1. Chylinski K, Le Rhun A, Charpentier E (2013) The tracrRNA and Cas9 families of type II CRISPR-Cas immunity systems. RNA Biol 10: 726-737.
