# Supplementary material for: MacSyFinder: A Program to Mine Genomes for Molecular Systems with an Application to CRISPR-Cas Systems
Source: PLoS One. 2014 Oct 17;9(10):e110726. doi: 10.1371/journal.pone.0110726 (PMC4201578; doi:10.1371/journal.pone.0110726)
Supplement: Table S4 — Validation of the CRISPR-Cas systems detection on systems with in vivo effects listed in the review by Bondy-Denomy et. al 2014 [28] . (PDF) [file pone.0110726.s007.pdf]

## Table S4. Validation of the CRISPR-Cas systems detection on systems with *in vivo* effects listed in the review by Bondy-Denomy et. al 2014 (Table 1) [1]

| Bacterial species | Type | References | Detection |
|---|---|---|---|
| *Campylobacter jejuni* | II-C | [2] | YES* |
| *Clostridium difficile* | I-B | [3] | YES |
| *Enterococcus sp.* | II-A | [4] | YES |
| *Escherichia coli* | I-E, I-F | [5-7] | YES |
| *Francisella tularensis* subsp. *novicida* | II-B | [8,9] | YES |
| *Haloferax volcanii* | I-B | [10] | YES |
| *Mycoplasma gallisepticum* | II | [11] | YES - One II-U detected |
| *Neisseria meningitidis* | II-C | [12] | YES* |
| *Pectobacterium atrosepticum* | I-F | [13] | YES |
| *Porphyromonas gingivalis* | I-C, III-B | [14] | YES |
| *Pseudomonas aeruginosa* | I-F | [15,16] | YES |
| *Salmonella enterica* serovar Typhimurium | I-E | [17] | YES |
| *Staphylococcus epidermidis* | III-A | [18] | YES |
| *Streptococcus agalactiae* | II-A | [19] | YES |
| *Streptococcus pyogenes* | II-A, I-C | [20,21] | YES |
| *Streptococcus thermophilus* | II-A | [22] | YES |
| *Sulfolobus* spp. | I-A | [23-25] | YES |
| *Thermococcus kodakarensis* | I-A, I-B | [26] | YES |
| *Xanthomonas oryzae* | I-C | [27] | YES |
| *Yersinia pestis* | I-F | [28] | YES |
| *Erwinia amylovora* | I-E | [29] | YES§ |
| *Lactococcus lactis* | III-A | [30] | NA - Plasmids not in our dataset |
| *Leptospirillum* group II | III | [31,32] | NA - Environmental sample |
| *Vibrio cholerae* | I-F | [33] | NA - *V. cholerae* variant not in our dataset |

NA - Not applicable because the data were not in our genomic dataset; green "Detection" cells mean the systems were detected. *Type II-C: detected clusters containing only Cas1, Cas2 and Cas9 recently proposed as Type II-C [34]. Because they are difficult to distinguish with degraded Type II-A or Type II-B systems, we preferred classify them as Type II-U (U for unclassified). §In the paper by Bondy-Denomy et. al 2014 [1] (Table 1), a type I-F is attributed to *Erwinia amylovora* based on a publication by Rezzonico et. al. 2011 [29]. However in this paper, the Type I-F was described in *E. pyrifoliae* and *tasmaniensis*, not in *E. amylovora* (Figure 1).

## References

1. Bondy-Denomy J, Davidson AR (2014) To acquire or resist: the complex biological effects of CRISPR-Cas systems. Trends Microbiol 22: 218-225.
2. Dugar G, Herbig A, Forstner KU, Heidrich N, Reinhardt R, et al. (2013) High-resolution transcriptome maps reveal strain-specific regulatory features of multiple Campylobacter jejuni isolates. PLoS Genet 9: e1003495.
3. Soutourina OA, Monot M, Boudry P, Saujet L, Pichon C, et al. (2013) Genome-wide identification of regulatory RNAs in the human pathogen Clostridium difficile. PLoS Genet 9: e1003493.
4. Palmer KL, Gilmore MS (2010) Multidrug-resistant enterococci lack CRISPR-cas. MBio 1.
5. Almendros C, Guzman NM, Diez-Villasenor C, Garcia-Martinez J, Mojica FJ (2012) Target motifs affecting natural immunity by a constitutive CRISPR-Cas system in Escherichia coli. PLoS ONE 7: e50797.

6. Touchon M, Charpentier S, Clermont O, Rocha EP, Denamur E, et al. (2011) CRISPR distribution within the Escherichia coli species is not suggestive of immunity-associated diversifying selection. J Bacteriol 193: 2460-2467.
7. Westra ER, Pul U, Heidrich N, Jore MM, Lundgren M, et al. (2010) H-NS-mediated repression of CRISPR-based immunity in Escherichia coli K12 can be relieved by the transcription activator LeuO. Mol Microbiol 77: 1380-1393.
8. Schunder E, Rydzewski K, Grunow R, Heuner K (2013) First indication for a functional CRISPR/Cas system in Francisella tularensis. International journal of medical microbiology : IJMM 303: 51-60.
9. Sampson TR, Saroj SD, Llewellyn AC, Tzeng YL, Weiss DS (2013) A CRISPR/Cas system mediates bacterial innate immune evasion and virulence. Nature 497: 254-257.
10. Fischer S, Maier LK, Stoll B, Brendel J, Fischer E, et al. (2012) An archaeal immune system can detect multiple protospacer adjacent motifs (PAMs) to target invader DNA. The Journal of biological chemistry 287: 33351-33363.
11. Delaney NF, Balenger S, Bonneaud C, Marx CJ, Hill GE, et al. (2012) Ultrafast evolution and loss of CRISPRs following a host shift in a novel wildlife pathogen, Mycoplasma gallisepticum. PLoS Genet 8: e1002511.
12. Zhang Y, Heidrich N, Ampattu BJ, Gunderson CW, Seifert HS, et al. (2013) Processing-independent CRISPR RNAs limit natural transformation in Neisseria meningitidis. Mol Cell 50: 488-503.
13. Vercoe RB, Chang JT, Dy RL, Taylor C, Gristwood T, et al. (2013) Cytotoxic chromosomal targeting by CRISPR/Cas systems can reshape bacterial genomes and expel or remodel pathogenicity islands. PLoS Genet 9: e1003454.
14. Watanabe T, Nozawa T, Aikawa C, Amano A, Maruyama F, et al. (2013) CRISPR regulation of intraspecies diversification by limiting IS transposition and intercellular recombination. Genome Biol Evol 5: 1099-1114.
15. Cady KC, Bondy-Denomy J, Heussler GE, Davidson AR, O'Toole GA (2012) The CRISPR/Cas adaptive immune system of Pseudomonas aeruginosa mediates resistance to naturally occurring and engineered phages. J Bacteriol 194: 5728-5738.
16. Bondy-Denomy J, Pawluk A, Maxwell KL, Davidson AR (2013) Bacteriophage genes that inactivate the CRISPR/Cas bacterial immune system. Nature 493: 429-432.
17. Medina-Aparicio L, Rebollar-Flores JE, Gallego-Hernandez AL, Vazquez A, Olvera L, et al. (2011) The CRISPR/Cas immune system is an operon regulated by LeuO, H-NS, and leucine-responsive regulatory protein in Salmonella enterica serovar Typhi. J Bacteriol 193: 2396-2407.
18. Marraffini LA, Sontheimer EJ (2008) CRISPR interference limits horizontal gene transfer in staphylococci by targeting DNA. Science 322: 1843-1845.
19. Lopez-Sanchez MJ, Sauvage E, Da Cunha V, Clermont D, Ratsima Hariniaina E, et al. (2012) The highly dynamic CRISPR1 system of Streptococcus agalactiae controls the diversity of its mobilome. Mol Microbiol 85: 1057-1071.
20. Deltcheva E, Chylinski K, Sharma CM, Gonzales K, Chao Y, et al. (2011) CRISPR RNA maturation by trans-encoded small RNA and host factor RNase III. Nature 471: 602-607.
21. Nozawa T, Furukawa N, Aikawa C, Watanabe T, Haobam B, et al. (2011) CRISPR inhibition of prophage acquisition in Streptococcus pyogenes. PLoS ONE 6: e19543.
22. Barrangou R, Fremaux C, Deveau H, Richards M, Boyaval P, et al. (2007) CRISPR provides acquired resistance against viruses in prokaryotes. Science 315: 1709-1712.
23. Manica A, Zebec Z, Teichmann D, Schleper C (2011) In vivo activity of CRISPR-mediated virus defence in a hyperthermophilic archaeon. Mol Microbiol 80: 481-491.
24. Gudbergsdottir S, Deng L, Chen Z, Jensen JV, Jensen LR, et al. (2011) Dynamic properties of the Sulfolobus CRISPR/Cas and CRISPR/Cmr systems when challenged with vector-borne viral and plasmid genes and protospacers. Mol Microbiol 79: 35-49.
25. Erdmann S, Garrett RA (2012) Selective and hyperactive uptake of foreign DNA by adaptive immune systems of an archaeon via two distinct mechanisms. Mol Microbiol 85: 1044-1056.
26. Elmore JR, Yokooji Y, Sato T, Olson S, Glover CV, 3rd, et al. (2013) Programmable plasmid interference by the CRISPR-Cas system in Thermococcus kodakarensis. RNA Biol 10: 828-840.
27. Semenova E, Nagornykh M, Pyatnitskiy M, Artamonova, II, Severinov K (2009) Analysis of CRISPR system function in plant pathogen Xanthomonas oryzae. FEMS Microbiol Lett 296: 110-116.
28. Cui Y, Li Y, Gorge O, Platonov ME, Yan Y, et al. (2008) Insight into microevolution of Yersinia pestis by clustered regularly interspaced short palindromic repeats. PLoS ONE 3: e2652.
29. Rezzonico F, Smits TH, Duffy B (2011) Diversity, evolution, and functionality of clustered regularly interspaced short palindromic repeat (CRISPR) regions in the fire blight pathogen Erwinia amylovora. Appl Environ Microbiol 77: 3819-3829.

30. Millen AM, Horvath P, Boyaval P, Romero DA (2012) Mobile CRISPR/Cas-mediated bacteriophage resistance in Lactococcus lactis. PLoS ONE 7: e51663.
31. Tyson GW, Banfield JF (2008) Rapidly evolving CRISPRs implicated in acquired resistance of microorganisms to viruses. Environ Microbiol 10: 200-207.
32. Andersson AF, Banfield JF (2008) Virus population dynamics and acquired virus resistance in natural microbial communities. Science 320: 1047-1050.
33. Seed KD, Lazinski DW, Calderwood SB, Camilli A (2013) A bacteriophage encodes its own CRISPR/Cas adaptive response to evade host innate immunity. Nature 494: 489-491.
34. Chylinski K, Le Rhun A, Charpentier E (2013) The tracrRNA and Cas9 families of type II CRISPR-Cas immunity systems. RNA Biol 10: 726-737.
