# Supplementary material for: MacSyFinder: A Program to Mine Genomes for Molecular Systems with an Application to CRISPR-Cas Systems
Source: PLoS One. 2014 Oct 17;9(10):e110726. doi: 10.1371/journal.pone.0110726 (PMC4201578; doi:10.1371/journal.pone.0110726)
Supplement: Text S1 — Supporting text (PDF file). (PDF) [file pone.0110726.s008.pdf]

Text S1. Supporting text

Object-oriented implementation

MacSyFinder is coded in Python using object-oriented programming. The main classes are described in this section and a full description of the API is provided in the documentation (File S1). The *System* class models the molecular systems and contains a list of instances of the *Gene* class, which models each component of a given *System*. The *Homolog* class (resp. *Analog*) encapsulates a *Gene* and models relationships of homology (resp. analogy) between components. *Gene* refers to an instance of the *Profile* object that corresponds to a Hidden Markov model (HMM) protein profile that is used for similarity search purpose. The *Config* class handles the program parameters, including Hmmer search parameters, and the set of sequences to query (represented by the *Database* object). The *Database* stores information on the dataset, including necessary information to detect systems in both linear and circular chromosomes, and when suitable, data for graphical representation of *System* instances in their genomic context (see documentation in File S1). A set of parsers and object factories are used to fill the objects from command-line and input files (*i.e.*, the optional configuration file and the XML files describing the systems, File S1), and to ensure their uniqueness and integrity. Once these objects are initialized and the detection is launched, Hmmer is executed on the sequences of the database (optionally in parallel) with a unique list of profiles corresponding to the systems to detect. Subsequently, Hmmer output files are parsed, and selected hits (given the search parameters provided) are used to fill *Hit* objects, which contain information for the detection of the systems. During the treatment of the *Hits* for *Systems* detection, the occurrences of the systems (*SystemOccurence* objects) are filled, and the decision rules associated with the systems (quorum and co-localization) are applied.

Dependencies of MacSyView

MacSyView was coded in Javascript and uses third-party libraries that are included in the package, and accredited in the COPYRIGHT file. It includes among others the Raphael library for systems drawing, the Bootstrap library for HTML design and the Mustache library for HTML templating in Javascript.

CRISPR-Cas type and subtype assignment: command-lines

The command-lines below were used to run the analyses described in the Application section ("all" means that all systems defined in the definition folders will be searched). In these commands "CASprofiles" is the directory containing the protein profiles. "DEF-General", "DEF-Typing" and "DEF-SubTyping" are the directories containing respectively the system models for the General, Typing, and Subtyping levels of classification.

General model:

```
macsyfinder -w 20 -d DEF-General/ -p CASprofiles/ --db-type gembase --sequence-db alltogether.prot --topology-file alltogether.topology all
```

Typing models:

```
macsyfinder -w 20 -d DEF-Typing/ -p CASprofiles/ --db-type gembase --sequence-db alltogether.prot --topology-file alltogether.topology all
```

Subtyping models:

```
macsyfinder -w 20 -d DEF-SubTyping/ -p CASprofiles/ --db-type gembase --sequence-db alltogether.prot --topology-file alltogether.topology all
```

Specificity of the protein profiles for typing and subtyping CRISPR-Cas systems

The specificity of the 53 protein profiles constructed by [1] are consistent with those previously described in [2], with a few exceptions. Two profiles previously described as Type I-A specific (TIGR01908 and TIGR02670) profiles, were mostly found in Type I-B. TIGR02582, mostly found in Type III-A, is also found in some Type I-B. TIGR03641 and TIGR04093, characteristic of Type I-B and I-D respectively, are found in some Type III. Profiles of Type III-U are often associated with other systems, especially Type III-A, III-B and I-B. We found that, as expected, the 36 additional HMM profiles available in TIGRFAM database (Materials and Methods) are more specific to given subtypes. For instance, while the profile TIGR00287 matches Cas1 protein of the three types, new profiles can distinguish the Cas1 protein of the six different subtypes I (Table S1 and Fig. 5).

References

1. Haft DH, Selengut J, Mongodin EF, Nelson KE (2005) A guild of 45 CRISPR-associated (Cas) protein families and multiple CRISPR/Cas subtypes exist in prokaryotic genomes. PLoS Comput Biol 1: e60.
2. Makarova KS, Haft DH, Barrangou R, Brouns SJ, Charpentier E, et al. (2011) Evolution and classification of the CRISPR-Cas systems. Nat Rev Microbiol 9: 467-477.
