# Supplementary material for: MacSyFinder: A Program to Mine Genomes for Molecular Systems with an Application to CRISPR-Cas Systems
Source: PLoS One. 2014 Oct 17;9(10):e110726. doi: 10.1371/journal.pone.0110726 (PMC4201578; doi:10.1371/journal.pone.0110726)
Supplement: File S1 — MacSyFinder's documentation file (PDF file). (PDF) [file pone.0110726.s009.pdf]

MacSyFinder Documentation

Release 1.0.0-RC3

Sophie Abby, Bertrand Néron

September 11, 2014

CONTENTS

I Running MacSyFinder **3**

1 Installation **5**
1.1 MacSyFinder dependencies . . . 5
1.2 Installation procedure . . . 5
1.3 Uninstalling MacSyFinder . . . 6

2 MacSyFinder Quick Start **7**
2.1 First trial with a test dataset . . . 7
2.2 Visualizing expected results with MacSyView . . . 8

3 Input and Options of MacSyFinder **9**
3.1 Input dataset . . . 9
3.2 Command-line options . . . 9
3.3 Configuration file . . . 12
3.4 In-house input files . . . 14

4 Output format **17**
4.1 Hmmer results outputs . . . 17
4.2 Systems detection results . . . 17
4.3 Logs and configuration files . . . 19
4.4 File for MacSyview: results.macsyfinder.json . . . 19

5 MacSyView: visualizing MacSyFinder's results! **21**
5.1 MacSyView How To . . . 21
5.2 Graphical output description . . . 21
5.3 Technology used . . . 22
5.4 Screenshot . . . 22

II MacSyFinder functioning **25**

6 Macromolecular systems definition **27**
6.1 Principles . . . 27
6.2 The XML hierarchy . . . 28

7 MacSyFinder implementation overview **31**
7.1 The System object . . . 32
7.2 The Gene object . . . 32
7.3 The Profile object . . . 33
7.4 Reporting Hmmer search results . . . 33

8 MacSyFinder functioning 35
8.1 Functioning overview 35
8.2 A. Searching for systems' components 35
8.3 B. Assessing systems presence 36

III MacSyFinder API documentation 39

9 Configuration API 41
9.1 Config API reference 41

10 Database API 47
10.1 database API reference 47

11 System API 51
11.1 SystemBank API reference 51
11.2 System API reference 52

12 The Parser of Systems definitions 55
12.1 SystemParser API reference 56

13 Gene API 59
13.1 GeneBank API reference 59
13.2 Gene API reference 60
13.3 Homolog API reference 63
13.4 Analog API reference 63

14 Profile API 65
14.1 ProfileFactory API reference 65
14.2 Profile API reference 65

15 HMMReport API 67
15.1 HMMReport API reference 67
15.2 GeneralHMMReport API reference 69
15.3 OrderedHMMReport 69
15.4 GembaseHMMReport 69
15.5 Hit 69

16 search_genes API reference 71

17 search_systems API reference 73

18 MacSyFinder errors 83
18.1 macsypy_error API reference 83

IV Indices and tables 85

Python Module Index 89

Index 91

MacSyFinder is a program to model and detect macromolecular systems, genetic pathways... in protein datasets. In prokaryotes, these systems have often evolutionarily conserved properties: they are made of conserved components, and are encoded in compact loci (conserved genetic architecture). The user models these systems with MacSyFinder to reflect these conserved features, and to allow their efficient detection.

Criteria for systems detection include **component content (quorum)**, and **genomic co-localization**. Each component corresponds to a hidden Markov model (HMM) protein profile to perform homology searches with the program Hmmer.

In order to model macromolecular systems, the user:

- builds or gather from databanks **HMM protein profiles** for components of interest,
- defines **decision rules** for each system in a dedicated XML grammar (see *Macromolecular systems definition*).

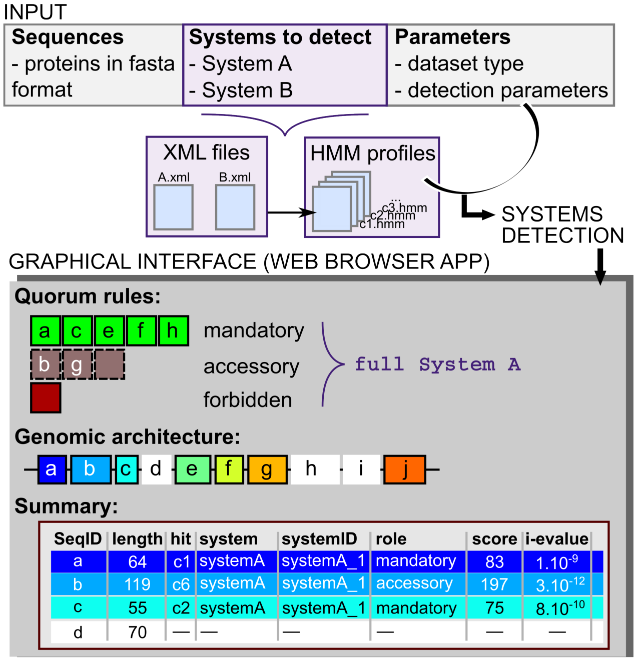

Part I

Running MacSyFinder

CHAPTER

ONE

INSTALLATION

1.1 MacSyFinder dependencies

MacSyFinder has two dependencies:

- the *formatdb* (>=2.2.26) or *makeblastdb* (>=2.2.28) tools provided with the Blast suite of programs

(**http://blast.ncbi.nlm.nih.gov/Blast.cgi?CMD=Web&PAGE_TYPE=BlastDocs&DOC_TYPE=Download**)

- the program *Hmmer* version 3.0 (http://hmmer.janelia.org/).

formatdb or *makeblastdb* and *hmmsearch* programs should be installed (*e.g.*, in the PATH) in order to use MacSyFinder. Otherwise, the paths to these executables must be specified in the command-line: see the *command-line options*.

Python version 2.7 is required to run MacSyFinder: https://docs.python.org/2.7/index.html

1.2 Installation procedure

First unarchive the source codes package, and enter the extracted folder:

```
tar -xzvf macsyfinder-x.x.tar.gz
cd macsyfinder-x.x
```

MacSyFinder installation follows classical "pythonic" installation procedure (see http://docs.python.org/2/install/):

```
python setup.py build
(sudo) python setup.py install
```

It is **highly recommanded** to run tests before performing the full installation:

```
python setup.py build
python setup.py test -vv
(sudo) python setup.py install
```

Note: Super-user privileges (*i.e.*, `sudo`) are necesserary if you want to install the program in the general file architecture.

Note: If you do not have the privileges, or if you do not want to install MacSyFinder in the Python libraries of your system, you can install MacSyFinder in a virtual environment (http://www.virtualenv.org/).

Procedures specific to MacSyFinder can be used instead of default. Please run the command for full options:

```
python setup.py --help
```

The main ones are:

```
python setup.py install --prefix /usr/local/home/bob/my_programs # Specifies an installation path
```

=> It will install MacSyFinder and required data (profiles folder and systems definition folders) in the Home directory of "bob", in the "my_progams" folder (useful if you do not have super-user privileges).

> **Warning:** When installing a new version of MacSyFinder, do not forget to uninstall the previous version installed !

1.3 Uninstalling MacSyFinder

To uninstall MacSyFinder (the last version installed), run:

```
(sudo) python setup.py uninstall
```

CHAPTER

TWO

MACSYFINDER QUICK START

In order to run MacSyFinder on your favorite dataset as soon as you have installed it, you can simply follow the next steps:

- Type: "`macsyfinder -h`" to see all options available. All command-line options are described in the *Command-line options section*.
- On a "metagenomic" dataset for example:

  "`macsyfinder --db-type unordered --sequence-db metagenome.fasta all`" will detect all systems modelled in .xml files placed in the default definition folder in a metagenomic dataset.

  "`macsyfinder --db-type unordered --sequence-db metagenome.fasta -d mydefinitions/ all`" will detect all systems modelled in .xml files placed in the *"mydefinitions"* folder.
- On a completely assembled genome (where the gene order is known, and is relevant for systems detection):

  "`macsyfinder --db-type ordered-replicon --sequence-db mygenome.fasta -d mydefinitions/ SystemA SystemB`" will detect the systems *"SystemA"* and *"SystemB"* in a complete genome from *"SystemA.xml"* and *"SystemB.xml"* definition files placed in the folder *"mydefinitions"*.

See *Input dataset* for more on input datasets.

Note: Systems have to be spelled in a case-sensitive way to run their detection from the command-line. The name of the system corresponds to the suffix defined for xml files (.xml by default), for example *"toto"* for a system defined in *"toto.xml"*.

The *"all"* keyword allows to detect all systems available in the definition folder in a single run. See the *Command-line options*.

2.1 First trial with a test dataset

We included a test dataset in the MacSyFinder package. **By default, it will be installed** in /share/macsyfinder or /usr/share/macsyfinder. But it can be located elsewhere if it was specified during installation.

This dataset consists in the detection of CRISPR-Cas SubTypes with the definitions in the /share/macsyfinder/DEF folder, using the profiles in the /share/macsyfinder/profiles folder. This classification was previously described in Makarova et al. 2011, and the profiles are from the TIGRFAM database (release 13 of August 15 2012) and some of them were specifically designed for CRISPR-Cas classification (Haft et. al, 2005). The definitions are detailed in the MacSyFinder's paper.

As a sequence dataset, we propose three replicons in /share/macsyfinder/sequence_data/datatest_gembase.fasta:

- *Escherichia coli* str. K-12 substr. MG1655 chromosome (ESCO001c01a). Genbank accession number: NC_000913.
- *Haloarcula marismortui* ATCC 43049 plasmid pNG400 (HAMA001p04a). Genbank accession number: NC_006392.
- *Legionella pneumophila* str. Paris, complete genome (LEPN003c01a). Genbank accession number: NC_006368.

They were concatenated in a single fasta file, following the "gembase" format proposed *here*, and thus MacSyfinder will treat the three different replicons separately for systems inference.

To run the detection and classification of all subtypes, type:

```
"macsyfinder --db-type gembase --sequence-db
/share/macsyfinder/sequence_data/datatest_gembase.fasta all"
```

To run the detection of the Type-IE subtype only, type:

```
"macsyfinder --db-type gembase --sequence-db
/share/macsyfinder/sequence_data/datatest_gembase.fasta CAS-TypeIE"
```

A sample topology file is included /share/macsyfinder/sequence_data/datatest_gembase.topology, and follows the convention in *here*. It allows to specify a different topology "linear" or "circular" for each replicon in the "gembase" format. Otherwise, by default the topology is set to "circular". It can also be specified in the commmand-line (see the *Command-line options*).

To run the detection using the topology file, type:

```
"macsyfinder --db-type gembase --sequence-db
/share/macsyfinder/sequence_data/datatest_gembase.fasta
--topology-file /share/macsyfinder/sequence_data/datatest_gembase.topology all"
```

2.2 Visualizing expected results with MacSyView

To have an idea of what should be detected with the above test dataset, run *MacSyView*, the web-browser application for MacSyFinder's results visualization. To do that, open the expected JSON result file with MacSyView: /share/macsyfinder/sequence_data/results.macsyfinder.json.

A screenshot of MacSyView is included *here*.

CHAPTER

THREE

INPUT AND OPTIONS OF MACSYFINDER

3.1 Input dataset

The input dataset must be a set of protein sequences in **Fasta format** (see http://en.wikipedia.org/wiki/FASTA_format).

The *base section* in the configuration file (see *Configuration file*) can be used to specify **the path** and the **type of dataset** to deal with, as well as the *–sequence_db* and *–db_type* parameters respectively, described in the *Command-line options* (see *Input options*).

Four types of protein datasets are supported:

- *unordered* : a set of sequences (*e.g.* a metagenomic dataset)
- *unordered_replicon* : a set of sequences corresponding to a complete genome (*e.g.* an unassembled complete genome)
- *ordered_replicon* : a set of sequences corresponding to an ordered complete replicon (*e.g.* an assembled complete genome)
- *gembase* : a set of multiple ordered replicons, which format follows the convention described in *Gembase format*.

For "ordered" ("ordered_replicon" or "gembase") datasets only, MacSyFinder can take into account the **shape of the genome**: "linear", or "circular" for detection. The default is set to "circular".

This can be set with the *–replicon_topology* parameter from *Command-line options* (see *Input options*), or in the configuration in the *base section*.

With the "gembase" format, it is possible to specify a topology per replicon with a topology file (see *Gembase format* and *Topology files*).

3.2 Command-line options

Positional arguments:

```
systems                 The systems to detect. This is an obligatory option
                        with no keyword associated to it. To detect all systems
                        described in .xml available, set to "all" (case insensitive).
                        Otherwise, a single or multiple systems can be specified.
                        For example: "SystemA SystemB".
```

Optional arguments:

```
-h, --help            Show the help message and exit
```

Input dataset options:

```
--sequence-db SEQUENCE_DB
                      Path to the sequence dataset in fasta format.

--db-type {unordered_replicon,ordered_replicon,gembase,unordered}
                      The type of dataset to deal with. "unordered_replicon"
                      corresponds to a non-assembled genome, "unordered" to
                      a metagenomic dataset, "ordered_replicon" to an
                      assembled genome, and "gembase" to a set of replicons
                      where sequence identifiers follow this convention:
                      ">RepliconName_SequenceID"

--replicon-topology {linear,circular}
                      The topology of the replicons (this option is
                      meaningful only if the db_type is 'ordered_replicon'
                      or 'gembase'

--topology-file TOPOLOGY-FILE
                      Topology file path. The topology file allows to
                      specify a topology (linear or circular) for each
                      replicon (this option is meaningful only if the
                      db_type is 'ordered_replicon' or 'gembase'. A topology
                      file is a tabular file with two columns: the 1st is
                      the replicon name, and the 2nd the corresponding
                      topology: "RepliconA linear"

--idx                 Forces to build the indexes for the sequence dataset
                      even if they were presviously computed and present at
                      the dataset location (default = False)
```

Systems detection options:

```
--inter-gene-max-space SYSTEM VALUE
                      Co-localization criterion: maximum number of
                      components non-matched by a profile allowed between
                      two matched components for them to be considered
                      contiguous. Option only meaningful for 'ordered'
                      datasets. The first value must match a system name,
                      the second a number of components. This option can be
                      repeated several times:
                      "--inter-gene-max-space T3SS 12 --inter-gene-max-space Flagellum 20"

--min-mandatory-genes-required SYSTEM VALUE
                      The minimal number of mandatory genes required for
                      system assessment. The first value must correspond to
                      a system name, the second value to an integer. This
                      option can be repeated several times:
                      "--min-mandatory-genes-required T2SS 15
                      --min-mandatory-genes-required Flagellum 10"

--min-genes-required SYSTEM VALUE
                      The minimal number of genes required for system
                      assessment (includes both 'mandatory' and 'accessory'
                      components). The first value must correspond to a
                      system name, the second value to an integer. This
```

```
                      option can be repeated several times:
                      "--min-genes-required T2SS 15 --min-genes-required Flagellum 10"

--max-nb-genes SYSTEM VALUE
                      The maximal number of genes allowed in the system.

--multi-loci SYSTEM
                      Specifies if the system can be detected as a 'scattered'
                      system. (default: False)
```

Options for Hmmer execution and hits filtering:

```
--hmmer HMMER_EXE     Path to the Hmmer program.

--index-db INDEX_DB_EXE
                      The indexer to be used for Hmmer. The value can be
                      either 'makeblastdb' or 'formatdb' or the path to one
                      of these binary (default = makeblastb).

--e-value-search E_VALUE_RES
                      Maximal e-value for hits to be reported during Hmmer
                      search. (default = 1)

--i-evalue-select I_EVALUE_SEL
                      Maximal independent e-value for Hmmer hits to be
                      selected for system detection. (default = 0.001)

--coverage-profile COVERAGE_PROFILE
                      Minimal profile coverage required in the hit alignment
                      to allow the hit selection for system detection.
                      (default = 0.5)
```

Path options:

```
-d DEF_DIR, --def DEF_DIR
                      Path to the systems definition files.

-r RES_SEARCH_DIR, --res-search-dir RES_SEARCH_DIR
                      Path to the directory where to store MacSyFinder search
                      results directories.

--res-search-suffix RES_SEARCH_SUFFIX
                      The suffix to give to Hmmer raw output files.

--res-extract-suffix RES_EXTRACT_SUFFIX
                      The suffix to give to filtered hits output files.

-p PROFILE_DIR, --profile-dir PROFILE_DIR
                      Path to the profiles directory.

--profile-suffix PROFILE_SUFFIX
                      The suffix of profile files. For each 'Gene' element,
                      the corresponding profile is searched in the
                      'profile_dir', in a file which name is based on the
                      Gene name + the profile suffix. For instance, if the
                      Gene is named 'gspG' and the suffix is '.hmm3', then
                      the profile should be placed in the specified folder
                      'profile_dir' and be named 'gspG.hmm3'.
                      (default: ".hmm")
```

General options:

```
-w WORKER_NB, --worker WORKER_NB
                      Number of workers to be used by MacSyFinder. In the case
                      the user wants to run MacSyFinder in a multi-thread mode.
                      All workers can be used with the value '0'. (default = 1)

-v, --verbosity       Increases the verbosity level. There are 4 levels:
                      Error messages (default), Warning (-v), Info (-vv) and
                      Debug(-vvv).

--log LOG_FILE        Path to the directory where to store the 'macsyfinder.log'
                      log file.

--config CFG_FILE     Path to a putative MacSyFinder configuration file to be
                      used.

--previous-run PREVIOUS_RUN
                      Path to a previous MacSyFinder run directory. It allows to
                      skip the Hmmer search step on same dataset, as it uses
                      previous run results and thus parameters regarding
                      Hmmer detection. The configuration file from this
                      previous run will be used.
                      It is in conflict with options:
                      --config,
                      --sequence_db,
                      --profile-suffix,
                      --res-extract-suffix,
                      --e-value-res,
                      --db-type,
                      --hmmer
```

3.3 Configuration file

Options to run MacSyFinder can be specified in a configuration file. The *Config* handles all configuration options for MacSyFinder. Three locations are parsed to find configuration files:

- $PREFIX/etc/macsyfinder/macsyfinder.conf
- $(HOME)/.macsyfinder/macsyfinder.conf
- ./macsyfinder.conf

Moreover these three locations options can be passed on the command-line.

Each file can define options, at the end all options are added. If an option is specified several times:

Note: The precedence rules from the less important to the more important are:

$PREFIX/etc/macsyfinder/macsyfinder.conf < $(HOME)/.macsyfinder/macsyfinder.conf < ./macsyfinder.conf < "command-line" options

This means that command-line options will always bypass those from the configuration files. In the same flavor, options altering the definition of systems found in the command-line or the configuration file will always overwhelm values from systems' *XML definition files*.

The configuration files must follow the Python "ini" file syntax. The Config object provides some default values and performs some validations of the values, for instance:

In MacSyFinder, five sections are defined:

- **base** : all information related to the protein dataset under study
  - *file* : the path to the dataset in Fasta format (*no default value*)
  - *type* : the type of dataset to handle, four types are supported:
    - *unordered* : a set of sequences (*e.g.* a metagenomic dataset)
    - *unordered_replicon* : a set of sequences corresponding to a complete replicon (*e.g.* an unassembled complete genome)
    - *ordered_replicon* : a set of sequences corresponding to a complete replicon ordered (*e.g.* an assembled complete genome)
    - *gembase* : a set of multiple ordered replicons.

    (*no default value*)
  - *replicon_topology* : the topology of the replicon under study. Two topologies are supported: 'linear' and 'circular' (*default* = 'circular') This option will be ignored if the dataset type is not ordered (*i.e.* "unordered_replicon" or "unordered").
- **system**
  - *inter_gene_max_space* = list of system name and integer separated by spaces. These values will supersede the values found in the system definition file.
  - *min_mandatory_genes_required* = list of system name and integer separated by spaces. These values will supersede the values found in the system definition file.
  - *min_genes_required* = list of system name and integer separated by spaces. These values will supersede the values found in the system definition file.
- **hmmer**
  - *hmmer_exe* (default= *hmmsearch*)
  - *index_db_exe* the executable to use to build the index for the hmm. The value can be 'makeblastdb' or 'formatdb' or the absolute path toward one of these two binaries (default= *makeblastdb*)
  - *e_value_res* = (default= *1*)
  - *i_evalue_sel* = (default= *0.5*)
  - *coverage_profile* = (default= *0.5*)
- **directories**
  - *res_search_dir* = (default= *./datatest/res_search*)
  - *res_search_suffix* = (default= *.search_hmm.out*)
  - *profile_dir* = (default= *./profiles*)
  - *profile_suffix* = (default= *.fasta-aln_edit.hmm*)
  - *res_extract_suffix* = (default= *.res_hmm_extract*)
  - *def_dir* = (default= *./DEF/*)
- **general**

- ***log_level*: (default= *debug*) This corresponds to an integer code:**

Level	Numeric value
CRITICAL	50
ERROR	40
WARNING	30
INFO	20
DEBUG	10
NOTSET	0

- *log_file* = (default = macsyfinder.log in directory of the run)

Example of a configuration file:

```
 [base]
 prefix = /path/to/macsyfinder/home/
 file = %(prefix)s/dataset/prru_psae.001.c01.fasta
 type = gembase
 replicon_topology = circular

 [system]
 inter_gene_max_space = T2SS 22 Flagellum 44
 min_mandatory_genes_required = T2SS 6 Flagellum 4
 min_genes_required = T2SS 8 Flagellum 4

 [hmmer]
 hmmer_exe = hmmsearch
 index_db_exe = makeblastdb
 e_value_res = 1
 i_evalue_sel = 0.5
 coverage_profile = 0.5

 [directories]
 prefix = /path/to/macsyfinder/home/
 def_dir = %(prefix)s/data/DEF
 res_search_dir = %(prefix)s/dataset/res_search/
 res_search_suffix = .raw_hmm
 profile_dir = %(prefix)s/data/profiles
 profile_suffix = .fasta-aln.hmm
 res_extract_suffix = .res_hmm

[general]
log_level = debug
```

Note: After a run, the corresponding configuration file ("macsyfinder.conf") is generated as a (re-usable) output file that stores every options used in the run. It is stored in the results' directory (see *the output section*).

3.4 In-house input files

3.4.1 Gembase format

In order to allow the users running MacSyFinder on a bunch of genomes in a single run, we propose to adopt the following convention to fulfill the requirements for the "gembase db_type". It consists in providing for each protein, both the replicon name and a protein identifier separated by a "_" in the first field of fasta headers. For instance:

```
>PlasmidA_0001 YP_003225072.1_ | putative stcE protein
MKLKYLSCMILASLAMGAFAATAADNNSAIYFNTTQPVNDLQGGLAAEVK
FAQSQILSAHPKEGESQQHLTSLRKSLLLVRLVKADDKTPVQVEARDAND
KILGTLTLSPPSSLPDTVYHLDGVPADGIDFTPQNGTKKIINTVAEVNKL
SDASGSSIKSYLANNALVEIQTANGRWIRDMYLPQGAELEGKMVRFVSYA
GYNSTVFYGDRKVTLSVGNTLLFKYVNGQWFRSGELENNRIAYAQHTWSA
ELPAHWIVPGLNLVIKQGNLSGSLNDINVGAPGELLLHTIDIGMLTTPRG
RFDFAKDKEAHREYFQTIPVSRMIVNNYAPLHLKEVMLPTGTLLTDADPG
>PlasmidA_0002 YP_003225073.1_ | type II secretion protein EtpC
MLFFLSSRRDRNLFIKDIALKMLTPNWVLCVILLIAGYQLVSVIRHFWLT
PATSASDLSHVSVSETAVTDEHTEENFVFTLFGTASPPLSEGKVQKTTSS
LSDDLLSGGDLDVRGILYSSVTEHSVAIFAHNNRQFSLGIGEKVPGYDAT
ISAIFSDHIVINYQGKNASLPLRYDNPAKRNAQDDNNLIVGPVTTQANFR
VKNIFDIMSLSPVTVNNTLSGYRLSPGKASSLFYNAGLHDNDLAVLLNGS
ELRDTRQAKQIMKQLTELKEIKITVERDGQLYDAFIAVGEN
....
>ChromosomeA_0001 _YP_003573410.1_ | adhesin-like protein
MKKLFLFAALLMTGFAFYSCEDVVDNPAQDPAQSWNYSVSVKFADFDFNG
AVDENSVPYTYKAPTTLYVLNEENTLMGTITTDAAPAIGDYGTYAGTLTG
SIGNNLIITTKIGNDLTKQDGTLKSAIENGIVQTAEVPIKIYNANSGTLT
TASAKMDNTAAIAYTSLGYIKGGDKILFVEGNQTFEWTVNEEFDPYTSTD
LYIALPMNTDPETEYTISSDSKDGYTRGGTFKLADYPTLAAGKVSNYIGG
IPFIQTGVDLTKWDAYMRTDPNNTWYMNNINNGWPATFSQEVEDGKSFIV
TQSGPTLDSLNVVVGGVTGKEVNVTLNNIRLGKDRSINIGDKHGWVEYDG
THDIYGWGAKANVTLIGENECETLYIQCPATKKGEGTLNYKNLSIDSYGS
>ChromosomeA_0020 _YP_003573411.1_ | hypothetical protein
MKRIVLITLVSILTTFQAIAQVANGFYRVQNNASSRYITLRDNAVGTVDY
SSTNVDLSNIVTWSGFDKVKSNPASIIYVEQHDSKYDLKVQGTGIYAITG
GRTYLELRPKDSGYILAVTYNGMEGRLYDSEEDVDGEGYVKRSGNSAYQY
WSFIPVDTENNYIGLQPTVQVGDNYYGTLYASYPFKAASSGIKFYYVDAI
....
```

3.4.2 Topology files

To be able to attribute a topology per replicon/genome when using the Gembase format, we propose the user to build a "topology file" in the form of a tabular file with two columns separated by a ":". The 1st column is the replicon name, and the 2nd the corresponding topology. Comments can be written after a "#".

For example:

```
# comment line
PlasmidA : circular
ChromosomeA : linear
ChromosomeB : circular
```

Note: A topology file can be specified on the command-line with the "`--topology-file`" parameter.

CHAPTER

FOUR

OUTPUT FORMAT

MacSyFinder provides different types of outputs. At each run, MacSyFinder creates a new folder, whose name is based on a fixed prefix and a random suffix, for instance "macsyfinder-20130128_08-57-46". MacSyFinder outputs are stored in this run-specific folder.

4.1 Hmmer results outputs

Raw Hmmer outputs are provided, as long with processed tabular outputs that includes hits filtered as specified by the user. For instance, the Hmmer search for SctC homologs with the corresponding profile will produce as a result two files: "sctC.search_hmm.out" and "sctC.res_hmm_extract".

The processed output "sctC.res_hmm_extract" recalls on the first lines the parameters used for hits filtering and relevant information on the matches, as for instance:

```
# gene: sctC extract from /Users/bob/macsyfinder_results/
      macsyfinder-20130128_08-57-46/sctC.search_hmm.out hmm output
# profile length= 544
# i_evalue threshold= 0.001000
# coverage threshold= 0.500000
# hit_id replicon_name position_hit hit_sequence_length gene_name gene_system i_eval score
      profile_coverage sequence_coverage begin end
PSAE001c01_006940       PSAE001c01      3450    803     sctC    T3SS    1.1e-41 141.6
      0.588235  0.419676        395     731
PSAE001c01_018920       PSAE001c01      4634    776     sctC    T3SS    9.2e-48 161.7
      0.976103  0.724227        35      596
PSAE001c01_031420       PSAE001c01      5870    658     sctC    T3SS    2.7e-52 176.7
      0.963235  0.844985        49      604
PSAE001c01_051090       PSAE001c01      7801    714     sctC    T3SS    1.9e-46 157.4
      0.571691  0.463585        374     704
```

Note: Each tabular output file contains a header line describing each column in the output.

4.2 Systems detection results

Different types of tabular outputs are provided. Headers are provided with the content of the lines in the file.

- macsyfinder.tab - for **"ordered" datasets only** (db_type is "ordered_replicon" or "gembase"). It provides a summary of the number of each type of systems that have been detected.

- macsyfinder.report - contains all the sequence identifiers of proteins detected as being part of a system, along with statistics on the Hmmer hit, and the status of the component in the system.
- macsyfinder.summary - contains a line of information for each detected system.

4.2.1 macsyfinder.tab

For each replicon, a line gives the occurrences of systems that were asked for detection. For example, if the detection was run for the Flagellum and the T6SS, the output will look like:

```
#Replicon Flagellum_single_locus Flagellum_multi_loci T6SS_single_locus T6SS_multi_loci
escherichia06 1   1   1   0
```

which means that this "escherichia06" genome harbors 1 flagellum in a single locus, 1 flagellum scattered on multiple loci, and 1 T6SS in a single locus.

4.2.2 macsyfinder.report

Each line corresponds to a "hit" that has been assigned to a detected system. It includes:

- Hit_Id - the sequence identifier of the hit
- Replicon_name - the name of the replicon it belongs to
- Position - the position of the sequence in the replicon
- Sequence_length - the length of the sequence
- Gene - the name of the components matched with the profile
- Reference_system - the system that includes the component matched
- Predicted_system - the system assigned
- System_Id - the unique identifier attributed to the detected system
- System_status - the status of the detected system
- Gene_status - the status of the component in the assigned system's definition
- i-evalue - Hmmer statistics, the indepent-evalue
- Score - Hmmer score
- Profile_coverage - the percentage of the profile covered by the alignment with the sequence
- Sequence_coverage - the percentage of the sequence covered by the alignment with the profile
- Begin_match - the position in the sequence where the profile match begins
- End_match - the position in the sequence where the profile match ends

4.2.3 macsyfinder.summary

Each line corresponds to a system that has been detected. It includes:

- Replicon_name - the name of the replicon
- System_Id - the unique identifier attributed to the detected system
- Reference_system - the type of system detected
- System_status - the status of the system

- Nb_loci - the number of loci that constitutes the system
- Nb_Ref_mandatory - the number of mandatory genes in the system definition
- Nb_Ref_accessory - the number of accessory genes in the system definition
- Nb_Ref_Genes_detected_NR - the number of different components (accessory+mandatory) in the system
- Nb_Genes_with_match - the number of components detected with the profiles in the system
- System_length - the full number of components (with match or not) in the locus (or loci) that constitutes the system
- Nb_Mandatory_NR - the number of different mandatory components matched
- Nb_Accessory_NR - the number of different accessory components matched
- Nb_missing_mandatory - the number of mandatory components from the system definition with no match in this system occurrence
- Nb_missing_accessory - the number of accessory components from the system definition with no match in this system occurrence
- List_missing_mandatory - the list of the missing mandatory components
- List_missing_accessory - the list of the missing accessory components
- Loci_positions - the sequence position (rank of the fasta sequence in the input sequence file) of the different loci encoding the system
- Occur_Mandatory - counts of the mandatory components
- Occur_Accessory - counts of the accessory components
- Occur_Forbidden - counts of the forbidden components

4.3 Logs and configuration files

Three specific output files are built to store information on the MacSyFinder execution:

- macsyfinder.out - contains information on the procedure during systems detection: clusters found, decisions made for system inference... The same information is also displayed on the standard output.
- macsyfinder.conf - contains the configuration information of the run. It is useful to recover the parameters used for the run.
- macsyfinder.log - the log file, contains raw information on the run. Please send it to us with any bug report.

4.4 File for MacSyview: results.macsyfinder.json

This file in JSON format is used by MacSyView, for graphical output purpose. It must be loaded through MacSyView to graphically visualize detected systems. For more details, see *MacSyView's description*.

CHAPTER

FIVE

MACSYVIEW: VISUALIZING MACSYFINDER'S RESULTS!

MacSyView is a standalone web-browser application to visualize MacSyFinder's detected systems. MacSyView relies on JSON files outputted by MacSyFinder to display the list of detected systems, and a detailed view of each system. It allows visualizing the content of systems, their genomic context, and generates SVG files that can be exported for drawing purpose.

5.1 MacSyView How To

1. Run MacSyFinder to detect your favorite system!
2. Launch MacSyView:

- Either, **run the wrapper 'macsyview'** installed with MacSyFinder's binaries (*i.e.*, *macsyfinder* - for Linux).
- Or **open with your web-browser the html page**: /usr/share/macsyview/index.html or /share/macsyview/index.html (or in the path specified during installation for data associated with MacSyFinder).

3. Select the .json output file in the output directory of the run
4. Choose the system you want to visualize in the list...
5. ...and here it is!

Note: The MacSyView application runs everything on the user's computer, even if it uses the technologies of Web browsers. No data are sent out of the user's device.

5.2 Graphical output description

The content of the system view depends on the type of the input dataset.

- upper panel: an overview of the effectives of detected components is displayed **for all types** of datasets, per type of components in the system definition. It is a direct representation of how the definition was fulfilled during detection.
- middle panel: only for **ordered datasets**, the detected system is shown in its genomic context, including nearby proteins that were not annotated as system's components.

- lower panel: for **all datasets**, a table containing information on detected components (and eventually nearby proteins for ordered datasets) is displayed. It includes sequence information, and in the case of system's components, Hmmer hit information, and function assigned in the system.

5.3 Technology used

MacSyView was coded in Javascript and uses third-party libraries that are all accredited in the COPYRIGHT file distributed with the MacSyFinder/MacSyView package.

It includes among others:

- the Raphael library for systems drawing,
- the Bootstrap library for HTML design and
- the Mustache library for HTML templating in Javascript.

The JQuery, JQuery-mousewheel and Raphael.Export libraries were also used.

It was tested on Chromium and Firefox for Linux, and on Chrome, Firefox and Safari for Mac OS X.

5.4 Screenshot

Here is a view of one of the three systems detected with the example dataset *presented here*:

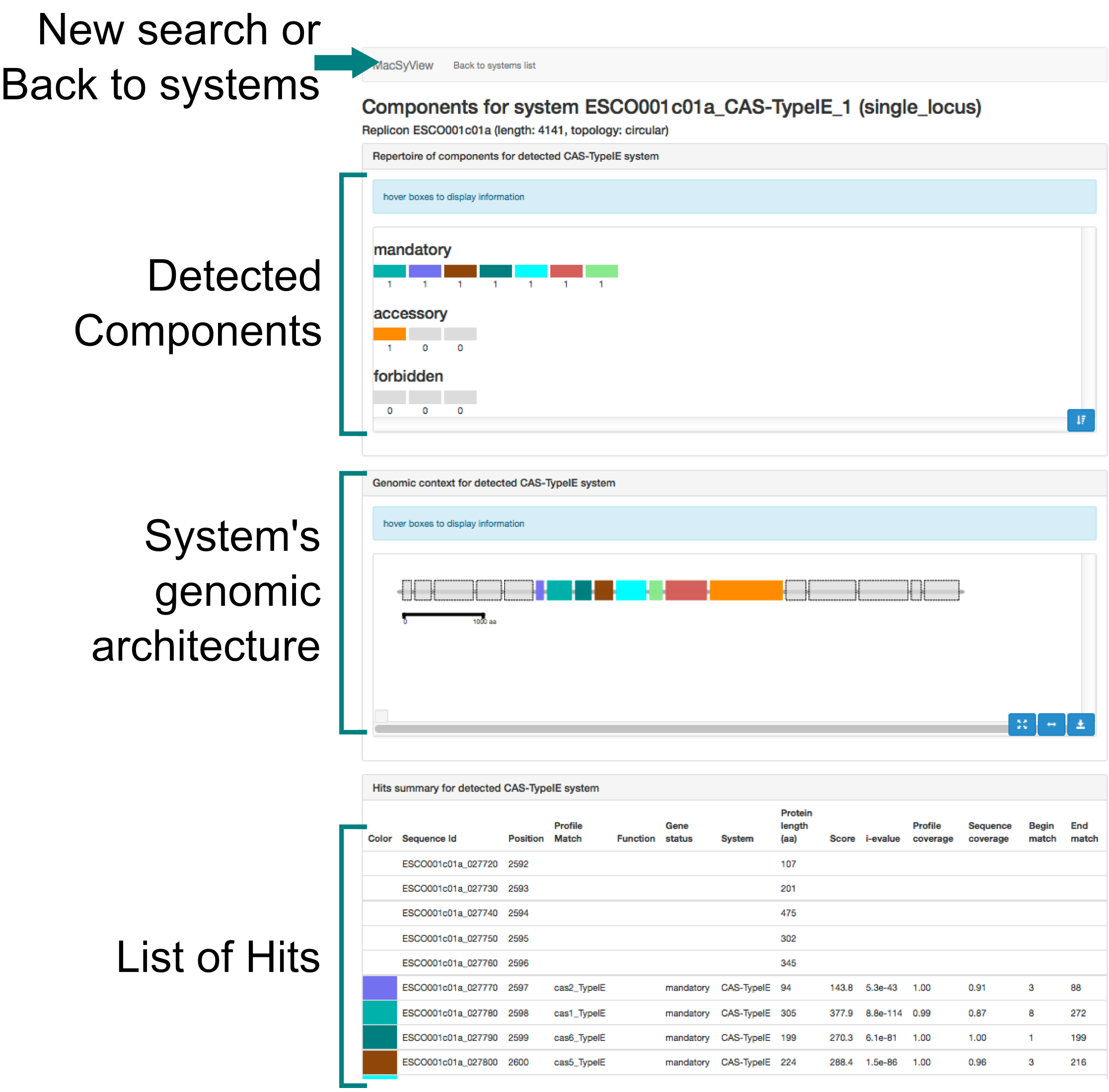
New search or
Back to systems
Detected
Components
System's
genomic
architecture
List of Hits
MacSyView
Back to systems list
Components for system ESCO001c01a_CAS-TypeIE_1 (single_locus)
Replicon ESCO001c01a (length: 4141, topology: circular)
Repertoire of components for detected CAS-TypeIE system
hover boxes to display information
mandatory
accessory
forbidden
Genomic context for detected CAS-TypeIE system
hover boxes to display information
Hits summary for detected CAS-TypeIE system
Color Sequence Id Position Profile Match Function Gene status System Protein length (aa) Score i-evalue Profile coverage Sequence coverage Begin match End match
ESCO001c01a_027720 2592 107
ESCO001c01a_027730 2593 201
ESCO001c01a_027740 2594 475
ESCO001c01a_027750 2595 302
ESCO001c01a_027760 2596 345
ESCO001c01a_027770 2597 cas2_TypeIE mandatory CAS-TypeIE 94 143.8 5.3e-43 1.00 0.91 3 88
ESCO001c01a_027780 2598 cas1_TypeIE mandatory CAS-TypeIE 305 377.9 8.8e-114 0.99 0.87 8 272
ESCO001c01a_027790 2599 cas6_TypeIE mandatory CAS-TypeIE 199 270.3 6.1e-81 1.00 1.00 1 199
ESCO001c01a_027800 2600 cas5_TypeIE mandatory CAS-TypeIE 224 288.4 1.5e-86 1.00 0.96 3 216

Part II

MacSyFinder functioning

CHAPTER

SIX

MACROMOLECULAR SYSTEMS DEFINITION

6.1 Principles

MacSyFinder relies on the definition of models of macromolecular systems with an **XML grammar** that is described *below*.

A system is defined in a dedicated file named after the system (*e.g.*, 'T1SS.xml' for T1SS, the Type 1 Secretion System) by a set of **components** (*i.e.* proteins, or protein-coding genes given the context) with different attributes and that are used for **content description**. Some components are specific to the system, and some are possibly from other systems. In the latter case, the full description of the gene with its attributes must be defined in the XML file of the original system. Features regarding **co-localization** parameters for system detection are also defined in this system-specific file.

Three distinct types of components can be used to model a given system content, and which corresponds to Gene objects, and the corresponding HMM protein profiles.

- **Mandatory** components represent essential components to be found to infer the System presence.
- **Accessory** components correspond to components that can be found in some systems occurrence, or fastly evolving components that are hard to detect with a single profile.
- **Forbidden** components are components which presence is eliminatory for the System assessment.

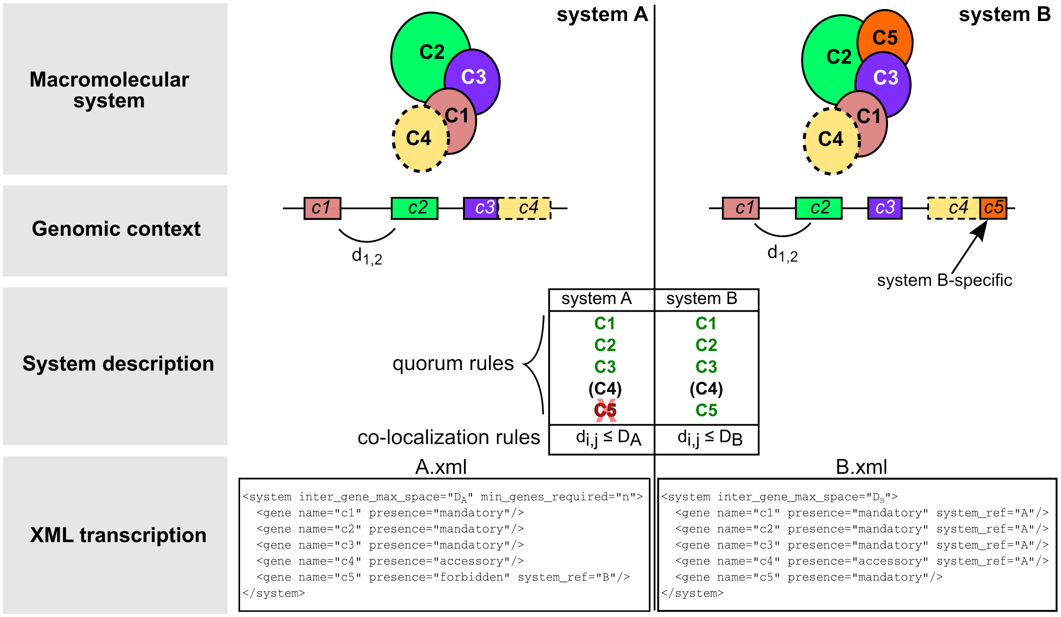

6.2 The XML hierarchy

- The element root is "system".
  - It has a mandatory attribute: "inter_gene_max_space", an integer representing the maximal number of components without a match between two components with a match for a component profile.
  - The element "system" may have attributes:
    - "min_mandatory_genes_required": an integer representing the minimal number of mandatory genes required to infer the system presence.
    - "min_genes_required": an integer representing the minimal number of mandatory or accessory genes (whose corresponding proteins match a profile of the system) required to infer the system presence.
    - "max_nb_genes": an integer representing the maximal number of mandatory or accessory genes in the system.
    - "multi_loci": a boolean set to True ("1", "true" or "True") to allow the definition of "scattered" systems (systems encoded by different loci). If not specified, *default value is false*.
  - The system contains one or more element "gene".
- The element "gene" has several mandatory attributes:
  - "name": which must match to a profile in the profile directory.
  - "presence": which can take three values "mandatory", "accessory", "forbidden".

  The element "gene" may have other attributes:

  - "system_ref": which is a reference to the macromolecular system from where the gene comes from (this attribute is used for forbidden gene and homologs gene). If system_ref is not specified, it means the gene is from the current system.

    - "loner": which is a boolean. If a gene is loner that means this gene can be isolated on the genome (*default false*).
    - "exchangeable": which is a boolean. If a gene is exchangeable (value set to "1", "true" or "True") that means this gene or one of its homologs or analogs can be interchanged for the assessment of the presence of the macromolecular system (*default false*).
    - "multi_system": which is a boolean. If a gene is "multi_system" (value set to "1", "true" or "True"), it means that it can be used to fill by multiple systems occurrences. (*default false*).
    - "aligned": which is a boolean (this attribute is used only for homologs).
    - "inter_gene_max_space": an integer that defines gene-wise value of system's "inter_gene_max_space" parameter (see above).
    - an element "homologs" that contains a list of homologous genes that can potentially match the same sequences. They can potentially be functionally equivalent to the reference gene if it was declared "exchangeable"
    - an element "analogs" that contains a list of analogous genes that can potentially be functionally equivalent, if the parent gene was declared "exchangeable".
- The elements "homologs" and "analogs" can contain one or more element "gene".

Example of a system definition in XML:

```
<system inter_gene_max_space="5">
  <gene name="gspD" presence="mandatory">
     <homologs>
         <gene name="sctC" system_ref="T3SS"/>
     </homologs>
  </gene>
  <gene name="sctN_FLG" presence="mandatory" loner="1" exchangeable="1"/>
     <analogs>
         <gene name="gspE" system_ref="T2SS"/>
         <gene name="pilT" system_ref="T4P"/>
     </analogs>
  <gene name="sctV_FLG" presence="mandatory"/>
  <gene name="flp" presence="accessory"/>
</system>
```

Warning:

- a gene is identified by its name.
- a gene can be defined only **once** in all systems.
- other occurrences of this gene must be specified as references (using the "system_ref" attribute to specify what is the native system).
- if a gene is specified with the attribute "system_ref", it means it has been (or has to be) defined in the system specified by "system_ref".
- if a gene is not specified with the attribute "system_ref", it means it belongs to the current system, where it has to be defined with all its features.

CHAPTER

SEVEN

MACSYFINDER IMPLEMENTATION OVERVIEW

MacSyFinder is implemented with an object-oriented architecture. The objects are described in the current *API documentation*. An overview of the main classes used to model the systems to be detected is provided below.

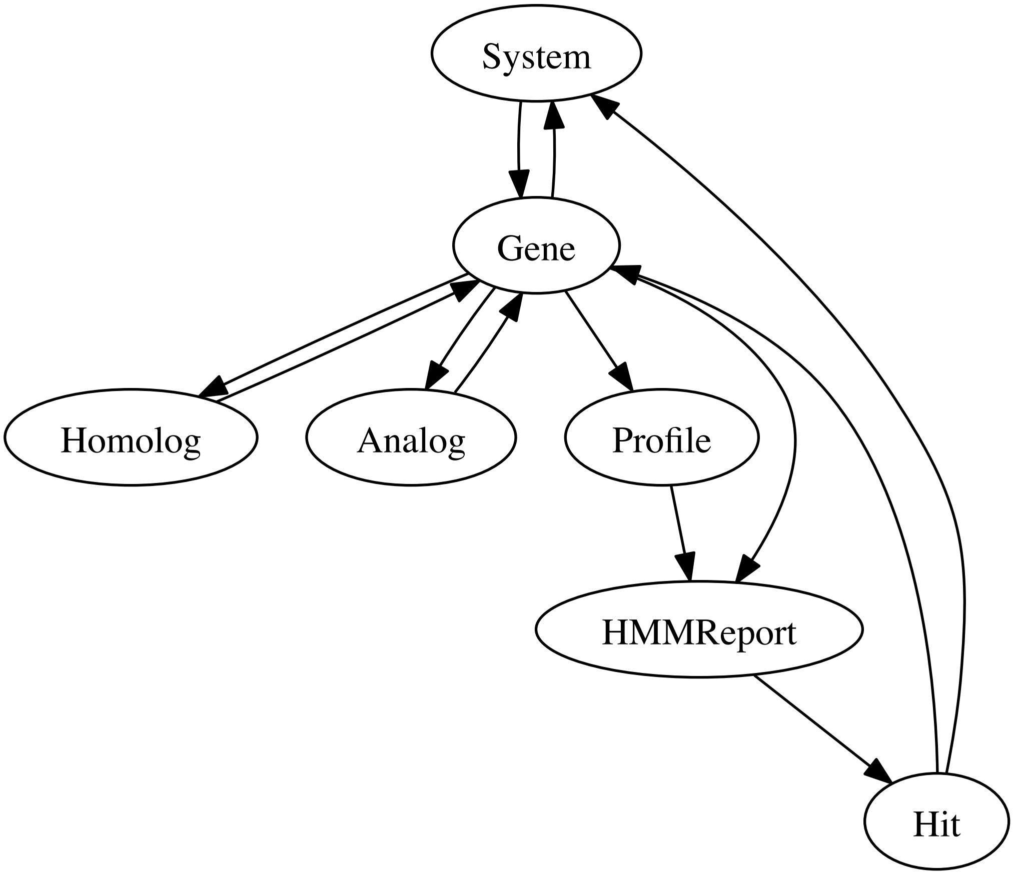

The *"System"* class models the systems to detect and contains a list of instances of the *"Gene"* class, which models each component of a given System. The *"Homolog"* and *"Analog"* classes encapsulate a "Gene" and model respectively relationships of homology and analogy between components.

A *"Gene"* represents a component from the System and refers to an instance of the *"Profile"* object that corresponds to an hidden Markov model protein profile (used for sequence similarity search with the Hmmer program).

The *"Config"* class (see the *Configuration API*) handles the program parameters, including Hmmer search parameters, and the set of sequences to query (represented by the "Database" object).

The *"Database"* stores information on the dataset, including necessary information to detect systems in both linear and circular chromosomes (see the *Database API*).

A set of parsers and object factories are used to fill the objects from command-line and input files (*i.e.* the optional configuration file and the XML files describing the systems), and to ensure their uniqueness and integrity.

Once these objects are initialized and the detection is launched, Hmmer is executed on the sequences of the database (optionally in parallel) with a unique list of profiles corresponding to the systems to detect. Subsequently, Hmmer output files are parsed, and selected hits (given the search parameters provided) are used to fill *"Hits"* objects, which contain information for the detection of the systems.

During the treatment of the *"Hits"* for *"Systems"* detection, the occurrences of the systems (*"SystemOccurence"* objects) are filled, and the **decision rules** associated with the systems (quorum and co-localization in the case of an "ordered" dataset) are applied. See the following sections for more details on above objects.

7.1 The System object

The *System object* represents a macromolecular system to detect. It is defined *via* a definition file in XML stored in a dedicated location that can be specified *via* the configuration file, or the command-line (*-d* parameter). See *The XML hierarchy* for more details on the XML grammar.

An object *SystemParser* is used to build a system object from its XML definition file.

A system is named after the file name of its XML definition. A system has an attribute *inter_gene_max_space* which is an integer, and three kind of components are listed in function of their presence in the system:

- The genes that must be present in the genome to define this system ("mandatory").
- The genes that can be present, but do not have to be found in every case ("accessory").
- The genes that must not be present in the system ("forbidden").

Note: a complete description of macromolecular systems modelling is available in the section *Macromolecular systems definition*

7.2 The Gene object

The *Gene object* represents genes encoding the protein components of a System. Each Gene points out its System of origin (`macsypy.system.System`). A Gene must have a correponding HMM protein profile. These profiles are represented by Profile objects (`macsypy.gene.Profile`), and must be named after the gene name. For instance, the gene *gspD* will correspond to the "gspD.hmm" profile file. See *The Profile object*). A Gene has several properties described in the *Gene API*.

A Gene may have Homologs or Analogs. An *"Homolog"* (resp. *"Analog"*) object encapsulates a Gene and has a reference to the Gene it is homolog (resp. *"analog"*) to. See the *Homolog API* and *Analog API* for more details.

Warning: To optimize computation and to avoid concurrency problems when we search several systems, each gene must be instanciated only once, and stored in a *"gene_bank"*. gene_bank is a `macsypy.gene.GeneBank` object. The gene_bank and system_bank are filled by the system_parser (`macsypy.system_parser.SystemParser`)

7.3 The Profile object

Each *"Gene"* component corresponds to a *"Profile"*. The *"Profile"* object is used for the search of the gene with Hmmer. Thus, a *"Profile"* must match a HMM file, which name is based on the profile name. For instance, the *gspG* gene has the corresponding "gspG.hmm" profile file provided at a dedicated location.

7.4 Reporting Hmmer search results

A *"HMMReport"* (`macsypy.report.HMMReport`) object represents the results of a Hmmer program search on the input dataset with a hidden Markov model protein profile. This object has methods to extract and build *"Hits"* that are then analyzed for systems assessment.

It analyses Hmmer raw outputs, and applies filters on the matches (according to *Hmmer options*). See *Hmmer results outputs* for details on the resulting output files. For profile matches selected with the filtering parameters, *"Hit"* objects are built (see *the Hit API*).

MACSYFINDER FUNCTIONING

8.1 Functioning overview

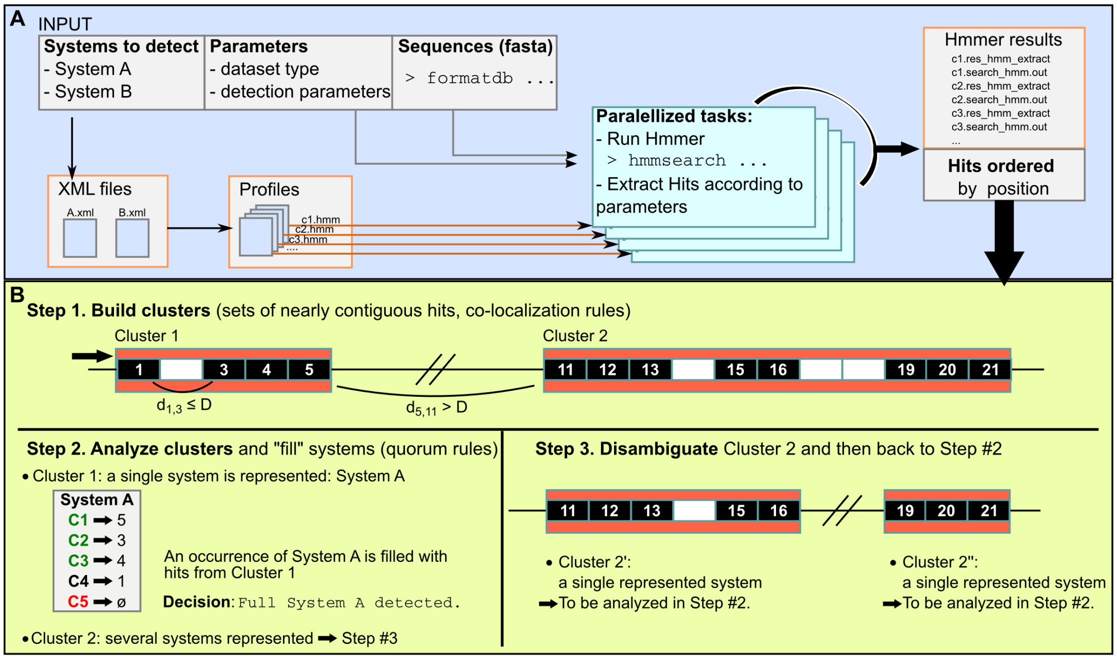

8.2 A. Searching for systems' components

MacSyFinder is run from the command-line using a variety of input files and options. See *Input dataset* for more details.

Initially, MacSyFinder **searches for the components** of a system by sequence similarity search.

From the list of systems to detect, a non-redundant list of components to search is built. For each system, the list includes:

- mandatory components
- accessory components
- forbidden components

- homologs and/or analogs of these three types of components in the case they are *"exchangeable"*

Hmmer is run on the corresponding set of HMM profiles, and the hits are filtered according to criteria defined by the user (see *Hmmer options* and *HMMReport API*). This step, and the extraction of significant hits can be performed in parallel (*-w* command-line option). See the *Command-line options*, and the *search_genes API* for more details.

8.3 B. Assessing systems presence

The following steps depend on whether the input dataset is *ordered* (complete or nearly complete genome(s)), or *unordered* (metagenomes, or unassembled genome) (see *Input dataset*). In the case of **ordered datasets**, the hits of the previous analysis are used to build *clusters of co-localized genes* as defined in the XML files. These clusters are then scanned to check for the model specifications like minimal quorum of "Mandatory" or "Accessory" genes or the absence of "Forbidden" components. When the gene order is unknown the power of the analysis is more limited. In this case, and depending on the type of dataset, the presence of systems can be suggested only on the basis of the quorum of genes. The results are outputted in a tabular and graphical form (see *Output format*).

8.3.1 For *ordered* datasets:

1. The search starts first with the formation of clusters of contiguous hits **(co-localization criterion)** for each replicon. Two hits are said contiguous if their genomic location is separated by less than D protein-encoding gene, D being the maximum of the parameter "inter_gene_max_space" from the two genes with hits (system-specific, of gene-specific parameter).
2. Clusters are then scanned, and those containing only genes from a single system are kept for further analyses (step 4.), wether those with multiple systems represented are analysed with a disambiguation step (step 3.).
3. The disambiguation step consists in splitting clusters that contains genes from different systems into sub-clusters that contain genes from a single system. Valid sub-clusters are then analysed like other clusters (step 2.). In the complex cases where genes from a same system are scattered into the cluster, then corresponding sub-clusters will not be further analysed for system detection.
4. Valid clusters are used to fill system occurrences (`macsypy.search_systems.SystemOccurence`). In this step, the **quorum** criteria for the system assessment are checked according to the system's definition. In the case a single locus fills a complete system occurrence, it is stored and reported in the output files (**"single-locus"** occurrence). Otherwise, if the cluster corresponds to a valid but incomplete system, it is stored for inclusion in a scattered system occurrence (**"multi-loci"** occurrence).
5. When all clusters, "loner" genes and "multi_system" genes were scanned for inclusion in system occurrences, a decision is made for every system occurrence regarding the **quorum rules** defined for the corresponding system.

Note: When the "multi_loci" option is turned on, a single "multi-loci" system is assessed per replicon, even if it could correspond to multiple scattered systems. Thus, the "single-locus" systems correspond to a more powerful mode of detection.

Warning: Cases where systems are consecutive will be treated, and separate systems will be detected, but complex cases of detection, *i.e.* when systems' components are intermingled will not be considered.

8.3.2 For *unordered* datasets:

1. The Hits are grouped by system.

2. They are used to fill a single system occurrence (`macsypy.search_systems.SystemOccurence`) per system type.

Note: The "unordered" mode of detection is less powerful, as a single occurrence of a given system is filled for an entire dataset with hits that origin is unknown. Please consider "systems assessments" with caution in this mode.

Part III

MacSyFinder API documentation

CHAPTER

NINE

CONFIGURATION API

Options to run MacSyFinder can be specified in a *Configuration file*. The API described below handles all configuration options for MacSyFinder. The Config object provides some default values, and performs some validations of the values.

9.1 Config API reference

class macsypy.config.**Config**(*cfg_file='', sequence_db=None, db_type=None, replicon_topology=None, topology_file=None, inter_gene_max_space=None, min_mandatory_genes_required=None, min_genes_required=None, max_nb_genes=None, multi_loci=None, hmmer_exe=None, index_db_exe=None, e_value_res=None, i_evalue_sel=None, coverage_profile=None, def_dir=None, res_search_dir=None, res_search_suffix=None, profile_dir=None, profile_suffix=None, res_extract_suffix=None, log_level=None, log_file=None, worker_nb=None, config_file=None, previous_run=None, build_indexes=None*)

Parse configuration files and handle the configuration according to the following file location precedence: /etc/macsyfinder/macsyfinder.conf < ~/.macsyfinder/macsyfinder.conf < .macsyfinder.conf

If a configuration file is given on the command-line, this file will be used. *In fine* the arguments passed on the command-line have the highest priority.

__init__(*cfg_file='', sequence_db=None, db_type=None, replicon_topology=None, topology_file=None, inter_gene_max_space=None, min_mandatory_genes_required=None, min_genes_required=None, max_nb_genes=None, multi_loci=None, hmmer_exe=None, index_db_exe=None, e_value_res=None, i_evalue_sel=None, coverage_profile=None, def_dir=None, res_search_dir=None, res_search_suffix=None, profile_dir=None, profile_suffix=None, res_extract_suffix=None, log_level=None, log_file=None, worker_nb=None, config_file=None, previous_run=None, build_indexes=None*)

Parameters

- **cfg_file** (*string*) – the path to the MacSyFinder configuration file to use
- **previous_run** (*string*) – the path to the results directory of a previous run
- **sequence_db** (*string*) – the path to the sequence input dataset (fasta format)
- **db_type** (*string*) – the type of dataset to deal with. "unordered_replicon" corresponds to a non-assembled genome, "unordered" to a metagenomic dataset, "ordered_replicon" to an assembled genome, and "gembase" to a set of replicons where sequence identifiers follow this convention ">RepliconName_SequenceID"."

- **replicon_topology** (*string*) – the topology ('linear' or 'circular') of the replicons. This option is meaningful only if the db_type is 'ordered_replicon' or 'gembase'
- **topology_file** (*string*) – a tabular file of mapping between replicon names and the corresponding topology (e.g. "RepliconA linear")
- **inter_gene_max_space** (*list of list of 2 elements [[string system, integer space] , ...]*) –
- **min_mandatory_genes_required** (*list of list of 2 elements [[string system, integer] , ...]*) –
- **min_genes_required** (*list of list of 2 elements [[string system, integer] , ...]*) –
- **max_nb_genes** (*list of list of 2 elements [[string system, integer] , ...]*) –
- **multi_loci** (*string*) –
- **hmmer_exe** (*string*) – the Hmmer "hmmsearch" executable
- **index_db_exe** (*string*) – the indexer executable ("makeblastdb" or "formatdb")
- **e_value_res** (*float*) – maximal e-value for hits to be reported during Hmmer search
- **i_evalue_sel** (*float*) – maximal independent e-value for Hmmer hits to be selected for system detection
- **coverage_profile** (*float*) – minimal profile coverage required in the hit alignment to allow the hit selection for system detection
- **def_dir** (*string*) – the path to the directory containing systems definition files (.xml)
- **res_search_dir** (*string*) – the path to the directory where to store MacSyFinder search results directories.
- **res_search_suffix** (*string*) – the suffix to give to Hmmer raw output files
- **res_extract_suffix** (*string*) – the suffix to give to filtered hits output files
- **profile_dir** (*string*) – path to the profiles directory
- **profile_suffix** (*string*) – the suffix of profile files. For each 'Gene' element, the corresponding profile is searched in the 'profile_dir', in a file which name is based on the Gene name + the profile suffix.
- **log_level** (*int*) – the level of log output
- **log_file** (*string*) – the path to the directory to write MacSyFinder log files
- **worker_nb** (*int*) – maximal number of processes to be used in parallel (multi-thread run, 0 use all cores availables)
- **build_indexes** (*boolean*) – build the indexes from the sequence dataset in fasta format

`__weakref__`
list of weak references to the object (if defined)

`_validate`(*cmde_line_opt*, *cmde_line_values*)
Get all configuration values and check the validity of their values. Create the working directory

Parameters

- **cmde_line_opt** (*dict, all values are cast in string*) – the options from the command line
- **cmde_line_values** (*dict, values are not cast*) – the options from the command line

Returns all the options for this execution

Return type dictionary

build_indexes

Returns True if the indexes must be rebuilt, False otherwise

Return type boolean

coverage_profile

Returns the coverage threshold used to select a hit for systems detection and for the Hmmer report (filtered hits)

Return type float

db_type

Returns the type of the input sequence dataset. The allowed values are :'unordered_replicon', 'ordered_replicon', 'gembase', 'unordered'

Return type string

def_dir

Returns the path to the directory where are stored definitions of secretion systems (.xml files)

Return type string

e_value_res

Returns The e_value threshold used by Hmmer to report hits in the Hmmer raw output file

Return type float

hmmer_dir

Returns the name of the directory where the hmmer results are stored

Return type string

hmmer_exe

Returns the name of the binary to execute for homology search from HMM protein profiles (Hmmer)

Return type string

i_evalue_sel

Returns the i_evalue threshold used to select a hit for systems detection and for the Hmmer report (filtered hits)

Return type float

index_db_exe

Returns the name of the binary to index the input sequences dataset for Hmmer

Return type string

inter_gene_max_space(*system*)

Parameters **system** (*string*) – the name of a system

Returns the maximum number of components with no match allowed between two genes with a match to consider them contiguous (at the system level)

Return type integer

max_nb_genes(*system*)

Parameters **system** (*string*) – the name of a system

Returns the maximum number of genes to assess the system presence

Return type integer

`min_genes_required`(*system*)

Parameters **system** (*string*) – the name of a system

Returns the genes (mandatory+accessory) quorum to assess the system presence

Return type integer

`min_mandatory_genes_required`(*system*)

Parameters **system** (*string*) – the name of a system

Returns the mandatory genes quorum to assess the system presence

Return type integer

`multi_loci`(*system*)

Parameters **system** (*string*) – the name of a system

Returns the genes (mandatory+accessory) quorum to assess the system presence

Return type boolean

`previous_run`

Returns the path to the previous run directory to use (to recover Hmmer raw output)

Return type string

`profile_dir`

Returns the path to the directory where are the HMM protein profiles which corresponds to Gene

Return type string

`profile_suffix`

Returns the suffix for profile files

Return type string

`replicon_topology`

Returns the topology of the replicons. Two values are supported 'linear' (default) and circular. Only relevant for 'ordered' datasets

Return type string

`res_extract_suffix`

Returns the suffix of extract files (tabulated files after HMM output parsing and filtering of hits)

Return type string

`res_search_dir`
:return the path to the directory to store results of MacSyFinder runs :rtype: string

`res_search_suffix`

Returns the suffix for Hmmer raw output files

Return type string

`save`(*dir_path*)
save the configuration used for this run in the ini format file

`sequence_db`

Returns the path to the input sequence dataset (in fasta format)

Return type string

`topology_file`

Returns the path to the file of replicons topology.

Return type string

`worker_nb`

Returns the maximum number of parallel jobs

Return type int

`working_dir`

Returns the path to the working directory to use for this run

Rtpe string

CHAPTER

TEN

DATABASE API

The “database” object handles the indexes of the sequence dataset in fasta format, and other useful information on the input dataset.

MacSyFinder needs several indexes to run, and speed up the analyses.

- index for hmmsearch (Hmmer program)
- index for MacSyFinder

hmmsearch needs to index the sequences to speed up the analyses. The indexes are built by the external tools *formatdb* (ftp://ftp.ncbi.nlm.nih.gov/blast/executables/release/LATEST/ncbi.tar.gz) or *makeblastdb*. MacSyFinder tries to find formatdb indexes in the same directory as the sequence file. If the indexes are present MacSyFinder uses these index, otherwise it builds these indexes using formatdb or makeblastdb.

MacSyFinder needs also to have the length of each sequence and its position in the database to compute some statistics on Hmmer hits. Thus it also builds an index (with .idx suffix) that is stored in the same directory as the sequence dataset. If this file is found in the same folder than the input dataset, MacSyFinder will use it. Otherwise, it will build it.

The user can force MacSyFinder to rebuild these indexes with the “–idx” option on the command-line.

Additionally, for ordered datasets (db_type = ‘gembase’ or ‘ordered_replicon’), MacSyFinder builds an internal “database” from these indexes to store information about replicons, their begin and end positions, and their topology. The begin and end positions of each replicon are computed from the sequence file, and the topology from the parsing of the topology file (–topology-file, see *Topology files*).

10.1 database API reference

class `macsypy.database.`**`Indexes`**(*cfg*)

Handle the indexes for macsyfinder:

- find the indexes for hmmer, or build them using formatdb or makeblastdb external tools
- find the indexes required by macsyfinder to compute some scores, or build them.

`__init__`(*cfg*)

The constructor retrieves the file of indexes in the case they are not present or the user asked for build indexes (–idx) Launch the indexes building.

Parameters **cfg** (`macsypy.config.Config` object) – the configuration

`__weakref__`

list of weak references to the object (if defined)

_build_hmmer_indexes()
build the index files for hmmer using the formatdb or makeblastdb tool

_build_my_indexes()
Build macsyfinder indexes. These indexes are stored in a file.

The file format is the following:

- one entry per line, with each line having this format:
- sequence id;sequence length;sequence rank

build(*force=False*)
Build the indexes from the sequence dataset in fasta format

Parameters **force** (*boolean*) – If True, force the index building even if the index files are present in the sequence dataset folder

find_hmmer_indexes()

Returns The hmmer index files. If indexes are inconsistent (some file(s) missing), a Runtime Error is raised

Return type list of string

find_my_indexes()

Returns the file of macsyfinder indexes if it exists in the dataset folder, None otherwise.

Return type string

class macsypy.database.**RepliconDB**(*cfg*)
Stores information (topology, min, max, [genes]) for all replicons in the sequence_db the Replicon object must be instantiated only for sequence_db of type 'gembase' or 'ordered_replicon'

__contains__(*replicon_name*)

Parameters **replicon_name** (*string*) – the name of the replicon

Returns True if replicon_name is in the repliconDB, false otherwise.

Return type boolean

__getitem__(*replicon_name*)

Parameters **replicon_name** (*string*) – the name of the replicon to get information on

Returns the RepliconInfo for the provided replicon_name

Return type RepliconInfo object

Raise KeyError if replicon_name is not in repliconDB

__init__(*cfg*)

Parameters **cfg** (macsypy.config.Config object) – The configuration object

---

Note: This class can be instanciated only if the db_type is 'gembase' or 'ordered_replicon'

__weakref__
list of weak references to the object (if defined)

_fill_gembase_min_max(*topology*, *default_topology*)
For each replicon_name of a gembase dataset, it fills the internal dictionary with a namedtuple Replicon-Info

Parameters

- **topology** (*dict*) – the topologies for each replicon (parsed from the file specified with the option –topology-file)
- **default_topology** (*string*) – the topology provided by the config.replicon_topology

_fill_ordered_min_max(*default_topology=None*)
For the replicon_name of the ordered_replicon sequence base, fill the internal dict with RepliconInfo

Parameters **default_topology** (*string*) – the topology provided by config.replicon_topology

_fill_topology()
Fill the internal dictionary with min and max positions for each replicon_name of the sequence_db

get(*replicon_name*, *default=None*)

Parameters

- **replicon_name** (*string*) – the name of the replicon to get informations
- **default** (*any*) – the value to return if the replicon_name is not in the RepliconDB

Returns the RepliconInfo for replicon_name if replicon_name is in the repliconDB, else default. If default is not given, it is set to None, so that this method never raises a KeyError.

Return type `RepliconInfo` object

items()

Returns a copy of the RepliconDB as a list of (replicon_name, RepliconInfo) pairs

iteritems()

Returns an iterator over the RepliconDB as a list (replicon_name, RepliconInfo) pairs

replicon_infos()

Returns a copy of the RepliconDB as list of replicons info

Return type RepliconInfo instance

replicon_names()

Returns a copy of the RepliconDB as a list of replicon_names

class macsypy.database.**RepliconInfo**
RepliconInfo(topology, min, max, genes)

__getnewargs__()
Return self as a plain tuple. Used by copy and pickle.

static __new__(*_cls*, *topology*, *min*, *max*, *genes*)
Create new instance of RepliconInfo(topology, min, max, genes)

__repr__()
Return a nicely formatted representation string

_asdict()
Return a new OrderedDict which maps field names to their values

classmethod _make(*iterable*, *new=<built-in method __new__ of type object at 0x10cacd840>*, *len=<built-in function len>*)
Make a new RepliconInfo object from a sequence or iterable

_replace(*_self*, ***kwds*)
Return a new RepliconInfo object replacing specified fields with new values

genes
Alias for field number 3

max
Alias for field number 2

min
Alias for field number 1

topology
Alias for field number 0

macsypy.database.**fasta_iter**(*fasta_file*)

Parameters **fasta_file** (*file object*) – the file containing all input sequences in fasta format.

Author http://biostar.stackexchange.com/users/36/brentp

Returns for a given fasta file, it returns an iterator which yields tuples (string id, string comment, int sequence length)

Return type iterator

CHAPTER

ELEVEN

SYSTEM API

It represents a macromolecular system to detect. See *The System object* for an overview of its implementation.

Note: a complete description of macromolecular systems modelling is available in the section *Macromolecular systems definition*

11.1 SystemBank API reference

class `macsypy.system.`**`SystemBank`**

Build and store all Systems objects. Systems must not be instanciated directly. This system factory must be used. It ensures there is a unique instance of a system for a given system name. To get a system, use the method __getitem__ via the "[]". If the System is already cached in the SystemBank, it is returned. Otherwise a new system is built, stored and then returned.

`__contains__`(*system*)

Implement the membership test operator

Parameters **system** (`macsypy.system.System` object) – the system to test

Returns True if the system is in the System factory, False otherwise

Return type boolean

`__getitem__`(*name*)

Parameters **name** (*string*) – the name of the system

Returns the system corresponding to the name. If the system already exists, return it, otherwise build it and return it.

Return type `macsypy.system.System` object

`__iter__`()

Return an iterator object on the systems contained in the bank

`__len__`()

Returns the number of systems stored in the bank

Return type integer

`__weakref__`

list of weak references to the object (if defined)

`add_system`(*system*)

Parameters **system** (`macsypy.system.System` object) – the system to add

Raise KeyError if a system with the same name is already registered.

Note: Don't instanciate your own SystemBank use the system_bank at the top level of the module.

```
from macsypy.system import system_bank
```

11.2 System API reference

class macsypy.system.**System**(*cfg*, *name*, *inter_gene_max_space*, *min_mandatory_genes_required=None*, *min_genes_required=None*, *max_nb_genes=None*, *multi_loci=False*)

Handle a secretion system.

`__init__`(*cfg*, *name*, *inter_gene_max_space*, *min_mandatory_genes_required=None*, *min_genes_required=None*, *max_nb_genes=None*, *multi_loci=False*)

Parameters

- **cfg** (`macsypy.config.Config` object) – the configuration object
- **name** (*string*) – the name of the system
- **inter_gene_max_space** (*integer*) – the maximum distance between two genes (**co-localization** parameter)
- **min_mandatory_genes_required** (*integer*) – the quorum of mandatory genes to define this system
- **min_genes_required** (*integer*) – the quorum of genes to define this system
- **max_nb_genes** (*integer*) –
- **multi_loci** (*boolean*) –

`__weakref__`

list of weak references to the object (if defined)

`accessory_genes`

Returns the list of genes that are allowed in this secretion system

Return type list of `macsypy.gene.Gene` objects

`add_accessory_gene`(*gene*)

Add a gene to the list of accessory genes

Parameters **gene** (`macsypy.gene.Gene` object) – gene that is allowed to be present in this system

`add_forbidden_gene`(*gene*)

Add a gene to the list of forbidden genes

Parameters **gene** (`macsypy.genen.Gene` object) – gene that must not be found in this system

`add_mandatory_gene`(*gene*)

Add a gene to the list of mandatory genes

Parameters **gene** (`macsypy.gene.Gene` object) – gene that is mandatory for this system

`forbidden_genes`

Returns the list of genes that are forbidden in this secretion system

Return type list of `macsypy.gene.Gene` objects

`get_gene`(*gene_name*)

Parameters **gene_name** (*string*) – the name of the gene to get

Returns the gene corresponding to gene_name.

Return type a `macsypy.gene.Gene` object.

Raise KeyError the system does not contain any gene with name gene_name.

`get_gene_ref`(*gene*)

Parameters **gene** (a `macsypy.gene.Gene` or macsypy.gene.Homolog‘ or macsypy.gene.Analog‘ object.) – the gene to get the gene reference.

Returns the gene reference of the gene if exists (if the gene is an Homolog or an Analog), otherwise return None.

Return type `macsypy.gene.Gene` object or None

Raise KeyError if gene is not in the system

`inter_gene_max_space`

Returns set the maximum distance allowed between 2 genes for this system

Return type integer

`mandatory_genes`

Returns the list of genes that are mandatory in this secretion system

Return type list of `macsypy.gene.Gene` objects

`max_nb_genes`

Returns the maximum number of genes to assess the system presence.

Return type int (or None)

`min_genes_required`

Returns get the minimum number of genes to assess for the system presence.

Return type integer

`min_mandatory_genes_required`

Returns get the quorum of mandatory genes required for this system

Return type integer

`multi_loci`

Returns True if the system is authorized to be inferred from multiple loci, False otherwise

Return type boolean

CHAPTER

TWELVE

THE PARSER OF SYSTEMS DEFINITIONS

The system parser object "SystemParser" instanciates Systems and Genes objects from XML system definitions (see *Macromolecular systems definition*). The parsing consists in three phases.

Phase 1.

- each Gene is parsed from the System it is defined
- From the list of System to detect, the list of Systems to parse is established

Phase 2.

- For each system to parse
  - create the System
  - add this System to the system_bank
  - create the Genes defined in this System with their attributes but not their Homologs
  - add these Genes in the gene_bank

Phase 3.

- For each System to search
  - For each Gene defined in this System:
    * create the Homologs by encapsulating Genes from the gene_bank
    * add the Gene to the System

For instance:

```
Syst_1
<system inter_gene_max_space="10">
    <gene name=”A” mandatory=”1” loner="1">
        <homologs>
            <gene name=”B” sys_ref=”Syst_2”>
        </homologs>
    </gene>
<system>

Syst_2
<system inter_gene_max_space="15">
    <gene name=”B” mandatory=”1”>
        <homologs>
```

```
            <gene name="B" sys_ref="Syst_1"
            <gene name="C" sys_ref="Syst_3">
        </homologs>
    </gene>
<system>

Syst_3
<system inter_gene_max_space="20">
    <gene name="c" mandatory="1" />
<system>
```

With the example above:

- the Syst_1 has a gene_A
- the gene_A has homolog gene_B
- the gene_B has a reference to Syst_2
- gene_B attributes from the Syst_2 are used to build the Gene
- the Syst_2 has attributes as defined in the corresponding XML file (inter_gene_max_space ,...)

Contrariwise:

- the gene_B has no Homologs
- the Syst_2 has no Genes

Note: The only "full" Systems (*i.e.,* with all corresponding Genes created) are those to detect.

12.1 SystemParser API reference

class `macsypy.system_parser.`**`SystemParser`**(*cfg*, *system_bank*, *gene_bank*)

Build a System instance from the corresponding System definition described in the XML file (named after the system's name) found at the dedicated location ("-d" command-line option).

`__init__`(*cfg*, *system_bank*, *gene_bank*)

Constructor

Parameters

- **cfg** (`macsypy.config.Config` object) – the configuration object of this run
- **system_bank** (`macsypy.system.SystemBank` object) – the system factory
- **gene_bank** (`macsypy.gene.GeneBank` object) – the gene factory

`__weakref__`

list of weak references to the object (if defined)

`_create_genes`(*system*, *system_node*)

Create genes belonging to the systems. Be careful, the returned genes have not their homologs/analogs set yet.

Parameters

- **system** (`macsypy.system.System` object) – the System currently parsing
- **system_node** (:class"*ET.ElementTree* object) – the element gene

Returns a list of the genes belonging to the system.

Return type [macsypy.gene.Gene, ...]

_create_system (*system_name*, *system_node*)

Parameters

- **system_name** – the name of the system to create. This name must match a XML file in the definition directory ("-d" option in the command-line)
- **system_node** (:class"*ET.ElementTree* object.) – the node corresponding to the system.

Returns the system corresponding to the name.

Return type macsypy.system.System object.

_fill (*system*, *system_node*)

Fill the system with genes found in this system definition. Add homologs to the genes if necessary.

Parameters

- **system** (macsypy.system.System object) – the system to fill
- **system_node** (:class"*ET.ElementTree* object) – the "node" in the XML hierarchy corresponding to the system

_parse_analog (*node*, *gene_ref*, *curr_system*)

Parse a xml element gene and build the corresponding object

Parameters

- **node** (xml.etree.ElementTree.Element object.) – a "node" corresponding to the gene element in the XML hierarchy
- **gene_ref** (class:*macsypy.gene.Gene* object.) – the gene which this gene is homolog to

Returns the gene object corresponding to the node

Return type macsypy.gene.Analog object

_parse_homolog (*node*, *gene_ref*, *curr_system*)

Parse a xml element gene and build the corresponding object

Parameters

- **node** (xml.etree.ElementTree.Element object.) – a "node" corresponding to the gene element in the XML hierarchy
- **gene_ref** (class:*macsypy.gene.Gene* object) – the gene which this gene is homolog to

Returns the gene object corresponding to the node

Return type macsypy.gene.Homolog object

check_consistency (*systems*)

Check the consistency of the co-localization features between the different values given as an input: between XML definitions, configuration file, and command-line options.

Parameters **systems** (list of *class:txssnalib.system.System* object) – the list of systems to check

Raise macsypy.macsypy_error.SystemInconsistencyError if one test fails

(see feature)

In the different possible situations, different requirements need to be fulfilled ("mandatory_genes" and "accessory_genes" consist of lists of genes defined as such in the system definition):

- **If:** min_mandatory_genes_required = None ; min_genes_required = None
- **Then:** min_mandatory_genes_required = min_genes_required = len(mandatory_genes)

always True by Systems design

- **If:** min_mandatory_genes_required = value ; min_genes_required = None
- **Then:** min_mandatory_genes_required <= len(mandatory_genes)
- AND min_genes_required = min_mandatory_genes_required

always True by design

- **If:** min_mandatory_genes_required = None ; min_genes_required = Value
- **Then:** min_mandatory_genes_required = len(mandatory_genes)
- AND min_genes_required >= min_mandatory_genes_required
- AND min_genes_required <= len(mandatory_genes+accessory_genes)

to be checked

- **If:** min_mandatory_genes_required = Value ; min_genes_required = Value
- **Then:** min_genes_required <= len(accessory_genes+mandatory_genes)
- AND min_genes_required >= min_mandatory_genes_required
- AND min_mandatory_genes_required <= len(mandatory_genes)

to be checked

parse(*systems_2_detect*)

Parse systems definition in XML format to build the corresponding system objects, and add them to the system factory after checking its consistency. To get the system ask it to system_bank :param systems_2_detect: a list with the names of the systems to parse :type systems_2_detect: list of string

system_to_parse(*sys_2_parse*, *parsed_systems*)

Parameters **sys_2_parse** (*[string, ...]*) – the list of systems to parse

Returns the list of systems' names to parse. Scan the whole chain of 'system_ref' in a recursive way.

Return type [string, ...]

CHAPTER

THIRTEEN

GENE API

The Gene object represents genes encoding the protein components of a System. See *The Gene object* for an overview of the implementation.

> **Warning:** To optimize computation and to avoid concurrency problems when we search several systems, each gene must be instanciated only once, and stored in gene_bank. gene_bank is a `macsypy.gene.GeneBank` object. The gene_bank and system_bank (`macsypy.system.SystemBank` object) are filled by a system_parser (`macsypy.system_parser.SystemParser`)

Example to get a gene object:

```
from macsypy.system import system_bank
from macsypy.config import Config
from macsypy.gene import gene_bank


#get a system
t2ss =  system_bank["T2SS"]

#get of a gene
pilO = gene_bank["pilO"]
```

13.1 GeneBank API reference

class `macsypy.gene.`**`GeneBank`**

Store all Gene objects. Ensure that genes are instanciated only once.

`__contains__`(*gene*)

Implement the membership test operator

Parameters gene (`macsypy.gene.Gene` object) – the gene to test

Returns True if the gene is in, False otherwise

Return type boolean

`__getitem__`(*name*)

Parameters

- **name** (*string*) – the name of the Gene
- **cfg** (`macsypy.config.Config` object) – the configuration object

Returns return the Gene corresponding to the name. If the Gene already exists, return it, otherwise, build it and return it

Return type `macsypy.gene.Gene` object

__iter__()
Return an iterator object on the genes contained in the bank

__weakref__
list of weak references to the object (if defined)

add_gene(*gene*)

Parameters
- **name** (*string*) – the name of the gene
- **cfg** (macsypy.config.Config object) – the configuration

Returns return gene corresponding to the name. If the gene already exists, return it, otherwise, build it and return it

Return type macsypy.gene.Gene object

Raise KeyError if a gene with the same name is already registered

Note: Don't instanciate your own GeneFactory use the gene_bank at the top level of the module.

```
from macsypy.gene import gene_bank
```

13.2 Gene API reference

class macsypy.gene.**Gene**(*cfg*, *name*, *system*, *profiles_registry*, *loner=False*, *exchangeable=False*, *multi_system=False*, *inter_gene_max_space=None*)
Handle Gene of a (secretion) System

__eq__(*gene*)

Returns True if the gene names (gene.name) are the same, False otherwise.

Parameters **gene** (macsypy.gene.Gene object.) – the query of the test

Return type boolean.

__init__(*cfg*, *name*, *system*, *profiles_registry*, *loner=False*, *exchangeable=False*, *multi_system=False*, *inter_gene_max_space=None*)
handle gene

Parameters

- **cfg** (macsypy.config.Config object) – the configuration object.
- **name** (*string.*) – the name of the Gene.
- **system** (macsypy.system.System object.) – the system that owns this Gene
- **profiles_registry** (macsypy.registries.ProfilesRegistry object.) – where all the paths profiles where register.
- **loner** (*boolean.*) – True if the Gene can be isolated on the genome (with no contiguous genes), False otherwise.
- **exchangeable** (*boolean.*) – True if this Gene can be replaced with one of its homologs or analogs whithout any effects on the system assessment, False otherwise.
- **multi_system** (*boolean.*) – True if this Gene can belong to different occurrences of this System.
- **inter_gene_max_space** (*integer*) – the maximum space between this Gene and another gene of the System.

__str__()
Print the name of the gene and of its homologs/analogs.

__weakref__
list of weak references to the object (if defined)

add_analog(*analog*)
Add an analogous gene to the Gene

Parameters **analog** (`macsypy.gene.Analog` object) – analog to add

add_homolog(*homolog*)
Add a homolog gene to the Gene

Parameters **homolog** (`macsypy.gene.Homolog` object) – homolog to add

exchangeable

Returns True if this gene can be replaced with one of its homologs or analogs whithout any effects on the system, False otherwise.

Return type boolean.

get_analogs()

Returns the Gene analogs

Type list of `macsypy.gene.Analog` object

get_compatible_systems(*system_list*, *include_forbidden=True*)
Test every system in system_list for compatibility with the gene using the is_authorized function.

Parameters

- **system_list** (*list of strings*) – a list of system names to test
- **include_forbidden** (*boolean*) – tells if forbidden genes should be considered as defining a compatible systems or not

Returns the list of compatible systems

Return type list of string, or void list if none compatible

get_homologs()

Returns the Gene homologs

Type list of `macsypy.gene.Homolog` object

inter_gene_max_space

Returns The maximum distance allowed between this gene and another gene for them to be considered co-localized. If the value is not set at the Gene level, return the value set at the System level.

Return type integer.

is_accessory(*system*)

Returns True if the gene is within the *accessory* genes of the system, False otherwise.

Parameters **system** (`macsypy.system.System` object.) – the query of the test

Return type boolean.

is_analog(*gene*)

Returns True if the two genes are analogs, False otherwise.

Parameters **gene** (macsypy.gene.Gene object.) – the query of the test

Return type boolean.

is_authorized(*system*, *include_forbidden=True*)

Returns True if the genes are found in the System definition file (.xml), False otherwise.

Parameters

- **system** (macsypy.system.System object.) – the query of the test
- **include_forbidden** (*boolean*) – tells if forbidden genes should be considered as "authorized" or not

Return type boolean.

is_forbidden(*system*)

Returns True if the gene is within the *forbidden* genes of the system, False otherwise.

Parameters **system** (macsypy.system.System object.) – the query of the test

Return type boolean.

is_homolog(*gene*)

Returns True if the two genes are homologs, False otherwise.

Parameters **gene** (macsypy.gene.Gene object.) – the query of the test

Return type boolean.

is_mandatory(*system*)

Returns True if the gene is within the *mandatory* genes of the system, False otherwise.

Parameters **system** (macsypy.system.System object.) – the query of the test

Return type boolean.

loner

Returns True if the gene can be isolated on the genome, False otherwise

Return type boolean

multi_system

Returns True if this Gene can belong to different occurrences of **this System** (and can be used for multiple System assessments), False otherwise.

Return type boolean.

profile = None

Variables **profile** – The HMM protein Profile corresponding to this gene macsypy.gene.Profile object

system

Returns the System that owns this Gene

Return type macsypy.system.System object

Note: All attributes/methods which are not directly implemented in Homolog are redirected to that of the encapsulated Gene.

13.3 Homolog API reference

class macsypy.gene.**Homolog**(*gene*, *gene_ref*, *aligned=False*)

Handle homologs, encapsulate a Gene

__init__(*gene*, *gene_ref*, *aligned=False*)

Parameters

- **gene** (macsypy.gene.Gene object.) – the gene
- **gene_ref** (macsypy.gene.Gene object.) – the gene to which the current is homolog.
- **aligned** (*boolean*) – if True, the profile of this gene overlaps totally the sequence of the reference gene profile. Otherwise, only partial overlapping between the profiles.

__weakref__

list of weak references to the object (if defined)

gene = None

Variables **gene** – gene

gene_ref

Returns the gene to which this one is homolog to (reference gene)

Return type macsypy.gene.Gene object

is_aligned()

Returns True if this gene homolog is aligned to its homolog, False otherwise.

Return type boolean

13.4 Analog API reference

class macsypy.gene.**Analog**(*gene*, *gene_ref*)

Handle analogs, encapsulate a Gene

__init__(*gene*, *gene_ref*)

Parameters

- **gene** (macsypy.gene.Gene object.) – the gene
- **gene_ref** (macsypy.gene.Gene object.) – the gene to which the current is analog.

__weakref__

list of weak references to the object (if defined)

gene = None

Variables **gene** – gene

gene_ref

Returns the gene to which this one is analog to (reference gene)

Return type macsypy.gene.Gene object

CHAPTER

FOURTEEN

PROFILE API

The *Profile object* is used for the search of the gene with Hmmer. A *"Profile"* must match a HMM protein profile file, which name is based on the profile name. For instance, the *gspG* gene has the corresponding "gspG.hmm" profile file provided at a dedicated location.

14.1 ProfileFactory API reference

class `macsypy.gene.`**`ProfileFactory`**

Build and store all Profile objects. Profiles must not be instanciated directly. The profile_factory must be used. The profile_factory ensures there is only one instance of profile for a given name. To get a profile, use the method get_profile. If the profile is already cached, this instance is returned. Otherwise a new profile is built, stored in the profile_factory and then returned.

`get_profile`(*gene*, *cfg*, *profiles_registry*)

Parameters

- **gene** (`macsypy.gene.Gene` or `macsypy.gene.Homolog` or `macsypy.gene.Analog` object) – the gene associated to this profile
- **profiles_registry** (*the registry of profiles*) – the registry where are stored the path of the profiles
- **profiles_registry** – `macsypy.registries.ProfilesRegistry` instance.

Returns the profile corresponding to the name. If the profile already exists, return it. Otherwise build it, store it and return it.

Return type `macsypy.gene.Profile` object

14.2 Profile API reference

class `macsypy.gene.`**`Profile`**(*gene*, *cfg*, *path*)

Handle a HMM protein profile

`__init__`(*gene*, *cfg*, *path*)

Parameters

- **gene** – the gene corresponding to this profile
- **cfg** (`macsypy.config.Config` object) – the configuration
- **path** (*string*) – the path to the hmm profile.

`__len__`()

Returns the length of the HMM protein profile

Return type int

`__str__``()`
Print the name of the corresponding gene and the path to the HMM profile.

`__weakref__`
list of weak references to the object (if defined)

`_len``()`
Parse the HMM profile file to get and store the length. This private method is called at the Profile init.

`execute``()`
Launch the Hmmer search (hmmsearch executable) with this profile

Returns an object storing information on th results of the HMM search (HMMReport)

Return type `macsypy.report.HMMReport` object

CHAPTER

FIFTEEN

HMMREPORT API

A *"HMMReport"* object represents the results of a Hmmer program search on a dataset with a hidden Markov model protein profile (see *this section*). This object has methods to extract and filter Hmmer raw outputs (see *generated output files*), and then build Hits relevant for system detection. For matches selected with the filtering parameters, *"Hit"* objects (`macsypy.HMMReport.Hit`) are built.

15.1 HMMReport API reference

class `macsypy.report.`**`HMMReport`**(*gene*, *hmmer_output*, *cfg*)

Handle the results from the HMM search. Extract a synthetic report from the raw hmmer output, after having applied a hit filtering. This class is an **abstract class**. There are two implementations of this abstract class depending on wether the input sequence dataset is "ordered" ("gembase" or "ordered_replicon" db_type) or not ("unordered" or "unordered_replicon" db_type).

`__init__`(*gene*, *hmmer_output*, *cfg*)

Parameters

- **gene** (`macsypy.gene.Gene` object) – the gene corresponding to the profile search reported here
- **hmmer_output** (*string*) – The path to the raw Hmmer output file
- **cfg** (`macsypy.config.Config` object) – the configuration object

`__metaclass__`

alias of `ABCMeta`

`__str__`()

Print information on filtered hits

`__weakref__`

list of weak references to the object (if defined)

`_build_my_db`(*hmm_output*)

Build the keys of a dictionary object to store sequence identifiers of hits.

Parameters **hmm_output** (*string*) – the path to the hmmsearch output to parse.

Returns a dictionary containing a key for each sequence id of the hits

Return type dict

`_fill_my_db`(*macsyfinder_idx*, *db*)

Fill the dictionary with information on the matched sequences

Parameters

- **macsyfinder_idx** (*string*) – the path the macsyfinder index corresponding to the dataset
- **db** (*dict*) – the database containing all sequence id of the hits.

`_hit_start` (*line*)

Parameters **line** (*string*) – the line to parse

Returns True if it's the begining of a new hit in Hmmer raw output files. False otherwise

Return type boolean.

`_parse_hmm_body` (*hit_id*, *gene_profile_lg*, *seq_lg*, *coverage_treshold*, *replicon_name*, *position_hit*, *i_evalue_sel*, *b_grp*)

Parse the raw Hmmer output to extract the hits, and filter them with threshold criteria selected ("coverage_profile" and "i_evalue_select" command-line parameters)

Parameters

- **hit_id** (*string*) – the sequence identifier
- **gene_profile_lg** (*integer*) – the length of the profile matched
- **seq_lg** (*integer*) – the length of the sequence
- **coverage_treshold** (*float*) – the minimal coverage of the profile to be reached in the Hmmer alignment for hit selection
- **replicon_name** (*string*) – the identifier of the replicon
- **position_hit** (*integer*) – the rank of the sequence matched in the input dataset file
- **i_evalue_sel** (*float*) – the maximal i-evalue (independent evalue) for hit selection
- **b_grp** (*list of list of strings*) – the Hmmer output lines to deal with (grouped by hit)

Returns a set of hits

Return type list of `macsypy.report.Hit` objects

`_parse_hmm_header` (*h_grp*)

Parameters **h_grp** (*sequence of string*) – the sequence of string return by groupby function representing the header of a hit

Returns the sequence identifier from a set of lines that corresponds to a single hit

Return type string

`best_hit` ()

Return the best hit among multiple hits

`extract` ()

Parse the raw Hmmer output file and produce a new synthetic report file by applying a filter on hits. Contain selected and sorted hits (**this abstract method is implemented in inherited classes**)

`save_extract` ()

Write the string representation of the extact report in a file. The name of this file is the concatenation of the gene name and of the "res_extract_suffix" from the config object

15.2 GeneralHMMReport API reference

class `macsypy.report.`**`GeneralHMMReport`**(*gene*, *hmmer_output*, *cfg*)

Handle HMM report. Extract a synthetic report from the raw hmmer output. Dedicated to any type of 'unordered' datasets.

`extract`()

Parse the output file of hmmer compute from an unordered genes base and produced a new synthetic report file.

15.3 OrderedHMMReport

class `macsypy.report.`**`OrderedHMMReport`**(*gene*, *hmmer_output*, *cfg*)

Handle HMM report. Extract a synthetic report from the raw hmmer output. Dedicated to 'ordered_replicon' datasets.

`extract`()

Parse the output file of Hmmer obtained from a search in an ordered set of sequences and produce a new synthetic report file.

15.4 GembaseHMMReport

class `macsypy.report.`**`GembaseHMMReport`**(*gene*, *hmmer_output*, *cfg*)

Handle HMM report. Extract a synthetic report from the raw hmmer output. Dedicated to 'gembase' format datasets.

`extract`()

Parse the output file of Hmmer obtained from a search in a 'gembase' set of sequences and produce a new synthetic report file.

15.5 Hit

class `macsypy.report.`**`Hit`**(*gene*, *system*, *hit_id*, *hit_seq_length*, *replicon_name*, *position_hit*, *i_eval*, *score*, *profile_coverage*, *sequence_coverage*, *begin_match*, *end_match*)

Handle the hits filtered from the Hmmer search. The hits are instanciated by `HMMReport.extract()` method

`__cmp__`(*other*)

Compare two Hits. If the sequence identifier is the same, do the comparison on the score. Otherwise, do it on alphabetical comparison of the sequence identifier.

Parameters **other** (`macsypy.report.Hit` object) – the hit to compare to the current object

Returns the result of the comparison

`__eq__`(*other*)

Return True if two hits are totally equivalent, False otherwise.

Parameters **other** (`macsypy.report.Hit` object) – the hit to compare to the current object

Returns the result of the comparison

Return type boolean

__init__(*gene*, *system*, *hit_id*, *hit_seq_length*, *replicon_name*, *position_hit*, *i_eval*, *score*, *profile_coverage*, *sequence_coverage*, *begin_match*, *end_match*)

Parameters

- **gene** (`macsypy.gene.Gene` object) – the gene corresponding to this profile
- **system** (`macsypy.system.System` object) – the system to which this gene belongs
- **hit_id** (*string*) – the identifier of the hit
- **hit_seq_length** (*integer*) – the length of the hit sequence
- **replicon_name** (*string*) – the name of the replicon
- **position_hit** (*integer*) – the rank of the sequence matched in the input dataset file
- **i_eval** (*float*) – the best-domain evalue (i-evalue, "independent evalue")
- **score** (*float*) – the score of the hit
- **profile_coverage** (*float*) – percentage of the profile that matches the hit sequence
- **sequence_coverage** (*float*) – percentage of the hit sequence that matches the profile
- **begin_match** (*integer*) – where the hit with the profile starts in the sequence
- **end_match** (*integer*) – where the hit with the profile ends in the sequence

__str__()
Print useful information on the Hit: regarding Hmmer statistics, and sequence information

__weakref__
list of weak references to the object (if defined)

get_position()

Returns the position of the hit (rank in the input dataset file)

Return type integer

get_syst_inter_gene_max_space()

Returns the 'inter_gene_max_space' parameter defined for the gene of the hit

Return type integer

CHAPTER

SIXTEEN

SEARCH_GENES API REFERENCE

macsypy.search_genes.**search_genes**(*genes*, *cfg*)

For each gene of the list, use the corresponding profile to perform an Hmmer search, and parse the output to generate a HMMReport that is saved in a file after Hit filtering. These tasks are performed in parallel using threads. The number of workers can be limited by worker_nb directive in the config object or in the command-line with the "-w" option.

Parameters

- **genes** (list of macsypy.gene.Gene objects) – the genes to search in the input sequence dataset
- **cfg** (macsypy.config.Config object) – the configuration object

CHAPTER

SEVENTEEN

SEARCH_SYSTEMS API REFERENCE

class macsypy.search_systems.**Cluster**(*systems_to_detect*)

Stores a set of contiguous hits. The Cluster object can have different states regarding its content in different genes' systems:

- ineligible: not a cluster to analyze
- clear: a single system is represented in the cluster
- ambiguous: several systems are represented in the cluster => might need a disambiguation

__init__(*systems_to_detect*)

Parameters **systems_to_detect** (a list of macsypy.system.System) – the list of systems to be detected in this run

__len__()

Returns the length of the Cluster, *i.e.*, the number of hits stored in it

Return type integer

__str__()

print of the Cluster's hits stored in terms of components, and corresponding sequence identifier and positions

__weakref__

list of weak references to the object (if defined)

add(*hit*)

Add a Hit to a Cluster. Hits are always added at the end of the cluster (appended to the list of hits). Thus, 'begin' and 'end' positions of the Cluster are always the position of the 1st and of the last hit respectively.

Parameters **hit** (a macsypy.report.Hit) – the Hit to add

Raise a macsypy.macsypy_error.SystemDetectionError

compatible_systems

Returns the list of the names of compatible systems represented by the cluster

Return type string

putative_system

Returns the name of the putative system represented by the cluster

Return type string

save (*force=False*)
Check the status of the cluster regarding systems which have hits in it. Update systems represented, and assign a putative system (self._putative_system), which is the system with most hits in the cluster. The systems represented are stored in a dictionary in the self.systems variable. The execution of this function can be forced, even if it has already run for the cluster with the option force=True.

state

Returns the state of the Cluster of hits

Return type string

class macsypy.search_systems.**ClustersHandler**
Deals with sets of clusters found in a dataset. Conceived to store only clusters from a same replicon.

__init__ ()

Parameters **cfg** (macsypy.config.Config) – The configuration object built from default and user parameters.

__weakref__
list of weak references to the object (if defined)

circularize (*rep_info*, *end_hits*, *systems_to_detect*)
This function takes into account the circularity of the replicon by merging clusters when appropriate (typically at replicon's ends). It has to be called only if the replicon_topology is set to "circular".

Parameters

- **rep_info** (a namedTuple "RepliconInfo" macsypy.database.RepliconInfo) – an entry extracted from the macsypy.database.RepliconDB
- **end_hits** (a list of macsypy.report.Hit) – a set of hits at ends of the replicon that were not introduced in clusters, and that might be part of a system overlapping the two "ends" of the replicon
- **systems_to_detect** (a list of :class:'macsypy.system.System) – the set of systems to detect in this run

class macsypy.search_systems.**SystemNameGenerator**
Creates and stores the names of detected systems. Ensures the uniqueness of the names.

__weakref__
list of weak references to the object (if defined)

_computeBasename (*replicon*, *system*)
Computes the base name to be used for unique name generation

Parameters

- **replicon** (*string*) – the replicon name
- **system** (*string*) – the system name

Returns the base name

Return type string

getSystemName (*replicon*, *system*)
Generates a unique system name based on the replicon's name and the system's name.

Parameters

- **replicon** (*string*) – the replicon name
- **system** (*string*) – the system name

Returns a unique system name

Return type string

class macsypy.search_systems.**SystemOccurence**(*system*)

This class is instantiated for a specific system that has been asked for detection. It can be filled step by step with hits. A decision can then be made according to the parameters defined *e.g.* quorum of genes.

The SystemOccurence object has a "state" parameter, with the possible following values:

- "empty" if the SystemOccurence has not yet been filled with genes of the decision rule of the system
- "no_decision" if the filling process has started but the decision rule has not yet been applied to this occurence
- "single_locus"
- "multi_loci"
- "uncomplete"

__init__(*system*)

Parameters **system** (macsypy.system.System) – the system to "fill" with hits.

__str__()

Returns Information of the component content of the SystemOccurence.

Return type string

__weakref__

list of weak references to the object (if defined)

compute_missing_genes_list(*gene_dict*)

Parameters **gene_dict** (*dict*) – a dictionary with gene's names as keys and number of occurrences as values

Returns the list of genes with no occurence in the gene counter.

Return type list

compute_system_length(*rep_info*)

Returns the length of the system, all loci gathered, in terms of protein number (even those not matching any system gene)

Parameters **rep_info** (a namedTuple "RepliconInfo" macsypy.database.RepliconInfo) – an entry extracted from the macsypy.database.RepliconDB

Return type integer

count_genes(*gene_dict*)

Counts the number of genes with at least one occurrence in a dictionary with a counter of genes.

Parameters **gene_dict** (*dict*) – a dictionary with gene's names as keys and number of occurrences as values

Return type integer

count_genes_tot(*gene_dict*)

Counts the number of matches in a dictionary with a counter of genes, independently of the nb of genes matched.

Parameters **gene_dict** (*dict*) – a dictionary with gene's names as keys and number of occurrences as values

Return type integer

count_missing_genes (*gene_dict*)
Counts the number of genes with no occurence in the gene counter.

Parameters **gene_dict** (*dict*) – a dictionary with gene's names as keys and number of occurrences as values

Return type integer

decision_rule ()

This function applies the decision rules for system assessment in terms of quorum:

- the absence of forbidden genes is checked
- the minimal number of mandatory genes is checked ("min_mandatory_genes_required")
- the minimal number of genes in the system is checked ("min_genes_required")

When a decision is made, the status (self.status) of the `macsypy.search_systems.SystemOccurence` is set either to:

- "single_locus" when a complete system in the form of a single cluster was found
- "multi_loci" when a complete system in the form of several clusters was found
- "uncomplete" when no system was assessed (quorum not reached)
- "empty" when no gene for this system was found
- "exclude" when no system was assessed (at least one forbidden gene was found)

Returns a printable message of the output decision with this SystemOccurrence

Return type string

fill_with_cluster (*cluster*)
Adds hits from a cluster to a system occurence, and check which are their status according to the system definition. Set the system occurence state to "no_decision" after calling of this function.

Parameters **cluster** (`macsypy.search_systems.Cluster`) – the set of contiguous genes to treat for `macsypy.search_systems.SystemOccurence` inclusion.

fill_with_hits (*hits*, *include_forbidden*)
Adds hits to a system occurence, and check what are their status according to the system definition. Set the system occurence state to "no_decision" after calling of this function.

Note: Forbidden genes will only be included if they do belong to the current system (and not to another specified with "system_ref" in the current system's definition).

Parameters **hits** – a list of Hits to treat for `macsypy.search_systems.SystemOccurence` inclusion.

fill_with_multi_systems_genes (*multi_systems_hits*)
This function fills the SystemOccurrence with genes putatively coming from other systems (feature "multi_system"). Those genes are used only if the occurrence of the corresponding gene was not yet filled with a gene from a cluster of the system.

Parameters **multi_systems_hits** – a list of hits of genes that are "multi_system" which correspond to mandatory or accessory genes from the current system for which to fill a SystemOccurrence

get_gene_counter_output (*forbid_exclude=False*)

Parameters **forbid_exclude** (*boolean*) – exclude the forbidden components if set to True. False by default.

Returns A dictionary ready for printing in system summary, with genes (mandatory, accessory and forbidden if specified) occurences in the system occurrence.

get_gene_ref (*gene*)

Parameters **gene** (`macsypy.gene.Gene`, or `macsypy.gene.Homolog` or `macsypy.gene.Analog` object) – the gene to get it's gene reference

Returns object `macsypy.gene.Gene` or None

Return type `macsypy.gene.Gene` object or None

Raise KeyError if the system does not contain any gene gene.

get_summary (*replicon_name*, *rep_info*)

Gives a summary of the system occurrence in terms of gene content and localization.

Parameters

- **replicon_name** (*string*) – the name of the replicon
- **rep_info** (a namedTuple "RepliconInfo" `macsypy.database.RepliconInfo`) – an entry extracted from the `macsypy.database.RepliconDB`

Returns a tabulated summary of the `macsypy.search_systems.SystemOccurence`

Return type string

get_summary_header ()

Returns a string with the description of the summary returned by self.get_summary()

Return type string

get_summary_unordered (*replicon_name*)

Gives a summary of the system occurrence in terms of gene content only (specific of "unordered" datasets).

Parameters **replicon_name** (*string*) – the name of the replicon

Returns a tabulated summary of the `macsypy.search_systems.SystemOccurence`

Return type string

get_system_name_unordered (*suffix='_putative'*)

Attributes a name to the system occurrence for an "unordered" dataset => generating a generic name based on the system name and the suffix given.

Parameters **suffix** (*string*) – the suffix to be used for generating the systemOccurrence's name

Returns a name for a system in an "unordered" dataset to the `macsypy.search_systems.SystemOccurence`

Return type string

get_system_unique_name (*replicon_name*)

Attributes unique name to the system occurrence with the class `macsypy.search_systems.SystemNameGenerator`. Generates the name if not already set.

Parameters **replicon_name** (*string*) – the name of the replicon

Returns the unique name of the `macsypy.search_systems.SystemOccurence`

Return type string

is_complete()

Test for SystemOccurrence completeness.

Returns True if the state of the SystemOccurrence is "single_locus" or "multi_loci", False otherwise.

Return type boolean

nb_syst_genes

This value is set after a decision was made on the system in `macsypy.search_systems.SystemOccurence:decision_rule()`

Returns the number of mandatory and accessory genes with at least one occurence (number of different accessory genes)

Return type integer

state

Returns the state of the systemOccurrence.

Return type string

`macsypy.search_systems.`**analyze_clusters_replicon**(*clusters*, *systems*, *multi_systems_genes*)

Analyzes sets of contiguous hits (clusters) stored in a ClustersHandler for system detection:

- split clusters if needed
- delete them if they are not relevant
- add eventual genes from other systems "multi_system" genes
- check the QUORUM for each system to detect, *i.e.* mandatory + accessory - forbidden

Only for "ordered" datasets representing a whole replicon. Reports systems occurence.

Parameters

- **clusters** (`macsypy.search_systems.ClustersHandler`) – the set of clusters to analyze
- **systems** (a list of `macsypy.system.System`) – the set of systems to detect
- **multi_systems_genes** – a dictionary with genes that could belong to multiple systems (keys are system names)

Returns a set of systems occurence filled with hits found in clusters

Return type a list of `macsypy.search_systems.SystemOccurence`

`macsypy.search_systems.`**build_clusters**(*hits*, *systems_to_detect*, *rep_info*)

Gets sets of contiguous hits according to the minimal inter_gene_max_space between two genes. Only for "ordered" datasets.

Parameters

- **hits** (a list of `macsypy.report.Hit`) – a list of Hmmer hits to analyze
- **systems_to_detect** (a list of `macsypy.system.System`) – the list of systems to detect
- **cfg** (`macsypy.config.Config`) – the configuration object built from default and user parameters.
- **rep_info** (a namedTuple "RepliconInfo" `macsypy.database.RepliconInfo`) – an entry extracted from the `macsypy.database.RepliconDB`

Returns a set of clusters and a dictionary with "multi_system" genes stored in a system-wise way for further utilization.

Return type macsypy.search_systems.ClustersHandler

macsypy.search_systems.**disambiguate_cluster**(*cluster*)

This disambiguation step is used on clusters with hits for multiple systems (when cluster.state is set to "ambiguous"). It returns a "cleansed" list of clusters, ready to use for system occurence detection (and that are "clear" cases). It:

- splits the cluster in two if it seems that two systems are nearby
- removes single hits that are not forbidden for the "main" system and that are at one end of the current cluster in this case, check that they are not "loners", cause "loners" can be stored.

Parameters **cluster** (macsypy.search_systems.Cluster) – the cluster to "disambiguate"

macsypy.search_systems.**get_best_hits**(*hits*, *tosort=False*, *criterion='score'*)

Returns from a putatively redundant list of hits, a list of best matching hits. Analyzes quorum and co-localization if required for system detection. By default, hits are already sorted by position, and the hit with the best score is kept, then the best i-evalue. Possible criteria are:

- maximal score (criterion="score")
- minimal i-evalue (criterion="i_eval")
- maximal percentage of the profile covered by the alignment with the query sequence (criterion="profile_coverage")

Parameters

- **tosort** (*boolean*) – tells if the hits have to be sorted
- **criterion** (*string*) – the criterion to base the sorting on

Returns the list of best matching hits

Return type list of macsypy.report.Hit

Raise a macsypy.macsypy_error.MacsypyError

macsypy.search_systems.**get_compatible_systems**(*systems_list1*, *systems_list2*)

Returns the intersection of the two input systems lists.

Parameters **systems_list2** (*systems_list1,*) – two lists of systems

Returns a list of systems, or an empty list if no common system

Return type a list of macsypy.system.System

macsypy.search_systems.**search_systems**(*hits*, *systems*, *cfg*)

Runs search of systems from a set of hits. Criteria for system assessment will depend on the kind of input dataset provided:

- analyze **quorum and co-localization** for "ordered_replicon" and "gembase" datasets.
- analyze **quorum only** (and in a limited way) for "unordered_replicon" and "unordered" datasets.

Parameters

- **hits** (list of macsypy.report.Hit) – the list of hits for input systems components
- **systems** (list of macsypy.system.System) – the list of systems asked for detection

- **cfg** (`macsypy.config.Config`) – the configuration object

class `macsypy.search_systems.`**`systemDetectionReportOrdered`**(*replicon_name*, *systems_occurences_list*, *cfg*)

Stores the detected systems to report for each replicon:

- by system name,
- by state of the systems (single vs multi loci)

`__init__`(*replicon_name*, *systems_occurences_list*, *cfg*)

Parameters

- **replicon_name** (*string*) – the name of the replicon
- **systems_occurences_list** (list of `macsypy.search_systems.SystemOccurence`) – the list of system's occurrences to consider

`_match2json`(*valid_hit*, *so*)

Parameters

- **valid_hit** (class:*macsypy.search_system.ValidHit* object.) – the valid hit to transform in to json.
- **so** (class:*macsypy.search_system.SystemOccurence.*) – the system occurence where the valid hit come from.

`counter_output`()

Builds a counter of systems per replicon, with different "states" separated (single-locus vs multi-loci systems)

Returns the counter of systems

Return type Counter

`json_output`(*json_path*, *json_data*)

`report_output`(*reportfilename*, *print_header=False*)

Writes a report of sequences forming the detected systems, with information in their status in the system, their localization on replicons, and statistics on the Hits.

Parameters

- **reportfilename** (*string*) – the output file name
- **print_header** (*boolean*) – True if the header has to be written. False otherwise

`summary_output`(*reportfilename*, *rep_info*, *print_header=False*)

Writes a report with the summary of systems detected in replicons. For each system, a summary is done including:

- the number of mandatory/accessory genes in the reference system (as defined in XML files)
- the number of mandatory/accessory genes detected
- the number and list of missing genes
- the number of loci encoding the system

Parameters

- **rep_info** (a namedTuple "RepliconInfo" `macsypy.database.RepliconInfo`) – an entry extracted from the `macsypy.database.RepliconDB`

- **print_header** (*boolean*) – True if the header has to be written. False otherwise

system_2_json (*rep_db*)
Generates the report in json format

Parameters

- **path** (*string*) – the path to a file where to write the report in json format
- **rep_db** (a class:*macsypy.database.RepliconDB* object) – the replicon database

tabulated_output (*system_occurence_states*, *system_names*, *reportfilename*, *print_header=False*)
Write a tabulated output with number of detected systems for each replicon.

Parameters

- **system_occurence_states** (*list of string*) – the different forms of detected systems to consider
- **reportfilename** (*string*) – the output file name
- **print_header** (*boolean*) – True if the header has to be written. False otherwise

Return type string

tabulated_output_header (*system_occurence_states*, *system_names*)
Returns a string containing the header of the tabulated output

Parameters **system_occurence_states** (*list of string*) – the different forms of detected systems to consider

Return type string

class macsypy.search_systems.**systemDetectionReportUnordered** (*systems_occurences_list*, *cfg*)

Stores a report for putative detected systems gathering all hits from a search in an unordered dataset:

- by system.

Mandatory and accessory genes only are reported in the "json" and "report" output, but all hits matching a system component are reported in the "summary".

__init__ (*systems_occurences_list*, *cfg*)

Parameters **systems_occurences_list** (list of macsypy.search_systems.SystemOccurence) – the list of system's occurrences to consider

json_output (*json_path*)
Generates the report in json format

Parameters **path** (*string*) – the path to a file where to write the report in json format

report_output (*reportfilename*, *print_header=False*)
Writes a report of sequences forming the detected systems, with information in their status in the system, their localization on replicons, and statistics on the Hits.

Parameters

- **reportfilename** (*string*) – the output file name
- **print_header** (*boolean*) – True if the header has to be written. False otherwise

summary_output (*reportfilename*, *print_header=False*)
Writes a report with the summary for putative systems in an unordered dataset. For each system, a summary is done including:

- the number of mandatory/accessory genes in the reference system (as defined in XML files)
- the number of mandatory/accessory genes detected

Parameters

- **reportfilename** (*string*) – the output file name
- **print_header** (*boolean*) – True if the header has to be written. False otherwise

class `macsypy.search_systems.`**`validSystemHit`**(*hit*, *detected_system*, *gene_status*)

Encapsulates a `macsypy.report.Hit` This class stores a Hit that has been attributed to a detected system. Thus, it also stores:

- the system,
- the status of the gene in this system,

It also aims at storing information for results extraction:

- system extraction (e.g. genomic positions)
- sequence extraction

`__init__`(*hit*, *detected_system*, *gene_status*)

Parameters

- **hit** (`macsypy.report.Hit`) – a hit to base the validSystemHit on
- **detected_system** (*string*) – the name of the predicted System
- **gene_status** (*string*) – the "role" of the gene in the predicted system

`__weakref__`

list of weak references to the object (if defined)

`output_system_header`()

Returns the header for the output file

Return type string

CHAPTER

EIGHTEEN

MACSYFINDER ERRORS

18.1 macsypy_error API reference

exception `macsypy.macsypy_error.`**`MacsypyError`**

The base class for MacSyFinder specific exceptions.

`__weakref__`

list of weak references to the object (if defined)

exception `macsypy.macsypy_error.`**`SystemDetectionError`**

Raised when the detection of systems from Hits encountered a problem.

exception `macsypy.macsypy_error.`**`SystemInconsistencyError`**

Raised when a secretion system is not consistent.

Part IV

Indices and tables

- *genindex*
- *modindex*
- *search*

PYTHON MODULE INDEX

m

macsypy.config, 41
macsypy.database, 47
macsypy.gene, 65
macsypy.macsypy_error, 83
macsypy.report, 69
macsypy.search_genes, 71
macsypy.search_systems, 73
macsypy.system, 52
macsypy.system_parser, 56

INDEX

Symbols

__cmp__() (macsypy.report.Hit method), 69
__contains__() (macsypy.database.RepliconDB method), 48
__contains__() (macsypy.gene.GeneBank method), 59
__contains__() (macsypy.system.SystemBank method), 51
__eq__() (macsypy.gene.Gene method), 60
__eq__() (macsypy.report.Hit method), 69
__getitem__() (macsypy.database.RepliconDB method), 48
__getitem__() (macsypy.gene.GeneBank method), 59
__getitem__() (macsypy.system.SystemBank method), 51
__getnewargs__() (macsypy.database.RepliconInfo method), 49
__init__() (macsypy.config.Config method), 41
__init__() (macsypy.database.Indexes method), 47
__init__() (macsypy.database.RepliconDB method), 48
__init__() (macsypy.gene.Analog method), 63
__init__() (macsypy.gene.Gene method), 60
__init__() (macsypy.gene.Homolog method), 63
__init__() (macsypy.gene.Profile method), 65
__init__() (macsypy.report.HMMReport method), 67
__init__() (macsypy.report.Hit method), 69
__init__() (macsypy.search_systems.Cluster method), 73
__init__() (macsypy.search_systems.ClustersHandler method), 74
__init__() (macsypy.search_systems.SystemOccurence method), 75
__init__() (macsypy.search_systems.systemDetectionReportOrdered method), 80
__init__() (macsypy.search_systems.systemDetectionReportUnordered method), 81
__init__() (macsypy.search_systems.validSystemHit method), 82
__init__() (macsypy.system.System method), 52
__init__() (macsypy.system_parser.SystemParser method), 56
__iter__() (macsypy.gene.GeneBank method), 59
__iter__() (macsypy.system.SystemBank method), 51
__len__() (macsypy.gene.Profile method), 65
__len__() (macsypy.search_systems.Cluster method), 73
__len__() (macsypy.system.SystemBank method), 51
__metaclass__ (macsypy.report.HMMReport attribute), 67
__new__() (macsypy.database.RepliconInfo static method), 49
__repr__() (macsypy.database.RepliconInfo method), 49
__str__() (macsypy.gene.Gene method), 60
__str__() (macsypy.gene.Profile method), 66
__str__() (macsypy.report.HMMReport method), 67
__str__() (macsypy.report.Hit method), 70
__str__() (macsypy.search_systems.Cluster method), 73
__str__() (macsypy.search_systems.SystemOccurence method), 75
__weakref__ (macsypy.config.Config attribute), 42
__weakref__ (macsypy.database.Indexes attribute), 47
__weakref__ (macsypy.database.RepliconDB attribute), 48
__weakref__ (macsypy.gene.Analog attribute), 63
__weakref__ (macsypy.gene.Gene attribute), 61
__weakref__ (macsypy.gene.GeneBank attribute), 60
__weakref__ (macsypy.gene.Homolog attribute), 63
__weakref__ (macsypy.gene.Profile attribute), 66
__weakref__ (macsypy.macsypy_error.MacsypyError attribute), 83
__weakref__ (macsypy.report.HMMReport attribute), 67
__weakref__ (macsypy.report.Hit attribute), 70
__weakref__ (macsypy.search_systems.Cluster attribute), 73
__weakref__ (macsypy.search_systems.ClustersHandler attribute), 74
__weakref__ (macsypy.search_systems.SystemNameGenerator attribute), 74
__weakref__ (macsypy.search_systems.SystemOccurence attribute), 75
__weakref__ (macsypy.search_systems.validSystemHit attribute), 82
__weakref__ (macsypy.system.System attribute), 52
__weakref__ (macsypy.system.SystemBank attribute), 51
__weakref__ (macsypy.system_parser.SystemParser attribute), 56
_asdict() (macsypy.database.RepliconInfo method), 49

_build_hmmer_indexes() (macsypy.database.Indexes method), 47
_build_my_db() (macsypy.report.HMMReport method), 67
_build_my_indexes() (macsypy.database.Indexes method), 48
_computeBasename() (macsypy.search_systems.SystemNameGenerator method), 74
_create_genes() (macsypy.system_parser.SystemParser method), 56
_create_system() (macsypy.system_parser.SystemParser method), 57
_fill() (macsypy.system_parser.SystemParser method), 57
_fill_gembase_min_max() (macsypy.database.RepliconDB method), 48
_fill_my_db() (macsypy.report.HMMReport method), 67
_fill_ordered_min_max() (macsypy.database.RepliconDB method), 49
_fill_topology() (macsypy.database.RepliconDB method), 49
_hit_start() (macsypy.report.HMMReport method), 68
_len() (macsypy.gene.Profile method), 66
_make() (macsypy.database.RepliconInfo class method), 49
_match2json() (macsypy.search_systems.systemDetectionReportOrdered method), 80
_parse_analog() (macsypy.system_parser.SystemParser method), 57
_parse_hmm_body() (macsypy.report.HMMReport method), 68
_parse_hmm_header() (macsypy.report.HMMReport method), 68
_parse_homolog() (macsypy.system_parser.SystemParser method), 57
_replace() (macsypy.database.RepliconInfo method), 49
_validate() (macsypy.config.Config method), 42

A

accessory_genes (macsypy.system.System attribute), 52
add() (macsypy.search_systems.Cluster method), 73
add_accessory_gene() (macsypy.system.System method), 52
add_analog() (macsypy.gene.Gene method), 61
add_forbidden_gene() (macsypy.system.System method), 52
add_gene() (macsypy.gene.GeneBank method), 60
add_homolog() (macsypy.gene.Gene method), 61
add_mandatory_gene() (macsypy.system.System method), 52
add_system() (macsypy.system.SystemBank method), 51
Analog (class in macsypy.gene), 63
analyze_clusters_replicon() (in module macsypy.search_systems), 78

B

best_hit() (macsypy.report.HMMReport method), 68
build() (macsypy.database.Indexes method), 48
build_clusters() (in module macsypy.search_systems), 78
build_indexes (macsypy.config.Config attribute), 42

C

check_consistency() (macsypy.system_parser.SystemParser method), 57
circularize() (macsypy.search_systems.ClustersHandler method), 74
Cluster (class in macsypy.search_systems), 73
ClustersHandler (class in macsypy.search_systems), 74
compatible_systems (macsypy.search_systems.Cluster attribute), 73
compute_missing_genes_list() (macsypy.search_systems.SystemOccurence method), 75
compute_system_length() (macsypy.search_systems.SystemOccurence method), 75
Config (class in macsypy.config), 41
count_genes() (macsypy.search_systems.SystemOccurence method), 75
count_genes_tot() (macsypy.search_systems.SystemOccurence method), 75
count_missing_genes() (macsypy.search_systems.SystemOccurence method), 76
counter_output() (macsypy.search_systems.systemDetectionReportOrdered method), 80
coverage_profile (macsypy.config.Config attribute), 43

D

db_type (macsypy.config.Config attribute), 43
decision_rule() (macsypy.search_systems.SystemOccurence method), 76
def_dir (macsypy.config.Config attribute), 43
disambiguate_cluster() (in module macsypy.search_systems), 79

E

e_value_res (macsypy.config.Config attribute), 43
exchangeable (macsypy.gene.Gene attribute), 61
execute() (macsypy.gene.Profile method), 66
extract() (macsypy.report.GembaseHMMReport method), 69
extract() (macsypy.report.GeneralHMMReport method), 69
extract() (macsypy.report.HMMReport method), 68

extract() (macsypy.report.OrderedHMMReport method), 69

F

fasta_iter() (in module macsypy.database), 50
fill_with_cluster() (macsypy.search_systems.SystemOccurence method), 76
fill_with_hits() (macsypy.search_systems.SystemOccurence method), 76
fill_with_multi_systems_genes() (macsypy.search_systems.SystemOccurence method), 76
find_hmmer_indexes() (macsypy.database.Indexes method), 48
find_my_indexes() (macsypy.database.Indexes method), 48
forbidden_genes (macsypy.system.System attribute), 52

G

GembaseHMMReport (class in macsypy.report), 69
Gene (class in macsypy.gene), 60
gene (macsypy.gene.Analog attribute), 63
gene (macsypy.gene.Homolog attribute), 63
gene_ref (macsypy.gene.Analog attribute), 63
gene_ref (macsypy.gene.Homolog attribute), 63
GeneBank (class in macsypy.gene), 59
GeneralHMMReport (class in macsypy.report), 69
genes (macsypy.database.RepliconInfo attribute), 49
get() (macsypy.database.RepliconDB method), 49
get_analogs() (macsypy.gene.Gene method), 61
get_best_hits() (in module macsypy.search_systems), 79
get_compatible_systems() (in module macsypy.search_systems), 79
get_compatible_systems() (macsypy.gene.Gene method), 61
get_gene() (macsypy.system.System method), 52
get_gene_counter_output() (macsypy.search_systems.SystemOccurence method), 76
get_gene_ref() (macsypy.search_systems.SystemOccurence method), 77
get_gene_ref() (macsypy.system.System method), 53
get_homologs() (macsypy.gene.Gene method), 61
get_position() (macsypy.report.Hit method), 70
get_profile() (macsypy.gene.ProfileFactory method), 65
get_summary() (macsypy.search_systems.SystemOccurence method), 77
get_summary_header() (macsypy.search_systems.SystemOccurence method), 77
get_summary_unordered() (macsypy.search_systems.SystemOccurence method), 77
get_syst_inter_gene_max_space() (macsypy.report.Hit method), 70
get_system_name_unordered() (macsypy.search_systems.SystemOccurence method), 77
get_system_unique_name() (macsypy.search_systems.SystemOccurence method), 77
getSystemName() (macsypy.search_systems.SystemNameGenerator method), 74

H

Hit (class in macsypy.report), 69
hmmer_dir (macsypy.config.Config attribute), 43
hmmer_exe (macsypy.config.Config attribute), 43
HMMReport (class in macsypy.report), 67
Homolog (class in macsypy.gene), 63

I

i_evalue_sel (macsypy.config.Config attribute), 43
index_db_exe (macsypy.config.Config attribute), 43
Indexes (class in macsypy.database), 47
inter_gene_max_space (macsypy.gene.Gene attribute), 61
inter_gene_max_space (macsypy.system.System attribute), 53
inter_gene_max_space() (macsypy.config.Config method), 43
is_accessory() (macsypy.gene.Gene method), 61
is_aligned() (macsypy.gene.Homolog method), 63
is_analog() (macsypy.gene.Gene method), 61
is_authorized() (macsypy.gene.Gene method), 62
is_complete() (macsypy.search_systems.SystemOccurence method), 78
is_forbidden() (macsypy.gene.Gene method), 62
is_homolog() (macsypy.gene.Gene method), 62
is_mandatory() (macsypy.gene.Gene method), 62
items() (macsypy.database.RepliconDB method), 49
iteritems() (macsypy.database.RepliconDB method), 49

J

json_output() (macsypy.search_systems.systemDetectionReportOrdered method), 80
json_output() (macsypy.search_systems.systemDetectionReportUnordered method), 81

L

loner (macsypy.gene.Gene attribute), 62

M

macsypy.config (module), 41
macsypy.database (module), 47
macsypy.gene (module), 59, 60, 63, 65

macsypy.macsypy_error (module), 83
macsypy.report (module), 67, 69
macsypy.search_genes (module), 71
macsypy.search_systems (module), 73
macsypy.system (module), 51, 52
macsypy.system_parser (module), 56
MacsypyError, 83
mandatory_genes (macsypy.system.System attribute), 53
max (macsypy.database.RepliconInfo attribute), 50
max_nb_genes (macsypy.system.System attribute), 53
max_nb_genes() (macsypy.config.Config method), 43
min (macsypy.database.RepliconInfo attribute), 50
min_genes_required (macsypy.system.System attribute), 53
min_genes_required() (macsypy.config.Config method), 44
min_mandatory_genes_required (macsypy.system.System attribute), 53
min_mandatory_genes_required() (macsypy.config.Config method), 44
multi_loci (macsypy.system.System attribute), 53
multi_loci() (macsypy.config.Config method), 44
multi_system (macsypy.gene.Gene attribute), 62

N

nb_syst_genes (macsypy.search_systems.SystemOccurence attribute), 78

O

OrderedHMMReport (class in macsypy.report), 69
output_system_header() (macsypy.search_systems.validSystemHit method), 82

P

parse() (macsypy.system_parser.SystemParser method), 58
previous_run (macsypy.config.Config attribute), 44
Profile (class in macsypy.gene), 65
profile (macsypy.gene.Gene attribute), 62
profile_dir (macsypy.config.Config attribute), 44
profile_suffix (macsypy.config.Config attribute), 44
ProfileFactory (class in macsypy.gene), 65
putative_system (macsypy.search_systems.Cluster attribute), 73

R

replicon_infos() (macsypy.database.RepliconDB method), 49
replicon_names() (macsypy.database.RepliconDB method), 49
replicon_topology (macsypy.config.Config attribute), 44
RepliconDB (class in macsypy.database), 48
RepliconInfo (class in macsypy.database), 49
report_output() (macsypy.search_systems.systemDetectionReportOrdered method), 80
report_output() (macsypy.search_systems.systemDetectionReportUnordered method), 81
res_extract_suffix (macsypy.config.Config attribute), 44
res_search_dir (macsypy.config.Config attribute), 44
res_search_suffix (macsypy.config.Config attribute), 44

S

save() (macsypy.config.Config method), 44
save() (macsypy.search_systems.Cluster method), 73
save_extract() (macsypy.report.HMMReport method), 68
search_genes() (in module macsypy.search_genes), 71
search_systems() (in module macsypy.search_systems), 79
sequence_db (macsypy.config.Config attribute), 45
state (macsypy.search_systems.Cluster attribute), 74
state (macsypy.search_systems.SystemOccurence attribute), 78
summary_output() (macsypy.search_systems.systemDetectionReportOrdered method), 80
summary_output() (macsypy.search_systems.systemDetectionReportUnordered method), 81
System (class in macsypy.system), 52
system (macsypy.gene.Gene attribute), 62
system_2_json() (macsypy.search_systems.systemDetectionReportOrdered method), 81
system_to_parse() (macsypy.system_parser.SystemParser method), 58
SystemBank (class in macsypy.system), 51
SystemDetectionError, 83
systemDetectionReportOrdered (class in macsypy.search_systems), 80
systemDetectionReportUnordered (class in macsypy.search_systems), 81
SystemInconsistencyError, 83
SystemNameGenerator (class in macsypy.search_systems), 74
SystemOccurence (class in macsypy.search_systems), 75
SystemParser (class in macsypy.system_parser), 56

T

tabulated_output() (macsypy.search_systems.systemDetectionReportOrdered method), 81
tabulated_output_header() (macsypy.search_systems.systemDetectionReportOrdered method), 81
topology (macsypy.database.RepliconInfo attribute), 50
topology_file (macsypy.config.Config attribute), 45

V

validSystemHit (class in macsypy.search_systems), 82

W

worker_nb (macsypy.config.Config attribute), 45
working_dir (macsypy.config.Config attribute), 45
