# Supplementary material for: MacSyFinder: A Program to Mine Genomes for Molecular Systems with an Application to CRISPR-Cas Systems
Source: PLoS One. 2014 Oct 17;9(10):e110726. doi: 10.1371/journal.pone.0110726 (PMC4201578; doi:10.1371/journal.pone.0110726)
Supplement: Data S1 — The MacSyFinder/MacSyView package (compressed tarball archive). (GZ) [file pone.0110726.s010.gz › macsyfinder-1.0.0-RC3/macsyview/app/bower_components/raphael.export/tests/index.html]

Raphael.Export Tests


# Raphael.Export Tests

## 

## 


## Visual Tests for SVG Browsers

Left = Raphael, Right = Raphael.Export (via DOM insert)
