# Supplementary material for: MacSyFinder: A Program to Mine Genomes for Molecular Systems with an Application to CRISPR-Cas Systems
Source: PLoS One. 2014 Oct 17;9(10):e110726. doi: 10.1371/journal.pone.0110726 (PMC4201578; doi:10.1371/journal.pone.0110726)
Supplement: Data S1 — The MacSyFinder/MacSyView package (compressed tarball archive). (GZ) [file pone.0110726.s010.gz › macsyfinder-1.0.0-RC3/doc/_build/html/config.html]

Configuration API — MacSyFinder 1.0.0-RC3 documentation


### Navigation

- index
- modules |
- next |
- previous |
- MacSyFinder documentation »

# Configuration API¶

Options to run MacSyFinder can be specified in a *Configuration file*. The API described below handles all configuration options for MacSyFinder. The Config object provides some default values, and performs some validations of the values.

## Config API reference¶

*class* macsypy.config.Config(*cfg\_file=''*, *sequence\_db=None*, *db\_type=None*, *replicon\_topology=None*, *topology\_file=None*, *inter\_gene\_max\_space=None*, *min\_mandatory\_genes\_required=None*, *min\_genes\_required=None*, *max\_nb\_genes=None*, *multi\_loci=None*, *hmmer\_exe=None*, *index\_db\_exe=None*, *e\_value\_res=None*, *i\_evalue\_sel=None*, *coverage\_profile=None*, *def\_dir=None*, *res\_search\_dir=None*, *res\_search\_suffix=None*, *profile\_dir=None*, *profile\_suffix=None*, *res\_extract\_suffix=None*, *log\_level=None*, *log\_file=None*, *worker\_nb=None*, *config\_file=None*, *previous\_run=None*, *build\_indexes=None*)[source]¶
:   Parse configuration files and handle the configuration according to the following file location precedence:
    /etc/macsyfinder/macsyfinder.conf < ~/.macsyfinder/macsyfinder.conf < .macsyfinder.conf

    If a configuration file is given on the command-line, this file will be used.
    *In fine* the arguments passed on the command-line have the highest priority.

    \_\_init\_\_(*cfg\_file=''*, *sequence\_db=None*, *db\_type=None*, *replicon\_topology=None*, *topology\_file=None*, *inter\_gene\_max\_space=None*, *min\_mandatory\_genes\_required=None*, *min\_genes\_required=None*, *max\_nb\_genes=None*, *multi\_loci=None*, *hmmer\_exe=None*, *index\_db\_exe=None*, *e\_value\_res=None*, *i\_evalue\_sel=None*, *coverage\_profile=None*, *def\_dir=None*, *res\_search\_dir=None*, *res\_search\_suffix=None*, *profile\_dir=None*, *profile\_suffix=None*, *res\_extract\_suffix=None*, *log\_level=None*, *log\_file=None*, *worker\_nb=None*, *config\_file=None*, *previous\_run=None*, *build\_indexes=None*)[source]¶
    :   |  |  |
        | --- | --- |
        | Parameters: | - **cfg\_file** (*string*) – the path to the MacSyFinder configuration file to use - **previous\_run** (*string*) – the path to the results directory of a previous run - **sequence\_db** (*string*) – the path to the sequence input dataset (fasta format) - **db\_type** (*string*) – the type of dataset to deal with.   “unordered\_replicon” corresponds to a non-assembled genome,   “unordered” to a metagenomic dataset,   “ordered\_replicon” to an assembled genome, and   “gembase” to a set of replicons where sequence identifiers follow this convention “>RepliconName\_SequenceID”.” - **replicon\_topology** (*string*) – the topology (‘linear’ or ‘circular’) of the replicons. This option is meaningful only if the db\_type is ‘ordered\_replicon’ or ‘gembase’ - **topology\_file** (*string*) – a tabular file of mapping between replicon names and the corresponding topology (e.g. “RepliconA linear”) - **inter\_gene\_max\_space** (*list of list of 2 elements [[ string system, integer space] , ...]*) – - **min\_mandatory\_genes\_required** (*list of list of 2 elements [[ string system, integer ] , ...]*) – - **min\_genes\_required** (*list of list of 2 elements [[ string system, integer ] , ...]*) – - **max\_nb\_genes** (*list of list of 2 elements [[ string system, integer ] , ...]*) – - **multi\_loci** (*string*) – - **hmmer\_exe** (*string*) – the Hmmer “hmmsearch” executable - **index\_db\_exe** (*string*) – the indexer executable (“makeblastdb” or “formatdb”) - **e\_value\_res** (*float*) – maximal e-value for hits to be reported during Hmmer search - **i\_evalue\_sel** (*float*) – maximal independent e-value for Hmmer hits to be selected for system detection - **coverage\_profile** (*float*) – minimal profile coverage required in the hit alignment to allow the hit selection for system detection - **def\_dir** (*string*) – the path to the directory containing systems definition files (.xml) - **res\_search\_dir** (*string*) – the path to the directory where to store MacSyFinder search results directories. - **res\_search\_suffix** (*string*) – the suffix to give to Hmmer raw output files - **res\_extract\_suffix** (*string*) – the suffix to give to filtered hits output files - **profile\_dir** (*string*) – path to the profiles directory - **profile\_suffix** (*string*) – the suffix of profile files. For each ‘Gene’ element, the corresponding profile is searched in the ‘profile\_dir’, in a file which name is based on the Gene name + the profile suffix. - **log\_level** (*int*) – the level of log output - **log\_file** (*string*) – the path to the directory to write MacSyFinder log files - **worker\_nb** (*int*) – maximal number of processes to be used in parallel (multi-thread run, 0 use all cores availables) - **build\_indexes** (*boolean*) – build the indexes from the sequence dataset in fasta format |

    \_\_weakref\_\_¶
    :   list of weak references to the object (if defined)

    \_validate(*cmde\_line\_opt*, *cmde\_line\_values*)[source]¶
    :   Get all configuration values and check the validity of their values.
        Create the working directory

        |  |  |
        | --- | --- |
        | Parameters: | - **cmde\_line\_opt** (*dict, all values are cast in string*) – the options from the command line - **cmde\_line\_values** (*dict, values are not cast*) – the options from the command line |
        | Returns: | all the options for this execution |
        | Return type: | dictionary |

    build\_indexes[source]¶
    :   |  |  |
        | --- | --- |
        | Returns: | True if the indexes must be rebuilt, False otherwise |
        | Return type: | boolean |

    coverage\_profile[source]¶
    :   |  |  |
        | --- | --- |
        | Returns: | the coverage threshold used to select a hit for systems detection and for the Hmmer report (filtered hits) |
        | Return type: | float |

    db\_type[source]¶
    :   |  |  |
        | --- | --- |
        | Returns: | the type of the input sequence dataset. The allowed values are :’unordered\_replicon’, ‘ordered\_replicon’, ‘gembase’, ‘unordered’ |
        | Return type: | string |

    def\_dir[source]¶
    :   |  |  |
        | --- | --- |
        | Returns: | the path to the directory where are stored definitions of secretion systems (.xml files) |
        | Return type: | string |

    e\_value\_res[source]¶
    :   |  |  |
        | --- | --- |
        | Returns: | The e\_value threshold used by Hmmer to report hits in the Hmmer raw output file |
        | Return type: | float |

    hmmer\_dir[source]¶
    :   |  |  |
        | --- | --- |
        | Returns: | the name of the directory where the hmmer results are stored |
        | Return type: | string |

    hmmer\_exe[source]¶
    :   |  |  |
        | --- | --- |
        | Returns: | the name of the binary to execute for homology search from HMM protein profiles (Hmmer) |
        | Return type: | string |

    i\_evalue\_sel[source]¶
    :   |  |  |
        | --- | --- |
        | Returns: | the i\_evalue threshold used to select a hit for systems detection and for the Hmmer report (filtered hits) |
        | Return type: | float |

    index\_db\_exe[source]¶
    :   |  |  |
        | --- | --- |
        | Returns: | the name of the binary to index the input sequences dataset for Hmmer |
        | Return type: | string |

    inter\_gene\_max\_space(*system*)[source]¶
    :   |  |  |
        | --- | --- |
        | Parameters: | **system** (*string*) – the name of a system |
        | Returns: | the maximum number of components with no match allowed between two genes with a match to consider them contiguous (at the system level) |
        | Return type: | integer |

    max\_nb\_genes(*system*)[source]¶
    :   |  |  |
        | --- | --- |
        | Parameters: | **system** (*string*) – the name of a system |
        | Returns: | the maximum number of genes to assess the system presence |
        | Return type: | integer |

    min\_genes\_required(*system*)[source]¶
    :   |  |  |
        | --- | --- |
        | Parameters: | **system** (*string*) – the name of a system |
        | Returns: | the genes (mandatory+accessory) quorum to assess the system presence |
        | Return type: | integer |

    min\_mandatory\_genes\_required(*system*)[source]¶
    :   |  |  |
        | --- | --- |
        | Parameters: | **system** (*string*) – the name of a system |
        | Returns: | the mandatory genes quorum to assess the system presence |
        | Return type: | integer |

    multi\_loci(*system*)[source]¶
    :   |  |  |
        | --- | --- |
        | Parameters: | **system** (*string*) – the name of a system |
        | Returns: | the genes (mandatory+accessory) quorum to assess the system presence |
        | Return type: | boolean |

    previous\_run[source]¶
    :   |  |  |
        | --- | --- |
        | Returns: | the path to the previous run directory to use (to recover Hmmer raw output) |
        | Return type: | string |

    profile\_dir[source]¶
    :   |  |  |
        | --- | --- |
        | Returns: | the path to the directory where are the HMM protein profiles which corresponds to Gene |
        | Return type: | string |

    profile\_suffix[source]¶
    :   |  |  |
        | --- | --- |
        | Returns: | the suffix for profile files |
        | Return type: | string |

    replicon\_topology[source]¶
    :   |  |  |
        | --- | --- |
        | Returns: | the topology of the replicons. Two values are supported ‘linear’ (default) and circular. Only relevant for ‘ordered’ datasets |
        | Return type: | string |

    res\_extract\_suffix[source]¶
    :   |  |  |
        | --- | --- |
        | Returns: | the suffix of extract files (tabulated files after HMM output parsing and filtering of hits) |
        | Return type: | string |

    res\_search\_dir[source]¶
    :   :return the path to the directory to store results of MacSyFinder runs
        :rtype: string

    res\_search\_suffix[source]¶
    :   |  |  |
        | --- | --- |
        | Returns: | the suffix for Hmmer raw output files |
        | Return type: | string |

    save(*dir\_path*)[source]¶
    :   save the configuration used for this run in the ini format file

    sequence\_db[source]¶
    :   |  |  |
        | --- | --- |
        | Returns: | the path to the input sequence dataset (in fasta format) |
        | Return type: | string |

    topology\_file[source]¶
    :   |  |  |
        | --- | --- |
        | Returns: | the path to the file of replicons topology. |
        | Return type: | string |

    worker\_nb[source]¶
    :   |  |  |
        | --- | --- |
        | Returns: | the maximum number of parallel jobs |
        | Return type: | int |

    working\_dir[source]¶
    :   |  |  |
        | --- | --- |
        | Returns: | the path to the working directory to use for this run |
        | Rtpe : | string |

### Table Of Contents

- Configuration API
  - Config API reference

#### Previous topic

MacSyFinder functioning

#### Next topic

Database API

### This Page

- Show Source

### Quick search


Enter search terms or a module, class or function name.

### Navigation

- index
- modules |
- next |
- previous |
- MacSyFinder documentation »

© Copyright 2014, Sophie Abby, Bertrand Néron.
Created using Sphinx 1.1.3.
