# Supplementary material for: MacSyFinder: A Program to Mine Genomes for Molecular Systems with an Application to CRISPR-Cas Systems
Source: PLoS One. 2014 Oct 17;9(10):e110726. doi: 10.1371/journal.pone.0110726 (PMC4201578; doi:10.1371/journal.pone.0110726)
Supplement: Data S1 — The MacSyFinder/MacSyView package (compressed tarball archive). (GZ) [file pone.0110726.s010.gz › macsyfinder-1.0.0-RC3/doc/_build/html/database.html]

Database API — MacSyFinder 1.0.0-RC3 documentation


### Navigation

- index
- modules |
- next |
- previous |
- MacSyFinder documentation »

# Database API¶

The “database” object handles the indexes of the sequence dataset in fasta format, and other useful information on the input dataset.

MacSyFinder needs several indexes to run, and speed up the analyses.

> - index for hmmsearch (Hmmer program)
> - index for MacSyFinder

hmmsearch needs to index the sequences to speed up the analyses. The indexes are built by the external tools
*formatdb* (ftp://ftp.ncbi.nlm.nih.gov/blast/executables/release/LATEST/ncbi.tar.gz) or *makeblastdb*. MacSyFinder tries to find formatdb indexes in the same directory as the sequence file. If the indexes are present
MacSyFinder uses these index, otherwise it builds these indexes using formatdb or makeblastdb.

MacSyFinder needs also to have the length of each sequence and its position in the database to compute some statistics on Hmmer hits. Thus it also builds an index (with .idx suffix) that is stored in the same directory as the sequence dataset. If this file is found in the same folder than the input dataset, MacSyFinder will use it. Otherwise, it will build it.

The user can force MacSyFinder to rebuild these indexes with the “–idx” option on the command-line.

Additionally, for ordered datasets ( db\_type = ‘gembase’ or ‘ordered\_replicon’ ), MacSyFinder builds an internal “database” from these indexes to store information about replicons, their begin and end positions, and their topology.
The begin and end positions of each replicon are computed from the sequence file, and the topology from the parsing of the topology file (–topology-file, see *Topology files*).

## database API reference¶

*class* macsypy.database.Indexes(*cfg*)[source]¶
:   Handle the indexes for macsyfinder:

    > - find the indexes for hmmer, or build them using formatdb or makeblastdb external tools
    > - find the indexes required by macsyfinder to compute some scores, or build them.

    \_\_init\_\_(*cfg*)[source]¶
    :   The constructor retrieves the file of indexes in the case they are not present
        or the user asked for build indexes (–idx)
        Launch the indexes building.

        |  |  |
        | --- | --- |
        | Parameters: | **cfg** (macsypy.config.Config object) – the configuration |

    \_\_weakref\_\_¶
    :   list of weak references to the object (if defined)

    \_build\_hmmer\_indexes()[source]¶
    :   build the index files for hmmer using the formatdb or makeblastdb tool

    \_build\_my\_indexes()[source]¶
    :   Build macsyfinder indexes. These indexes are stored in a file.

        The file format is the following:
        :   - one entry per line, with each line having this format:
            - sequence id;sequence length;sequence rank

    build(*force=False*)[source]¶
    :   Build the indexes from the sequence dataset in fasta format

        |  |  |
        | --- | --- |
        | Parameters: | **force** (*boolean*) – If True, force the index building even if the index files are present in the sequence dataset folder |

    find\_hmmer\_indexes()[source]¶
    :   |  |  |
        | --- | --- |
        | Returns: | The hmmer index files. If indexes are inconsistent (some file(s) missing), a Runtime Error is raised |
        | Return type: | list of string |

    find\_my\_indexes()[source]¶
    :   |  |  |
        | --- | --- |
        | Returns: | the file of macsyfinder indexes if it exists in the dataset folder, None otherwise. |
        | Return type: | string |

*class* macsypy.database.RepliconDB(*cfg*)[source]¶
:   Stores information (topology, min, max, [genes]) for all replicons in the sequence\_db
    the Replicon object must be instantiated only for sequence\_db of type ‘gembase’ or ‘ordered\_replicon’

    \_\_contains\_\_(*replicon\_name*)[source]¶
    :   |  |  |
        | --- | --- |
        | Parameters: | **replicon\_name** (*string*) – the name of the replicon |
        | Returns: | True if replicon\_name is in the repliconDB, false otherwise. |
        | Return type: | boolean |

    \_\_getitem\_\_(*replicon\_name*)[source]¶
    :   |  |  |
        | --- | --- |
        | Parameters: | **replicon\_name** (*string*) – the name of the replicon to get information on |
        | Returns: | the RepliconInfo for the provided replicon\_name |
        | Return type: | RepliconInfo object |
        | Raise : | KeyError if replicon\_name is not in repliconDB |

    \_\_init\_\_(*cfg*)[source]¶
    :   |  |  |
        | --- | --- |
        | Parameters: | **cfg** (macsypy.config.Config object) – The configuration object |

        Note

        This class can be instanciated only if the db\_type is ‘gembase’ or ‘ordered\_replicon’

    \_\_weakref\_\_¶
    :   list of weak references to the object (if defined)

    \_fill\_gembase\_min\_max(*topology*, *default\_topology*)[source]¶
    :   For each replicon\_name of a gembase dataset, it fills the internal dictionary with a namedtuple RepliconInfo

        |  |  |
        | --- | --- |
        | Parameters: | - **topology** (*dict*) – the topologies for each replicon   (parsed from the file specified with the option –topology-file) - **default\_topology** (*string*) – the topology provided by the config.replicon\_topology |

    \_fill\_ordered\_min\_max(*default\_topology=None*)[source]¶
    :   For the replicon\_name of the ordered\_replicon sequence base, fill the internal dict with RepliconInfo

        |  |  |
        | --- | --- |
        | Parameters: | **default\_topology** (*string*) – the topology provided by config.replicon\_topology |

    \_fill\_topology()[source]¶
    :   Fill the internal dictionary with min and max positions for each replicon\_name of the sequence\_db

    get(*replicon\_name*, *default=None*)[source]¶
    :   |  |  |
        | --- | --- |
        | Parameters: | - **replicon\_name** (*string*) – the name of the replicon to get informations - **default** (*any*) – the value to return if the replicon\_name is not in the RepliconDB |
        | Returns: | the RepliconInfo for replicon\_name if replicon\_name is in the repliconDB, else default. If default is not given, it is set to None, so that this method never raises a KeyError. |
        | Return type: | RepliconInfo object |

    items()[source]¶
    :   |  |  |
        | --- | --- |
        | Returns: | a copy of the RepliconDB as a list of (replicon\_name, RepliconInfo) pairs |

    iteritems()[source]¶
    :   |  |  |
        | --- | --- |
        | Returns: | an iterator over the RepliconDB as a list (replicon\_name, RepliconInfo) pairs |

    replicon\_infos()[source]¶
    :   |  |  |
        | --- | --- |
        | Returns: | a copy of the RepliconDB as list of replicons info |
        | Return type: | RepliconInfo instance |

    replicon\_names()[source]¶
    :   |  |  |
        | --- | --- |
        | Returns: | a copy of the RepliconDB as a list of replicon\_names |

*class* macsypy.database.RepliconInfo¶
:   RepliconInfo(topology, min, max, genes)

    \_\_getnewargs\_\_()¶
    :   Return self as a plain tuple. Used by copy and pickle.

    *static* \_\_new\_\_(*\_cls*, *topology*, *min*, *max*, *genes*)¶
    :   Create new instance of RepliconInfo(topology, min, max, genes)

    \_\_repr\_\_()¶
    :   Return a nicely formatted representation string

    \_asdict()¶
    :   Return a new OrderedDict which maps field names to their values

    *classmethod* \_make(*iterable*, *new=<built-in method \_\_new\_\_ of type object at 0x10cacd840>*, *len=<built-in function len>*)¶
    :   Make a new RepliconInfo object from a sequence or iterable

    \_replace(*\_self*, *\*\*kwds*)¶
    :   Return a new RepliconInfo object replacing specified fields with new values

    genes¶
    :   Alias for field number 3

    max¶
    :   Alias for field number 2

    min¶
    :   Alias for field number 1

    topology¶
    :   Alias for field number 0

macsypy.database.fasta\_iter(*fasta\_file*)[source]¶
:   |  |  |
    | --- | --- |
    | Parameters: | **fasta\_file** (*file object*) – the file containing all input sequences in fasta format. |
    | Author : | http://biostar.stackexchange.com/users/36/brentp |
    | Returns: | for a given fasta file, it returns an iterator which yields tuples (string id, string comment, int sequence length) |
    | Return type: | iterator |

### Table Of Contents

- Database API
  - database API reference

#### Previous topic

Configuration API

#### Next topic

System API

### This Page

- Show Source

### Quick search


Enter search terms or a module, class or function name.

### Navigation

- index
- modules |
- next |
- previous |
- MacSyFinder documentation »

© Copyright 2014, Sophie Abby, Bertrand Néron.
Created using Sphinx 1.1.3.
