# Supplementary material for: MacSyFinder: A Program to Mine Genomes for Molecular Systems with an Application to CRISPR-Cas Systems
Source: PLoS One. 2014 Oct 17;9(10):e110726. doi: 10.1371/journal.pone.0110726 (PMC4201578; doi:10.1371/journal.pone.0110726)
Supplement: Data S1 — The MacSyFinder/MacSyView package (compressed tarball archive). (GZ) [file pone.0110726.s010.gz › macsyfinder-1.0.0-RC3/doc/_build/html/functioning.html]

MacSyFinder functioning — MacSyFinder 1.0.0-RC3 documentation


### Navigation

- index
- modules |
- next |
- previous |
- MacSyFinder documentation »

# MacSyFinder functioning¶

## Functioning overview¶

## A. Searching for systems’ components¶

MacSyFinder is run from the command-line using a variety of input files and options. See *Input dataset* for more details.

Initially, MacSyFinder **searches for the components** of a system by sequence similarity search.

From the list of systems to detect, a non-redundant list of components to search is built. For each system, the list includes:

> - mandatory components
> - accessory components
> - forbidden components
> - homologs and/or analogs of these three types of components in the case they are *“exchangeable”*

Hmmer is run on the corresponding set of HMM profiles, and the hits are filtered according to criteria defined by the user (see *Hmmer options* and *HMMReport API*). This step, and the extraction of significant hits can be performed in parallel (-w command-line option). See the *Command-line options*, and the *search\_genes API* for more details.

## B. Assessing systems presence¶

The following steps depend on whether the input dataset is *ordered* (complete or nearly complete genome(s)), or *unordered* (metagenomes, or unassembled genome) (see *Input dataset*).
In the case of **ordered datasets**, the hits of the previous analysis are used to build *clusters of co-localized genes* as defined in the XML files. These clusters are then scanned to check for the model specifications like minimal quorum of “Mandatory” or “Accessory” genes or the absence of “Forbidden” components.
When the gene order is unknown the power of the analysis is more limited. In this case, and depending on the type of dataset, the presence of systems can be suggested only on the basis of the quorum of genes. The results are outputted in a tabular and graphical form (see *Output format*).

### For *ordered* datasets:¶

1. The search starts first with the formation of clusters of contiguous hits **(co-localization criterion)** for each replicon. Two hits are said contiguous if their genomic location is separated by less than D protein-encoding gene, D being the maximum of the parameter “inter\_gene\_max\_space” from the two genes with hits (system-specific, of gene-specific parameter).
2. Clusters are then scanned, and those containing only genes from a single system are kept for further analyses (step 4.), wether those with multiple systems represented are analysed with a disambiguation step (step 3.).
3. The disambiguation step consists in splitting clusters that contains genes from different systems into sub-clusters that contain genes from a single system. Valid sub-clusters are then analysed like other clusters (step 2.). In the complex cases where genes from a same system are scattered into the cluster, then corresponding sub-clusters will not be further analysed for system detection.
4. Valid clusters are used to fill system occurrences (macsypy.search\_systems.SystemOccurence). In this step, the **quorum** criteria for the system assessment are checked according to the system’s definition. In the case a single locus fills a complete system occurrence, it is stored and reported in the output files (**“single-locus”** occurrence). Otherwise, if the cluster corresponds to a valid but incomplete system, it is stored for inclusion in a scattered system occurrence (**“multi-loci”** occurrence).
5. When all clusters, “loner” genes and “multi\_system” genes were scanned for inclusion in system occurrences, a decision is made for every system occurrence regarding the **quorum rules** defined for the corresponding system.

Note

When the “multi\_loci” option is turned on, a single “multi-loci” system is assessed per replicon, even if it could correspond to multiple scattered systems. Thus, the “single-locus” systems correspond to a more powerful mode of detection.

Warning

Cases where systems are consecutive will be treated, and separate systems will be detected, but complex cases of detection, *i.e.* when systems’ components are intermingled will not be considered.

### For *unordered* datasets:¶

1. The Hits are grouped by system.
2. They are used to fill a single system occurrence (macsypy.search\_systems.SystemOccurence) per system type.

Note

The “unordered” mode of detection is less powerful, as a single occurrence of a given system is filled for an entire dataset with hits that origin is unknown. Please consider “systems assessments” with caution in this mode.

### Table Of Contents

- MacSyFinder functioning
  - Functioning overview
  - A. Searching for systems’ components
  - B. Assessing systems presence
    - For *ordered* datasets:
    - For *unordered* datasets:

#### Previous topic

MacSyFinder implementation overview

#### Next topic

Configuration API

### This Page

- Show Source

### Quick search


Enter search terms or a module, class or function name.

### Navigation

- index
- modules |
- next |
- previous |
- MacSyFinder documentation »

© Copyright 2014, Sophie Abby, Bertrand Néron.
Created using Sphinx 1.1.3.
