# Supplementary material for: MacSyFinder: A Program to Mine Genomes for Molecular Systems with an Application to CRISPR-Cas Systems
Source: PLoS One. 2014 Oct 17;9(10):e110726. doi: 10.1371/journal.pone.0110726 (PMC4201578; doi:10.1371/journal.pone.0110726)
Supplement: Data S1 — The MacSyFinder/MacSyView package (compressed tarball archive). (GZ) [file pone.0110726.s010.gz › macsyfinder-1.0.0-RC3/doc/_build/html/gembase_convention.html]

Gembase format — MacSyFinder 1.0.0-RC3 documentation


### Navigation

- index
- modules |
- next |
- previous |
- MacSyFinder documentation »
- Input and Options of MacSyFinder »

# Gembase format¶

In order to allow the users running MacSyFinder on a bunch of genomes in a single run, we propose to adopt the following convention to fulfill the requirements for the “gembase db\_type”. It consists in providing for each protein, both the replicon name and a protein identifier separated by a “\_” in the first field of fasta headers.
For instance:

```
>PlasmidA_0001 YP_003225072.1_ | putative stcE protein
MKLKYLSCMILASLAMGAFAATAADNNSAIYFNTTQPVNDLQGGLAAEVK
FAQSQILSAHPKEGESQQHLTSLRKSLLLVRLVKADDKTPVQVEARDAND
KILGTLTLSPPSSLPDTVYHLDGVPADGIDFTPQNGTKKIINTVAEVNKL
SDASGSSIKSYLANNALVEIQTANGRWIRDMYLPQGAELEGKMVRFVSYA
GYNSTVFYGDRKVTLSVGNTLLFKYVNGQWFRSGELENNRIAYAQHTWSA
ELPAHWIVPGLNLVIKQGNLSGSLNDINVGAPGELLLHTIDIGMLTTPRG
RFDFAKDKEAHREYFQTIPVSRMIVNNYAPLHLKEVMLPTGTLLTDADPG
>PlasmidA_0002 YP_003225073.1_ | type II secretion protein EtpC
MLFFLSSRRDRNLFIKDIALKMLTPNWVLCVILLIAGYQLVSVIRHFWLT
PATSASDLSHVSVSETAVTDEHTEENFVFTLFGTASPPLSEGKVQKTTSS
LSDDLLSGGDLDVRGILYSSVTEHSVAIFAHNNRQFSLGIGEKVPGYDAT
ISAIFSDHIVINYQGKNASLPLRYDNPAKRNAQDDNNLIVGPVTTQANFR
VKNIFDIMSLSPVTVNNTLSGYRLSPGKASSLFYNAGLHDNDLAVLLNGS
ELRDTRQAKQIMKQLTELKEIKITVERDGQLYDAFIAVGEN
....
>ChromosomeA_0001 _YP_003573410.1_ | adhesin-like protein
MKKLFLFAALLMTGFAFYSCEDVVDNPAQDPAQSWNYSVSVKFADFDFNG
AVDENSVPYTYKAPTTLYVLNEENTLMGTITTDAAPAIGDYGTYAGTLTG
SIGNNLIITTKIGNDLTKQDGTLKSAIENGIVQTAEVPIKIYNANSGTLT
TASAKMDNTAAIAYTSLGYIKGGDKILFVEGNQTFEWTVNEEFDPYTSTD
LYIALPMNTDPETEYTISSDSKDGYTRGGTFKLADYPTLAAGKVSNYIGG
IPFIQTGVDLTKWDAYMRTDPNNTWYMNNINNGWPATFSQEVEDGKSFIV
TQSGPTLDSLNVVVGGVTGKEVNVTLNNIRLGKDRSINIGDKHGWVEYDG
THDIYGWGAKANVTLIGENECETLYIQCPATKKGEGTLNYKNLSIDSYGS
>ChromosomeA_0020 _YP_003573411.1_ | hypothetical protein
MKRIVLITLVSILTTFQAIAQVANGFYRVQNNASSRYITLRDNAVGTVDY
SSTNVDLSNIVTWSGFDKVKSNPASIIYVEQHDSKYDLKVQGTGIYAITG
GRTYLELRPKDSGYILAVTYNGMEGRLYDSEEDVDGEGYVKRSGNSAYQY
WSFIPVDTENNYIGLQPTVQVGDNYYGTLYASYPFKAASSGIKFYYVDAI
....
```

# Topology files¶

To be able to attribute a topology per replicon/genome when using the Gembase format, we propose the user to build a “topology file” in the form of a tabular file with two columns separated by a ”:”. The 1st column is the replicon name, and the 2nd the corresponding topology. Comments can be written after a “#”.

For example:

```
# comment line
PlasmidA : circular
ChromosomeA : linear
ChromosomeB : circular
```

Note

A topology file can be specified on the command-line with the “--topology-file” parameter.

### Table Of Contents

- Gembase format
- Topology files

#### Previous topic

Input and Options of MacSyFinder

#### Next topic

Output format

### This Page

- Show Source

### Quick search


Enter search terms or a module, class or function name.

### Navigation

- index
- modules |
- next |
- previous |
- MacSyFinder documentation »
- Input and Options of MacSyFinder »

© Copyright 2014, Sophie Abby, Bertrand Néron.
Created using Sphinx 1.1.3.
