# Supplementary material for: MacSyFinder: A Program to Mine Genomes for Molecular Systems with an Application to CRISPR-Cas Systems
Source: PLoS One. 2014 Oct 17;9(10):e110726. doi: 10.1371/journal.pone.0110726 (PMC4201578; doi:10.1371/journal.pone.0110726)
Supplement: Data S1 — The MacSyFinder/MacSyView package (compressed tarball archive). (GZ) [file pone.0110726.s010.gz › macsyfinder-1.0.0-RC3/doc/_build/html/gene.html]

Gene API — MacSyFinder 1.0.0-RC3 documentation


### Navigation

- index
- modules |
- next |
- previous |
- MacSyFinder documentation »

# Gene API¶

The Gene object represents genes encoding the protein components of a System. See *The Gene object* for an overview of the implementation.

Warning

To optimize computation and to avoid concurrency problems when we search several systems, each gene must be instanciated only once, and stored in gene\_bank.
gene\_bank is a macsypy.gene.GeneBank object.
The gene\_bank and system\_bank (macsypy.system.SystemBank object) are filled by a system\_parser (macsypy.system\_parser.SystemParser)

Example to get a gene object:

```
from macsypy.system import system_bank
from macsypy.config import Config
from macsypy.gene import gene_bank


#get a system
t2ss =  system_bank["T2SS"]

#get of a gene
pilO = gene_bank["pilO"]
```

## GeneBank API reference¶

> *class* macsypy.gene.GeneBank[source]¶
> :   Store all Gene objects. Ensure that genes are instanciated only once.
>
>     \_\_contains\_\_(*gene*)[source]¶
>     :   Implement the membership test operator
>
>         |  |  |
>         | --- | --- |
>         | Parameters: | **gene** (macsypy.gene.Gene object) – the gene to test |
>         | Returns: | True if the gene is in, False otherwise |
>         | Return type: | boolean |
>
>     \_\_getitem\_\_(*name*)[source]¶
>     :   |  |  |
>         | --- | --- |
>         | Parameters: | - **name** (*string*) – the name of the Gene - **cfg** (macsypy.config.Config object) – the configuration object |
>         | Returns: | return the Gene corresponding to the name. If the Gene already exists, return it, otherwise, build it and return it |
>         | Return type: | macsypy.gene.Gene object |
>
>     \_\_iter\_\_()[source]¶
>     :   Return an iterator object on the genes contained in the bank
>
>     \_\_weakref\_\_¶
>     :   list of weak references to the object (if defined)
>
>     add\_gene(*gene*)[source]¶
>     :   |  |  |
>         | --- | --- |
>         | Parameters: | - **name** (*string*) – the name of the gene - **cfg** (macsypy.config.Config object) – the configuration |
>         | Returns: | return gene corresponding to the name. If the gene already exists, return it, otherwise, build it and return it |
>         | Return type: | macsypy.gene.Gene object |
>         | Raise : | KeyError if a gene with the same name is already registered |

Note

Don’t instanciate your own GeneFactory use the gene\_bank at the top level of the module.

```
from macsypy.gene import gene_bank
```

## Gene API reference¶

*class* macsypy.gene.Gene(*cfg*, *name*, *system*, *profiles\_registry*, *loner=False*, *exchangeable=False*, *multi\_system=False*, *inter\_gene\_max\_space=None*)[source]¶
:   Handle Gene of a (secretion) System

    \_\_eq\_\_(*gene*)[source]¶
    :   |  |  |
        | --- | --- |
        | Returns: | True if the gene names (gene.name) are the same, False otherwise. |
        | Parameters: | **gene** (macsypy.gene.Gene object.) – the query of the test |
        | Return type: | boolean. |

    \_\_init\_\_(*cfg*, *name*, *system*, *profiles\_registry*, *loner=False*, *exchangeable=False*, *multi\_system=False*, *inter\_gene\_max\_space=None*)[source]¶
    :   handle gene

        |  |  |
        | --- | --- |
        | Parameters: | - **cfg** (macsypy.config.Config object) – the configuration object. - **name** (*string.*) – the name of the Gene. - **system** (macsypy.system.System object.) – the system that owns this Gene - **profiles\_registry** (macsypy.registries.ProfilesRegistry object.) – where all the paths profiles where register. - **loner** (*boolean.*) – True if the Gene can be isolated on the genome (with no contiguous genes), False otherwise. - **exchangeable** (*boolean.*) – True if this Gene can be replaced with one of its homologs or analogs whithout any effects on the system assessment, False otherwise. - **multi\_system** (*boolean.*) – True if this Gene can belong to different occurrences of this System. - **inter\_gene\_max\_space** (*integer*) – the maximum space between this Gene and another gene of the System. |

    \_\_str\_\_()[source]¶
    :   Print the name of the gene and of its homologs/analogs.

    \_\_weakref\_\_¶
    :   list of weak references to the object (if defined)

    add\_analog(*analog*)[source]¶
    :   Add an analogous gene to the Gene

        |  |  |
        | --- | --- |
        | Parameters: | **analog** (macsypy.gene.Analog object) – analog to add |

    add\_homolog(*homolog*)[source]¶
    :   Add a homolog gene to the Gene

        |  |  |
        | --- | --- |
        | Parameters: | **homolog** (macsypy.gene.Homolog object) – homolog to add |

    exchangeable[source]¶
    :   |  |  |
        | --- | --- |
        | Returns: | True if this gene can be replaced with one of its homologs or analogs whithout any effects on the system, False otherwise. |
        | Return type: | boolean. |

    get\_analogs()[source]¶
    :   |  |  |
        | --- | --- |
        | Returns: | the Gene analogs |
        | Type : | list of macsypy.gene.Analog object |

    get\_compatible\_systems(*system\_list*, *include\_forbidden=True*)[source]¶
    :   Test every system in system\_list for compatibility with the gene using the is\_authorized function.

        |  |  |
        | --- | --- |
        | Parameters: | - **system\_list** (*list of strings*) – a list of system names to test - **include\_forbidden** (*boolean*) – tells if forbidden genes should be considered as defining a compatible systems or not |
        | Returns: | the list of compatible systems |
        | Return type: | list of string, or void list if none compatible |

    get\_homologs()[source]¶
    :   |  |  |
        | --- | --- |
        | Returns: | the Gene homologs |
        | Type : | list of macsypy.gene.Homolog object |

    inter\_gene\_max\_space[source]¶
    :   |  |  |
        | --- | --- |
        | Returns: | The maximum distance allowed between this gene and another gene for them to be considered co-localized. If the value is not set at the Gene level, return the value set at the System level. |
        | Return type: | integer. |

    is\_accessory(*system*)[source]¶
    :   |  |  |
        | --- | --- |
        | Returns: | True if the gene is within the *accessory* genes of the system, False otherwise. |
        | Parameters: | **system** (macsypy.system.System object.) – the query of the test |
        | Return type: | boolean. |

    is\_analog(*gene*)[source]¶
    :   |  |  |
        | --- | --- |
        | Returns: | True if the two genes are analogs, False otherwise. |
        | Parameters: | **gene** (macsypy.gene.Gene object.) – the query of the test |
        | Return type: | boolean. |

    is\_authorized(*system*, *include\_forbidden=True*)[source]¶
    :   |  |  |
        | --- | --- |
        | Returns: | True if the genes are found in the System definition file (.xml), False otherwise. |
        | Parameters: | - **system** (macsypy.system.System object.) – the query of the test - **include\_forbidden** (*boolean*) – tells if forbidden genes should be considered as “authorized” or not |
        | Return type: | boolean. |

    is\_forbidden(*system*)[source]¶
    :   |  |  |
        | --- | --- |
        | Returns: | True if the gene is within the *forbidden* genes of the system, False otherwise. |
        | Parameters: | **system** (macsypy.system.System object.) – the query of the test |
        | Return type: | boolean. |

    is\_homolog(*gene*)[source]¶
    :   |  |  |
        | --- | --- |
        | Returns: | True if the two genes are homologs, False otherwise. |
        | Parameters: | **gene** (macsypy.gene.Gene object.) – the query of the test |
        | Return type: | boolean. |

    is\_mandatory(*system*)[source]¶
    :   |  |  |
        | --- | --- |
        | Returns: | True if the gene is within the *mandatory* genes of the system, False otherwise. |
        | Parameters: | **system** (macsypy.system.System object.) – the query of the test |
        | Return type: | boolean. |

    loner[source]¶
    :   |  |  |
        | --- | --- |
        | Returns: | True if the gene can be isolated on the genome, False otherwise |
        | Return type: | boolean |

    multi\_system[source]¶
    :   |  |  |
        | --- | --- |
        | Returns: | True if this Gene can belong to different occurrences of **this System** (and can be used for multiple System assessments), False otherwise. |
        | Return type: | boolean. |

    profile *= None*¶
    :   |  |  |
        | --- | --- |
        | Variables: | **profile** – The HMM protein Profile corresponding to this gene macsypy.gene.Profile object |

    system[source]¶
    :   |  |  |
        | --- | --- |
        | Returns: | the System that owns this Gene |
        | Return type: | macsypy.system.System object |

Note

All attributes/methods which are not directly implemented in Homolog are redirected to that of the encapsulated Gene.

## Homolog API reference¶

*class* macsypy.gene.Homolog(*gene*, *gene\_ref*, *aligned=False*)[source]¶
:   Handle homologs, encapsulate a Gene

    \_\_init\_\_(*gene*, *gene\_ref*, *aligned=False*)[source]¶
    :   |  |  |
        | --- | --- |
        | Parameters: | - **gene** (macsypy.gene.Gene object.) – the gene - **gene\_ref** (macsypy.gene.Gene object.) – the gene to which the current is homolog. - **aligned** (*boolean*) – if True, the profile of this gene overlaps totally the sequence of the reference gene profile.   Otherwise, only partial overlapping between the profiles. |

    \_\_weakref\_\_¶
    :   list of weak references to the object (if defined)

    gene *= None*¶
    :   |  |  |
        | --- | --- |
        | Variables: | **gene** – gene |

    gene\_ref[source]¶
    :   |  |  |
        | --- | --- |
        | Returns: | the gene to which this one is homolog to (reference gene) |
        | Return type: | macsypy.gene.Gene object |

    is\_aligned()[source]¶
    :   |  |  |
        | --- | --- |
        | Returns: | True if this gene homolog is aligned to its homolog, False otherwise. |
        | Return type: | boolean |

## Analog API reference¶

*class* macsypy.gene.Analog(*gene*, *gene\_ref*)[source]¶
:   Handle analogs, encapsulate a Gene

    \_\_init\_\_(*gene*, *gene\_ref*)[source]¶
    :   |  |  |
        | --- | --- |
        | Parameters: | - **gene** (macsypy.gene.Gene object.) – the gene - **gene\_ref** (macsypy.gene.Gene object.) – the gene to which the current is analog. |

    \_\_weakref\_\_¶
    :   list of weak references to the object (if defined)

    gene *= None*¶
    :   |  |  |
        | --- | --- |
        | Variables: | **gene** – gene |

    gene\_ref[source]¶
    :   |  |  |
        | --- | --- |
        | Returns: | the gene to which this one is analog to (reference gene) |
        | Return type: | macsypy.gene.Gene object |

### Table Of Contents

- Gene API
  - GeneBank API reference
  - Gene API reference
  - Homolog API reference
  - Analog API reference

#### Previous topic

The Parser of Systems definitions

#### Next topic

Profile API

### This Page

- Show Source

### Quick search


Enter search terms or a module, class or function name.

### Navigation

- index
- modules |
- next |
- previous |
- MacSyFinder documentation »

© Copyright 2014, Sophie Abby, Bertrand Néron.
Created using Sphinx 1.1.3.
