# Supplementary material for: MacSyFinder: A Program to Mine Genomes for Molecular Systems with an Application to CRISPR-Cas Systems
Source: PLoS One. 2014 Oct 17;9(10):e110726. doi: 10.1371/journal.pone.0110726 (PMC4201578; doi:10.1371/journal.pone.0110726)
Supplement: Data S1 — The MacSyFinder/MacSyView package (compressed tarball archive). (GZ) [file pone.0110726.s010.gz › macsyfinder-1.0.0-RC3/doc/_build/html/genindex.html]

Index — MacSyFinder 1.0.0-RC3 documentation


### Navigation

- index
- modules |
- MacSyFinder documentation »

# Index

**\_**
| **A**
| **B**
| **C**
| **D**
| **E**
| **F**
| **G**
| **H**
| **I**
| **J**
| **L**
| **M**
| **N**
| **O**
| **P**
| **R**
| **S**
| **T**
| **V**
| **W**

## \_

|  |  |
| --- | --- |
| \_\_cmp\_\_() (macsypy.report.Hit method)  \_\_contains\_\_() (macsypy.database.RepliconDB method)  (macsypy.gene.GeneBank method)  (macsypy.system.SystemBank method)  \_\_eq\_\_() (macsypy.gene.Gene method)  (macsypy.report.Hit method)  \_\_getitem\_\_() (macsypy.database.RepliconDB method)  (macsypy.gene.GeneBank method)  (macsypy.system.SystemBank method)  \_\_getnewargs\_\_() (macsypy.database.RepliconInfo method)  \_\_init\_\_() (macsypy.config.Config method)  (macsypy.database.Indexes method)  (macsypy.database.RepliconDB method)  (macsypy.gene.Analog method)  (macsypy.gene.Gene method)  (macsypy.gene.Homolog method)  (macsypy.gene.Profile method)  (macsypy.report.HMMReport method)  (macsypy.report.Hit method)  (macsypy.search\_systems.Cluster method)  (macsypy.search\_systems.ClustersHandler method)  (macsypy.search\_systems.SystemOccurence method)  (macsypy.search\_systems.systemDetectionReportOrdered method)  (macsypy.search\_systems.systemDetectionReportUnordered method)  (macsypy.search\_systems.validSystemHit method)  (macsypy.system.System method)  (macsypy.system\_parser.SystemParser method)  \_\_iter\_\_() (macsypy.gene.GeneBank method)  (macsypy.system.SystemBank method)  \_\_len\_\_() (macsypy.gene.Profile method)  (macsypy.search\_systems.Cluster method)  (macsypy.system.SystemBank method)  \_\_metaclass\_\_ (macsypy.report.HMMReport attribute)  \_\_new\_\_() (macsypy.database.RepliconInfo static method)  \_\_repr\_\_() (macsypy.database.RepliconInfo method)  \_\_str\_\_() (macsypy.gene.Gene method)  (macsypy.gene.Profile method)  (macsypy.report.HMMReport method)  (macsypy.report.Hit method)  (macsypy.search\_systems.Cluster method)  (macsypy.search\_systems.SystemOccurence method)  \_\_weakref\_\_ (macsypy.config.Config attribute)  (macsypy.database.Indexes attribute)  (macsypy.database.RepliconDB attribute)  (macsypy.gene.Analog attribute)  (macsypy.gene.Gene attribute)  (macsypy.gene.GeneBank attribute)  (macsypy.gene.Homolog attribute)  (macsypy.gene.Profile attribute)  (macsypy.macsypy\_error.MacsypyError attribute)  (macsypy.report.HMMReport attribute)  (macsypy.report.Hit attribute)  (macsypy.search\_systems.Cluster attribute)  (macsypy.search\_systems.ClustersHandler attribute)  (macsypy.search\_systems.SystemNameGenerator attribute)  (macsypy.search\_systems.SystemOccurence attribute)  (macsypy.search\_systems.validSystemHit attribute)  (macsypy.system.System attribute)  (macsypy.system.SystemBank attribute)  (macsypy.system\_parser.SystemParser attribute)  \_asdict() (macsypy.database.RepliconInfo method)  \_build\_hmmer\_indexes() (macsypy.database.Indexes method)  \_build\_my\_db() (macsypy.report.HMMReport method)  \_build\_my\_indexes() (macsypy.database.Indexes method)  \_computeBasename() (macsypy.search\_systems.SystemNameGenerator method) | \_create\_genes() (macsypy.system\_parser.SystemParser method)  \_create\_system() (macsypy.system\_parser.SystemParser method)  \_fill() (macsypy.system\_parser.SystemParser method)  \_fill\_gembase\_min\_max() (macsypy.database.RepliconDB method)  \_fill\_my\_db() (macsypy.report.HMMReport method)  \_fill\_ordered\_min\_max() (macsypy.database.RepliconDB method)  \_fill\_topology() (macsypy.database.RepliconDB method)  \_hit\_start() (macsypy.report.HMMReport method)  \_len() (macsypy.gene.Profile method)  \_make() (macsypy.database.RepliconInfo class method)  \_match2json() (macsypy.search\_systems.systemDetectionReportOrdered method)  \_parse\_analog() (macsypy.system\_parser.SystemParser method)  \_parse\_hmm\_body() (macsypy.report.HMMReport method)  \_parse\_hmm\_header() (macsypy.report.HMMReport method)  \_parse\_homolog() (macsypy.system\_parser.SystemParser method)  \_replace() (macsypy.database.RepliconInfo method)  \_validate() (macsypy.config.Config method) |

## A

|  |  |
| --- | --- |
| accessory\_genes (macsypy.system.System attribute)  add() (macsypy.search\_systems.Cluster method)  add\_accessory\_gene() (macsypy.system.System method)  add\_analog() (macsypy.gene.Gene method)  add\_forbidden\_gene() (macsypy.system.System method)  add\_gene() (macsypy.gene.GeneBank method) | add\_homolog() (macsypy.gene.Gene method)  add\_mandatory\_gene() (macsypy.system.System method)  add\_system() (macsypy.system.SystemBank method)  Analog (class in macsypy.gene)  analyze\_clusters\_replicon() (in module macsypy.search\_systems) |

## B

|  |  |
| --- | --- |
| best\_hit() (macsypy.report.HMMReport method)  build() (macsypy.database.Indexes method) | build\_clusters() (in module macsypy.search\_systems)  build\_indexes (macsypy.config.Config attribute) |

## C

|  |  |
| --- | --- |
| check\_consistency() (macsypy.system\_parser.SystemParser method)  circularize() (macsypy.search\_systems.ClustersHandler method)  Cluster (class in macsypy.search\_systems)  ClustersHandler (class in macsypy.search\_systems)  compatible\_systems (macsypy.search\_systems.Cluster attribute)  compute\_missing\_genes\_list() (macsypy.search\_systems.SystemOccurence method)  compute\_system\_length() (macsypy.search\_systems.SystemOccurence method) | Config (class in macsypy.config)  count\_genes() (macsypy.search\_systems.SystemOccurence method)  count\_genes\_tot() (macsypy.search\_systems.SystemOccurence method)  count\_missing\_genes() (macsypy.search\_systems.SystemOccurence method)  counter\_output() (macsypy.search\_systems.systemDetectionReportOrdered method)  coverage\_profile (macsypy.config.Config attribute) |

## D

|  |  |
| --- | --- |
| db\_type (macsypy.config.Config attribute)  decision\_rule() (macsypy.search\_systems.SystemOccurence method) | def\_dir (macsypy.config.Config attribute)  disambiguate\_cluster() (in module macsypy.search\_systems) |

## E

|  |  |
| --- | --- |
| e\_value\_res (macsypy.config.Config attribute)  exchangeable (macsypy.gene.Gene attribute) | execute() (macsypy.gene.Profile method)  extract() (macsypy.report.GembaseHMMReport method)  (macsypy.report.GeneralHMMReport method)  (macsypy.report.HMMReport method)  (macsypy.report.OrderedHMMReport method) |

## F

|  |  |
| --- | --- |
| fasta\_iter() (in module macsypy.database)  fill\_with\_cluster() (macsypy.search\_systems.SystemOccurence method)  fill\_with\_hits() (macsypy.search\_systems.SystemOccurence method)  fill\_with\_multi\_systems\_genes() (macsypy.search\_systems.SystemOccurence method) | find\_hmmer\_indexes() (macsypy.database.Indexes method)  find\_my\_indexes() (macsypy.database.Indexes method)  forbidden\_genes (macsypy.system.System attribute) |

## G

|  |  |
| --- | --- |
| GembaseHMMReport (class in macsypy.report)  Gene (class in macsypy.gene)  gene (macsypy.gene.Analog attribute)  (macsypy.gene.Homolog attribute)  gene\_ref (macsypy.gene.Analog attribute)  (macsypy.gene.Homolog attribute)  GeneBank (class in macsypy.gene)  GeneralHMMReport (class in macsypy.report)  genes (macsypy.database.RepliconInfo attribute)  get() (macsypy.database.RepliconDB method)  get\_analogs() (macsypy.gene.Gene method)  get\_best\_hits() (in module macsypy.search\_systems)  get\_compatible\_systems() (in module macsypy.search\_systems)  (macsypy.gene.Gene method)  get\_gene() (macsypy.system.System method) | get\_gene\_counter\_output() (macsypy.search\_systems.SystemOccurence method)  get\_gene\_ref() (macsypy.search\_systems.SystemOccurence method)  (macsypy.system.System method)  get\_homologs() (macsypy.gene.Gene method)  get\_position() (macsypy.report.Hit method)  get\_profile() (macsypy.gene.ProfileFactory method)  get\_summary() (macsypy.search\_systems.SystemOccurence method)  get\_summary\_header() (macsypy.search\_systems.SystemOccurence method)  get\_summary\_unordered() (macsypy.search\_systems.SystemOccurence method)  get\_syst\_inter\_gene\_max\_space() (macsypy.report.Hit method)  get\_system\_name\_unordered() (macsypy.search\_systems.SystemOccurence method)  get\_system\_unique\_name() (macsypy.search\_systems.SystemOccurence method)  getSystemName() (macsypy.search\_systems.SystemNameGenerator method) |

## H

|  |  |
| --- | --- |
| Hit (class in macsypy.report)  hmmer\_dir (macsypy.config.Config attribute)  hmmer\_exe (macsypy.config.Config attribute) | HMMReport (class in macsypy.report)  Homolog (class in macsypy.gene) |

## I

|  |  |
| --- | --- |
| i\_evalue\_sel (macsypy.config.Config attribute)  index\_db\_exe (macsypy.config.Config attribute)  Indexes (class in macsypy.database)  inter\_gene\_max\_space (macsypy.gene.Gene attribute)  (macsypy.system.System attribute)  inter\_gene\_max\_space() (macsypy.config.Config method)  is\_accessory() (macsypy.gene.Gene method)  is\_aligned() (macsypy.gene.Homolog method)  is\_analog() (macsypy.gene.Gene method) | is\_authorized() (macsypy.gene.Gene method)  is\_complete() (macsypy.search\_systems.SystemOccurence method)  is\_forbidden() (macsypy.gene.Gene method)  is\_homolog() (macsypy.gene.Gene method)  is\_mandatory() (macsypy.gene.Gene method)  items() (macsypy.database.RepliconDB method)  iteritems() (macsypy.database.RepliconDB method) |

## J

json\_output() (macsypy.search\_systems.systemDetectionReportOrdered method)
:   (macsypy.search\_systems.systemDetectionReportUnordered method)

## L

loner (macsypy.gene.Gene attribute)

## M

|  |  |
| --- | --- |
| macsypy.config (module)  macsypy.database (module)  macsypy.gene (module), [1], [2], [3], [4], [5]  macsypy.macsypy\_error (module)  macsypy.report (module), [1], [2], [3], [4]  macsypy.search\_genes (module)  macsypy.search\_systems (module)  macsypy.system (module), [1]  macsypy.system\_parser (module)  MacsypyError  mandatory\_genes (macsypy.system.System attribute) | max (macsypy.database.RepliconInfo attribute)  max\_nb\_genes (macsypy.system.System attribute)  max\_nb\_genes() (macsypy.config.Config method)  min (macsypy.database.RepliconInfo attribute)  min\_genes\_required (macsypy.system.System attribute)  min\_genes\_required() (macsypy.config.Config method)  min\_mandatory\_genes\_required (macsypy.system.System attribute)  min\_mandatory\_genes\_required() (macsypy.config.Config method)  multi\_loci (macsypy.system.System attribute)  multi\_loci() (macsypy.config.Config method)  multi\_system (macsypy.gene.Gene attribute) |

## N

nb\_syst\_genes (macsypy.search\_systems.SystemOccurence attribute)

## O

|  |  |
| --- | --- |
| OrderedHMMReport (class in macsypy.report) | output\_system\_header() (macsypy.search\_systems.validSystemHit method) |

## P

|  |  |
| --- | --- |
| parse() (macsypy.system\_parser.SystemParser method)  previous\_run (macsypy.config.Config attribute)  Profile (class in macsypy.gene)  profile (macsypy.gene.Gene attribute) | profile\_dir (macsypy.config.Config attribute)  profile\_suffix (macsypy.config.Config attribute)  ProfileFactory (class in macsypy.gene)  putative\_system (macsypy.search\_systems.Cluster attribute) |

## R

|  |  |
| --- | --- |
| replicon\_infos() (macsypy.database.RepliconDB method)  replicon\_names() (macsypy.database.RepliconDB method)  replicon\_topology (macsypy.config.Config attribute)  RepliconDB (class in macsypy.database)  RepliconInfo (class in macsypy.database) | report\_output() (macsypy.search\_systems.systemDetectionReportOrdered method)  (macsypy.search\_systems.systemDetectionReportUnordered method)  res\_extract\_suffix (macsypy.config.Config attribute)  res\_search\_dir (macsypy.config.Config attribute)  res\_search\_suffix (macsypy.config.Config attribute) |

## S

|  |  |
| --- | --- |
| save() (macsypy.config.Config method)  (macsypy.search\_systems.Cluster method)  save\_extract() (macsypy.report.HMMReport method)  search\_genes() (in module macsypy.search\_genes)  search\_systems() (in module macsypy.search\_systems)  sequence\_db (macsypy.config.Config attribute)  state (macsypy.search\_systems.Cluster attribute)  (macsypy.search\_systems.SystemOccurence attribute)  summary\_output() (macsypy.search\_systems.systemDetectionReportOrdered method)  (macsypy.search\_systems.systemDetectionReportUnordered method)  System (class in macsypy.system)  system (macsypy.gene.Gene attribute)  system\_2\_json() (macsypy.search\_systems.systemDetectionReportOrdered method) | system\_to\_parse() (macsypy.system\_parser.SystemParser method)  SystemBank (class in macsypy.system)  SystemDetectionError  systemDetectionReportOrdered (class in macsypy.search\_systems)  systemDetectionReportUnordered (class in macsypy.search\_systems)  SystemInconsistencyError  SystemNameGenerator (class in macsypy.search\_systems)  SystemOccurence (class in macsypy.search\_systems)  SystemParser (class in macsypy.system\_parser) |

## T

|  |  |
| --- | --- |
| tabulated\_output() (macsypy.search\_systems.systemDetectionReportOrdered method)  tabulated\_output\_header() (macsypy.search\_systems.systemDetectionReportOrdered method) | topology (macsypy.database.RepliconInfo attribute)  topology\_file (macsypy.config.Config attribute) |

## V

validSystemHit (class in macsypy.search\_systems)

## W

|  |  |
| --- | --- |
| worker\_nb (macsypy.config.Config attribute) | working\_dir (macsypy.config.Config attribute) |

### Quick search


Enter search terms or a module, class or function name.

### Navigation

- index
- modules |
- MacSyFinder documentation »

© Copyright 2014, Sophie Abby, Bertrand Néron.
Created using Sphinx 1.1.3.
