# Supplementary material for: MacSyFinder: A Program to Mine Genomes for Molecular Systems with an Application to CRISPR-Cas Systems
Source: PLoS One. 2014 Oct 17;9(10):e110726. doi: 10.1371/journal.pone.0110726 (PMC4201578; doi:10.1371/journal.pone.0110726)
Supplement: Data S1 — The MacSyFinder/MacSyView package (compressed tarball archive). (GZ) [file pone.0110726.s010.gz › macsyfinder-1.0.0-RC3/doc/_build/html/HMMReport.html]

HMMReport API — MacSyFinder 1.0.0-RC3 documentation


### Navigation

- index
- modules |
- next |
- previous |
- MacSyFinder documentation »

# HMMReport API¶

A *“HMMReport”* object represents the results of a Hmmer program search on a dataset with a hidden Markov model protein profile (see *this section*).
This object has methods to extract and filter Hmmer raw outputs (see *generated output files*), and then build Hits relevant for system detection.
For matches selected with the filtering parameters, *“Hit”* objects (macsypy.HMMReport.Hit) are built.

## HMMReport API reference¶

*class* macsypy.report.HMMReport(*gene*, *hmmer\_output*, *cfg*)[source]¶
:   Handle the results from the HMM search. Extract a synthetic report from the raw hmmer output, after having applied a hit filtering.
    This class is an **abstract class**. There are two implementations of this abstract class
    depending on wether the input sequence dataset is “ordered” (“gembase” or “ordered\_replicon” db\_type) or not (“unordered” or “unordered\_replicon” db\_type).

    \_\_init\_\_(*gene*, *hmmer\_output*, *cfg*)[source]¶
    :   |  |  |
        | --- | --- |
        | Parameters: | - **gene** (macsypy.gene.Gene object) – the gene corresponding to the profile search reported here - **hmmer\_output** (*string*) – The path to the raw Hmmer output file - **cfg** (macsypy.config.Config object) – the configuration object |

    \_\_metaclass\_\_¶
    :   alias of ABCMeta

    \_\_str\_\_()[source]¶
    :   Print information on filtered hits

    \_\_weakref\_\_¶
    :   list of weak references to the object (if defined)

    \_build\_my\_db(*hmm\_output*)[source]¶
    :   Build the keys of a dictionary object to store sequence identifiers of hits.

        |  |  |
        | --- | --- |
        | Parameters: | **hmm\_output** (*string*) – the path to the hmmsearch output to parse. |
        | Returns: | a dictionary containing a key for each sequence id of the hits |
        | Return type: | dict |

    \_fill\_my\_db(*macsyfinder\_idx*, *db*)[source]¶
    :   Fill the dictionary with information on the matched sequences

        |  |  |
        | --- | --- |
        | Parameters: | - **macsyfinder\_idx** (*string*) – the path the macsyfinder index corresponding to the dataset - **db** (*dict*) – the database containing all sequence id of the hits. |

    \_hit\_start(*line*)[source]¶
    :   |  |  |
        | --- | --- |
        | Parameters: | **line** (*string*) – the line to parse |
        | Returns: | True if it’s the begining of a new hit in Hmmer raw output files. False otherwise |
        | Return type: | boolean. |

    \_parse\_hmm\_body(*hit\_id*, *gene\_profile\_lg*, *seq\_lg*, *coverage\_treshold*, *replicon\_name*, *position\_hit*, *i\_evalue\_sel*, *b\_grp*)[source]¶
    :   Parse the raw Hmmer output to extract the hits, and filter them with threshold criteria selected (“coverage\_profile” and “i\_evalue\_select” command-line parameters)

        |  |  |
        | --- | --- |
        | Parameters: | - **hit\_id** (*string*) – the sequence identifier - **gene\_profile\_lg** (*integer*) – the length of the profile matched - **seq\_lg** (*integer*) – the length of the sequence - **coverage\_treshold** (*float*) – the minimal coverage of the profile to be reached in the Hmmer alignment for hit selection - **replicon\_name** (*string*) – the identifier of the replicon - **position\_hit** (*integer*) – the rank of the sequence matched in the input dataset file - **i\_evalue\_sel** (*float*) – the maximal i-evalue (independent evalue) for hit selection - **b\_grp** (*list of list of strings*) – the Hmmer output lines to deal with (grouped by hit) |
        | Returns: | a set of hits |
        | Return type: | list of macsypy.report.Hit objects |

    \_parse\_hmm\_header(*h\_grp*)[source]¶
    :   |  |  |
        | --- | --- |
        | Parameters: | **h\_grp** (*sequence of string*) – the sequence of string return by groupby function representing the header of a hit |
        | Returns: | the sequence identifier from a set of lines that corresponds to a single hit |
        | Return type: | string |

    best\_hit()[source]¶
    :   Return the best hit among multiple hits

    extract()[source]¶
    :   Parse the raw Hmmer output file and produce a new synthetic report file by applying a filter on hits.
        Contain selected and sorted hits ( **this abstract method is implemented in inherited classes** )

    save\_extract()[source]¶
    :   Write the string representation of the extact report in a file.
        The name of this file is the concatenation of the gene name and of the “res\_extract\_suffix” from the config object

## GeneralHMMReport API reference¶

*class* macsypy.report.GeneralHMMReport(*gene*, *hmmer\_output*, *cfg*)[source]¶
:   Handle HMM report. Extract a synthetic report from the raw hmmer output.
    Dedicated to any type of ‘unordered’ datasets.

    extract()[source]¶
    :   Parse the output file of hmmer compute from an unordered genes base
        and produced a new synthetic report file.

## OrderedHMMReport¶

*class* macsypy.report.OrderedHMMReport(*gene*, *hmmer\_output*, *cfg*)[source]¶
:   Handle HMM report. Extract a synthetic report from the raw hmmer output.
    Dedicated to ‘ordered\_replicon’ datasets.

    extract()[source]¶
    :   Parse the output file of Hmmer obtained from a search in an ordered set of sequences
        and produce a new synthetic report file.

## GembaseHMMReport¶

*class* macsypy.report.GembaseHMMReport(*gene*, *hmmer\_output*, *cfg*)[source]¶
:   Handle HMM report. Extract a synthetic report from the raw hmmer output.
    Dedicated to ‘gembase’ format datasets.

    extract()[source]¶
    :   Parse the output file of Hmmer obtained from a search in a ‘gembase’ set of sequences
        and produce a new synthetic report file.

## Hit¶

*class* macsypy.report.Hit(*gene*, *system*, *hit\_id*, *hit\_seq\_length*, *replicon\_name*, *position\_hit*, *i\_eval*, *score*, *profile\_coverage*, *sequence\_coverage*, *begin\_match*, *end\_match*)[source]¶
:   Handle the hits filtered from the Hmmer search. The hits are instanciated by HMMReport.extract() method

    \_\_cmp\_\_(*other*)[source]¶
    :   Compare two Hits. If the sequence identifier is the same, do the comparison on the score. Otherwise, do it on alphabetical comparison of the sequence identifier.

        |  |  |
        | --- | --- |
        | Parameters: | **other** (macsypy.report.Hit object) – the hit to compare to the current object |
        | Returns: | the result of the comparison |

    \_\_eq\_\_(*other*)[source]¶
    :   Return True if two hits are totally equivalent, False otherwise.

        |  |  |
        | --- | --- |
        | Parameters: | **other** (macsypy.report.Hit object) – the hit to compare to the current object |
        | Returns: | the result of the comparison |
        | Return type: | boolean |

    \_\_init\_\_(*gene*, *system*, *hit\_id*, *hit\_seq\_length*, *replicon\_name*, *position\_hit*, *i\_eval*, *score*, *profile\_coverage*, *sequence\_coverage*, *begin\_match*, *end\_match*)[source]¶
    :   |  |  |
        | --- | --- |
        | Parameters: | - **gene** (macsypy.gene.Gene object) – the gene corresponding to this profile - **system** (macsypy.system.System object) – the system to which this gene belongs - **hit\_id** (*string*) – the identifier of the hit - **hit\_seq\_length** (*integer*) – the length of the hit sequence - **replicon\_name** (*string*) – the name of the replicon - **position\_hit** (*integer*) – the rank of the sequence matched in the input dataset file - **i\_eval** (*float*) – the best-domain evalue (i-evalue, “independent evalue”) - **score** (*float*) – the score of the hit - **profile\_coverage** (*float*) – percentage of the profile that matches the hit sequence - **sequence\_coverage** (*float*) – percentage of the hit sequence that matches the profile - **begin\_match** (*integer*) – where the hit with the profile starts in the sequence - **end\_match** (*integer*) – where the hit with the profile ends in the sequence |

    \_\_str\_\_()[source]¶
    :   Print useful information on the Hit: regarding Hmmer statistics, and sequence information

    \_\_weakref\_\_¶
    :   list of weak references to the object (if defined)

    get\_position()[source]¶
    :   |  |  |
        | --- | --- |
        | Returns: | the position of the hit (rank in the input dataset file) |
        | Return type: | integer |

    get\_syst\_inter\_gene\_max\_space()[source]¶
    :   |  |  |
        | --- | --- |
        | Returns: | the ‘inter\_gene\_max\_space’ parameter defined for the gene of the hit |
        | Return type: | integer |

### Table Of Contents

- HMMReport API
  - HMMReport API reference
  - GeneralHMMReport API reference
  - OrderedHMMReport
  - GembaseHMMReport
  - Hit

#### Previous topic

Profile API

#### Next topic

search\_genes API reference

### This Page

- Show Source

### Quick search


Enter search terms or a module, class or function name.

### Navigation

- index
- modules |
- next |
- previous |
- MacSyFinder documentation »

© Copyright 2014, Sophie Abby, Bertrand Néron.
Created using Sphinx 1.1.3.
