# Supplementary material for: MacSyFinder: A Program to Mine Genomes for Molecular Systems with an Application to CRISPR-Cas Systems
Source: PLoS One. 2014 Oct 17;9(10):e110726. doi: 10.1371/journal.pone.0110726 (PMC4201578; doi:10.1371/journal.pone.0110726)
Supplement: Data S1 — The MacSyFinder/MacSyView package (compressed tarball archive). (GZ) [file pone.0110726.s010.gz › macsyfinder-1.0.0-RC3/doc/_build/html/implementation.html]

MacSyFinder implementation overview — MacSyFinder 1.0.0-RC3 documentation


### Navigation

- index
- modules |
- next |
- previous |
- MacSyFinder documentation »

# MacSyFinder implementation overview¶

MacSyFinder is implemented with an object-oriented architecture. The objects are described in the current *API documentation*. An overview of the main classes used to model the systems to be detected is provided below.

The *“System”* class models the systems to detect and contains a list of instances of the *“Gene”* class, which models each component of a given System. The *“Homolog”* and *“Analog”* classes encapsulate a “Gene” and model respectively relationships of homology and analogy between components.

A *“Gene”* represents a component from the System and refers to an instance of the *“Profile”* object that corresponds to an hidden Markov model protein profile (used for sequence similarity search with the Hmmer program).

The *“Config”* class (see the *Configuration API*) handles the program parameters, including Hmmer search parameters, and the set of sequences to query (represented by the “Database” object).

The *“Database”* stores information on the dataset, including necessary information to detect systems in both linear and circular chromosomes (see the *Database API*).

A set of parsers and object factories are used to fill the objects from command-line and input files (*i.e.* the optional configuration file and the XML files describing the systems), and to ensure their uniqueness and integrity.

Once these objects are initialized and the detection is launched, Hmmer is executed on the sequences of the database (optionally in parallel) with a unique list of profiles corresponding to the systems to detect. Subsequently, Hmmer output files are parsed, and selected hits (given the search parameters provided) are used to fill *“Hits”* objects, which contain information for the detection of the systems.

During the treatment of the *“Hits”* for *“Systems”* detection, the occurrences of the systems (*“SystemOccurence”* objects) are filled, and the **decision rules** associated with the systems (quorum and co-localization in the case of an “ordered” dataset) are applied. See the following sections for more details on above objects.

## The System object¶

The *System object* represents a macromolecular system to detect.
It is defined *via* a definition file in XML stored in a dedicated location that can be specified *via* the configuration file, or the command-line (-d parameter). See *The XML hierarchy* for more details on the XML grammar.

An object *SystemParser* is used to build a system object from its XML definition file.

A system is named after the file name of its XML definition.
A system has an attribute inter\_gene\_max\_space which is an integer,
and three kind of components are listed in function of their presence in the system:

- The genes that must be present in the genome to define this system (“mandatory”).
- The genes that can be present, but do not have to be found in every case (“accessory”).
- The genes that must not be present in the system (“forbidden”).

Note

a complete description of macromolecular systems modelling is available in the section *Macromolecular systems definition*

## The Gene object¶

The *Gene object* represents genes encoding the protein components of a System.
Each Gene points out its System of origin (macsypy.system.System). A Gene must have a correponding HMM protein profile. These profiles are represented by Profile objects (macsypy.gene.Profile), and must be named after the gene name. For instance, the gene *gspD* will correspond to the “gspD.hmm” profile file. See *The Profile object*). A Gene has several properties described in the *Gene API*.

A Gene may have Homologs or Analogs. An *“Homolog”* (resp. *“Analog”*) object encapsulates a Gene and has a reference to the Gene it is homolog (resp. *“analog”*) to. See the *Homolog API* and *Analog API* for more details.

Warning

To optimize computation and to avoid concurrency problems when we search several systems, each gene must be instanciated only once, and stored in a *“gene\_bank”*.
gene\_bank is a macsypy.gene.GeneBank object.
The gene\_bank and system\_bank are filled by the system\_parser (macsypy.system\_parser.SystemParser)

## The Profile object¶

Each *“Gene”* component corresponds to a *“Profile”*. The *“Profile”* object is used for the search of the gene with Hmmer. Thus, a *“Profile”* must match a HMM file, which name is based on the profile name. For instance, the *gspG* gene has the corresponding “gspG.hmm” profile file provided at a dedicated location.

## Reporting Hmmer search results¶

A *“HMMReport”* (macsypy.report.HMMReport) object represents the results of a Hmmer program search on the input dataset with a hidden Markov model protein profile.
This object has methods to extract and build *“Hits”* that are then analyzed for systems assessment.

It analyses Hmmer raw outputs, and applies filters on the matches (according to *Hmmer options*). See *Hmmer results outputs* for details on the resulting output files.
For profile matches selected with the filtering parameters, *“Hit”* objects are built (see *the Hit API*).

### Table Of Contents

- MacSyFinder implementation overview
  - The System object
  - The Gene object
  - The Profile object
  - Reporting Hmmer search results

#### Previous topic

Macromolecular systems definition

#### Next topic

MacSyFinder functioning

### This Page

- Show Source

### Quick search


Enter search terms or a module, class or function name.

### Navigation

- index
- modules |
- next |
- previous |
- MacSyFinder documentation »

© Copyright 2014, Sophie Abby, Bertrand Néron.
Created using Sphinx 1.1.3.
