# Supplementary material for: MacSyFinder: A Program to Mine Genomes for Molecular Systems with an Application to CRISPR-Cas Systems
Source: PLoS One. 2014 Oct 17;9(10):e110726. doi: 10.1371/journal.pone.0110726 (PMC4201578; doi:10.1371/journal.pone.0110726)
Supplement: Data S1 — The MacSyFinder/MacSyView package (compressed tarball archive). (GZ) [file pone.0110726.s010.gz › macsyfinder-1.0.0-RC3/doc/_build/html/index.html]

Welcome to MacSyFinder’s documentation! — MacSyFinder 1.0.0-RC3 documentation


### Navigation

- index
- modules |
- next |
- MacSyFinder documentation »

# Welcome to MacSyFinder’s documentation!¶

> MacSyFinder is a program to model and detect macromolecular systems, genetic pathways... in protein datasets. In prokaryotes, these systems have often evolutionarily conserved properties: they are made of conserved components, and are encoded in compact loci (conserved genetic architecture). The user models these systems with MacSyFinder to reflect these conserved features, and to allow their efficient detection.
>
> Criteria for systems detection include **component content (quorum)**, and **genomic co-localization**. Each component corresponds to a hidden Markov model (HMM) protein profile to perform homology searches with the program Hmmer.
>
> In order to model macromolecular systems, the user:
> :   - builds or gather from databanks **HMM protein profiles** for components of interest,
>     - defines **decision rules** for each system in a dedicated XML grammar (see *Macromolecular systems definition*).

# Running MacSyFinder¶

- Installation
  - MacSyFinder dependencies
  - Installation procedure
  - Uninstalling MacSyFinder
- MacSyFinder Quick Start
  - First trial with a test dataset
  - Visualizing expected results with MacSyView
- Input and Options of MacSyFinder
  - Input dataset
  - Command-line options
  - Configuration file
  - In-house input files
- Output format
  - Hmmer results outputs
  - Systems detection results
  - Logs and configuration files
  - File for MacSyview: results.macsyfinder.json
- MacSyView: visualizing MacSyFinder’s results!
  - MacSyView How To
  - Graphical output description
  - Technology used
  - Screenshot

# MacSyFinder functioning¶

- Macromolecular systems definition
  - Principles
  - The XML hierarchy
- MacSyFinder implementation overview
  - The System object
  - The Gene object
  - The Profile object
  - Reporting Hmmer search results
- MacSyFinder functioning
  - Functioning overview
  - A. Searching for systems’ components
  - B. Assessing systems presence

# MacSyFinder API documentation¶

- Configuration API
  - Config API reference
- Database API
  - database API reference
- System API
  - SystemBank API reference
  - System API reference
- The Parser of Systems definitions
  - SystemParser API reference
- Gene API
  - GeneBank API reference
  - Gene API reference
  - Homolog API reference
  - Analog API reference
- Profile API
  - ProfileFactory API reference
  - Profile API reference
- HMMReport API
  - HMMReport API reference
  - GeneralHMMReport API reference
  - OrderedHMMReport
  - GembaseHMMReport
  - Hit
- search\_genes API reference
- search\_systems API reference
- MacSyFinder errors
  - macsypy\_error API reference

# Indices and tables¶

- *Index*
- *Module Index*
- *Search Page*

### Table Of Contents

- Welcome to MacSyFinder’s documentation!
- Running MacSyFinder
- MacSyFinder functioning
- MacSyFinder API documentation
- Indices and tables

#### Next topic

Installation

### This Page

- Show Source

### Quick search


Enter search terms or a module, class or function name.

### Navigation

- index
- modules |
- next |
- MacSyFinder documentation »

© Copyright 2014, Sophie Abby, Bertrand Néron.
Created using Sphinx 1.1.3.
