# Supplementary material for: MacSyFinder: A Program to Mine Genomes for Molecular Systems with an Application to CRISPR-Cas Systems
Source: PLoS One. 2014 Oct 17;9(10):e110726. doi: 10.1371/journal.pone.0110726 (PMC4201578; doi:10.1371/journal.pone.0110726)
Supplement: Data S1 — The MacSyFinder/MacSyView package (compressed tarball archive). (GZ) [file pone.0110726.s010.gz › macsyfinder-1.0.0-RC3/doc/_build/html/input.html]

Input and Options of MacSyFinder — MacSyFinder 1.0.0-RC3 documentation


### Navigation

- index
- modules |
- next |
- previous |
- MacSyFinder documentation »

# Input and Options of MacSyFinder¶

## Input dataset¶

The input dataset must be a set of protein sequences in **Fasta format** (see http://en.wikipedia.org/wiki/FASTA\_format).

The *base section* in the configuration file (see *Configuration file*) can be used to specify **the path** and the **type of dataset** to deal with, as well as the –sequence\_db and –db\_type parameters respectively, described in the *Command-line options* (see *Input options*).

> Four types of protein datasets are supported:
>
> > - *unordered* : a set of sequences (*e.g.* a metagenomic dataset)
> > - *unordered\_replicon* : a set of sequences corresponding to a complete genome (*e.g.* an unassembled complete genome)
> > - *ordered\_replicon* : a set of sequences corresponding to an ordered complete replicon (*e.g.* an assembled complete genome)
> > - *gembase* : a set of multiple ordered replicons, which format follows the convention described in *Gembase format*.

For “ordered” (“ordered\_replicon” or “gembase”) datasets only, MacSyFinder can take into account the **shape of the genome**: “linear”, or “circular” for detection. The default is set to “circular”.

> This can be set with the –replicon\_topology parameter from *Command-line options* (see *Input options*), or in the configuration in the *base section*.
>
> With the “gembase” format, it is possible to specify a topology per replicon with a topology file (see *Gembase format* and *Topology files*).

## Command-line options¶

Positional arguments:

```
systems               The systems to detect. This is an obligatory option
                      with no keyword associated to it. To detect all systems
                      described in .xml available, set to "all" (case insensitive).
                      Otherwise, a single or multiple systems can be specified.
                      For example: "SystemA SystemB".
```

Optional arguments:

```
-h, --help            Show the help message and exit
```

Input dataset options:

```
--sequence-db SEQUENCE_DB
                      Path to the sequence dataset in fasta format.

--db-type {unordered_replicon,ordered_replicon,gembase,unordered}
                      The type of dataset to deal with. "unordered_replicon"
                      corresponds to a non-assembled genome, "unordered" to
                      a metagenomic dataset, "ordered_replicon" to an
                      assembled genome, and "gembase" to a set of replicons
                      where sequence identifiers follow this convention:
                      ">RepliconName_SequenceID"

--replicon-topology {linear,circular}
                      The topology of the replicons (this option is
                      meaningful only if the db_type is 'ordered_replicon'
                      or 'gembase'

--topology-file TOPOLOGY-FILE
                      Topology file path. The topology file allows to
                      specify a topology (linear or circular) for each
                      replicon (this option is meaningful only if the
                      db_type is 'ordered_replicon' or 'gembase'. A topology
                      file is a tabular file with two columns: the 1st is
                      the replicon name, and the 2nd the corresponding
                      topology: "RepliconA linear"

--idx                 Forces to build the indexes for the sequence dataset
                      even if they were presviously computed and present at
                      the dataset location (default = False)
```

Systems detection options:

```
--inter-gene-max-space SYSTEM VALUE
                      Co-localization criterion: maximum number of
                      components non-matched by a profile allowed between
                      two matched components for them to be considered
                      contiguous. Option only meaningful for 'ordered'
                      datasets. The first value must match a system name,
                      the second a number of components. This option can be
                      repeated several times:
                      "--inter-gene-max-space T3SS 12 --inter-gene-max-space Flagellum 20"

--min-mandatory-genes-required SYSTEM VALUE
                      The minimal number of mandatory genes required for
                      system assessment. The first value must correspond to
                      a system name, the second value to an integer. This
                      option can be repeated several times:
                      "--min-mandatory-genes-required T2SS 15
                      --min-mandatory-genes-required Flagellum 10"

--min-genes-required SYSTEM VALUE
                      The minimal number of genes required for system
                      assessment (includes both 'mandatory' and 'accessory'
                      components). The first value must correspond to a
                      system name, the second value to an integer. This
                      option can be repeated several times:
                      "--min-genes-required T2SS 15 --min-genes-required Flagellum 10"

--max-nb-genes SYSTEM VALUE
                      The maximal number of genes allowed in the system.

--multi-loci SYSTEM
                      Specifies if the system can be detected as a 'scattered'
                      system. (default: False)
```

Options for Hmmer execution and hits filtering:

```
--hmmer HMMER_EXE     Path to the Hmmer program.

--index-db INDEX_DB_EXE
                      The indexer to be used for Hmmer. The value can be
                      either 'makeblastdb' or 'formatdb' or the path to one
                      of these binary (default = makeblastb).

--e-value-search E_VALUE_RES
                      Maximal e-value for hits to be reported during Hmmer
                      search. (default = 1)

--i-evalue-select I_EVALUE_SEL
                      Maximal independent e-value for Hmmer hits to be
                      selected for system detection. (default = 0.001)

--coverage-profile COVERAGE_PROFILE
                      Minimal profile coverage required in the hit alignment
                      to allow the hit selection for system detection.
                      (default = 0.5)
```

Path options:

```
-d DEF_DIR, --def DEF_DIR
                      Path to the systems definition files.

-r RES_SEARCH_DIR, --res-search-dir RES_SEARCH_DIR
                      Path to the directory where to store MacSyFinder search
                      results directories.

--res-search-suffix RES_SEARCH_SUFFIX
                      The suffix to give to Hmmer raw output files.

--res-extract-suffix RES_EXTRACT_SUFFIX
                      The suffix to give to filtered hits output files.

-p PROFILE_DIR, --profile-dir PROFILE_DIR
                      Path to the profiles directory.

--profile-suffix PROFILE_SUFFIX
                      The suffix of profile files. For each 'Gene' element,
                      the corresponding profile is searched in the
                      'profile_dir', in a file which name is based on the
                      Gene name + the profile suffix. For instance, if the
                      Gene is named 'gspG' and the suffix is '.hmm3', then
                      the profile should be placed in the specified folder
                      'profile_dir' and be named 'gspG.hmm3'.
                      (default: ".hmm")
```

General options:

```
-w WORKER_NB, --worker WORKER_NB
                      Number of workers to be used by MacSyFinder. In the case
                      the user wants to run MacSyFinder in a multi-thread mode.
                      All workers can be used with the value '0'. (default = 1)

-v, --verbosity       Increases the verbosity level. There are 4 levels:
                      Error messages (default), Warning (-v), Info (-vv) and
                      Debug(-vvv).

--log LOG_FILE        Path to the directory where to store the 'macsyfinder.log'
                      log file.

--config CFG_FILE     Path to a putative MacSyFinder configuration file to be
                      used.

--previous-run PREVIOUS_RUN
                      Path to a previous MacSyFinder run directory. It allows to
                      skip the Hmmer search step on same dataset, as it uses
                      previous run results and thus parameters regarding
                      Hmmer detection. The configuration file from this
                      previous run will be used.
                      It is in conflict with options:
                      --config,
                      --sequence_db,
                      --profile-suffix,
                      --res-extract-suffix,
                      --e-value-res,
                      --db-type,
                      --hmmer
```

## Configuration file¶

Options to run MacSyFinder can be specified in a configuration file. The *Config* handles all configuration options for MacSyFinder.
Three locations are parsed to find configuration files:

> - $PREFIX/etc/macsyfinder/macsyfinder.conf
> - $(HOME)/.macsyfinder/macsyfinder.conf
> - ./macsyfinder.conf

Moreover these three locations options can be passed on the command-line.

Each file can define options, at the end all options are added. If an option is specified several times:

Note

The precedence rules from the less important to the more important are:

$PREFIX/etc/macsyfinder/macsyfinder.conf < $(HOME)/.macsyfinder/macsyfinder.conf < ./macsyfinder.conf < “command-line” options

This means that command-line options will always bypass those from the configuration files. In the same flavor, options altering the definition of systems found in the command-line or the configuration file will always overwhelm values from systems’ *XML definition files*.

The configuration files must follow the Python “ini” file syntax.
The Config object provides some default values and performs some validations of the values, for instance:

In MacSyFinder, five sections are defined:

> > - **base** : all information related to the protein dataset under study
> >
> >   - *file* : the path to the dataset in Fasta format (*no default value*)
> >   - *type* : the type of dataset to handle, four types are supported:
> >
> >     > - *unordered* : a set of sequences (*e.g.* a metagenomic dataset)
> >     > - *unordered\_replicon* : a set of sequences corresponding to a complete replicon (*e.g.* an unassembled complete genome)
> >     > - *ordered\_replicon* : a set of sequences corresponding to a complete replicon ordered (*e.g.* an assembled complete genome)
> >     > - *gembase* : a set of multiple ordered replicons.
> >
> >     (*no default value*)
> >   - *replicon\_topology* : the topology of the replicon under study. Two topologies are supported: ‘linear’ and ‘circular’ (*default* = ‘circular’)
> >     This option will be ignored if the dataset type is not ordered (*i.e.* “unordered\_replicon” or “unordered”).
> > - **system**
> >
> >   - *inter\_gene\_max\_space* = list of system name and integer separated by spaces. These values will supersede the values found in the system definition file.
> >   - *min\_mandatory\_genes\_required* = list of system name and integer separated by spaces. These values will supersede the values found in the system definition file.
> >   - *min\_genes\_required* = list of system name and integer separated by spaces. These values will supersede the values found in the system definition file.
> > - **hmmer**
> >
> >   - *hmmer\_exe* (default= *hmmsearch* )
> >   - *index\_db\_exe* the executable to use to build the index for the hmm. The value can be ‘makeblastdb’ or ‘formatdb’ or the absolute path toward one of these two binaries (default= *makeblastdb* )
> >   - *e\_value\_res* = (default= *1* )
> >   - *i\_evalue\_sel* = (default= *0.5* )
> >   - *coverage\_profile* = (default= *0.5* )
> > - **directories**
> >
> >   - *res\_search\_dir* = (default= *./datatest/res\_search* )
> >   - *res\_search\_suffix* = (default= *.search\_hmm.out* )
> >   - *profile\_dir* = (default= *./profiles* )
> >   - *profile\_suffix* = (default= *.fasta-aln\_edit.hmm* )
> >   - *res\_extract\_suffix* = (default= *.res\_hmm\_extract* )
> >   - *def\_dir* = (default= *./DEF/* )
> > - **general**
> >
> >   - *log\_level*: (default= *debug* ) This corresponds to an integer code:
> >     :   | Level | Numeric value |
> >         | --- | --- |
> >         | CRITICAL | 50 |
> >         | ERROR | 40 |
> >         | WARNING | 30 |
> >         | INFO | 20 |
> >         | DEBUG | 10 |
> >         | NOTSET | 0 |
> >   - *log\_file* = (default = macsyfinder.log in directory of the run)

Example of a configuration file:

```
 [base]
 prefix = /path/to/macsyfinder/home/
 file = %(prefix)s/dataset/prru_psae.001.c01.fasta
 type = gembase
 replicon_topology = circular

 [system]
 inter_gene_max_space = T2SS 22 Flagellum 44
 min_mandatory_genes_required = T2SS 6 Flagellum 4
 min_genes_required = T2SS 8 Flagellum 4

 [hmmer]
 hmmer_exe = hmmsearch
 index_db_exe = makeblastdb
 e_value_res = 1
 i_evalue_sel = 0.5
 coverage_profile = 0.5

 [directories]
 prefix = /path/to/macsyfinder/home/
 def_dir = %(prefix)s/data/DEF
 res_search_dir = %(prefix)s/dataset/res_search/
 res_search_suffix = .raw_hmm
 profile_dir = %(prefix)s/data/profiles
 profile_suffix = .fasta-aln.hmm
 res_extract_suffix = .res_hmm

[general]
log_level = debug
```

Note

After a run, the corresponding configuration file (“macsyfinder.conf”) is generated as a (re-usable) output file that stores every options used in the run. It is stored in the results’ directory (see *the output section*).

## In-house input files¶

- Gembase format
- Topology files

### Table Of Contents

- Input and Options of MacSyFinder
  - Input dataset
  - Command-line options
  - Configuration file
  - In-house input files

#### Previous topic

MacSyFinder Quick Start

#### Next topic

Gembase format

### This Page

- Show Source

### Quick search


Enter search terms or a module, class or function name.

### Navigation

- index
- modules |
- next |
- previous |
- MacSyFinder documentation »

© Copyright 2014, Sophie Abby, Bertrand Néron.
Created using Sphinx 1.1.3.
