# Supplementary material for: MacSyFinder: A Program to Mine Genomes for Molecular Systems with an Application to CRISPR-Cas Systems
Source: PLoS One. 2014 Oct 17;9(10):e110726. doi: 10.1371/journal.pone.0110726 (PMC4201578; doi:10.1371/journal.pone.0110726)
Supplement: Data S1 — The MacSyFinder/MacSyView package (compressed tarball archive). (GZ) [file pone.0110726.s010.gz › macsyfinder-1.0.0-RC3/doc/_build/html/installation.html]

Installation — MacSyFinder 1.0.0-RC3 documentation


### Navigation

- index
- modules |
- next |
- previous |
- MacSyFinder documentation »

# Installation¶

## MacSyFinder dependencies¶

MacSyFinder has two dependencies:
:   - the *formatdb* (>=2.2.26) or *makeblastdb* (>=2.2.28) tools provided with the Blast suite of programs

(http://blast.ncbi.nlm.nih.gov/Blast.cgi?CMD=Web&PAGE\_TYPE=BlastDocs&DOC\_TYPE=Download)
:   - the program *Hmmer* version 3.0 (http://hmmer.janelia.org/).

*formatdb* or *makeblastdb* and *hmmsearch* programs should be installed (*e.g.*, in the PATH) in order to use MacSyFinder. Otherwise, the paths to these executables must be specified in the command-line: see the *command-line options*.

**Python version 2.7** is required to run MacSyFinder: https://docs.python.org/2.7/index.html

## Installation procedure¶

First unarchive the source codes package, and enter the extracted folder:

```
tar -xzvf macsyfinder-x.x.tar.gz
cd macsyfinder-x.x
```

MacSyFinder installation follows classical “pythonic” installation procedure (see http://docs.python.org/2/install/):

```
python setup.py build
(sudo) python setup.py install
```

It is **highly recommanded** to run tests before performing the full installation:

```
python setup.py build
python setup.py test -vv
(sudo) python setup.py install
```

Note

Super-user privileges (*i.e.*, sudo) are necesserary if you want to install the program in the general file architecture.

Note

If you do not have the privileges, or if you do not want to install MacSyFinder in the Python libraries of your system,
you can install MacSyFinder in a virtual environment (http://www.virtualenv.org/).

Procedures specific to MacSyFinder can be used instead of default. Please run the command for full options:

```
python setup.py --help
```

The main ones are:

```
python setup.py install --prefix /usr/local/home/bob/my_programs # Specifies an installation path
```

=> It will install MacSyFinder and required data (profiles folder and systems definition folders) in the Home directory of “bob”, in the “my\_progams” folder (useful if you do not have super-user privileges).

Warning

When installing a new version of MacSyFinder, do not forget to uninstall the previous version installed !

## Uninstalling MacSyFinder¶

To uninstall MacSyFinder (the last version installed), run:

```
(sudo) python setup.py uninstall
```

### Table Of Contents

- Installation
  - MacSyFinder dependencies
  - Installation procedure
  - Uninstalling MacSyFinder

#### Previous topic

Welcome to MacSyFinder’s documentation!

#### Next topic

MacSyFinder Quick Start

### This Page

- Show Source

### Quick search


Enter search terms or a module, class or function name.

### Navigation

- index
- modules |
- next |
- previous |
- MacSyFinder documentation »

© Copyright 2014, Sophie Abby, Bertrand Néron.
Created using Sphinx 1.1.3.
