# Supplementary material for: MacSyFinder: A Program to Mine Genomes for Molecular Systems with an Application to CRISPR-Cas Systems
Source: PLoS One. 2014 Oct 17;9(10):e110726. doi: 10.1371/journal.pone.0110726 (PMC4201578; doi:10.1371/journal.pone.0110726)
Supplement: Data S1 — The MacSyFinder/MacSyView package (compressed tarball archive). (GZ) [file pone.0110726.s010.gz › macsyfinder-1.0.0-RC3/doc/_build/html/macsypy_error.html]

MacSyFinder errors — MacSyFinder 1.0.0-RC3 documentation


### Navigation

- index
- modules |
- previous |
- MacSyFinder documentation »

# MacSyFinder errors¶

## macsypy\_error API reference¶

*exception* macsypy.macsypy\_error.MacsypyError[source]¶
:   The base class for MacSyFinder specific exceptions.

    \_\_weakref\_\_¶
    :   list of weak references to the object (if defined)

*exception* macsypy.macsypy\_error.SystemDetectionError[source]¶
:   Raised when the detection of systems from Hits encountered a problem.

*exception* macsypy.macsypy\_error.SystemInconsistencyError[source]¶
:   Raised when a secretion system is not consistent.

### Table Of Contents

- MacSyFinder errors
  - macsypy\_error API reference

#### Previous topic

search\_systems API reference

### This Page

- Show Source

### Quick search


Enter search terms or a module, class or function name.

### Navigation

- index
- modules |
- previous |
- MacSyFinder documentation »

© Copyright 2014, Sophie Abby, Bertrand Néron.
Created using Sphinx 1.1.3.
