# Supplementary material for: MacSyFinder: A Program to Mine Genomes for Molecular Systems with an Application to CRISPR-Cas Systems
Source: PLoS One. 2014 Oct 17;9(10):e110726. doi: 10.1371/journal.pone.0110726 (PMC4201578; doi:10.1371/journal.pone.0110726)
Supplement: Data S1 — The MacSyFinder/MacSyView package (compressed tarball archive). (GZ) [file pone.0110726.s010.gz › macsyfinder-1.0.0-RC3/doc/_build/html/macsyview.html]

MacSyView: visualizing MacSyFinder’s results! — MacSyFinder 1.0.0-RC3 documentation


### Navigation

- index
- modules |
- next |
- previous |
- MacSyFinder documentation »

# MacSyView: visualizing MacSyFinder’s results!¶

MacSyView is a standalone web-browser application to visualize MacSyFinder’s detected systems. MacSyView relies on JSON files outputted by MacSyFinder to display the list of detected systems, and a detailed view of each system. It allows visualizing the content of systems, their genomic context, and generates SVG files that can be exported for drawing purpose.

## MacSyView How To¶

1. Run MacSyFinder to detect your favorite system!
2. Launch MacSyView:

> - Either, **run the wrapper `macsyview`** installed with MacSyFinder’s binaries (*i.e.*, macsyfinder - for Linux).
> - Or **open with your web-browser the html page**: /usr/share/macsyview/index.html or /share/macsyview/index.html (or in the path specified during installation for data associated with MacSyFinder).

3. Select the .json output file in the output directory of the run
4. Choose the system you want to visualize in the list...
5. ...and here it is!

Note

The MacSyView application runs everything on the user’s computer, even if it uses the technologies of Web browsers. No data are sent out of the user’s device.

## Graphical output description¶

The content of the system view depends on the type of the input dataset.

- upper panel: an overview of the effectives of detected components is displayed **for all types** of datasets, per type of components in the system definition. It is a direct representation of how the definition was fulfilled during detection.
- middle panel: only for **ordered datasets**, the detected system is shown in its genomic context, including nearby proteins that were not annotated as system’s components.
- lower panel: for **all datasets**, a table containing information on detected components (and eventually nearby proteins for ordered datasets) is displayed. It includes sequence information, and in the case of system’s components, Hmmer hit information, and function assigned in the system.

## Technology used¶

MacSyView was coded in Javascript and uses third-party libraries that are all accredited in the COPYRIGHT file distributed with the MacSyFinder/MacSyView package.

It includes among others:

> - the Raphael library for systems drawing,
> - the Bootstrap library for HTML design and
> - the Mustache library for HTML templating in Javascript.
>
> The JQuery, JQuery-mousewheel and Raphael.Export libraries were also used.

It was tested on Chromium and Firefox for Linux, and on Chrome, Firefox and Safari for Mac OS X.

## Screenshot¶

Here is a view of one of the three systems detected with the example dataset *presented here*:

### Table Of Contents

- MacSyView: visualizing MacSyFinder’s results!
  - MacSyView How To
  - Graphical output description
  - Technology used
  - Screenshot

#### Previous topic

Output format

#### Next topic

Macromolecular systems definition

### This Page

- Show Source

### Quick search


Enter search terms or a module, class or function name.

### Navigation

- index
- modules |
- next |
- previous |
- MacSyFinder documentation »

© Copyright 2014, Sophie Abby, Bertrand Néron.
Created using Sphinx 1.1.3.
