# Supplementary material for: MacSyFinder: A Program to Mine Genomes for Molecular Systems with an Application to CRISPR-Cas Systems
Source: PLoS One. 2014 Oct 17;9(10):e110726. doi: 10.1371/journal.pone.0110726 (PMC4201578; doi:10.1371/journal.pone.0110726)
Supplement: Data S1 — The MacSyFinder/MacSyView package (compressed tarball archive). (GZ) [file pone.0110726.s010.gz › macsyfinder-1.0.0-RC3/doc/_build/html/outputs.html]

Output format — MacSyFinder 1.0.0-RC3 documentation


### Navigation

- index
- modules |
- next |
- previous |
- MacSyFinder documentation »

# Output format¶

MacSyFinder provides different types of outputs. At each run, MacSyFinder creates a new folder, whose name is based on a fixed prefix and a random suffix, for instance “macsyfinder-20130128\_08-57-46”. MacSyFinder outputs are stored in this run-specific folder.

## Hmmer results outputs¶

Raw Hmmer outputs are provided, as long with processed tabular outputs that includes hits filtered as specified by the user. For instance, the Hmmer search for SctC homologs with the corresponding profile will produce as a result two files: “sctC.search\_hmm.out” and “sctC.res\_hmm\_extract”.

The processed output “sctC.res\_hmm\_extract” recalls on the first lines the parameters used for hits filtering and relevant information on the matches, as
for instance:

```
# gene: sctC extract from /Users/bob/macsyfinder_results/
      macsyfinder-20130128_08-57-46/sctC.search_hmm.out hmm output
# profile length= 544
# i_evalue threshold= 0.001000
# coverage threshold= 0.500000
# hit_id replicon_name position_hit hit_sequence_length gene_name gene_system i_eval score
      profile_coverage sequence_coverage begin end
PSAE001c01_006940       PSAE001c01      3450    803     sctC    T3SS    1.1e-41 141.6
      0.588235  0.419676        395     731
PSAE001c01_018920       PSAE001c01      4634    776     sctC    T3SS    9.2e-48 161.7
      0.976103  0.724227        35      596
PSAE001c01_031420       PSAE001c01      5870    658     sctC    T3SS    2.7e-52 176.7
      0.963235  0.844985        49      604
PSAE001c01_051090       PSAE001c01      7801    714     sctC    T3SS    1.9e-46 157.4
      0.571691  0.463585        374     704
```

Note

Each tabular output file contains a header line describing each column in the output.

## Systems detection results¶

Different types of tabular outputs are provided. Headers are provided with the content of the lines in the file.

> - macsyfinder.tab - for **“ordered” datasets only** (db\_type is “ordered\_replicon” or “gembase”). It provides a summary of the number of each type of systems that have been detected.
> - macsyfinder.report - contains all the sequence identifiers of proteins detected as being part of a system, along with statistics on the Hmmer hit, and the status of the component in the system.
> - macsyfinder.summary - contains a line of information for each detected system.

### macsyfinder.tab¶

For each replicon, a line gives the occurrences of systems that were asked for detection. For example, if the detection was run for the Flagellum and the T6SS, the output will look like:

```
#Replicon Flagellum_single_locus Flagellum_multi_loci T6SS_single_locus T6SS_multi_loci
escherichia06 1   1   1   0
```

which means that this “escherichia06” genome harbors 1 flagellum in a single locus, 1 flagellum scattered on multiple loci, and 1 T6SS in a single locus.

### macsyfinder.report¶

Each line corresponds to a “hit” that has been assigned to a detected system. It includes:
:   - Hit\_Id - the sequence identifier of the hit
    - Replicon\_name - the name of the replicon it belongs to
    - Position - the position of the sequence in the replicon
    - Sequence\_length - the length of the sequence
    - Gene - the name of the components matched with the profile
    - Reference\_system - the system that includes the component matched
    - Predicted\_system - the system assigned
    - System\_Id - the unique identifier attributed to the detected system
    - System\_status - the status of the detected system
    - Gene\_status - the status of the component in the assigned system’s definition
    - i-evalue - Hmmer statistics, the indepent-evalue
    - Score - Hmmer score
    - Profile\_coverage - the percentage of the profile covered by the alignment with the sequence
    - Sequence\_coverage - the percentage of the sequence covered by the alignment with the profile
    - Begin\_match - the position in the sequence where the profile match begins
    - End\_match - the position in the sequence where the profile match ends

### macsyfinder.summary¶

Each line corresponds to a system that has been detected. It includes:
:   - Replicon\_name - the name of the replicon
    - System\_Id - the unique identifier attributed to the detected system
    - Reference\_system - the type of system detected
    - System\_status - the status of the system
    - Nb\_loci - the number of loci that constitutes the system
    - Nb\_Ref\_mandatory - the number of mandatory genes in the system definition
    - Nb\_Ref\_accessory - the number of accessory genes in the system definition
    - Nb\_Ref\_Genes\_detected\_NR - the number of different components (accessory+mandatory) in the system
    - Nb\_Genes\_with\_match - the number of components detected with the profiles in the system
    - System\_length - the full number of components (with match or not) in the locus (or loci) that constitutes the system
    - Nb\_Mandatory\_NR - the number of different mandatory components matched
    - Nb\_Accessory\_NR - the number of different accessory components matched
    - Nb\_missing\_mandatory - the number of mandatory components from the system definition with no match in this system occurrence
    - Nb\_missing\_accessory - the number of accessory components from the system definition with no match in this system occurrence
    - List\_missing\_mandatory - the list of the missing mandatory components
    - List\_missing\_accessory - the list of the missing accessory components
    - Loci\_positions - the sequence position (rank of the fasta sequence in the input sequence file) of the different loci encoding the system
    - Occur\_Mandatory - counts of the mandatory components
    - Occur\_Accessory - counts of the accessory components
    - Occur\_Forbidden - counts of the forbidden components

## Logs and configuration files¶

Three specific output files are built to store information on the MacSyFinder execution:

> - macsyfinder.out - contains information on the procedure during systems detection: clusters found, decisions made for system inference... The same information is also displayed on the standard output.
> - macsyfinder.conf - contains the configuration information of the run. It is useful to recover the parameters used for the run.
> - macsyfinder.log - the log file, contains raw information on the run. Please send it to us with any bug report.

## File for MacSyview: results.macsyfinder.json¶

This file in JSON format is used by MacSyView, for graphical output purpose. It must be loaded through MacSyView to graphically visualize detected systems. For more details, see *MacSyView’s description*.

### Table Of Contents

- Output format
  - Hmmer results outputs
  - Systems detection results
    - macsyfinder.tab
    - macsyfinder.report
    - macsyfinder.summary
  - Logs and configuration files
  - File for MacSyview: results.macsyfinder.json

#### Previous topic

Gembase format

#### Next topic

MacSyView: visualizing MacSyFinder’s results!

### This Page

- Show Source

### Quick search


Enter search terms or a module, class or function name.

### Navigation

- index
- modules |
- next |
- previous |
- MacSyFinder documentation »

© Copyright 2014, Sophie Abby, Bertrand Néron.
Created using Sphinx 1.1.3.
