# Supplementary material for: MacSyFinder: A Program to Mine Genomes for Molecular Systems with an Application to CRISPR-Cas Systems
Source: PLoS One. 2014 Oct 17;9(10):e110726. doi: 10.1371/journal.pone.0110726 (PMC4201578; doi:10.1371/journal.pone.0110726)
Supplement: Data S1 — The MacSyFinder/MacSyView package (compressed tarball archive). (GZ) [file pone.0110726.s010.gz › macsyfinder-1.0.0-RC3/doc/_build/html/profile.html]

Profile API — MacSyFinder 1.0.0-RC3 documentation


### Navigation

- index
- modules |
- next |
- previous |
- MacSyFinder documentation »

# Profile API¶

The *Profile object* is used for the search of the gene with Hmmer. A *“Profile”* must match a HMM protein profile file, which name is based on the profile name. For instance, the *gspG* gene has the corresponding “gspG.hmm” profile file provided at a dedicated location.

## ProfileFactory API reference¶

> *class* macsypy.gene.ProfileFactory[source]¶
> :   Build and store all Profile objects. Profiles must not be instanciated directly.
>     The profile\_factory must be used. The profile\_factory ensures there is only one instance
>     of profile for a given name.
>     To get a profile, use the method get\_profile. If the profile is already cached, this instance is returned.
>     Otherwise a new profile is built, stored in the profile\_factory and then returned.
>
>     get\_profile(*gene*, *cfg*, *profiles\_registry*)[source]¶
>     :   |  |  |
>         | --- | --- |
>         | Parameters: | - **gene** (macsypy.gene.Gene or macsypy.gene.Homolog or macsypy.gene.Analog object) – the gene associated to this profile - **profiles\_registry** (*the registry of profiles*) – the registry where are stored the path of the profiles - **profiles\_registry** – macsypy.registries.ProfilesRegistry instance. |
>         | Returns: | the profile corresponding to the name. If the profile already exists, return it. Otherwise build it, store it and return it. |
>         | Return type: | macsypy.gene.Profile object |

## Profile API reference¶

*class* macsypy.gene.Profile(*gene*, *cfg*, *path*)[source]¶
:   Handle a HMM protein profile

    \_\_init\_\_(*gene*, *cfg*, *path*)[source]¶
    :   |  |  |
        | --- | --- |
        | Parameters: | - **gene** – the gene corresponding to this profile - **cfg** (macsypy.config.Config object) – the configuration - **path** (*string*) – the path to the hmm profile. |

    \_\_len\_\_()[source]¶
    :   |  |  |
        | --- | --- |
        | Returns: | the length of the HMM protein profile |
        | Return type: | int |

    \_\_str\_\_()[source]¶
    :   Print the name of the corresponding gene and the path to the HMM profile.

    \_\_weakref\_\_¶
    :   list of weak references to the object (if defined)

    \_len()[source]¶
    :   Parse the HMM profile file to get and store the length.
        This private method is called at the Profile init.

    execute()[source]¶
    :   Launch the Hmmer search (hmmsearch executable) with this profile

        |  |  |
        | --- | --- |
        | Returns: | an object storing information on th results of the HMM search (HMMReport) |
        | Return type: | macsypy.report.HMMReport object |

### Table Of Contents

- Profile API
  - ProfileFactory API reference
  - Profile API reference

#### Previous topic

Gene API

#### Next topic

HMMReport API

### This Page

- Show Source

### Quick search


Enter search terms or a module, class or function name.

### Navigation

- index
- modules |
- next |
- previous |
- MacSyFinder documentation »

© Copyright 2014, Sophie Abby, Bertrand Néron.
Created using Sphinx 1.1.3.
