# Supplementary material for: MacSyFinder: A Program to Mine Genomes for Molecular Systems with an Application to CRISPR-Cas Systems
Source: PLoS One. 2014 Oct 17;9(10):e110726. doi: 10.1371/journal.pone.0110726 (PMC4201578; doi:10.1371/journal.pone.0110726)
Supplement: Data S1 — The MacSyFinder/MacSyView package (compressed tarball archive). (GZ) [file pone.0110726.s010.gz › macsyfinder-1.0.0-RC3/doc/_build/html/py-modindex.html]

Python Module Index — MacSyFinder 1.0.0-RC3 documentation


### Navigation

- index
- modules |
- MacSyFinder documentation »

# Python Module Index

**m**

|  |  |  |
| --- | --- | --- |
|  |  |  |
|  | **m** |  |
|  | macsypy |  |
|  | macsypy.config |  |
|  | macsypy.database |  |
|  | macsypy.gene |  |
|  | macsypy.macsypy\_error |  |
|  | macsypy.report |  |
|  | macsypy.search\_genes |  |
|  | macsypy.search\_systems |  |
|  | macsypy.system |  |
|  | macsypy.system\_parser |  |

### Quick search


Enter search terms or a module, class or function name.

### Navigation

- index
- modules |
- MacSyFinder documentation »

© Copyright 2014, Sophie Abby, Bertrand Néron.
Created using Sphinx 1.1.3.
