# Supplementary material for: MacSyFinder: A Program to Mine Genomes for Molecular Systems with an Application to CRISPR-Cas Systems
Source: PLoS One. 2014 Oct 17;9(10):e110726. doi: 10.1371/journal.pone.0110726 (PMC4201578; doi:10.1371/journal.pone.0110726)
Supplement: Data S1 — The MacSyFinder/MacSyView package (compressed tarball archive). (GZ) [file pone.0110726.s010.gz › macsyfinder-1.0.0-RC3/doc/_build/html/quickstart.html]

MacSyFinder Quick Start — MacSyFinder 1.0.0-RC3 documentation


### Navigation

- index
- modules |
- next |
- previous |
- MacSyFinder documentation »

# MacSyFinder Quick Start¶

In order to run MacSyFinder on your favorite dataset as soon as you have installed it, you can simply follow the next steps:

- Type:
  “macsyfinder -h”
  to see all options available. All command-line options are described in the *Command-line options section*.
- On a “metagenomic” dataset for example:

  “macsyfinder --db-type unordered --sequence-db metagenome.fasta all”
  will detect all systems modelled in .xml files placed in the default definition folder in a metagenomic dataset.

  “macsyfinder --db-type unordered --sequence-db metagenome.fasta -d mydefinitions/ all”
  will detect all systems modelled in .xml files placed in the *“mydefinitions”* folder.
- On a completely assembled genome (where the gene order is known, and is relevant for systems detection):

  “macsyfinder --db-type ordered-replicon --sequence-db mygenome.fasta -d mydefinitions/ SystemA SystemB”
  will detect the systems *“SystemA”* and *“SystemB”* in a complete genome from *“SystemA.xml”* and *“SystemB.xml”* definition files placed in the folder *“mydefinitions”*.

See *Input dataset* for more on input datasets.

Note

Systems have to be spelled in a case-sensitive way to run their detection from the command-line. The name of the system corresponds to the suffix defined for xml files (.xml by default), for example *“toto”* for a system defined in *“toto.xml”*.

The *“all”* keyword allows to detect all systems available in the definition folder in a single run. See the *Command-line options*.

## First trial with a test dataset¶

We included a test dataset in the MacSyFinder package. **By default, it will be installed** in /share/macsyfinder or /usr/share/macsyfinder. But it can be located elsewhere if it was specified during installation.

This dataset consists in the detection of CRISPR-Cas SubTypes with the definitions in the /share/macsyfinder/DEF folder, using the profiles in the /share/macsyfinder/profiles folder. This classification was previously described in Makarova et al. 2011, and the profiles are from the TIGRFAM database (release 13 of August 15 2012) and some of them were specifically designed for CRISPR-Cas classification (Haft et. al, 2005). The definitions are detailed in the MacSyFinder’s paper.

As a sequence dataset, we propose three replicons in /share/macsyfinder/sequence\_data/datatest\_gembase.fasta:
:   - *Escherichia coli* str. K-12 substr. MG1655 chromosome (ESCO001c01a). Genbank accession number: NC\_000913.
    - *Haloarcula marismortui* ATCC 43049 plasmid pNG400 (HAMA001p04a). Genbank accession number: NC\_006392.
    - *Legionella pneumophila* str. Paris, complete genome (LEPN003c01a). Genbank accession number: NC\_006368.

They were concatenated in a single fasta file, following the “gembase” format proposed *here*, and thus MacSyfinder will treat the three different replicons separately for systems inference.

To run the detection and classification of all subtypes, type:

```
"macsyfinder --db-type gembase --sequence-db
/share/macsyfinder/sequence_data/datatest_gembase.fasta all"
```

To run the detection of the Type-IE subtype only, type:

```
"macsyfinder --db-type gembase --sequence-db
/share/macsyfinder/sequence_data/datatest_gembase.fasta CAS-TypeIE"
```

A sample topology file is included /share/macsyfinder/sequence\_data/datatest\_gembase.topology, and follows the convention in *here*. It allows to specify a different topology “linear” or “circular” for each replicon in the “gembase” format. Otherwise, by default the topology is set to “circular”. It can also be specified in the commmand-line (see the *Command-line options*).

To run the detection using the topology file, type:

```
"macsyfinder --db-type gembase --sequence-db
/share/macsyfinder/sequence_data/datatest_gembase.fasta
--topology-file /share/macsyfinder/sequence_data/datatest_gembase.topology all"
```

## Visualizing expected results with MacSyView¶

To have an idea of what should be detected with the above test dataset, run *MacSyView*, the web-browser application for MacSyFinder’s results visualization. To do that, open the expected JSON result file with MacSyView: /share/macsyfinder/sequence\_data/results.macsyfinder.json.

A screenshot of MacSyView is included *here*.

### Table Of Contents

- MacSyFinder Quick Start
  - First trial with a test dataset
  - Visualizing expected results with MacSyView

#### Previous topic

Installation

#### Next topic

Input and Options of MacSyFinder

### This Page

- Show Source

### Quick search


Enter search terms or a module, class or function name.

### Navigation

- index
- modules |
- next |
- previous |
- MacSyFinder documentation »

© Copyright 2014, Sophie Abby, Bertrand Néron.
Created using Sphinx 1.1.3.
