# Supplementary material for: MacSyFinder: A Program to Mine Genomes for Molecular Systems with an Application to CRISPR-Cas Systems
Source: PLoS One. 2014 Oct 17;9(10):e110726. doi: 10.1371/journal.pone.0110726 (PMC4201578; doi:10.1371/journal.pone.0110726)
Supplement: Data S1 — The MacSyFinder/MacSyView package (compressed tarball archive). (GZ) [file pone.0110726.s010.gz › macsyfinder-1.0.0-RC3/doc/_build/html/search_genes.html]

search\_genes API reference — MacSyFinder 1.0.0-RC3 documentation


### Navigation

- index
- modules |
- next |
- previous |
- MacSyFinder documentation »

# search\_genes API reference¶

macsypy.search\_genes.search\_genes(*genes*, *cfg*)[source]¶
:   For each gene of the list, use the corresponding profile to perform an Hmmer search, and parse the output
    to generate a HMMReport that is saved in a file after Hit filtering. These tasks are performed in parallel using threads.
    The number of workers can be limited by worker\_nb directive in the config object or in the command-line with the “-w” option.

    |  |  |
    | --- | --- |
    | Parameters: | - **genes** (list of macsypy.gene.Gene objects) – the genes to search in the input sequence dataset - **cfg** (macsypy.config.Config object) – the configuration object |

#### Previous topic

HMMReport API

#### Next topic

search\_systems API reference

### This Page

- Show Source

### Quick search


Enter search terms or a module, class or function name.

### Navigation

- index
- modules |
- next |
- previous |
- MacSyFinder documentation »

© Copyright 2014, Sophie Abby, Bertrand Néron.
Created using Sphinx 1.1.3.
