# Supplementary material for: MacSyFinder: A Program to Mine Genomes for Molecular Systems with an Application to CRISPR-Cas Systems
Source: PLoS One. 2014 Oct 17;9(10):e110726. doi: 10.1371/journal.pone.0110726 (PMC4201578; doi:10.1371/journal.pone.0110726)
Supplement: Data S1 — The MacSyFinder/MacSyView package (compressed tarball archive). (GZ) [file pone.0110726.s010.gz › macsyfinder-1.0.0-RC3/doc/_build/html/search_systems.html]

search\_systems API reference — MacSyFinder 1.0.0-RC3 documentation


### Navigation

- index
- modules |
- next |
- previous |
- MacSyFinder documentation »

# search\_systems API reference¶

*class* macsypy.search\_systems.Cluster(*systems\_to\_detect*)[source]¶
:   Stores a set of contiguous hits. The Cluster object can have different states regarding
    its content in different genes’ systems:

    > - ineligible: not a cluster to analyze
    > - clear: a single system is represented in the cluster
    > - ambiguous: several systems are represented in the cluster => might need a disambiguation

    \_\_init\_\_(*systems\_to\_detect*)[source]¶
    :   |  |  |
        | --- | --- |
        | Parameters: | **systems\_to\_detect** (a list of macsypy.system.System) – the list of systems to be detected in this run |

    \_\_len\_\_()[source]¶
    :   |  |  |
        | --- | --- |
        | Returns: | the length of the Cluster, *i.e.*, the number of hits stored in it |
        | Return type: | integer |

    \_\_str\_\_()[source]¶
    :   print of the Cluster’s hits stored in terms of components, and corresponding sequence identifier and positions

    \_\_weakref\_\_¶
    :   list of weak references to the object (if defined)

    add(*hit*)[source]¶
    :   Add a Hit to a Cluster.
        Hits are always added at the end of the cluster (appended to the list of hits). Thus, ‘begin’ and ‘end’ positions of the Cluster are always the position of the 1st and of the last hit respectively.

        |  |  |
        | --- | --- |
        | Parameters: | **hit** (a macsypy.report.Hit) – the Hit to add |
        | Raise : | a macsypy.macsypy\_error.SystemDetectionError |

    compatible\_systems[source]¶
    :   |  |  |
        | --- | --- |
        | Returns: | the list of the names of compatible systems represented by the cluster |
        | Return type: | string |

    putative\_system[source]¶
    :   |  |  |
        | --- | --- |
        | Returns: | the name of the putative system represented by the cluster |
        | Return type: | string |

    save(*force=False*)[source]¶
    :   Check the status of the cluster regarding systems which have hits in it.
        Update systems represented, and assign a putative system (self.\_putative\_system), which is the system with most hits in the cluster.
        The systems represented are stored in a dictionary in the self.systems variable.
        The execution of this function can be forced, even if it has already run for the cluster with the option force=True.

    state[source]¶
    :   |  |  |
        | --- | --- |
        | Returns: | the state of the Cluster of hits |
        | Return type: | string |

*class* macsypy.search\_systems.ClustersHandler[source]¶
:   Deals with sets of clusters found in a dataset. Conceived to store only clusters from a same replicon.

    \_\_init\_\_()[source]¶
    :   |  |  |
        | --- | --- |
        | Parameters: | **cfg** (macsypy.config.Config) – The configuration object built from default and user parameters. |

    \_\_weakref\_\_¶
    :   list of weak references to the object (if defined)

    circularize(*rep\_info*, *end\_hits*, *systems\_to\_detect*)[source]¶
    :   This function takes into account the circularity of the replicon by merging clusters when appropriate (typically at replicon’s ends).
        It has to be called only if the replicon\_topology is set to “circular”.

        |  |  |
        | --- | --- |
        | Parameters: | - **rep\_info** (a namedTuple “RepliconInfo” macsypy.database.RepliconInfo) – an entry extracted from the macsypy.database.RepliconDB - **end\_hits** (a list of macsypy.report.Hit) – a set of hits at ends of the replicon that were not introduced in clusters, and that might be part of a system overlapping the two “ends” of the replicon - **systems\_to\_detect** (a list of :class:`macsypy.system.System) – the set of systems to detect in this run |

*class* macsypy.search\_systems.SystemNameGenerator[source]¶
:   Creates and stores the names of detected systems. Ensures the uniqueness of the names.

    \_\_weakref\_\_¶
    :   list of weak references to the object (if defined)

    \_computeBasename(*replicon*, *system*)[source]¶
    :   Computes the base name to be used for unique name generation

        |  |  |
        | --- | --- |
        | Parameters: | - **replicon** (*string*) – the replicon name - **system** (*string*) – the system name |
        | Returns: | the base name |
        | Return type: | string |

    getSystemName(*replicon*, *system*)[source]¶
    :   Generates a unique system name based on the replicon’s name and the system’s name.

        |  |  |
        | --- | --- |
        | Parameters: | - **replicon** (*string*) – the replicon name - **system** (*string*) – the system name |
        | Returns: | a unique system name |
        | Return type: | string |

*class* macsypy.search\_systems.SystemOccurence(*system*)[source]¶
:   This class is instantiated for a specific system that has been asked for detection. It can be filled step by step with hits.
    A decision can then be made according to the parameters defined *e.g.* quorum of genes.

    The SystemOccurence object has a “state” parameter, with the possible following values:
    :   - “empty” if the SystemOccurence has not yet been filled with genes of the decision rule of the system
        - “no\_decision” if the filling process has started but the decision rule has not yet been applied to this occurence
        - “single\_locus”
        - “multi\_loci”
        - “uncomplete”

    \_\_init\_\_(*system*)[source]¶
    :   |  |  |
        | --- | --- |
        | Parameters: | **system** (macsypy.system.System) – the system to “fill” with hits. |

    \_\_str\_\_()[source]¶
    :   |  |  |
        | --- | --- |
        | Returns: | Information of the component content of the SystemOccurence. |
        | Return type: | string |

    \_\_weakref\_\_¶
    :   list of weak references to the object (if defined)

    compute\_missing\_genes\_list(*gene\_dict*)[source]¶
    :   |  |  |
        | --- | --- |
        | Parameters: | **gene\_dict** (*dict*) – a dictionary with gene’s names as keys and number of occurrences as values |
        | Returns: | the list of genes with no occurence in the gene counter. |
        | Return type: | list |

    compute\_system\_length(*rep\_info*)[source]¶
    :   Returns the length of the system, all loci gathered, in terms of protein number (even those not matching any system gene)

        |  |  |
        | --- | --- |
        | Parameters: | **rep\_info** (a namedTuple “RepliconInfo” macsypy.database.RepliconInfo) – an entry extracted from the macsypy.database.RepliconDB |
        | Return type: | integer |

    count\_genes(*gene\_dict*)[source]¶
    :   Counts the number of genes with at least one occurrence in a dictionary with a counter of genes.

        |  |  |
        | --- | --- |
        | Parameters: | **gene\_dict** (*dict*) – a dictionary with gene’s names as keys and number of occurrences as values |
        | Return type: | integer |

    count\_genes\_tot(*gene\_dict*)[source]¶
    :   Counts the number of matches in a dictionary with a counter of genes, independently of the nb of genes matched.

        |  |  |
        | --- | --- |
        | Parameters: | **gene\_dict** (*dict*) – a dictionary with gene’s names as keys and number of occurrences as values |
        | Return type: | integer |

    count\_missing\_genes(*gene\_dict*)[source]¶
    :   Counts the number of genes with no occurence in the gene counter.

        |  |  |
        | --- | --- |
        | Parameters: | **gene\_dict** (*dict*) – a dictionary with gene’s names as keys and number of occurrences as values |
        | Return type: | integer |

    decision\_rule()[source]¶
    :   This function applies the decision rules for system assessment in terms of quorum:
        :   - the absence of forbidden genes is checked
            - the minimal number of mandatory genes is checked (“min\_mandatory\_genes\_required”)
            - the minimal number of genes in the system is checked (“min\_genes\_required”)

        When a decision is made, the status (self.status) of the
        macsypy.search\_systems.SystemOccurence is set either to:

        > - “single\_locus” when a complete system in the form of a single cluster was found
        > - “multi\_loci” when a complete system in the form of several clusters was found
        > - “uncomplete” when no system was assessed (quorum not reached)
        > - “empty” when no gene for this system was found
        > - “exclude” when no system was assessed (at least one forbidden gene was found)

        |  |  |
        | --- | --- |
        | Returns: | a printable message of the output decision with this SystemOccurrence |
        | Return type: | string |

    fill\_with\_cluster(*cluster*)[source]¶
    :   Adds hits from a cluster to a system occurence, and check which are their status according to the system definition.
        Set the system occurence state to “no\_decision” after calling of this function.

        |  |  |
        | --- | --- |
        | Parameters: | **cluster** (macsypy.search\_systems.Cluster) – the set of contiguous genes to treat for macsypy.search\_systems.SystemOccurence inclusion. |

    fill\_with\_hits(*hits*, *include\_forbidden*)[source]¶
    :   Adds hits to a system occurence, and check what are their status according to the system definition.
        Set the system occurence state to “no\_decision” after calling of this function.

        Note

        Forbidden genes will only be included if they do belong to the current system (and not to another specified with “system\_ref” in the current system’s definition).

        |  |  |
        | --- | --- |
        | Parameters: | **hits** – a list of Hits to treat for macsypy.search\_systems.SystemOccurence inclusion. |

    fill\_with\_multi\_systems\_genes(*multi\_systems\_hits*)[source]¶
    :   This function fills the SystemOccurrence with genes putatively coming from other systems (feature “multi\_system”).
        Those genes are used only if the occurrence of the corresponding gene was not yet filled with a gene from a cluster of the system.

        |  |  |
        | --- | --- |
        | Parameters: | **multi\_systems\_hits** – a list of hits of genes that are “multi\_system” which correspond to mandatory or accessory genes from the current system for which to fill a SystemOccurrence |

    get\_gene\_counter\_output(*forbid\_exclude=False*)[source]¶
    :   |  |  |
        | --- | --- |
        | Parameters: | **forbid\_exclude** (*boolean*) – exclude the forbidden components if set to True. False by default. |
        | Returns: | A dictionary ready for printing in system summary, with genes (mandatory, accessory and forbidden if specified) occurences in the system occurrence. |

    get\_gene\_ref(*gene*)[source]¶
    :   |  |  |
        | --- | --- |
        | Parameters: | **gene** (macsypy.gene.Gene, or macsypy.gene.Homolog or macsypy.gene.Analog object) – the gene to get it’s gene reference |
        | Returns: | object macsypy.gene.Gene or None |
        | Return type: | macsypy.gene.Gene object or None |
        | Raise : | KeyError if the system does not contain any gene gene. |

    get\_summary(*replicon\_name*, *rep\_info*)[source]¶
    :   Gives a summary of the system occurrence in terms of gene content and localization.

        |  |  |
        | --- | --- |
        | Parameters: | - **replicon\_name** (*string*) – the name of the replicon - **rep\_info** (a namedTuple “RepliconInfo” macsypy.database.RepliconInfo) – an entry extracted from the macsypy.database.RepliconDB |
        | Returns: | a tabulated summary of the macsypy.search\_systems.SystemOccurence |
        | Return type: | string |

    get\_summary\_header()[source]¶
    :   Returns a string with the description of the summary returned by self.get\_summary()

        |  |  |
        | --- | --- |
        | Return type: | string |

    get\_summary\_unordered(*replicon\_name*)[source]¶
    :   Gives a summary of the system occurrence in terms of gene content only (specific of “unordered” datasets).

        |  |  |
        | --- | --- |
        | Parameters: | **replicon\_name** (*string*) – the name of the replicon |
        | Returns: | a tabulated summary of the macsypy.search\_systems.SystemOccurence |
        | Return type: | string |

    get\_system\_name\_unordered(*suffix='\_putative'*)[source]¶
    :   Attributes a name to the system occurrence for an “unordered” dataset => generating a generic name based on the system name and the suffix given.

        |  |  |
        | --- | --- |
        | Parameters: | **suffix** (*string*) – the suffix to be used for generating the systemOccurrence’s name |
        | Returns: | a name for a system in an “unordered” dataset to the macsypy.search\_systems.SystemOccurence |
        | Return type: | string |

    get\_system\_unique\_name(*replicon\_name*)[source]¶
    :   Attributes unique name to the system occurrence with the class macsypy.search\_systems.SystemNameGenerator.
        Generates the name if not already set.

        |  |  |
        | --- | --- |
        | Parameters: | **replicon\_name** (*string*) – the name of the replicon |
        | Returns: | the unique name of the macsypy.search\_systems.SystemOccurence |
        | Return type: | string |

    is\_complete()[source]¶
    :   Test for SystemOccurrence completeness.

        |  |  |
        | --- | --- |
        | Returns: | True if the state of the SystemOccurrence is “single\_locus” or “multi\_loci”, False otherwise. |
        | Return type: | boolean |

    nb\_syst\_genes[source]¶
    :   This value is set after a decision was made on the system in macsypy.search\_systems.SystemOccurence:decision\_rule()

        |  |  |
        | --- | --- |
        | Returns: | the number of mandatory and accessory genes with at least one occurence (number of different accessory genes) |
        | Return type: | integer |

    state[source]¶
    :   |  |  |
        | --- | --- |
        | Returns: | the state of the systemOccurrence. |
        | Return type: | string |

macsypy.search\_systems.analyze\_clusters\_replicon(*clusters*, *systems*, *multi\_systems\_genes*)[source]¶
:   Analyzes sets of contiguous hits (clusters) stored in a ClustersHandler for system detection:

    - split clusters if needed
    - delete them if they are not relevant
    - add eventual genes from other systems “multi\_system” genes
    - check the QUORUM for each system to detect, *i.e.* mandatory + accessory - forbidden

    Only for “ordered” datasets representing a whole replicon.
    Reports systems occurence.

    |  |  |
    | --- | --- |
    | Parameters: | - **clusters** (macsypy.search\_systems.ClustersHandler) – the set of clusters to analyze - **systems** (a list of macsypy.system.System) – the set of systems to detect - **multi\_systems\_genes** – a dictionary with genes that could belong to multiple systems (keys are system names) |
    | Returns: | a set of systems occurence filled with hits found in clusters |
    | Return type: | a list of macsypy.search\_systems.SystemOccurence |

macsypy.search\_systems.build\_clusters(*hits*, *systems\_to\_detect*, *rep\_info*)[source]¶
:   Gets sets of contiguous hits according to the minimal inter\_gene\_max\_space between two genes. Only for “ordered” datasets.

    |  |  |
    | --- | --- |
    | Parameters: | - **hits** (a list of macsypy.report.Hit) – a list of Hmmer hits to analyze - **systems\_to\_detect** (a list of macsypy.system.System) – the list of systems to detect - **cfg** (macsypy.config.Config) – the configuration object built from default and user parameters. - **rep\_info** (a namedTuple “RepliconInfo” macsypy.database.RepliconInfo) – an entry extracted from the macsypy.database.RepliconDB |
    | Returns: | a set of clusters and a dictionary with “multi\_system” genes stored in a system-wise way for further utilization. |
    | Return type: | macsypy.search\_systems.ClustersHandler |

macsypy.search\_systems.disambiguate\_cluster(*cluster*)[source]¶
:   This disambiguation step is used on clusters with hits for multiple systems (when cluster.state is set to “ambiguous”).
    It returns a “cleansed” list of clusters, ready to use for system occurence detection (and that are “clear” cases). It:

    - splits the cluster in two if it seems that two systems are nearby
    - removes single hits that are not forbidden for the “main” system and that are at one end of the current cluster in this case, check that they are not “loners”, cause “loners” can be stored.

    |  |  |
    | --- | --- |
    | Parameters: | **cluster** (macsypy.search\_systems.Cluster) – the cluster to “disambiguate” |

macsypy.search\_systems.get\_best\_hits(*hits*, *tosort=False*, *criterion='score'*)[source]¶
:   Returns from a putatively redundant list of hits, a list of best matching hits.
    Analyzes quorum and co-localization if required for system detection.
    By default, hits are already sorted by position, and the hit with the best score is kept, then the best i-evalue. Possible criteria are:

    - maximal score (criterion=”score”)
    - minimal i-evalue (criterion=”i\_eval”)
    - maximal percentage of the profile covered by the alignment with the query sequence (criterion=”profile\_coverage”)

    |  |  |
    | --- | --- |
    | Parameters: | - **tosort** (*boolean*) – tells if the hits have to be sorted - **criterion** (*string*) – the criterion to base the sorting on |
    | Returns: | the list of best matching hits |
    | Return type: | list of macsypy.report.Hit |
    | Raise : | a macsypy.macsypy\_error.MacsypyError |

macsypy.search\_systems.get\_compatible\_systems(*systems\_list1*, *systems\_list2*)[source]¶
:   Returns the intersection of the two input systems lists.

    |  |  |
    | --- | --- |
    | Parameters: | **systems\_list2** (*systems\_list1,*) – two lists of systems |
    | Returns: | a list of systems, or an empty list if no common system |
    | Return type: | a list of macsypy.system.System |

macsypy.search\_systems.search\_systems(*hits*, *systems*, *cfg*)[source]¶
:   Runs search of systems from a set of hits. Criteria for system assessment will depend on the kind of input dataset provided:

    > - analyze **quorum and co-localization** for “ordered\_replicon” and “gembase” datasets.
    > - analyze **quorum only** (and in a limited way) for “unordered\_replicon” and “unordered” datasets.

    |  |  |
    | --- | --- |
    | Parameters: | - **hits** (list of macsypy.report.Hit) – the list of hits for input systems components - **systems** (list of macsypy.system.System) – the list of systems asked for detection - **cfg** (macsypy.config.Config) – the configuration object |

*class* macsypy.search\_systems.systemDetectionReportOrdered(*replicon\_name*, *systems\_occurences\_list*, *cfg*)[source]¶
:   Stores the detected systems to report for each replicon:
    :   - by system name,
        - by state of the systems (single vs multi loci)

    \_\_init\_\_(*replicon\_name*, *systems\_occurences\_list*, *cfg*)[source]¶
    :   |  |  |
        | --- | --- |
        | Parameters: | - **replicon\_name** (*string*) – the name of the replicon - **systems\_occurences\_list** (list of macsypy.search\_systems.SystemOccurence) – the list of system’s occurrences to consider |

    \_match2json(*valid\_hit*, *so*)[source]¶
    :   |  |  |
        | --- | --- |
        | Parameters: | - **valid\_hit** (class:macsypy.search\_system.ValidHit object.) – the valid hit to transform in to json. - **so** (class:macsypy.search\_system.SystemOccurence.) – the system occurence where the valid hit come from. |

    counter\_output()[source]¶
    :   Builds a counter of systems per replicon, with different “states” separated (single-locus vs multi-loci systems)

        |  |  |
        | --- | --- |
        | Returns: | the counter of systems |
        | Return type: | Counter |

    json\_output(*json\_path*, *json\_data*)[source]¶

    report\_output(*reportfilename*, *print\_header=False*)[source]¶
    :   Writes a report of sequences forming the detected systems, with information in their status in the system,
        their localization on replicons, and statistics on the Hits.

        |  |  |
        | --- | --- |
        | Parameters: | - **reportfilename** (*string*) – the output file name - **print\_header** (*boolean*) – True if the header has to be written. False otherwise |

    summary\_output(*reportfilename*, *rep\_info*, *print\_header=False*)[source]¶
    :   Writes a report with the summary of systems detected in replicons. For each system, a summary is done including:

        > - the number of mandatory/accessory genes in the reference system (as defined in XML files)
        > - the number of mandatory/accessory genes detected
        > - the number and list of missing genes
        > - the number of loci encoding the system

        |  |  |
        | --- | --- |
        | Parameters: | - **rep\_info** (a namedTuple “RepliconInfo” macsypy.database.RepliconInfo) – an entry extracted from the macsypy.database.RepliconDB - **print\_header** (*boolean*) – True if the header has to be written. False otherwise |

    system\_2\_json(*rep\_db*)[source]¶
    :   Generates the report in json format

        |  |  |
        | --- | --- |
        | Parameters: | - **path** (*string*) – the path to a file where to write the report in json format - **rep\_db** (a class:macsypy.database.RepliconDB object) – the replicon database |

    tabulated\_output(*system\_occurence\_states*, *system\_names*, *reportfilename*, *print\_header=False*)[source]¶
    :   Write a tabulated output with number of detected systems for each replicon.

        |  |  |
        | --- | --- |
        | Parameters: | - **system\_occurence\_states** (*list of string*) – the different forms of detected systems to consider - **reportfilename** (*string*) – the output file name - **print\_header** (*boolean*) – True if the header has to be written. False otherwise |
        | Return type: | string |

    tabulated\_output\_header(*system\_occurence\_states*, *system\_names*)[source]¶
    :   Returns a string containing the header of the tabulated output

        |  |  |
        | --- | --- |
        | Parameters: | **system\_occurence\_states** (*list of string*) – the different forms of detected systems to consider |
        | Return type: | string |

*class* macsypy.search\_systems.systemDetectionReportUnordered(*systems\_occurences\_list*, *cfg*)[source]¶
:   Stores a report for putative detected systems gathering all hits from a search in an unordered dataset:
    :   - by system.

    Mandatory and accessory genes only are reported in the “json” and “report” output, but all hits matching a system component are reported in the “summary”.

    \_\_init\_\_(*systems\_occurences\_list*, *cfg*)[source]¶
    :   |  |  |
        | --- | --- |
        | Parameters: | **systems\_occurences\_list** (list of macsypy.search\_systems.SystemOccurence) – the list of system’s occurrences to consider |

    json\_output(*json\_path*)[source]¶
    :   Generates the report in json format

        |  |  |
        | --- | --- |
        | Parameters: | **path** (*string*) – the path to a file where to write the report in json format |

    report\_output(*reportfilename*, *print\_header=False*)[source]¶
    :   Writes a report of sequences forming the detected systems, with information in their status in the system,
        their localization on replicons, and statistics on the Hits.

        |  |  |
        | --- | --- |
        | Parameters: | - **reportfilename** (*string*) – the output file name - **print\_header** (*boolean*) – True if the header has to be written. False otherwise |

    summary\_output(*reportfilename*, *print\_header=False*)[source]¶
    :   Writes a report with the summary for putative systems in an unordered dataset. For each system, a summary is done including:

        > - the number of mandatory/accessory genes in the reference system (as defined in XML files)
        > - the number of mandatory/accessory genes detected

        |  |  |
        | --- | --- |
        | Parameters: | - **reportfilename** (*string*) – the output file name - **print\_header** (*boolean*) – True if the header has to be written. False otherwise |

*class* macsypy.search\_systems.validSystemHit(*hit*, *detected\_system*, *gene\_status*)[source]¶
:   Encapsulates a macsypy.report.Hit
    This class stores a Hit that has been attributed to a detected system. Thus, it also stores:

    - the system,
    - the status of the gene in this system,

    It also aims at storing information for results extraction:

    - system extraction (e.g. genomic positions)
    - sequence extraction

    \_\_init\_\_(*hit*, *detected\_system*, *gene\_status*)[source]¶
    :   |  |  |
        | --- | --- |
        | Parameters: | - **hit** (macsypy.report.Hit) – a hit to base the validSystemHit on - **detected\_system** (*string*) – the name of the predicted System - **gene\_status** (*string*) – the “role” of the gene in the predicted system |

    \_\_weakref\_\_¶
    :   list of weak references to the object (if defined)

    output\_system\_header()[source]¶
    :   |  |  |
        | --- | --- |
        | Returns: | the header for the output file |
        | Return type: | string |

#### Previous topic

search\_genes API reference

#### Next topic

MacSyFinder errors

### This Page

- Show Source

### Quick search


Enter search terms or a module, class or function name.

### Navigation

- index
- modules |
- next |
- previous |
- MacSyFinder documentation »

© Copyright 2014, Sophie Abby, Bertrand Néron.
Created using Sphinx 1.1.3.
