# Supplementary material for: MacSyFinder: A Program to Mine Genomes for Molecular Systems with an Application to CRISPR-Cas Systems
Source: PLoS One. 2014 Oct 17;9(10):e110726. doi: 10.1371/journal.pone.0110726 (PMC4201578; doi:10.1371/journal.pone.0110726)
Supplement: Data S1 — The MacSyFinder/MacSyView package (compressed tarball archive). (GZ) [file pone.0110726.s010.gz › macsyfinder-1.0.0-RC3/doc/_build/html/system.html]

System API — MacSyFinder 1.0.0-RC3 documentation


### Navigation

- index
- modules |
- next |
- previous |
- MacSyFinder documentation »

# System API¶

It represents a macromolecular system to detect. See *The System object* for an overview of its implementation.

Note

a complete description of macromolecular systems modelling is available in the section *Macromolecular systems definition*

## SystemBank API reference¶

> *class* macsypy.system.SystemBank[source]¶
> :   Build and store all Systems objects. Systems must not be instanciated directly.
>     This system factory must be used. It ensures there is a unique instance
>     of a system for a given system name.
>     To get a system, use the method \_\_getitem\_\_ via the “[]”. If the System is already cached in the SystemBank, it is returned.
>     Otherwise a new system is built, stored and then returned.
>
>     \_\_contains\_\_(*system*)[source]¶
>     :   Implement the membership test operator
>
>         |  |  |
>         | --- | --- |
>         | Parameters: | **system** (macsypy.system.System object) – the system to test |
>         | Returns: | True if the system is in the System factory, False otherwise |
>         | Return type: | boolean |
>
>     \_\_getitem\_\_(*name*)[source]¶
>     :   |  |  |
>         | --- | --- |
>         | Parameters: | **name** (*string*) – the name of the system |
>         | Returns: | the system corresponding to the name. If the system already exists, return it, otherwise build it and return it. |
>         | Return type: | macsypy.system.System object |
>
>     \_\_iter\_\_()[source]¶
>     :   Return an iterator object on the systems contained in the bank
>
>     \_\_len\_\_()[source]¶
>     :   |  |  |
>         | --- | --- |
>         | Returns: | the number of systems stored in the bank |
>         | Return type: | integer |
>
>     \_\_weakref\_\_¶
>     :   list of weak references to the object (if defined)
>
>     add\_system(*system*)[source]¶
>     :   |  |  |
>         | --- | --- |
>         | Parameters: | **system** (macsypy.system.System object) – the system to add |
>         | Raise : | KeyError if a system with the same name is already registered. |

Note

Don’t instanciate your own SystemBank use the system\_bank at the top level of the module.

```
from macsypy.system import system_bank
```

## System API reference¶

*class* macsypy.system.System(*cfg*, *name*, *inter\_gene\_max\_space*, *min\_mandatory\_genes\_required=None*, *min\_genes\_required=None*, *max\_nb\_genes=None*, *multi\_loci=False*)[source]¶
:   Handle a secretion system.

    \_\_init\_\_(*cfg*, *name*, *inter\_gene\_max\_space*, *min\_mandatory\_genes\_required=None*, *min\_genes\_required=None*, *max\_nb\_genes=None*, *multi\_loci=False*)[source]¶
    :   |  |  |
        | --- | --- |
        | Parameters: | - **cfg** (macsypy.config.Config object) – the configuration object - **name** (*string*) – the name of the system - **inter\_gene\_max\_space** (*integer*) – the maximum distance between two genes (**co-localization** parameter) - **min\_mandatory\_genes\_required** (*integer*) – the quorum of mandatory genes to define this system - **min\_genes\_required** (*integer*) – the quorum of genes to define this system - **max\_nb\_genes** (*integer*) – - **multi\_loci** (*boolean*) – |

    \_\_weakref\_\_¶
    :   list of weak references to the object (if defined)

    accessory\_genes[source]¶
    :   |  |  |
        | --- | --- |
        | Returns: | the list of genes that are allowed in this secretion system |
        | Return type: | list of macsypy.gene.Gene objects |

    add\_accessory\_gene(*gene*)[source]¶
    :   Add a gene to the list of accessory genes

        |  |  |
        | --- | --- |
        | Parameters: | **gene** (macsypy.gene.Gene object) – gene that is allowed to be present in this system |

    add\_forbidden\_gene(*gene*)[source]¶
    :   Add a gene to the list of forbidden genes

        |  |  |
        | --- | --- |
        | Parameters: | **gene** (macsypy.genen.Gene object) – gene that must not be found in this system |

    add\_mandatory\_gene(*gene*)[source]¶
    :   Add a gene to the list of mandatory genes

        |  |  |
        | --- | --- |
        | Parameters: | **gene** (macsypy.gene.Gene object) – gene that is mandatory for this system |

    forbidden\_genes[source]¶
    :   |  |  |
        | --- | --- |
        | Returns: | the list of genes that are forbidden in this secretion system |
        | Return type: | list of macsypy.gene.Gene objects |

    get\_gene(*gene\_name*)[source]¶
    :   |  |  |
        | --- | --- |
        | Parameters: | **gene\_name** (*string*) – the name of the gene to get |
        | Returns: | the gene corresponding to gene\_name. |
        | Return type: | a macsypy.gene.Gene object. |
        | Raise : | KeyError the system does not contain any gene with name gene\_name. |

    get\_gene\_ref(*gene*)[source]¶
    :   |  |  |
        | --- | --- |
        | Parameters: | **gene** (a macsypy.gene.Gene or macsypy.gene.Homolog` or macsypy.gene.Analog` object.) – the gene to get the gene reference. |
        | Returns: | the gene reference of the gene if exists (if the gene is an Homolog or an Analog), otherwise return None. |
        | Return type: | macsypy.gene.Gene object or None |
        | Raise : | KeyError if gene is not in the system |

    inter\_gene\_max\_space[source]¶
    :   |  |  |
        | --- | --- |
        | Returns: | set the maximum distance allowed between 2 genes for this system |
        | Return type: | integer |

    mandatory\_genes[source]¶
    :   |  |  |
        | --- | --- |
        | Returns: | the list of genes that are mandatory in this secretion system |
        | Return type: | list of macsypy.gene.Gene objects |

    max\_nb\_genes[source]¶
    :   |  |  |
        | --- | --- |
        | Returns: | the maximum number of genes to assess the system presence. |
        | Return type: | int (or None) |

    min\_genes\_required[source]¶
    :   |  |  |
        | --- | --- |
        | Returns: | get the minimum number of genes to assess for the system presence. |
        | Return type: | integer |

    min\_mandatory\_genes\_required[source]¶
    :   |  |  |
        | --- | --- |
        | Returns: | get the quorum of mandatory genes required for this system |
        | Return type: | integer |

    multi\_loci[source]¶
    :   |  |  |
        | --- | --- |
        | Returns: | True if the system is authorized to be inferred from multiple loci, False otherwise |
        | Return type: | boolean |

### Table Of Contents

- System API
  - SystemBank API reference
  - System API reference

#### Previous topic

Database API

#### Next topic

The Parser of Systems definitions

### This Page

- Show Source

### Quick search


Enter search terms or a module, class or function name.

### Navigation

- index
- modules |
- next |
- previous |
- MacSyFinder documentation »

© Copyright 2014, Sophie Abby, Bertrand Néron.
Created using Sphinx 1.1.3.
