# Supplementary material for: MacSyFinder: A Program to Mine Genomes for Molecular Systems with an Application to CRISPR-Cas Systems
Source: PLoS One. 2014 Oct 17;9(10):e110726. doi: 10.1371/journal.pone.0110726 (PMC4201578; doi:10.1371/journal.pone.0110726)
Supplement: Data S1 — The MacSyFinder/MacSyView package (compressed tarball archive). (GZ) [file pone.0110726.s010.gz › macsyfinder-1.0.0-RC3/doc/_build/html/system_definition.html]

Macromolecular systems definition — MacSyFinder 1.0.0-RC3 documentation


### Navigation

- index
- modules |
- next |
- previous |
- MacSyFinder documentation »

# Macromolecular systems definition¶

## Principles¶

MacSyFinder relies on the definition of models of macromolecular systems with an **XML grammar** that is described *below*.

A system is defined in a dedicated file named after the system (*e.g.*, ‘T1SS.xml’ for T1SS, the Type 1 Secretion System) by a set of **components** (*i.e.* proteins, or protein-coding genes given the context) with different attributes and that are used for **content description**. Some components are specific to the system, and some are possibly from other systems. In the latter case, the full description of the gene with its attributes must be defined in the XML file of the original system.
Features regarding **co-localization** parameters for system detection are also defined in this system-specific file.

Three distinct types of components can be used to model a given system content, and which corresponds to Gene objects, and the corresponding HMM protein profiles.

- **Mandatory** components represent essential components to be found to infer the System presence.
- **Accessory** components correspond to components that can be found in some systems occurrence, or fastly evolving components that are hard to detect with a single profile.
- **Forbidden** components are components which presence is eliminatory for the System assessment.

## The XML hierarchy¶

- The element root is “system”.

  - It has a mandatory attribute: “inter\_gene\_max\_space”, an integer representing the maximal number of components without a match between two components with a match for a component profile.
  - The element “system” may have attributes:

    > - “min\_mandatory\_genes\_required”: an integer representing the minimal number of mandatory genes required to infer the system presence.
    > - “min\_genes\_required”: an integer representing the minimal number of mandatory or accessory genes (whose corresponding proteins match a profile of the system) required to infer the system presence.
    > - “max\_nb\_genes”: an integer representing the maximal number of mandatory or accessory genes in the system.
    > - “multi\_loci”: a boolean set to True (“1”, “true” or “True”) to allow the definition of “scattered” systems (systems encoded by different loci). If not specified, *default value is false*.
  - The system contains one or more element “gene”.
- The element “gene” has several mandatory attributes:

  > - “name”: which must match to a profile in the profile directory.
  > - “presence”: which can take three values “mandatory”, “accessory”, “forbidden”.

> The element “gene” may have other attributes:
>
> > - “system\_ref”: which is a reference to the macromolecular system from where the gene comes from (this attribute is used for forbidden gene and homologs gene). If system\_ref is not specified, it means the gene is from the current system.
> > - “loner”: which is a boolean. If a gene is loner that means this gene can be isolated on the genome ( *default false* ).
> > - “exchangeable”: which is a boolean. If a gene is exchangeable (value set to “1”, “true” or “True”) that means this gene or one of its homologs or analogs can be interchanged for the assessment of the presence of the macromolecular system ( *default false* ).
> > - “multi\_system”: which is a boolean. If a gene is “multi\_system” (value set to “1”, “true” or “True”), it means that it can be used to fill by multiple systems occurrences. ( *default false* ).
> > - “aligned”: which is a boolean (this attribute is used only for homologs).
> > - “inter\_gene\_max\_space”: an integer that defines gene-wise value of system’s “inter\_gene\_max\_space” parameter (see above).
> > - an element “homologs” that contains a list of homologous genes that can potentially match the same sequences. They can potentially be functionally equivalent to the reference gene if it was declared “exchangeable”
> > - an element “analogs” that contains a list of analogous genes that can potentially be functionally equivalent, if the parent gene was declared “exchangeable”.

- The elements “homologs” and “analogs” can contain one or more element “gene”.

Example of a system definition in XML:

```
<system inter_gene_max_space="5">
  <gene name="gspD" presence="mandatory">
     <homologs>
         <gene name="sctC" system_ref="T3SS"/>
     </homologs>
  </gene>
  <gene name="sctN_FLG" presence="mandatory" loner="1" exchangeable="1"/>
     <analogs>
         <gene name="gspE" system_ref="T2SS"/>
         <gene name="pilT" system_ref="T4P"/>
     </analogs>
  <gene name="sctV_FLG" presence="mandatory"/>
  <gene name="flp" presence="accessory"/>
</system>
```

Warning

- a gene is identified by its name.
- a gene can be defined only **once** in all systems.
- other occurrences of this gene must be specified as references (using the “system\_ref” attribute to specify what is the native system).
- if a gene is specified with the attribute “system\_ref”, it means it has been (or has to be) defined in the system specified by “system\_ref”.
- if a gene is not specified with the attribute “system\_ref”, it means it belongs to the current system, where it has to be defined with all its features.

### Table Of Contents

- Macromolecular systems definition
  - Principles
  - The XML hierarchy

#### Previous topic

MacSyView: visualizing MacSyFinder’s results!

#### Next topic

MacSyFinder implementation overview

### This Page

- Show Source

### Quick search


Enter search terms or a module, class or function name.

### Navigation

- index
- modules |
- next |
- previous |
- MacSyFinder documentation »

© Copyright 2014, Sophie Abby, Bertrand Néron.
Created using Sphinx 1.1.3.
