# Supplementary material for: MacSyFinder: A Program to Mine Genomes for Molecular Systems with an Application to CRISPR-Cas Systems
Source: PLoS One. 2014 Oct 17;9(10):e110726. doi: 10.1371/journal.pone.0110726 (PMC4201578; doi:10.1371/journal.pone.0110726)
Supplement: Data S1 — The MacSyFinder/MacSyView package (compressed tarball archive). (GZ) [file pone.0110726.s010.gz › macsyfinder-1.0.0-RC3/doc/_build/html/system_parser.html]

The Parser of Systems definitions — MacSyFinder 1.0.0-RC3 documentation


### Navigation

- index
- modules |
- next |
- previous |
- MacSyFinder documentation »

# The Parser of Systems definitions¶

The system parser object “SystemParser” instanciates Systems and Genes objects from XML system definitions (see *Macromolecular systems definition*).
The parsing consists in three phases.

Phase 1.

> - each Gene is parsed from the System it is defined
> - From the list of System to detect, the list of Systems to parse is established

Phase 2.

> - For each system to parse
>   - create the System
>   - add this System to the system\_bank
>   - create the Genes defined in this System with their attributes but not their Homologs
>   - add these Genes in the gene\_bank

Phase 3.

> - For each System to search
>
>   - For each Gene defined in this System:
>
>     > - create the Homologs by encapsulating Genes from the gene\_bank
>     > - add the Gene to the System

For instance:

```
Syst_1
<system inter_gene_max_space="10">
    <gene name=”A” mandatory=”1” loner="1">
        <homologs>
            <gene name=”B” sys_ref=”Syst_2”>
        </homologs>
    </gene>
<system>

Syst_2
<system inter_gene_max_space="15">
    <gene name=”B” mandatory=”1”>
        <homologs>
            <gene name=”B” sys_ref=”Syst_1”
            <gene name=”C” sys_ref=”Syst_3”>
        </homologs>
    </gene>
<system>

Syst_3
<system inter_gene_max_space="20">
    <gene name=”c” mandatory=”1” />
<system>
```

With the example above:

- the Syst\_1 has a gene\_A
- the gene\_A has homolog gene\_B
- the gene\_B has a reference to Syst\_2
- gene\_B attributes from the Syst\_2 are used to build the Gene
- the Syst\_2 has attributes as defined in the corresponding XML file (inter\_gene\_max\_space ,...)

Contrariwise:

- the gene\_B has no Homologs
- the Syst\_2 has no Genes

Note

The only “full” Systems (*i.e.,* with all corresponding Genes created) are those to detect.

## SystemParser API reference¶

*class* macsypy.system\_parser.SystemParser(*cfg*, *system\_bank*, *gene\_bank*)[source]¶
:   Build a System instance from the corresponding System definition described in the XML file (named after the system’s name) found at the dedicated location (“-d” command-line option).

    \_\_init\_\_(*cfg*, *system\_bank*, *gene\_bank*)[source]¶
    :   Constructor

        |  |  |
        | --- | --- |
        | Parameters: | - **cfg** (macsypy.config.Config object) – the configuration object of this run - **system\_bank** (macsypy.system.SystemBank object) – the system factory - **gene\_bank** (macsypy.gene.GeneBank object) – the gene factory |

    \_\_weakref\_\_¶
    :   list of weak references to the object (if defined)

    \_create\_genes(*system*, *system\_node*)[source]¶
    :   Create genes belonging to the systems. Be careful, the returned genes have not their homologs/analogs set yet.

        |  |  |
        | --- | --- |
        | Parameters: | - **system** (macsypy.system.System object) – the System currently parsing - **system\_node** (:class”ET.ElementTree object) – the element gene |
        | Returns: | a list of the genes belonging to the system. |
        | Return type: | [macsypy.gene.Gene, ...] |

    \_create\_system(*system\_name*, *system\_node*)[source]¶
    :   |  |  |
        | --- | --- |
        | Parameters: | - **system\_name** – the name of the system to create. This name must match a XML file in the definition directory (“-d” option in the command-line) - **system\_node** (:class”ET.ElementTree object.) – the node corresponding to the system. |
        | Returns: | the system corresponding to the name. |
        | Return type: | macsypy.system.System object. |

    \_fill(*system*, *system\_node*)[source]¶
    :   Fill the system with genes found in this system definition. Add homologs to the genes if necessary.

        |  |  |
        | --- | --- |
        | Parameters: | - **system** (macsypy.system.System object) – the system to fill - **system\_node** (:class”ET.ElementTree object) – the “node” in the XML hierarchy corresponding to the system |

    \_parse\_analog(*node*, *gene\_ref*, *curr\_system*)[source]¶
    :   Parse a xml element gene and build the corresponding object

        |  |  |
        | --- | --- |
        | Parameters: | - **node** (xml.etree.ElementTree.Element object.) – a “node” corresponding to the gene element in the XML hierarchy - **gene\_ref** (class:macsypy.gene.Gene object.) – the gene which this gene is homolog to |
        | Returns: | the gene object corresponding to the node |
        | Return type: | macsypy.gene.Analog object |

    \_parse\_homolog(*node*, *gene\_ref*, *curr\_system*)[source]¶
    :   Parse a xml element gene and build the corresponding object

        |  |  |
        | --- | --- |
        | Parameters: | - **node** (xml.etree.ElementTree.Element object.) – a “node” corresponding to the gene element in the XML hierarchy - **gene\_ref** (class:macsypy.gene.Gene object) – the gene which this gene is homolog to |
        | Returns: | the gene object corresponding to the node |
        | Return type: | macsypy.gene.Homolog object |

    check\_consistency(*systems*)[source]¶
    :   Check the consistency of the co-localization features between the different values given as an input:
        between XML definitions, configuration file, and command-line options.

        |  |  |
        | --- | --- |
        | Parameters: | **systems** (list of class:txssnalib.system.System object) – the list of systems to check |
        | Raise : | macsypy.macsypy\_error.SystemInconsistencyError if one test fails |

        (see feature)

        In the different possible situations, different requirements need to be fulfilled (“mandatory\_genes” and “accessory\_genes” consist of lists of genes defined as such in the system definition):

        > - **If:** min\_mandatory\_genes\_required = None ; min\_genes\_required = None
        > - **Then:** min\_mandatory\_genes\_required = min\_genes\_required = len(mandatory\_genes)
        >
        > *always True by Systems design*
        >
        > - **If:** min\_mandatory\_genes\_required = value ; min\_genes\_required = None
        > - **Then:** min\_mandatory\_genes\_required <= len(mandatory\_genes)
        > - AND min\_genes\_required = min\_mandatory\_genes\_required
        >
        > *always True by design*
        >
        > - **If:** min\_mandatory\_genes\_required = None ; min\_genes\_required = Value
        > - **Then:** min\_mandatory\_genes\_required = len(mandatory\_genes)
        > - AND min\_genes\_required >= min\_mandatory\_genes\_required
        > - AND min\_genes\_required <= len(mandatory\_genes+accessory\_genes)
        >
        > *to be checked*
        >
        > - **If:** min\_mandatory\_genes\_required = Value ; min\_genes\_required = Value
        > - **Then:** min\_genes\_required <= len(accessory\_genes+mandatory\_genes)
        > - AND min\_genes\_required >= min\_mandatory\_genes\_required
        > - AND min\_mandatory\_genes\_required <= len(mandatory\_genes)
        >
        > *to be checked*

    parse(*systems\_2\_detect*)[source]¶
    :   Parse systems definition in XML format to build the corresponding system objects, and add them to the system factory after checking its consistency.
        To get the system ask it to system\_bank
        :param systems\_2\_detect: a list with the names of the systems to parse
        :type systems\_2\_detect: list of string

    system\_to\_parse(*sys\_2\_parse*, *parsed\_systems*)[source]¶
    :   |  |  |
        | --- | --- |
        | Parameters: | **sys\_2\_parse** (*[string, ...]*) – the list of systems to parse |
        | Returns: | the list of systems’ names to parse. Scan the whole chain of ‘system\_ref’ in a recursive way. |
        | Return type: | [string, ...] |

### Table Of Contents

- The Parser of Systems definitions
  - SystemParser API reference

#### Previous topic

System API

#### Next topic

Gene API

### This Page

- Show Source

### Quick search


Enter search terms or a module, class or function name.

### Navigation

- index
- modules |
- next |
- previous |
- MacSyFinder documentation »

© Copyright 2014, Sophie Abby, Bertrand Néron.
Created using Sphinx 1.1.3.
