# Supplementary material for: MacSyFinder: A Program to Mine Genomes for Molecular Systems with an Application to CRISPR-Cas Systems
Source: PLoS One. 2014 Oct 17;9(10):e110726. doi: 10.1371/journal.pone.0110726 (PMC4201578; doi:10.1371/journal.pone.0110726)
Supplement: Data S1 — The MacSyFinder/MacSyView package (compressed tarball archive). (GZ) [file pone.0110726.s010.gz › macsyfinder-1.0.0-RC3/doc/_build/html/_modules/macsypy/config.html]

macsypy.config — MacSyFinder 1.0.0-RC3 documentation


### Navigation

- index
- modules |
- MacSyFinder documentation »
- Module code »

# Source code for macsypy.config

```
# -*- coding: utf-8 -*-

################################################################################
# MacSyFinder - Detection of macromolecular systems in protein datasets        #
#               using systems modelling and similarity search.                 #
# Authors: Sophie Abby, Bertrand Néron                                         #
# Copyright © 2014  Institut Pasteur, Paris.                                   #
# See the COPYRIGHT file for details                                           #
#                                                                              #
# MacsyFinder is distributed under the terms of the GNU General Public License #
# (GPLv3). See the COPYING file for details.                                   #
################################################################################


import os
import sys
import inspect
from time import strftime
from ConfigParser import SafeConfigParser, NoSectionError, NoOptionError

_prefix_path = '$PREFIX'
_prefix_conf = '$PREFIXCONF'
_prefix_data = '$PREFIXDATA'
if 'MACSY_HOME' in os.environ and os.environ['MACSY_HOME']:
    _prefix_path = os.environ['MACSY_HOME']
    _prefix_conf = os.path.join(os.environ['MACSY_HOME'], 'etc')
    _prefix_data = os.path.join(os.environ['MACSY_HOME'], 'data')

import logging

[docs]class Config(object):
    """
    Parse configuration files and handle the configuration according to the following file location precedence:
    /etc/macsyfinder/macsyfinder.conf < ~/.macsyfinder/macsyfinder.conf < .macsyfinder.conf
    
    If a configuration file is given on the command-line, this file will be used.
    *In fine* the arguments passed on the command-line have the highest priority.
    """
    
    #if a new option is added think to add it also (if needed) in save
    options = ( 'cfg_file', 'previous_run', 'sequence_db', 'db_type', 'replicon_topology', 'topology_file', 
                'inter_gene_max_space', 'min_mandatory_genes_required', 'min_genes_required', 'max_nb_genes', 'multi_loci', 
                'hmmer_exe', 'index_db_exe', 'e_value_res', 'i_evalue_sel', 'coverage_profile', 
                'def_dir', 'res_search_dir', 'res_search_suffix', 'profile_dir', 'profile_suffix', 'res_extract_suffix', 
                'log_level', 'log_file', 'worker_nb', 'config_file', 'build_indexes')

[docs]    def __init__(self, cfg_file = "",
                sequence_db = None ,
                db_type = None,
                replicon_topology = None,
                topology_file = None,
                inter_gene_max_space = None,
                min_mandatory_genes_required = None,
                min_genes_required = None,
                max_nb_genes = None,
                multi_loci = None,
                hmmer_exe = None,
                index_db_exe = None,
                e_value_res = None,
                i_evalue_sel = None,
                coverage_profile = None,
                def_dir = None ,
                res_search_dir = None,
                res_search_suffix = None,
                profile_dir = None,
                profile_suffix = None,
                res_extract_suffix = None,
                log_level = None,
                log_file = None,
                worker_nb = None,
                config_file = None,
                previous_run = None,
                build_indexes = None
                ):
        """
        :param cfg_file: the path to the MacSyFinder configuration file to use 
        :type cfg_file: string
        :param previous_run: the path to the results directory of a previous run
        :type previous_run: string 
        :param sequence_db: the path to the sequence input dataset (fasta format)
        :type sequence_db: string
        :param db_type: the type of dataset to deal with. 
         \"unordered_replicon\" corresponds to a non-assembled genome, 
         \"unordered\" to a metagenomic dataset, 
         \"ordered_replicon\" to an assembled genome, and 
         \"gembase\" to a set of replicons where sequence identifiers follow this convention \">RepliconName_SequenceID\"."
        :type db_type: string
        :param replicon_topology: the topology ('linear' or 'circular') of the replicons. This option is meaningful only if the db_type is 'ordered_replicon' or 'gembase' 
        :type replicon_topology: string
        :param topology_file: a tabular file of mapping between replicon names and the corresponding topology (e.g. \"RepliconA linear\") 
        :type topology_file: string
        :param inter_gene_max_space:
        :type inter_gene_max_space: list of list of 2 elements [[ string system, integer space] , ...]
        :param min_mandatory_genes_required:
        :type min_mandatory_genes_required: list of list of 2 elements [[ string system, integer ] , ...]
        :param min_genes_required:
        :type min_genes_required: list of list of 2 elements [[ string system, integer ] , ...]
        :param max_nb_genes: 
        :type max_nb_genes: list of list of 2 elements [[ string system, integer ] , ...]
        :param multi_loci: 
        :type multi_loci: string
        :param hmmer_exe: the Hmmer \"hmmsearch\" executable
        :type hmmer_exe: string
        :param index_db_exe: the indexer executable (\"makeblastdb\" or \"formatdb\")
        :type index_db_exe: string
        :param e_value_res: maximal e-value for hits to be reported during Hmmer search
        :type  e_value_res: float
        :param i_evalue_sel: maximal independent e-value for Hmmer hits to be selected for system detection
        :type  i_evalue_sel: float
        :param coverage_profile: minimal profile coverage required in the hit alignment to allow the hit selection for system detection
        :type coverage_profile: float
        :param def_dir: the path to the directory containing systems definition files (.xml)
        :type def_dir: string
        :param res_search_dir: the path to the directory where to store MacSyFinder search results directories.
        :type  res_search_dir: string
        :param res_search_suffix: the suffix to give to Hmmer raw output files
        :type  res_search_suffix: string
        :param res_extract_suffix: the suffix to give to filtered hits output files
        :type  res_extract_suffix: string
        :param profile_dir: path to the profiles directory
        :type  profile_dir: string
        :param profile_suffix: the suffix of profile files. For each 'Gene' element, the corresponding profile is searched in the 'profile_dir', in a file which name is based on the Gene name + the profile suffix. 
        :type  profile_suffix: string
        :param log_level: the level of log output
        :type log_level: int
        :param log_file: the path to the directory to write MacSyFinder log files
        :type log_file: string
        :param worker_nb: maximal number of processes to be used in parallel (multi-thread run, 0 use all cores availables)
        :type worker_nb: int
        :param build_indexes: build the indexes from the sequence dataset in fasta format
        :type build_indexes: boolean
        """

        self._new_cfg_name = "macsyfinder.conf"
        if previous_run:
            prev_config = os.path.join(previous_run, self._new_cfg_name)
            if not os.path.exists(prev_config):
                raise ValueError("No config file found in dir %s" % previous_run)
            config_files = [prev_config]
        elif cfg_file:
            config_files = [cfg_file]
        else:
            config_files = [os.path.join( _prefix_conf, 'macsyfinder.conf'),
                           os.path.expanduser('~/.macsyfinder/macsyfinder.conf'),
                           'macsyfinder.conf']
        self._defaults = {'replicon_topology': 'circular',
                          'hmmer_exe' : 'hmmsearch',
                          'index_db_exe': 'makeblastdb',
                          'e_value_res' : "1",
                          'i_evalue_sel' : "0.001",
                          'coverage_profile' : "0.5",
                          'def_dir': os.path.join( _prefix_data, 'DEF'),
                          'res_search_dir' : os.getcwd() ,
                          'res_search_suffix' : '.search_hmm.out',
                          'res_extract_suffix' : '.res_hmm_extract',
                          'profile_dir' : os.path.join( _prefix_data, 'profiles'),
                          'profile_suffix' : '.hmm', 
                          'log_level': logging.WARNING,
                          'worker_nb' : '1'
                          }
        self.parser = SafeConfigParser(defaults = self._defaults)
        used_files = self.parser.read(config_files)

        frame = inspect.currentframe()
        args, _, _, values = inspect.getargvalues(frame)

        cmde_line_opt = {}
        for arg in args:
            if arg in self.options and values[arg] is not None:
                #the option in ConfigParser are store as string
                #so in save method I dump some options only if 
                #they are != than the default values in ConfigParser
                cmde_line_opt[arg] = str(values[arg])

        self.options = self._validate(cmde_line_opt, values)


[docs]    def _validate(self, cmde_line_opt, cmde_line_values):
        """
        Get all configuration values and check the validity of their values.
        Create the working directory

        :param cmde_line_opt: the options from the command line
        :type cmde_line_opt: dict, all values are cast in string
        :param cmde_line_values: the options from the command line
        :type cmde_line_values: dict, values are not cast
        :return: all the options for this execution
        :rtype: dictionary
        """  
        options = {}
        if 'sequence_db' in cmde_line_opt:
            cmde_line_opt['file'] = cmde_line_opt['sequence_db']

        try:
            options['res_search_dir'] = self.parser.get('directories', 'res_search_dir', vars = cmde_line_opt)
        except NoSectionError:
            if 'res_search_dir' in cmde_line_opt:
                options['res_search_dir'] = cmde_line_opt['res_search_dir']
            else:
                options['res_search_dir'] = self._defaults['res_search_dir']
        if not os.path.exists(options['res_search_dir']):
            raise ValueError( "%s: This results directory does not exist" % options['res_search_dir'])
        if not os.access(options['res_search_dir'], os.W_OK):
            raise ValueError("The results directory (%s) is not writable" % options['res_search_dir'])

        working_dir = os.path.join(options['res_search_dir'], "macsyfinder-" + strftime("%Y%m%d_%H-%M-%S"))
        try:
            os.mkdir(working_dir)
        except OSError, err:
            raise ValueError("cannot create MacSyFinder working directory %s : %s" % (working_dir, err))
        options['working_dir'] = working_dir

        hmmer_path = os.path.join(working_dir, self.hmmer_dir)
        try:
            os.mkdir(hmmer_path)
        except OSError, err:
            raise ValueError("cannot create MacSyFinder hmmer directory %s : %s" % (hmmer_path, err))

        try:
            log_level = self.parser.get('general', 'log_level', vars = cmde_line_opt)
        except (AttributeError, NoSectionError):
            log_level = self._defaults['log_level']
        else:
            try:
                log_level = int(log_level)
            except ValueError:
                try:
                    log_level = getattr(logging, log_level.upper())
                except AttributeError:
                    log_level = logging.ERROR
        options['log_level'] = log_level

        log_error = []
        try:
            log_file = self.parser.get('general', 'log_file', vars = cmde_line_opt)
            log_handler = logging.FileHandler(log_file)
            options['log_file'] = log_file
        except Exception , err:
            if not isinstance(err, NoOptionError):
                log_error.append(err)
            try:
                log_file = os.path.join( options['working_dir'], 'macsyfinder.log' )
                log_handler = logging.FileHandler(log_file)
                options['log_file'] = log_file
            except Exception , err:
                log_error.append(err)
                log_handler = logging.StreamHandler(sys.stderr)
                options['log_file'] = ''
        
        handler_formatter = logging.Formatter("%(levelname)-8s : %(filename)-10s : L %(lineno)d : %(asctime)s : %(message)s")
        log_handler.setFormatter(handler_formatter)
        log_handler.setLevel(log_level)

        root = logging.getLogger()
        root.setLevel( logging.NOTSET )

        logger = logging.getLogger('macsyfinder')
        logger.setLevel(log_level)
        logger.addHandler(log_handler)

        f_out_log_handler = logging.FileHandler(os.path.join(working_dir, 'macsyfinder.out'))
        f_out_handler_formatter = logging.Formatter("%(message)s")
        f_out_log_handler.setFormatter(f_out_handler_formatter)
        f_out_log_handler.setLevel(logging.INFO)

        c_out_log_handler = logging.StreamHandler(sys.stdout)
        c_out_handler_formatter = logging.Formatter("%(message)s")
        c_out_log_handler.setFormatter(f_out_handler_formatter)
        c_out_log_handler.setLevel(logging.INFO)

        out_logger = logging.getLogger('macsyfinder.out')
        out_logger.setLevel(logging.INFO)
        out_logger.addHandler(f_out_log_handler)
        out_logger.addHandler(c_out_log_handler)

        

        self._log = logging.getLogger('macsyfinder.config')
        for error in log_error:
            self._log.warn(error)
        try:
            if cmde_line_opt.get('previous_run', None):
                if os.path.exists(cmde_line_opt['previous_run']):
                    options['previous_run'] = cmde_line_opt['previous_run']
                else:
                    raise ValueError( "previous run directory '%s' was not found" % cmde_line_opt['previous_run'])
            try:
                options['sequence_db'] = self.parser.get( 'base', 'file', vars = cmde_line_opt )    
            except NoSectionError:
                sequence_db = cmde_line_opt.get( 'sequence_db' , None )
                if sequence_db is None:
                    raise ValueError( "No input sequence file specified")
                else:
                    options['sequence_db'] = sequence_db
            if not os.path.exists(options['sequence_db']):
                raise ValueError( "%s: The input sequence file does not exist " % options['sequence_db'])

            options['sequence_db'] = os.path.abspath(options['sequence_db'])
            val_4_db_type = ('unordered_replicon', 'ordered_replicon', 'gembase', 'unordered')
            if 'db_type' in cmde_line_opt:
                options['db_type'] = cmde_line_opt['db_type']
            else:
                try:
                    options['db_type'] = self.parser.get( 'base', 'type') 
                except (NoSectionError, NoOptionError):
                    raise ValueError( "You must specify the type of the input dataset (%s)." %  ', '.join(val_4_db_type) )
            if options['db_type'] not in val_4_db_type:
                raise ValueError( "Allowed values for the input dataset are : %s" % ', '.join(val_4_db_type))    
            val_4_replicon_topology = ('linear', 'circular')
            if 'replicon_topology' in cmde_line_opt:
                options['replicon_topology'] = cmde_line_opt['replicon_topology']
            else:
                try:
                    options['replicon_topology'] = self.parser.get( 'base', 'replicon_topology') 
                except (NoSectionError, NoOptionError):
                    options['replicon_topology'] =  self._defaults['replicon_topology']
            if options['replicon_topology'] not in val_4_replicon_topology:
                raise ValueError( "Allowed values for dataset replicon_topology are : %s" % ', '.join(val_4_replicon_topology))         
            if options['replicon_topology'] == 'circular' and options['db_type'] in ( 'unordered_replicon', 'unordered' ):
                self._log.warning("As the input dataset type 'db_type' is set to %s, the replicon_topology file was ignored")
            
            if 'topology_file' in cmde_line_opt:
                options['topology_file'] = cmde_line_opt['topology_file']
            else:
                try:
                    options['topology_file'] = self.parser.get( 'base', 'topology_file') 
                except (NoSectionError, NoOptionError):
                    options['topology_file'] = None
            if options['topology_file'] is not None:
                if not os.path.exists(options['topology_file']):
                    raise ValueError('topology_file cannot access {}: No such file'.format(options['topology_file']))
                elif not os.path.isfile(options['topology_file']):
                    raise ValueError('topology_file {} is not a regular file'.format(options['topology_file']))
            if self.parser.has_option("system", "inter_gene_max_space"):
                options['inter_gene_max_space'] = {}
                inter_gene_max_space = self.parser.get("system", "inter_gene_max_space" ) 
                inter_gene_max_space = inter_gene_max_space.split()
                it = iter( inter_gene_max_space )
                try:
                    for system in it:
                        interval = it.next()
                        try:
                            interval = int( interval)
                            options['inter_gene_max_space'][system] = interval
                        except ValueError:
                            raise ValueError("The 'inter_gene_max_space for system %s must be an integer, but you provided %s in the configuration file" % (system, interval))
                except StopIteration:
                    raise ValueError( "Invalid syntax for 'inter_gene_max_space': you must have a list of systems and corresponding 'inter_gene_max_space' separated by spaces")
            if 'inter_gene_max_space' in cmde_line_values and cmde_line_values['inter_gene_max_space'] is not None: 
                if not 'inter_gene_max_space' in options:
                    options['inter_gene_max_space'] = {}
                for item in cmde_line_values['inter_gene_max_space']:
                    system, interval = item
                    try:
                        interval = int( interval)
                        options['inter_gene_max_space'][system] = interval
                    except ValueError:
                        raise ValueError("The 'inter_gene_max_space for system %s must be an integer, but you provided %s on command line" % (system, interval))

            if self.parser.has_option("system", "min_mandatory_genes_required"):
                options['min_mandatory_genes_required'] = {}
                min_mandatory_genes_required = self.parser.get("system", "min_mandatory_genes_required" ) 
                min_mandatory_genes_required = min_mandatory_genes_required.split()
                it = iter( min_mandatory_genes_required )
                try:
                    for system in it:
                        quorum_mandatory_genes = it.next()
                        try:
                            quorum_mandatory_genes = int(quorum_mandatory_genes)
                            options['min_mandatory_genes_required'][system] = quorum_mandatory_genes
                        except ValueError:
                            raise ValueError( "The value for 'min_mandatory_genes_required' option for system %s must be an integer, but you provided %s in the configuration file" % (system, quorum_mandatory_genes))
                except StopIteration:
                    raise ValueError( "Invalid syntax for 'min_mandatory_genes_required': you must have a list of systems and corresponding 'min_mandatory_genes_required' separated by spaces")

            if 'min_mandatory_genes_required' in cmde_line_values and cmde_line_values['min_mandatory_genes_required'] is not None: 
                if not 'min_mandatory_genes_required' in options:
                    options['min_mandatory_genes_required'] = {}
                for item in cmde_line_values['min_mandatory_genes_required']:
                    system, quorum_mandatory_genes = item
                    try:
                        quorum_mandatory_genes = int(quorum_mandatory_genes)
                        options['min_mandatory_genes_required'][system] = quorum_mandatory_genes
                    except ValueError:
                        raise ValueError("The value for 'min_mandatory_genes_required' option for system %s must be an integer, but you provided %s on command line" % (system, quorum_mandatory_genes))


            if self.parser.has_option("system", "min_genes_required"):
                options['min_genes_required'] = {}
                min_genes_required = self.parser.get("system", "min_genes_required")
                min_genes_required = min_genes_required.split()
                it = iter(min_genes_required)
                try:
                    for system in it:
                        quorum_genes = it.next()
                        try:
                            quorum_genes = int(quorum_genes)
                            options['min_genes_required'][system] = quorum_genes
                        except ValueError:
                            raise ValueError("The value for 'min_genes_required' option for system %s must be an integer, but you provided %s in the configuration file" % (system, quorum_genes))
                except StopIteration:
                    raise ValueError("Invalid syntax for 'min_genes_required': you must have a list of systems and corresponding 'min_mandatory_genes_required' separated by spaces")
            if 'min_genes_required' in cmde_line_values and cmde_line_values['min_genes_required'] is not None: 
                if not 'min_genes_required' in options:
                    options['min_genes_required'] = {}
                for item in cmde_line_values['min_genes_required']:
                    system, quorum_genes = item
                    try:
                        quorum_genes = int( quorum_genes)
                        options['min_genes_required'][system] = quorum_genes
                    except ValueError:
                        raise ValueError("The value for 'min_genes_required' option for system %s must be an integer, but you provided %s on command line" % (system, quorum_genes))

            if self.parser.has_option("system", "max_nb_genes"):
                options['max_nb_genes'] = {}
                max_nb_genes = self.parser.get("system", "max_nb_genes") 
                max_nb_genes = max_nb_genes.split()
                it = iter(max_nb_genes)
                try:
                    for system in it:
                        max_genes = it.next()
                        try:
                            max_genes = int(max_genes)
                            options['max_nb_genes'][system] = max_genes
                        except ValueError:
                            raise ValueError("The value for 'max_nb_genes' option for system %s must be an integer, but you provided %s in the configuration file" % (system, max_genes))
                except StopIteration:
                    raise ValueError("Invalid syntax for 'max_nb_genes': you must have a list of systems and corresponding 'max_nb_genes' separated by spaces")
            if 'max_nb_genes' in cmde_line_values and cmde_line_values['max_nb_genes'] is not None: 
                if not 'max_nb_genes' in options:
                    options['max_nb_genes'] = {}
                for item in cmde_line_values['max_nb_genes']:
                    system, max_genes = item
                    try:
                        max_genes = int(max_genes)
                        options['max_nb_genes'][system] = max_genes
                    except ValueError:
                        raise ValueError("The value for 'max_nb_genes' option for system %s must be an integer, but you provided %s on command line" % (system, max_genes))

            if self.parser.has_option("system", "multi_loci"):
                options['multi_loci'] = self.parser.get("system", "multi_loci").split(',')
            else:
                options['multi_loci'] = []
            if 'multi_loci' in cmde_line_values and cmde_line_values['multi_loci'] is not None:
                if not 'min_genes_required' in options:
                    options['multi_loci'] = []
                for item in cmde_line_values['multi_loci'].split(','):
                    options['multi_loci'].append(item)

            try:
                options['hmmer_exe'] = self.parser.get('hmmer', 'hmmer_exe', vars = cmde_line_opt)
            except NoSectionError:
                if 'hmmer_exe' in cmde_line_opt:
                    options['hmmer_exe'] = cmde_line_opt['hmmer_exe']
                else:
                    options['hmmer_exe'] = self._defaults['hmmer_exe']

            try:
                options['index_db_exe'] = self.parser.get('base', 'index_db_exe', vars = cmde_line_opt)
            except NoSectionError:
                if 'index_db_exe' in cmde_line_opt:
                    options['index_db_exe'] = cmde_line_opt['index_db_exe']
                else:
                    options['index_db_exe'] = self._defaults['index_db_exe']

            try:
                e_value_res = self.parser.get('hmmer', 'e_value_res', vars = cmde_line_opt)
                options['e_value_res'] = float(e_value_res)
            except ValueError:
                msg = "Invalid value for hmmer e_value_res :{0}: (float expected)".format(e_value_res)
                raise ValueError( msg )
            except NoSectionError:
                if 'e_value_res' in cmde_line_opt:
                    options['e_value_res'] = float(cmde_line_opt['e_value_res'])
                else:
                    options['e_value_res'] = float(self._defaults['e_value_res'])
            try:
                i_evalue_sel = self.parser.get('hmmer', 'i_evalue_sel', vars = cmde_line_opt)
                options['i_evalue_sel'] = float(i_evalue_sel)
            except ValueError:
                msg = "Invalid value for hmmer i_evalue_sel :{0}: (float expected)".format(i_evalue_sel)
                raise ValueError( msg )
            except NoSectionError:
                if 'i_evalue_sel' in cmde_line_opt:
                    options['i_evalue_sel'] = float(cmde_line_opt['i_evalue_sel'])
                else:
                    options['i_evalue_sel'] = float(self._defaults['i_evalue_sel'])

            if options['i_evalue_sel'] > options['e_value_res']:
                raise ValueError( "i_evalue_sel (%f) must be lower or equal than e_value_res (%f)" %( options['i_evalue_sel'], options['e_value_res']) )

            try:
                coverage_profile = self.parser.get('hmmer', 'coverage_profile', vars = cmde_line_opt)
                options['coverage_profile'] = float(coverage_profile)
            except ValueError:
                msg = "Invalid value for hmmer coverage_profile :{0}: (float expected)".format(coverage_profile)
                raise ValueError( msg )
            except NoSectionError:
                if 'coverage_profile' in cmde_line_opt:
                    options['coverage_profile'] = float(cmde_line_opt['coverage_profile'])
                else:
                    options['coverage_profile'] = float(self._defaults['coverage_profile'])

            try:
                options['def_dir'] = self.parser.get('directories', 'def_dir', vars = cmde_line_opt)
            except NoSectionError:
                if 'def_dir' in cmde_line_opt:
                    options['def_dir'] = cmde_line_opt['def_dir']
                else:
                    options['def_dir'] = self._defaults['def_dir']
            if not os.path.exists(options['def_dir']):
                raise ValueError( "%s: No such definition directory" % options['def_dir'])       


            try:
                options['profile_dir'] = self.parser.get('directories', 'profile_dir', vars = cmde_line_opt)
            except NoSectionError:
                if 'profile_dir' in cmde_line_opt:
                    options['profile_dir'] = cmde_line_opt['profile_dir']
                else:
                    options['profile_dir'] = self._defaults['profile_dir']
            if not os.path.exists( options['profile_dir'] ):
                raise ValueError( "%s: No such profile directory" % options['profile_dir'])

            try:
                options['res_search_suffix'] =  self.parser.get('directories', 'res_search_suffix', vars = cmde_line_opt)
            except NoSectionError:
                if 'res_search_suffix' in cmde_line_opt:
                    options['res_search_suffix'] = cmde_line_opt['res_search_suffix']
                else:
                    options['res_search_suffix'] = self._defaults['res_search_suffix']
            try:
                options['res_extract_suffix'] = self.parser.get('directories', 'res_extract_suffix', vars = cmde_line_opt)
            except NoSectionError:
                if 'res_extract_suffix' in cmde_line_opt:
                    options['res_extract_suffix'] = cmde_line_opt['res_extract_suffix']
                else:
                    options['res_extract_suffix'] = self._defaults['res_extract_suffix']
            try:
                options['profile_suffix'] = self.parser.get('directories', 'profile_suffix', vars = cmde_line_opt)
            except NoSectionError:
                if 'profile_suffix' in cmde_line_opt:
                    options['profile_suffix'] = cmde_line_opt['profile_suffix']
                else:
                    options['profile_suffix'] = self._defaults['profile_suffix']
            try:
                worker_nb = self.parser.get('general', 'worker_nb', vars = cmde_line_opt)

            except NoSectionError:
                if 'worker_nb' in cmde_line_opt:
                    worker_nb = cmde_line_opt['worker_nb']
                else:
                    worker_nb = self._defaults['worker_nb']
            try:
                worker_nb = int(worker_nb)
                if worker_nb >= 0:
                    options['worker_nb'] = worker_nb
            except ValueError:
                msg = "the number of worker must be an integer"
                raise ValueError( msg)

        except ValueError, err:
            self._log.error(str(err), exc_info= True)
            if working_dir:
                import shutil
                try:
                    shutil.rmtree(working_dir)
                except:
                    pass
            raise err
        #build_indexes is not meaningfull in configuration file
        options['build_indexes']  = cmde_line_values['build_indexes']
        return options


[docs]    def save(self, dir_path ):
        """
        save the configuration used for this run in the ini format file
        """
        parser = SafeConfigParser()
        parser.add_section( 'base')
        parser.set( 'base', 'file', str(self.options['sequence_db']))
        parser.set( 'base', 'type', str(self.options['db_type']).lower())
        cfg_opts = [('base' ,('replicon_topology', 'topology_file', 'index_db_exe',)),
                    ('system', ('inter_gene_max_space', 'min_mandatory_genes_required', 'min_genes_required','max_nb_genes', 'multi_loci')),
                    ('hmmer', ('hmmer_exe', 'e_value_res', 'i_evalue_sel', 'coverage_profile' )),
                    ('directories', ('def_dir', 'res_search_dir', 'res_search_suffix', 'profile_dir', 'profile_suffix', 'res_extract_suffix')),
                    ('general', ('log_level', 'log_file', 'worker_nb'))
                    ]

        for section, directives in cfg_opts:
            if not parser.has_section(section):
                parser.add_section(section)
            for directive in directives:
                try:
                    if self.options[directive] is not None:
                        if directive in ('inter_gene_max_space', 'min_mandatory_genes_required', 'min_genes_required', 'max_nb_genes'):
                            s = ''
                            for system, space in self.options[directive].items():
                                s += " %s %s" % (system, space)
                            parser.set(section, directive, s)
                        elif directive != 'log_file' or self.options[directive] != os.path.join( self.options['working_dir'], 'macsyfinder.log'):
                            parser.set(section, directive, str(self.options[directive]))
                except KeyError:
                    pass
        with open( os.path.join(dir_path, self._new_cfg_name), 'w') as new_cfg:
            parser.write(new_cfg)

@property

[docs]    def sequence_db(self):
        """
        :return: the path to the input sequence dataset (in fasta format)
        :rtype: string 
        """
        return self.options['sequence_db']

@property

[docs]    def db_type(self):
        """
        :return: the type of the input sequence dataset. The allowed values are :'unordered_replicon', 'ordered_replicon', 'gembase', 'unordered'
        :rtype: string
        """
        return self.options['db_type']

@property

[docs]    def build_indexes(self):
        """
        :return: True if the indexes must be rebuilt, False otherwise
        :rtype: boolean
        """
        return self.options['build_indexes']

@property

[docs]    def replicon_topology(self):
        """
        :return: the topology of the replicons. Two values are supported 'linear' (default) and circular. Only relevant for 'ordered' datasets
        :rtype: string
        """
        return self.options['replicon_topology']

@property

[docs]    def topology_file(self):
        """
        :return: the path to the file of replicons topology. 
        :rtype: string
        """
        return self.options['topology_file']


[docs]    def inter_gene_max_space(self, system):
        """
        :param system: the name of a system 
        :type system: string
        :return: the maximum number of components with no match allowed between two genes with a match to consider them contiguous (at the system level)
        :rtype: integer 
        """
        try:
            return self.options['inter_gene_max_space'][system] 
        except KeyError:
            return None


[docs]    def min_mandatory_genes_required(self, system):
        """
        :param system: the name of a system 
        :type system: string
        :return: the mandatory genes quorum to assess the system presence
        :rtype: integer 
        """
        try:
            return self.options['min_mandatory_genes_required'][system] 
        except KeyError:
            return None


[docs]    def min_genes_required(self, system):
        """
        :param system: the name of a system 
        :type system: string
        :return: the genes (mandatory+accessory) quorum to assess the system presence
        :rtype: integer
        """
        try:
            return self.options['min_genes_required'][system] 
        except KeyError:
            return None


[docs]    def max_nb_genes(self, system):
        """
        :param system: the name of a system 
        :type system: string
        :return: the maximum number of genes to assess the system presence
        :rtype: integer
        """
        try:
            return self.options['max_nb_genes'][system] 
        except KeyError:
            return None


[docs]    def multi_loci(self, system):
        """
        :param system: the name of a system 
        :type system: string
        :return: the genes (mandatory+accessory) quorum to assess the system presence
        :rtype: boolean
        """
        try:
            return system in self.options['multi_loci'] 
        except KeyError:
            return False

@property

[docs]    def hmmer_exe(self):
        """
        :return: the name of the binary to execute for homology search from HMM protein profiles (Hmmer)
        :rtype: string 
        """
        return self.options['hmmer_exe']

@property

[docs]    def index_db_exe(self):
        """
        :return: the name of the binary to index the input sequences dataset for Hmmer
        :rtype: string 
        """
        return self.options['index_db_exe']

@property

[docs]    def e_value_res(self):
        """
        :return: The e_value threshold used by Hmmer to report hits in the Hmmer raw output file
        :rtype: float
        """
        return self.options['e_value_res']

@property

[docs]    def i_evalue_sel(self):
        """
        :return: the i_evalue threshold used to select a hit for systems detection and for the Hmmer report (filtered hits)
        :rtype: float
        """
        return self.options['i_evalue_sel']

@property

[docs]    def coverage_profile(self):
        """
        :return: the coverage threshold used to select a hit for systems detection and for the Hmmer report (filtered hits)
        :rtype: float
        """
        return self.options['coverage_profile']

@property

[docs]    def def_dir(self):
        """
        :return: the path to the directory where are stored definitions of secretion systems (.xml files)
        :rtype: string
        """
        return self.options['def_dir']

@property

[docs]    def res_search_dir(self):
        """
        :return the path to the directory to store results of MacSyFinder runs
        :rtype: string
        """
        return self.options['res_search_dir']

@property

[docs]    def working_dir(self):
        """
        :return: the path to the working directory to use for this run
        :rtpe: string
        """
        return self.options['working_dir']

@property

[docs]    def res_search_suffix(self):
        """
        :return: the suffix for Hmmer raw output files
        :rtype: string
        """
        return self.options['res_search_suffix']

@property

[docs]    def profile_dir(self):
        """
        :return: the path to the directory where are the HMM protein profiles which corresponds to Gene
        :rtype: string
        """
        return self.options['profile_dir']

@property

[docs]    def profile_suffix(self):
        """
        :return: the suffix for profile files
        :rtype: string
        """
        return self.options['profile_suffix']

@property

[docs]    def res_extract_suffix(self):
        """
        :return: the suffix of extract files (tabulated files after HMM output parsing and filtering of hits)
        :rtype: string
        """
        return self.options['res_extract_suffix']

@property

[docs]    def worker_nb(self):
        """
        :return: the maximum number of parallel jobs
        :rtype: int
        """
        return self.options.get('worker_nb', None)

@property

[docs]    def previous_run(self):
        """
        :return: the path to the previous run directory to use (to recover Hmmer raw output)
        :rtype: string
        """
        return self.options.get('previous_run', None)

@property

[docs]    def hmmer_dir(self):
        """
        :return: the name of the directory where the hmmer results are stored
        :rtype: string
        """
        return "hmmer_results"
```

### Quick search


Enter search terms or a module, class or function name.

### Navigation

- index
- modules |
- MacSyFinder documentation »
- Module code »

© Copyright 2014, Sophie Abby, Bertrand Néron.
Created using Sphinx 1.1.3.
