# Supplementary material for: MacSyFinder: A Program to Mine Genomes for Molecular Systems with an Application to CRISPR-Cas Systems
Source: PLoS One. 2014 Oct 17;9(10):e110726. doi: 10.1371/journal.pone.0110726 (PMC4201578; doi:10.1371/journal.pone.0110726)
Supplement: Data S1 — The MacSyFinder/MacSyView package (compressed tarball archive). (GZ) [file pone.0110726.s010.gz › macsyfinder-1.0.0-RC3/doc/_build/html/_modules/macsypy/database.html]

macsypy.database — MacSyFinder 1.0.0-RC3 documentation


### Navigation

- index
- modules |
- MacSyFinder documentation »
- Module code »

# Source code for macsypy.database

```
# -*- coding: utf-8 -*-

################################################################################
# MacSyFinder - Detection of macromolecular systems in protein datasets        #
#               using systems modelling and similarity search.                 #
# Authors: Sophie Abby, Bertrand Néron                                         #
# Copyright © 2014  Institut Pasteur, Paris.                                   #
# See the COPYRIGHT file for details                                           #
#                                                                              #
# MacsyFinder is distributed under the terms of the GNU General Public License #
# (GPLv3). See the COPYING file for details.                                   #
################################################################################


from itertools import groupby
from collections import namedtuple
from glob import glob
import os.path
import logging
_log = logging.getLogger('macsyfinder.' + __name__)
from subprocess import Popen
from macsypy_error import MacsypyError

[docs]def fasta_iter(fasta_file):
    """
    :param fasta_file: the file containing all input sequences in fasta format.
    :type fasta_file: file object
    :author: http://biostar.stackexchange.com/users/36/brentp
    :return: for a given fasta file, it returns an iterator which yields tuples
             (string id, string comment, int sequence length)
    :rtype: iterator
    """
    # ditch the boolean (x[0]) and just keep the header or sequence since
    # we know they alternate.
    faiter = (x[1] for x in groupby(fasta_file , lambda line: line[0] == ">"))
    for header in faiter:
        # drop the ">"
        header = header.next()[1:].strip()
        header = header.split()
        _id = header[0]
        comment = ' '.join(header[1:])
        seq = ''.join(s.strip() for s in faiter.next())
        length = len(seq)
        yield _id, comment, length


[docs]class Indexes(object):
    """
    Handle the indexes for macsyfinder:

     - find the indexes for hmmer, or build them using formatdb or makeblastdb external tools
     - find the indexes required by macsyfinder to compute some scores, or build them.
    """

[docs]    def __init__(self, cfg):
        """
        The constructor retrieves the file of indexes in the case they are not present
        or the user asked for build indexes (--idx) 
        Launch the indexes building. 

        :param cfg: the configuration 
        :type cfg: :class:`macsypy.config.Config` object
        """
        self.cfg = cfg
        self._fasta_path = cfg.sequence_db
        self.name = os.path.basename(cfg.sequence_db)
        self._hmmer_indexes = None  #list of path
        self._my_indexes = None  #path


[docs]    def build(self, force = False):
        """
        Build the indexes from the sequence dataset in fasta format

        :param force: If True, force the index building even if the index files are present in the sequence dataset folder
        :type force: boolean
        """
        hmmer_indexes = self.find_hmmer_indexes()
        my_indexes = self.find_my_indexes()

        ###########################
        # build indexes if needed #
        ###########################
        index_dir = os.path.abspath(os.path.dirname(self.cfg.sequence_db))

        if force or not hmmer_indexes or not my_indexes:
            #formatdb create indexes in the same directory as the sequence_db
            #so it must be writable
            #if the directory is not writable, formatdb do a Segmentation fault
            if not os.access(index_dir, os.R_OK|os.W_OK):
                msg = "cannot build indexes, (%s) is not writable" % index_dir
                _log.critical(msg)
                raise IOError(msg)

        if force or not hmmer_indexes:
            #self._build_hmmer_indexes() is asynchron
            hmmer_indexes_proc = self._build_hmmer_indexes()
        if force or not my_indexes:
            #self._build_my_indexes() is synchron
            self._build_my_indexes()

        ################################# 
        # synchronization point between #
        # hmmer_indexes and my_indexes  #
        #################################
        if force or not hmmer_indexes:
            hmmer_indexes_proc.wait()
            if hmmer_indexes_proc.returncode == 127:
                msg = "neither makeblastdb nor formatdb can be found, check your config or install makeblastb"
                _log.critical( msg, exc_info = True )
                raise RuntimeError(msg)
            if hmmer_indexes_proc.returncode != 0:
                msg = "an error occurred during databases indexation see formatdb.log"
                _log.critical( msg, exc_info = True )
                raise RuntimeError(msg)
        self._hmmer_indexes = self.find_hmmer_indexes()
        self._my_indexes = self.find_my_indexes()
        assert self._hmmer_indexes , "failed to create hmmer indexes"
        assert self._my_indexes, "failed create macsyfinder indexes"


[docs]    def find_hmmer_indexes(self):
        """
        :return: The hmmer index files. 
                 If indexes are inconsistent (some file(s) missing), a Runtime Error is raised
        :rtype: list of string 
        """
        suffixes = ('.phr', '.pin', '.psd', '.psi', '.psq', '.pal')
        idx = []
        file_nb = 0
        for suffix in suffixes:
            index_files = glob( "%s*%s" % (self._fasta_path, suffix))
            nb_of_index = len(index_files)
            if suffix != '.pal':
                if file_nb and file_nb != nb_of_index:
                    msg = "some index files are missing. Delete all index files (*.phr, *.pin, *.psd, *.psi, *.psq, *.pal) and try to rebuild them."
                    _log.critical(msg)
                    raise  RuntimeError(msg)
            else:
                if nb_of_index > 1:
                    msg = "too many .pal file . Delete all index files (*.phr, *.pin, *.psd, *.psi, *.psq, *.pal) and try to rebuild them."
                    _log.critical(msg)
                    raise  RuntimeError(msg)    
                elif file_nb > 1 and nb_of_index == 0:
                    msg = "some index files are missing. Delete all index files (*.phr, *.pin, *.psd, *.psi, *.psq, *.pal) and try to rebuild them."
                    _log.critical(msg)
                    raise  RuntimeError(msg)
                elif file_nb == 1 and nb_of_index == 1:
                    msg = "a virtual index is detected (.pal) but there is only one file per index type. Delete all index files  (*.phr, *.pin, *.psd, *.psi, *.psq, *.pal) and try to rebuild them."
                    _log.critical(msg)
                    raise  RuntimeError(msg)
            idx.extend(index_files)
            file_nb = nb_of_index
        return idx


[docs]    def find_my_indexes(self):
        """
        :return: the file of macsyfinder indexes if it exists in the dataset folder, None otherwise. 
        :rtype: string
        """ 
        path = os.path.join( os.path.dirname(self.cfg.sequence_db), self.name + ".idx")
        if os.path.exists(path):
            return path


[docs]    def _build_hmmer_indexes(self):
        """
        build the index files for hmmer using the formatdb or makeblastdb tool
        """
        index_dir = os.path.dirname(self.cfg.sequence_db)
        if self.cfg.index_db_exe.find('makeblast') != -1:
            command = "%s -title %s -in %s -dbtype prot -parse_seqids" % (self.cfg.index_db_exe,
                                                                      self.name,
                                                                      self.cfg.sequence_db)
        elif self.cfg.index_db_exe.find('formatdb') != -1:
            # -t  Title for database file [String]
            # -i Input file(s) for formatting [File In]
            # -p T Type of file = protein
            # -o T Parse SeqId and create indexes.
            # -s T Create indexes limited only to accessions
            command = "%s -t %s -i %s -p T -o T -s T" % ( self.cfg.index_db_exe,
                                                      self.name,
                                                      self.cfg.sequence_db
                                                          )
        else:
            raise MacsypyError("%s is not supported to index the sequence dataset. Please use makeblastdb or formatdb." % self.cfg.sequence_db)

        _log.debug("hmmer index command: {0}".format(command))
        err_path = os.path.join(index_dir, "formatdb.err")
        with  open(err_path, 'w') as err_file:
            try:
                formatdb = Popen( command ,
                                  shell = True ,
                                  stdout = err_file ,
                                  stdin  = None ,
                                  stderr = err_file ,
                                  close_fds = False ,
                                  )
            except Exception, err:
                msg = "unable to index the sequence dataset : %s : %s" % (command, err)
                _log.critical( msg, exc_info = True )
                raise err
            return formatdb


[docs]    def _build_my_indexes(self):
        """
        Build macsyfinder indexes. These indexes are stored in a file.

        The file format is the following:
         - one entry per line, with each line having this format:
         - sequence id;sequence length;sequence rank

        """
        try:
            with open(self._fasta_path, 'r') as fasta_file:
                with open(os.path.join(os.path.dirname(self.cfg.sequence_db), self.name + ".idx" ), 'w') as my_base:
                    f_iter = fasta_iter(fasta_file)
                    seq_nb = 0
                    for seqid, comment, length in f_iter:
                        seq_nb += 1
                        my_base.write("%s;%d;%d\n" % (seqid, length, seq_nb))
        except Exception, err:
            msg = "unable to index the sequence dataset: %s : %s" % (self.cfg.sequence_db, err)
            _log.critical(msg, exc_info = True)
            raise err

"""handle name, topology type, and min/max positions in the sequence dataset for a replicon"""
RepliconInfo = namedtuple('RepliconInfo', 'topology, min, max, genes')

[docs]class RepliconDB(object):
    """
    Stores information (topology, min, max, [genes]) for all replicons in the sequence_db
    the Replicon object must be instantiated only for sequence_db of type 'gembase' or 'ordered_replicon'
    """

    ordered_replicon_name = 'UserReplicon'

[docs]    def __init__(self, cfg):
        """
        :param cfg: The configuration object
        :type cfg: :class:`macsypy.config.Config` object

        .. note ::
            This class can be instanciated only if the db_type is 'gembase' or 'ordered_replicon' 
        """
        self.cfg = cfg
        assert self.cfg.db_type in ('gembase', 'ordered_replicon')
        idx = Indexes(self.cfg)
        self.sequence_idx = idx.find_my_indexes()
        self.topology_file = self.cfg.topology_file
        self._DB = {}
        if self.topology_file:
            topo_dict = self._fill_topology()
        else:
            topo_dict = {}
        if self.cfg.db_type == 'gembase':
            self._fill_gembase_min_max(topo_dict, default_topology = self.cfg.replicon_topology)
        else:
            self._fill_ordered_min_max(self.cfg.replicon_topology)


[docs]    def _fill_topology(self):
        """
        Fill the internal dictionary with min and max positions for each replicon_name of the sequence_db
        """
        topo_dict = {}
        with open(self.topology_file) as topo_f:
            for l in topo_f:
                if l.startswith('#'): 
                    continue
                replicon_name, topo = l.split(':')
                replicon_name = replicon_name.strip()
                topo = topo.strip().lower()
                topo_dict[replicon_name] = topo
        return topo_dict


[docs]    def _fill_ordered_min_max(self, default_topology = None):
        """
        For the replicon_name of the ordered_replicon sequence base, fill the internal dict with RepliconInfo

        :param default_topology: the topology provided by config.replicon_topology 
        :type default_topology: string
        """
        _min = 1
        #self.sequence_idx is a file with the following structure seq_id;seq_length;seq_rank\n
        with open(self.sequence_idx) as idx_f:
            _max = 0
            genes = []
            for l in idx_f:
                seq_id, length, _rank = l.split(";")
                genes.append((seq_id, length))
                _max += 1
            self._DB[self.ordered_replicon_name] = RepliconInfo(default_topology, _min, _max, genes)


[docs]    def _fill_gembase_min_max(self, topology, default_topology):
        """
        For each replicon_name of a gembase dataset, it fills the internal dictionary with a namedtuple RepliconInfo

        :param topology: the topologies for each replicon 
                         (parsed from the file specified with the option --topology-file)
        :type topology: dict
        :param default_topology: the topology provided by the config.replicon_topology 
        :type default_topology: string
        """
        def grp_replicon(line):
            """
            in gembase the idtentifier of fasta sequence follow the following schema: replicon_name_seq_name 
            so grp_replicon allow to group sequences belonging to the same replicon.
            """
            return line.split('_')[0]

        def parse_entry(entry):
            """
            parse an entry in the index file (.idx)
            an entry have the folowing format sequence_id;sequence lenght;sequence rank in replicon
            """
            entry = entry.rstrip()
            seq_id, length, rank = entry.split(';')
            replicon_name , seq_name = seq_id.split('_')
            return replicon_name, seq_name, length, int(rank)

        with open(self.sequence_idx) as idx_f:
            replicons = (x[1] for x in groupby(idx_f, grp_replicon))
            for replicon in replicons:
                genes = []
                entry = replicon.next()
                replicon_name, seq_name, seq_lenght, _min = parse_entry(entry)
                genes.append((seq_name, seq_lenght))
                for entry in replicon:
                    #pass all sequence of the replicon until the last one
                    _, seq_name, seq_lenght, _ = parse_entry(entry)
                    genes.append((seq_name, seq_lenght))
                _, seq_name, seq_lenght, _max = parse_entry(entry)
                genes.append((seq_name, seq_lenght))
                if replicon_name in topology:
                    self._DB[replicon_name] = RepliconInfo(topology[replicon_name], _min, _max, genes)
                else:
                    self._DB[replicon_name] = RepliconInfo(default_topology, _min, _max, genes)


[docs]    def __contains__(self, replicon_name):
        """
        :param replicon_name: the name of the replicon
        :type replicon_name: string
        :returns: True if replicon_name is in the repliconDB, false otherwise.
        :rtype: boolean
        """
        return replicon_name in self._DB


[docs]    def __getitem__(self, replicon_name):
        """
        :param replicon_name: the name of the replicon to get information on
        :type replicon_name: string
        :returns: the RepliconInfo for the provided replicon_name 
        :rtype: :class:`RepliconInfo` object
        :raise: KeyError if replicon_name is not in repliconDB
        """
        return self._DB[replicon_name]


[docs]    def get(self, replicon_name, default = None):
        """
        :param replicon_name: the name of the replicon to get informations
        :type replicon_name: string
        :param default: the value to return if the replicon_name is not in the RepliconDB
        :type default: any
        :returns: the RepliconInfo for replicon_name if replicon_name is in the repliconDB, else default. If default is not given, it is set to None, so that this method never raises a KeyError.
        :rtype: :class:`RepliconInfo` object
        """
        return self._DB.get(replicon_name, default)


[docs]    def items(self):
        """
        :return: a copy of the RepliconDB as a list of (replicon_name, RepliconInfo) pairs
        """
        return self._DB.items()


[docs]    def iteritems(self):
        """
        :return: an iterator over the RepliconDB as a list (replicon_name, RepliconInfo) pairs
        """
        return self._DB.iteritems()


[docs]    def replicon_names(self):
        """
        :return: a copy of the RepliconDB as a list of replicon_names
        """
        return self._DB.keys()


[docs]    def replicon_infos(self):
        """
        :return: a copy of the RepliconDB as list of replicons info
        :rtype: RepliconInfo instance
        """
        return self._DB.values()
```

### Quick search


Enter search terms or a module, class or function name.

### Navigation

- index
- modules |
- MacSyFinder documentation »
- Module code »

© Copyright 2014, Sophie Abby, Bertrand Néron.
Created using Sphinx 1.1.3.
