# Supplementary material for: MacSyFinder: A Program to Mine Genomes for Molecular Systems with an Application to CRISPR-Cas Systems
Source: PLoS One. 2014 Oct 17;9(10):e110726. doi: 10.1371/journal.pone.0110726 (PMC4201578; doi:10.1371/journal.pone.0110726)
Supplement: Data S1 — The MacSyFinder/MacSyView package (compressed tarball archive). (GZ) [file pone.0110726.s010.gz › macsyfinder-1.0.0-RC3/doc/_build/html/_modules/macsypy/gene.html]

macsypy.gene — MacSyFinder 1.0.0-RC3 documentation


### Navigation

- index
- modules |
- MacSyFinder documentation »
- Module code »

# Source code for macsypy.gene

```
# -*- coding: utf-8 -*-

################################################################################
# MacSyFinder - Detection of macromolecular systems in protein datasets        #
#               using systems modelling and similarity search.                 #
# Authors: Sophie Abby, Bertrand Néron                                         #
# Copyright © 2014  Institut Pasteur, Paris.                                   #
# See the COPYRIGHT file for details                                           #
#                                                                              #
# MacsyFinder is distributed under the terms of the GNU General Public License #
# (GPLv3). See the COPYING file for details.                                   #
################################################################################


import os
import logging
_log = logging.getLogger('macsyfinder.' + __name__)

from subprocess import Popen
from threading import Lock
from report import GembaseHMMReport, GeneralHMMReport, OrderedHMMReport
from macsypy_error import MacsypyError

[docs]class GeneBank(object):
    """
    Store all Gene objects. Ensure that genes are instanciated only once.
    """
    _genes_bank = {}

[docs]    def __getitem__(self, name):
        """
        :param name: the name of the Gene
        :type name: string
        :param cfg: the configuration object
        :type cfg: :class:`macsypy.config.Config` object
        :return: return the Gene corresponding to the name.
          If the Gene already exists, return it, otherwise, build it and return it
        :rtype: :class:`macsypy.gene.Gene` object
        """
        if name in self._genes_bank:
            return self._genes_bank[name]
        else:
            raise KeyError(name)


[docs]    def __contains__(self, gene):
        """
        Implement the membership test operator

        :param gene: the gene to test
        :type gene: :class:`macsypy.gene.Gene` object
        :return: True if the gene is in, False otherwise
        :rtype: boolean
        """
        return gene in self._genes_bank.values()


[docs]    def __iter__(self):
        """
        Return an iterator object on the genes contained in the bank
        """
        return self._genes_bank.itervalues()


[docs]    def add_gene(self, gene):
        """

        :param name: the name of the gene
        :type name: string
        :param cfg: the configuration
        :type cfg: :class:`macsypy.config.Config` object
        :return: return gene corresponding to the name.
          If the gene already exists, return it, otherwise, build it and return it
        :rtype: :class:`macsypy.gene.Gene` object
        :raise: KeyError if a gene with the same name is already registered
        """
        if gene in self._genes_bank:
            raise KeyError("a gene named %s is already registered" % gene.name)
        else:
            self._genes_bank[gene.name] = gene

gene_bank = GeneBank()

[docs]class Gene(object):
    """
    Handle Gene of a (secretion) System

    """

[docs]    def __init__(self, cfg, name,
                 system,
                 profiles_registry,
                 loner = False,
                 exchangeable = False,
                 multi_system = False,
                 inter_gene_max_space = None ):
        """
        handle gene

        :param cfg: the configuration object. 
        :type cfg: :class:`macsypy.config.Config` object
        :param name: the name of the Gene.
        :type name: string.
        :param system: the system that owns this Gene
        :type system: :class:`macsypy.system.System` object.
        :param profiles_registry: where all the paths profiles where register.
        :type profiles_registry: :class:`macsypy.registries.ProfilesRegistry` object.
        :param loner: True if the Gene can be isolated on the genome (with no contiguous genes), False otherwise.
        :type loner: boolean.
        :param exchangeable: True if this Gene can be replaced with one of its homologs or analogs \
          whithout any effects on the system assessment, False otherwise.
        :type exchangeable: boolean.
        :param multi_system: True if this Gene can belong to different occurrences of this System. 
        :type multi_system: boolean.
        :param inter_gene_max_space: the maximum space between this Gene and another gene of the System.
        :type inter_gene_max_space: integer
        """
        self.name = name 
        self.profile = profile_factory.get_profile(self, cfg, profiles_registry)
        """:ivar profile: The HMM protein Profile corresponding to this gene :class:`macsypy.gene.Profile` object"""

        self.homologs = []
        self.analogs = []
        self._system = system
        self._loner = loner
        self._exchangeable = exchangeable
        self._multi_system = multi_system
        self._inter_gene_max_space = inter_gene_max_space


[docs]    def __str__(self):
        """
        Print the name of the gene and of its homologs/analogs.
        """
        s = "name : %s" % self.name
        s += "\ninter_gene_max_space: %d"%self.inter_gene_max_space
        if self.loner:
            s+= "\nloner"
        if self.multi_system:
            s+= "\nmulti_system"
        if self.exchangeable:
            s+= "\nexchangeable"
        if self.homologs:
            s += "\n    homologs: "
            for h in self.homologs:
                s += h.name + ", "
            s = s[:-2]
        if self.analogs:
            s += "\n    analogs: "
            for a in self.analogs:
                s += a.name + ", "
            s = s[:-2]
        return s

@property

[docs]    def system(self):
        """
        :return: the System that owns this Gene
        :rtype: :class:`macsypy.system.System` object
        """
        return self._system

@property

[docs]    def loner(self):
        """
        :return: True if the gene can be isolated on the genome, False otherwise
        :rtype: boolean
        """
        return self._loner

@property

[docs]    def exchangeable(self):
        """
        :return: True if this gene can be replaced with one of its homologs or analogs whithout any effects on the system, False otherwise.
        :rtype: boolean.
        """
        return self._exchangeable

@property

[docs]    def multi_system(self):
        """
        :return: True if this Gene can belong to different occurrences of **this System** (and can be used for multiple System assessments), False otherwise.
        :rtype: boolean.
        """
        return self._multi_system

@property

[docs]    def inter_gene_max_space(self):
        """
        :return: The maximum distance allowed between this gene and another gene for them to be considered co-localized. 
                 If the value is not set at the Gene level, return the value set at the System level.
        :rtype: integer.
        """
        if self._inter_gene_max_space is not None:
            return self._inter_gene_max_space
        else:
            return self._system.inter_gene_max_space


[docs]    def add_homolog(self, homolog):
        """
        Add a homolog gene to the Gene

        :param homolog: homolog to add
        :type homolog:  :class:`macsypy.gene.Homolog` object 
        """
        self.homologs.append(homolog)


[docs]    def get_homologs(self):
        """
        :return: the Gene homologs
        :type: list of :class:`macsypy.gene.Homolog` object
        """
        return self.homologs


[docs]    def add_analog(self, analog):
        """
        Add an analogous gene to the Gene

        :param analog: analog to add
        :type analog:  :class:`macsypy.gene.Analog` object 
        """
        self.analogs.append(analog)


[docs]    def get_analogs(self):
        """
        :return: the Gene analogs
        :type: list of :class:`macsypy.gene.Analog` object
        """
        return self.analogs


[docs]    def __eq__(self, gene):
        """
        :return: True if the gene names (gene.name) are the same, False otherwise.
        :param gene: the query of the test
        :type gene: :class:`macsypy.gene.Gene` object.
        :rtype: boolean.
        """
        return self.name == gene.name


[docs]    def is_homolog(self, gene):
        """
        :return: True if the two genes are homologs, False otherwise.
        :param gene: the query of the test
        :type gene: :class:`macsypy.gene.Gene` object.
        :rtype: boolean.
        """

        if self == gene:
            return True
        else:
            for h in self.homologs:
                if gene == h.gene:
                    return True
        return False


[docs]    def is_analog(self, gene):
        """
        :return: True if the two genes are analogs, False otherwise.
        :param gene: the query of the test
        :type gene: :class:`macsypy.gene.Gene` object.
        :rtype: boolean.
        """

        if self == gene:
            return True
        else:
            for h in self.analogs:
                if gene == h.gene:
                    return True
        return False


[docs]    def is_mandatory(self, system):
        """
        :return: True if the gene is within the *mandatory* genes of the system, False otherwise.
        :param system: the query of the test
        :type system: :class:`macsypy.system.System` object.
        :rtype: boolean.
        """
        if self in system.mandatory_genes:
            return True
        else:
            return False


[docs]    def is_accessory(self, system):
        """
        :return: True if the gene is within the *accessory* genes of the system, False otherwise.
        :param system: the query of the test
        :type system: :class:`macsypy.system.System` object.
        :rtype: boolean.
        """
        if self in system.accessory_genes:
            return True
        else:
            return False


[docs]    def is_forbidden(self, system):
        """
        :return: True if the gene is within the *forbidden* genes of the system, False otherwise.
        :param system: the query of the test
        :type system: :class:`macsypy.system.System` object.
        :rtype: boolean.
        """
        if self in system.forbidden_genes:
            return True
        else:
            return False


[docs]    def is_authorized(self, system, include_forbidden = True):
        """
        :return: True if the genes are found in the System definition file (.xml), False otherwise.
        :param system: the query of the test
        :type system: :class:`macsypy.system.System` object.
        :param include_forbidden: tells if forbidden genes should be considered as "authorized" or not
        :type include_forbidden: boolean
        :rtype: boolean.
        """
        
        #print "\n- is_authorized? -"
        #print "%s in %s"%(self, system.name)
        if include_forbidden:
            for m in (system.mandatory_genes+system.accessory_genes+system.forbidden_genes):
                if self == m:
                    #print "Yes"
                    return True
                if (m.exchangeable and m.is_homolog(self)) or (m.exchangeable and m.is_analog(self)):
                    #print "Yes - via exchang"
                    return True
        else:
            for m in (system.mandatory_genes+system.accessory_genes):
                if self == m:
                    #print "Yes"
                    return True
                if (m.exchangeable and m.is_homolog(self)) or (m.exchangeable and m.is_analog(self)):
                    #print "Yes - via exchang"
                    return True 
        #print "No!"
        return False


[docs]    def get_compatible_systems(self, system_list, include_forbidden=True):
        """
        Test every system in system_list for compatibility with the gene using the is_authorized function.

        :param system_list: a list of system names to test
        :type system_list: list of strings        
        :param include_forbidden: tells if forbidden genes should be considered as defining a compatible systems or not
        :type include_forbidden: boolean
        :return: the list of compatible systems
        :rtype: list of string, or void list if none compatible
        """
        compatibles = []
        for s in system_list:
            if self.is_authorized(s, include_forbidden):
                compatibles.append(s)
        return compatibles


[docs]class Homolog(object):
    """
    Handle homologs, encapsulate a Gene
    """

[docs]    def __init__(self, gene, gene_ref, aligned = False ):
        """
        :param gene: the gene
        :type gene: :class:`macsypy.gene.Gene` object.
        :param gene_ref: the gene to which the current is homolog.
        :type gene_ref: :class:`macsypy.gene.Gene` object.
        :param aligned: if True, the profile of this gene overlaps totally the sequence of the reference gene profile.\ 
          Otherwise, only partial overlapping between the profiles. 
        :type aligned: boolean
        """
        self.gene = gene 
        """:ivar gene: gene """

        self.ref = gene_ref 
        self.aligned = aligned

def __getattr__(self, name):
        return getattr(self.gene, name)

[docs]    def is_aligned(self):
        """
        :return: True if this gene homolog is aligned to its homolog, False otherwise.
        :rtype: boolean
        """
        return self.aligned

@property

[docs]    def gene_ref(self):
        """
        :return: the gene to which this one is homolog to (reference gene)
        :rtype: :class:`macsypy.gene.Gene` object
        """
        return self.ref


[docs]class Analog(object):
    """
    Handle analogs, encapsulate a Gene
    """

[docs]    def __init__(self, gene, gene_ref):
        """
        :param gene: the gene
        :type gene: :class:`macsypy.gene.Gene` object.
        :param gene_ref: the gene to which the current is analog.
        :type gene_ref: :class:`macsypy.gene.Gene` object.
        """
        self.gene = gene 
        """:ivar gene: gene """

        self.ref = gene_ref

def __getattr__(self, name):
        return getattr(self.gene, name)

    @property

[docs]    def gene_ref(self):
        """
        :return: the gene to which this one is analog to (reference gene)
        :rtype: :class:`macsypy.gene.Gene` object
        """
        return self.ref


[docs]class ProfileFactory():
    """
    Build and store all Profile objects. Profiles must not be instanciated directly.
    The profile_factory must be used. The profile_factory ensures there is only one instance
    of profile for a given name.
    To get a profile, use the method get_profile. If the profile is already cached, this instance is returned.
    Otherwise a new profile is built, stored in the profile_factory and then returned.

    """
    _profiles = {}

[docs]    def get_profile(self, gene, cfg, profiles_registry):
        """
        :param gene: the gene associated to this profile
        :type gene: :class:`macsypy.gene.Gene` or :class:`macsypy.gene.Homolog` or :class:`macsypy.gene.Analog` object
        :param profiles_registry: the registry where are stored the path of the profiles
        :type profiles_registry: the registry of profiles
        :param profiles_registry: :class:`macsypy.registries.ProfilesRegistry` instance.
        :return: the profile corresponding to the name.
                 If the profile already exists, return it. Otherwise build it, store it and return it.
        :rtype: :class:`macsypy.gene.Profile` object
        """
        if gene.name in self._profiles:
            profile = self._profiles[gene.name]
        else:
            path = profiles_registry.get(gene.name)
            if path is None:
                raise MacsypyError("{0}: No such profile".format(gene.name))
            profile = Profile(gene, cfg, path)
            self._profiles[gene.name] = profile
        return profile

profile_factory = ProfileFactory()

[docs]class Profile(object):
    """
    Handle a HMM protein profile
    """

[docs]    def __init__(self, gene, cfg, path):
        """

        :param gene: the gene corresponding to this profile
        :type gene_name: :class:`macsypy.secretion.Gene` object
        :param cfg: the configuration 
        :type cfg: :class:`macsypy.config.Config` object
        :param path: the path to the hmm profile.
        :type path: string
        """
        self.gene = gene 
        self.path = path
        self.len = self._len()
        self.cfg = cfg 
        self.hmm_raw_output = None
        self._report = None
        self._lock = Lock()


[docs]    def __len__(self):
        """
        :return: the length of the HMM protein profile
        :rtype: int
        """
        return self.len


[docs]    def _len(self):
        """
        Parse the HMM profile file to get and store the length.
        This private method is called at the Profile init.
        """
        with open(self.path) as f:
            for l in f:
                if l.startswith("LENG"):
                    length = int(l.split()[1])
                    break
        return length


[docs]    def __str__(self):
        """
        Print the name of the corresponding gene and the path to the HMM profile.
        """
        return "%s : %s" % (self.gene.name, self.path)


[docs]    def execute(self):
        """
        Launch the Hmmer search (hmmsearch executable) with this profile

        :return: an object storing information on th results of the HMM search (HMMReport)
        :rtype:  :class:`macsypy.report.HMMReport` object
        """
        with self._lock:
            # the results of HMM is cached 
            # so HMMsearch is executed only once per run
            # if this method is called several times the first call induce the execution of HMMsearch and generate a report
            # the other calls return directly this report
            if self._report is not None:
                return self._report
            output_path = os.path.join(self.cfg.working_dir, self.cfg.hmmer_dir, self.gene.name + self.cfg.res_search_suffix)
            err_path = os.path.join(self.cfg.working_dir, self.cfg.hmmer_dir, self.gene.name + os.path.splitext(self.cfg.res_search_suffix)[0] + ".err" )

            with  open(err_path, 'w') as err_file:
                options = { "hmmer_exe" : self.cfg.hmmer_exe,
                            "output_file" : output_path ,
                            "e_value_res" : self.cfg.e_value_res,
                            "profile" : self.path,
                            "sequence_db" : self.cfg.sequence_db,
                           }
                #command = "%(hmmer_exe)s -o %(output_file)s -E %(e_value_res)d %(profile)s %(sequence_db)s" % options
                command = "{hmmer_exe} --cpu 0 -o {output_file} -E {e_value_res:f} {profile} {sequence_db} ".format(**options)
                _log.info( "{0} Hmmer command line : {1}".format(self.gene.name, command))
                try:
                    hmmer = Popen( command ,
                                   shell = True ,
                                   stdout = None ,
                                   stdin  = None ,
                                   stderr = err_file ,
                                   close_fds = False ,
                                   )
                except Exception, err:
                    msg = "Hmmer execution failed: command = %s : %s" % ( command , err)
                    _log.critical( msg, exc_info = True )
                    raise err

                hmmer.wait()

            if hmmer.returncode != 0:
                if hmmer.returncode == -15:
                    msg = "the Hmmer execution was aborted: command = %s : return code = %d check %s" % (command, hmmer.returncode, err_path)
                    _log.critical(msg)
                    return
                else:
                    msg = "an error occurred during Hmmer execution: command = %s : return code = %d check %s" % (command, hmmer.returncode, err_path)
                    _log.debug(msg, exc_info = True )
                    _log.critical(msg)
                    raise RuntimeError(msg)
            self.hmm_raw_output = output_path
            if self.cfg.db_type == 'gembase':
                report = GembaseHMMReport(self.gene, output_path, self.cfg )
            elif self.cfg.db_type == 'ordered_replicon':
                report = OrderedHMMReport(self.gene, output_path, self.cfg )
            else:
                report = GeneralHMMReport(self.gene, output_path, self.cfg )
            self._report = report
            return report
```

### Quick search


Enter search terms or a module, class or function name.

### Navigation

- index
- modules |
- MacSyFinder documentation »
- Module code »

© Copyright 2014, Sophie Abby, Bertrand Néron.
Created using Sphinx 1.1.3.
