# Supplementary material for: MacSyFinder: A Program to Mine Genomes for Molecular Systems with an Application to CRISPR-Cas Systems
Source: PLoS One. 2014 Oct 17;9(10):e110726. doi: 10.1371/journal.pone.0110726 (PMC4201578; doi:10.1371/journal.pone.0110726)
Supplement: Data S1 — The MacSyFinder/MacSyView package (compressed tarball archive). (GZ) [file pone.0110726.s010.gz › macsyfinder-1.0.0-RC3/doc/_build/html/_modules/macsypy/report.html]

macsypy.report — MacSyFinder 1.0.0-RC3 documentation


### Navigation

- index
- modules |
- MacSyFinder documentation »
- Module code »

# Source code for macsypy.report

```
# -*- coding: utf-8 -*-

################################################################################
# MacSyFinder - Detection of macromolecular systems in protein datasets        #
#               using systems modelling and similarity search.                 #
# Authors: Sophie Abby, Bertrand Néron                                         #
# Copyright © 2014  Institut Pasteur, Paris.                                   #
# See the COPYRIGHT file for details                                           #
#                                                                              #
# MacsyFinder is distributed under the terms of the GNU General Public License #
# (GPLv3). See the COPYING file for details.                                   #
################################################################################


import os
import logging
_log = logging.getLogger('macsyfinder.' + __name__)


import abc
from threading import Lock
from itertools import groupby
from database import Indexes, RepliconDB

[docs]class HMMReport(object):
    """
    Handle the results from the HMM search. Extract a synthetic report from the raw hmmer output, after having applied a hit filtering.
    This class is an **abstract class**. There are two implementations of this abstract class
    depending on wether the input sequence dataset is "ordered" ("gembase" or "ordered_replicon" db_type) or not ("unordered" or "unordered_replicon" db_type).
    """

    __metaclass__ = abc.ABCMeta

[docs]    def __init__(self, gene, hmmer_output, cfg):
        """
        :param gene: the gene corresponding to the profile search reported here
        :type gene: :class:`macsypy.gene.Gene` object
        :param hmmer_output: The path to the raw Hmmer output file
        :type hmmer_output: string
        :param cfg: the configuration object
        :type cfg: :class:`macsypy.config.Config` object
        """
        self.gene = gene
        self._hmmer_raw_out = hmmer_output
        self._extract_out = None
        self.hits = []
        self.cfg = cfg
        self._lock = Lock()

@abc.abstractmethod

[docs]    def extract(self):
        """
        Parse the raw Hmmer output file and produce a new synthetic report file by applying a filter on hits.
        Contain selected and sorted hits ( **this abstract method is implemented in inherited classes** )
        """
        pass


[docs]    def __str__(self):
        """
        Print information on filtered hits
        """
        s = "# gene: %s extract from %s hmm output\n" % (self.gene.name, self._hmmer_raw_out)
        s += "# profile length= %d\n" % len(self.gene.profile)
        s += "# i_evalue threshold= %f\n" % self.cfg.i_evalue_sel
        s += "# coverage threshold= %f\n" % self.cfg.coverage_profile
        #s += "# hit_id replicon_name position_hit gene_name gene_system i_eval score coverage\n"
        #s += "# hit_id replicon_name position_hit gene_name gene_system i_eval score profile_coverage sequence_coverage begin end\n"
        s += "# hit_id replicon_name position_hit hit_sequence_length gene_name gene_system i_eval score profile_coverage sequence_coverage begin end\n"
        for h in self.hits:
            s += str(h)
        return s


[docs]    def save_extract(self):
        """
        Write the string representation of the extact report in a file.
        The name of this file is the concatenation of the gene name and of the "res_extract_suffix" from the config object
        """
        with self._lock:
            extract_out_name = self.gene.name + self.cfg.res_extract_suffix
            self._extract_out = os.path.join(self.cfg.working_dir, self.cfg.hmmer_dir, extract_out_name)
            with open(self._extract_out, 'w') as _file:
                _file.write(str(self))


[docs]    def best_hit(self):
        """
        Return the best hit among multiple hits
        """
        try:
            return self.hits[0]
        except IndexError:
            return None


[docs]    def _hit_start(self, line):
        """
        :param line: the line to parse
        :type line: string
        :return: True if it's the begining of a new hit in Hmmer raw output files.\
         False otherwise
        :rtype: boolean.
        """
        return line.startswith(">>")


[docs]    def _build_my_db(self, hmm_output):
        """
        Build the keys of a dictionary object to store sequence identifiers of hits.

        :param hmm_output: the path to the hmmsearch output to parse.
        :type hmm_output: string
        :return: a dictionary containing a key for each sequence id of the hits
        :rtype: dict
        """
        d = {}
        with open(hmm_output) as hmm_file:
            hits = (x[1] for x in groupby(hmm_file, self._hit_start) if x[0])
            for h in hits:
                d[self._parse_hmm_header(h)] = None
        return d


[docs]    def _fill_my_db(self, macsyfinder_idx, db):
        """
        Fill the dictionary with information on the matched sequences

        :param macsyfinder_idx: the path the macsyfinder index corresponding to the dataset
        :type  macsyfinder_idx: string
        :param db: the database containing all sequence id of the hits.
        :type db: dict
        """
        with open(macsyfinder_idx, 'r') as idx:
            for l in idx:
                seqid, length, rank = l.split(';')
                if seqid in db:
                    db[seqid] = (int(length), int(rank))


[docs]    def _parse_hmm_header(self, h_grp):
        """
        :param h_grp: the sequence of string return by groupby function representing the header of a hit 
        :type h_grp: sequence of string
        :returns: the sequence identifier from a set of lines that corresponds to a single hit
        :rtype: string
        """
        for line in h_grp:
            hit_id = line.split()[1]
        return hit_id


[docs]    def _parse_hmm_body(self, hit_id, gene_profile_lg, seq_lg, coverage_treshold, replicon_name, position_hit, i_evalue_sel, b_grp):
        """
        Parse the raw Hmmer output to extract the hits, and filter them with threshold criteria selected ("coverage_profile" and "i_evalue_select" command-line parameters) 

        :param hit_id: the sequence identifier
        :type hit_id: string 
        :param gene_profile_lg: the length of the profile matched
        :type gene_profile_lg: integer
        :param seq_lg: the length of the sequence
        :type seq_lg: integer
        :param coverage_treshold: the minimal coverage of the profile to be reached in the Hmmer alignment for hit selection
        :type coverage_treshold: float
        :param replicon_name: the identifier of the replicon
        :type replicon_name: string
        :param position_hit: the rank of the sequence matched in the input dataset file
        :type position_hit: integer
        :param i_evalue_sel: the maximal i-evalue (independent evalue) for hit selection
        :type i_evalue_sel: float
        :param b_grp: the Hmmer output lines to deal with (grouped by hit)
        :type b_grp: list of list of strings
        :returns: a set of hits
        :rtype: list of :class:`macsypy.report.Hit` objects

        """
        first_line = b_grp.next()
        if not first_line.startswith('   #    score'):
            return []
        else:
            hits = []
            for line in b_grp:
                if line[0] == '\n':
                    return hits
                elif line.startswith(" ---   ------ ----- --------"):
                    pass
                else:
                    fields = line.split()
                    try:
                        if(len(fields) > 1 and float(fields[5]) <= i_evalue_sel):
                            cov_profile = (float(fields[7]) - float(fields[6]) + 1) / gene_profile_lg
                            begin = int(fields[9])
                            end = int(fields[10])
                            cov_gene = (float(end) - float(begin) + 1) / seq_lg # To be added in Gene: sequence_length
                            if (cov_profile >= coverage_treshold):
                                i_eval = float(fields[5])
                                score = float(fields[2])
                                hits.append(Hit(self.gene,
                                                self.gene.system,
                                                hit_id,
                                                seq_lg,
                                                replicon_name,
                                                position_hit,
                                                i_eval,
                                                score,
                                                cov_profile,
                                                cov_gene,
                                                begin,
                                                end))
                    except ValueError, err:
                        msg = "Invalid line to parse :{0}:{1}".format(line, err)
                        _log.debug(msg)
                        raise ValueError(msg)


[docs]class GeneralHMMReport(HMMReport):
    """
    Handle HMM report. Extract a synthetic report from the raw hmmer output.
    Dedicated to any type of 'unordered' datasets.
    """
    #pass

[docs]    def extract(self):
        """
        Parse the output file of hmmer compute from an unordered genes base
        and produced a new synthetic report file.
        """

        with self._lock:
            # so the extract of a given HMM output is executed only once per run
            # if this method is called several times the first call induce the parsing of HMM out
            # the other calls do nothing
            if self.hits:
                return

            idx = Indexes(self.cfg)
            macsyfinder_idx = idx.find_my_indexes()
            my_db = self._build_my_db(self._hmmer_raw_out)
            self._fill_my_db(macsyfinder_idx, my_db)

            with open(self._hmmer_raw_out, 'r') as hmm_out:
                i_evalue_sel = self.cfg.i_evalue_sel
                coverage_treshold = self.cfg.coverage_profile
                gene_profile_lg = len(self.gene.profile)
                hmm_hits = (x[1] for x in groupby(hmm_out, self._hit_start))
                #drop summary
                hmm_hits.next()
                for hmm_hit in hmm_hits:
                    hit_id = self._parse_hmm_header(hmm_hit)
                    seq_lg, position_hit = my_db[hit_id]

                    #replicon_name = self.cfg. # Define a variable in further devt
                    replicon_name = "Unordered"

                    body = hmm_hits.next()
                    h = self._parse_hmm_body(hit_id, gene_profile_lg, seq_lg, coverage_treshold, replicon_name, position_hit, i_evalue_sel, body)
                    self.hits += h
                self.hits.sort()
                return self.hits


[docs]class OrderedHMMReport(HMMReport):
    """
    Handle HMM report. Extract a synthetic report from the raw hmmer output.
    Dedicated to 'ordered_replicon' datasets.
    """
    #pass

[docs]    def extract(self):
        """
        Parse the output file of Hmmer obtained from a search in an ordered set of sequences
        and produce a new synthetic report file.
        """

        with self._lock:
            # so the extract of a given HMM output is executed only once per run
            # if this method is called several times the first call induce the parsing of HMM out
            # the other calls do nothing
            if self.hits:
                return

            idx = Indexes(self.cfg)
            macsyfinder_idx = idx.find_my_indexes()
            my_db = self._build_my_db(self._hmmer_raw_out)
            self._fill_my_db(macsyfinder_idx, my_db)

            with open(self._hmmer_raw_out, 'r') as hmm_out:
                i_evalue_sel = self.cfg.i_evalue_sel
                coverage_treshold = self.cfg.coverage_profile
                gene_profile_lg = len(self.gene.profile)
                hmm_hits = (x[1] for x in groupby(hmm_out, self._hit_start))
                #drop summary
                hmm_hits.next()
                for hmm_hit in hmm_hits:
                    hit_id = self._parse_hmm_header(hmm_hit)
                    seq_lg, position_hit = my_db[hit_id]
                    replicon_name = RepliconDB.ordered_replicon_name

                    body = hmm_hits.next()
                    h = self._parse_hmm_body(hit_id, gene_profile_lg, seq_lg, coverage_treshold, replicon_name, position_hit, i_evalue_sel, body)
                    self.hits += h
                self.hits.sort()
                return self.hits


[docs]class GembaseHMMReport(HMMReport):
    """
    Handle HMM report. Extract a synthetic report from the raw hmmer output.
    Dedicated to 'gembase' format datasets.
    """

[docs]    def extract(self):
        """
        Parse the output file of Hmmer obtained from a search in a 'gembase' set of sequences
        and produce a new synthetic report file.
        """
        with self._lock:
            # so the extract of a given HMM output is executed only once per run
            # if this method is called several times the first call induce the parsing of HMM out
            # the other calls do nothing
            if self.hits:
                return

            idx = Indexes(self.cfg)
            macsyfinder_idx = idx.find_my_indexes()
            my_db = self._build_my_db(self._hmmer_raw_out)
            self._fill_my_db(macsyfinder_idx, my_db)

            with open(self._hmmer_raw_out, 'r') as hmm_out:
                i_evalue_sel = self.cfg.i_evalue_sel
                coverage_treshold = self.cfg.coverage_profile
                gene_profile_lg = len(self.gene.profile)
                hmm_hits = (x[1] for x in groupby(hmm_out, self._hit_start))
                #drop summary
                hmm_hits.next()
                for hmm_hit in hmm_hits:
                    hit_id = self._parse_hmm_header(hmm_hit)
                    seq_lg, position_hit = my_db[hit_id]
                    fields_hit = hit_id.split('_')
                    replicon_name = fields_hit[0]
                    body = hmm_hits.next()
                    h = self._parse_hmm_body(hit_id, gene_profile_lg, seq_lg, coverage_treshold, replicon_name, position_hit, i_evalue_sel, body)
                    self.hits += h
                self.hits.sort()
                return self.hits


[docs]class Hit(object):
    """
    Handle the hits filtered from the Hmmer search. The hits are instanciated by :py:meth:`HMMReport.extract` method
    """

[docs]    def __init__(self, gene, system, hit_id, hit_seq_length, replicon_name,
                 position_hit, i_eval, score, profile_coverage, sequence_coverage, begin_match, end_match):
        """
        :param gene: the gene corresponding to this profile
        :type gene: :class:`macsypy.gene.Gene` object
        :param system: the system to which this gene belongs
        :type system: :class:`macsypy.system.System` object
        :param hit_id: the identifier of the hit
        :type hit_id: string
        :param hit_seq_length: the length of the hit sequence
        :type hit_seq_length: integer
        :param replicon_name: the name of the replicon
        :type replicon_name: string
        :param position_hit: the rank of the sequence matched in the input dataset file
        :type position_hit: integer
        :param i_eval: the best-domain evalue (i-evalue, "independent evalue")
        :type i_eval: float
        :param score: the score of the hit
        :type score: float
        :param profile_coverage: percentage of the profile that matches the hit sequence
        :type profile_coverage: float
        :param sequence_coverage: percentage of the hit sequence that matches the profile
        :type sequence_coverage: float
        :param begin_match: where the hit with the profile starts in the sequence 
        :type begin_match: integer
        :param end_match: where the hit with the profile ends in the sequence 
        :type end_match: integer
        """
        self.gene = gene
        self.system = system
        self.id = hit_id
        self.seq_length = hit_seq_length
        self.replicon_name = replicon_name
        self.position = position_hit
        self.i_eval = i_eval
        self.score = score
        self.profile_coverage = profile_coverage
        self.sequence_coverage = sequence_coverage
        self.begin_match = begin_match
        self.end_match = end_match


[docs]    def __str__(self):
        """
        Print useful information on the Hit: regarding Hmmer statistics, and sequence information
        """
        return "%s\t%s\t%d\t%d\t%s\t%s\t%s\t%s\t%f\t%f\t%d\t%d\n" % (self.id,
                                                     self.replicon_name,
                                                     self.position,
                                                     self.seq_length,
                                                     self.gene.name,
                                                     self.system.name,
                                                     self.i_eval,
                                                     self.score,
                                                     self.profile_coverage, 
                                                     self.sequence_coverage,
                                                     self.begin_match,
                                                     self.end_match)


[docs]    def __cmp__(self, other):
        """
        Compare two Hits. If the sequence identifier is the same, do the comparison on the score. Otherwise, do it on alphabetical comparison of the sequence identifier.

        :param other: the hit to compare to the current object
        :type other: :class:`macsypy.report.Hit` object
        :return: the result of the comparison
        """
        if self.id == other.id:
            if not self.gene.is_homolog(other.gene): 
                _log.warning("Non homologs match: %s (%s) %s (%s) for %s"%(self.gene.name, self.system.name, other.gene.name, other.system.name, self.id))
            return cmp(self.score, other.score)
        else:
            return cmp(self.id, other.id)


[docs]    def __eq__(self, other):
        """
        Return True if two hits are totally equivalent, False otherwise.

        :param other: the hit to compare to the current object
        :type other: :class:`macsypy.report.Hit` object
        :return: the result of the comparison
        :rtype: boolean
        """
        return (
                self.gene.name == other.gene.name and
                self.system.name == other.system.name and
                self.id == other.id and
                self.seq_length == other.seq_length and
                self.replicon_name == other.replicon_name and
                self.position == other.position and
                self.i_eval == other.i_eval and
                self.score == other.score and
                self.profile_coverage == other.profile_coverage and
                self.sequence_coverage == other.sequence_coverage and
                self.begin_match == other.begin_match and
                self.end_match == other.end_match 
                )


[docs]    def get_position(self):
        """
        :returns: the position of the hit (rank in the input dataset file)
        :rtype: integer 
        """
        return self.position


[docs]    def get_syst_inter_gene_max_space(self):
        """
        :returns: the 'inter_gene_max_space' parameter defined for the gene of the hit
        :rtype: integer
        """
        return self.gene.system.inter_gene_max_space
```

### Quick search


Enter search terms or a module, class or function name.

### Navigation

- index
- modules |
- MacSyFinder documentation »
- Module code »

© Copyright 2014, Sophie Abby, Bertrand Néron.
Created using Sphinx 1.1.3.
