# Supplementary material for: MacSyFinder: A Program to Mine Genomes for Molecular Systems with an Application to CRISPR-Cas Systems
Source: PLoS One. 2014 Oct 17;9(10):e110726. doi: 10.1371/journal.pone.0110726 (PMC4201578; doi:10.1371/journal.pone.0110726)
Supplement: Data S1 — The MacSyFinder/MacSyView package (compressed tarball archive). (GZ) [file pone.0110726.s010.gz › macsyfinder-1.0.0-RC3/doc/_build/html/_modules/macsypy/search_genes.html]

macsypy.search\_genes — MacSyFinder 1.0.0-RC3 documentation


### Navigation

- index
- modules |
- MacSyFinder documentation »
- Module code »

# Source code for macsypy.search\_genes

```
# -*- coding: utf-8 -*-

################################################################################
# MacSyFinder - Detection of macromolecular systems in protein datasets        #
#               using systems modelling and similarity search.                 #
# Authors: Sophie Abby, Bertrand Néron                                         #
# Copyright © 2014  Institut Pasteur, Paris.                                   #
# See the COPYRIGHT file for details                                           #
#                                                                              #
# MacsyFinder is distributed under the terms of the GNU General Public License #
# (GPLv3). See the COPYING file for details.                                   #
################################################################################


import threading
import logging
_log = logging.getLogger('macsyfinder.' + __name__)
import signal
import sys
import shutil
import os.path
from report import GembaseHMMReport, GeneralHMMReport, OrderedHMMReport

[docs]def search_genes(genes, cfg):
    """
    For each gene of the list, use the corresponding profile to perform an Hmmer search, and parse the output
    to generate a HMMReport that is saved in a file after Hit filtering. These tasks are performed in parallel using threads.
    The number of workers can be limited by worker_nb directive in the config object or in the command-line with the \"-w\" option.

    :param genes: the genes to search in the input sequence dataset
    :type genes: list of :class:`macsypy.gene.Gene` objects
    :param cfg: the configuration object
    :type cfg: :class:`macsypy.config.Config` object
    """
    worker_nb = cfg.worker_nb
    if not worker_nb:
        worker_nb = len(genes)
    _log.debug("worker_nb = %d" % worker_nb)
    sema = threading.BoundedSemaphore(value = worker_nb)
    all_reports = []


    def stop(signum, frame):
        """stop the main process, its threads and subprocesses"""
        _log.critical('KILL all Processes')
        proc_grp_id = os.getpgid(0)
        os.killpg(proc_grp_id, signum)
        sys.exit(-1 * signum)


    signal.signal(signal.SIGTERM, stop)


    def search(gene, all_reports, sema):
        """
        Search gene in the database built from the input sequence file (execute \"hmmsearch\"), and produce a HMMReport

        :param gene: the gene to search
        :type gene: a :class:`macsypy.gene.Gene` object
        :param all_reports: a container to append the generated HMMReport objects
        :type all_reports: list of `macsypy.report.HMMReport` object (derived class depending on the input dataset type)
        :param sema: semaphore to limit the number of parallel workers
        :type sema: a threading.BoundedSemaphore
        """
        with sema:
            _log.info("search gene %s" % gene.name)
            profile = gene.profile
            report = profile.execute()
            if report:
                report.extract()
                report.save_extract()
                all_reports.append(report)


    def recover(gene, all_reports, cfg, sema):
        """
        Recover Hmmer output from a previous run, and produce a report

        :param gene: the gene to search
        :type gene: a :class:`macsypy.gene.Gene` object
        :param all_reports: a container to append the generated HMMReport object
        :type all_reports: list
        :param cfg: the configuration 
        :type cfg: :class:`macsypy.config.Config` object
        :param sema: semaphore to limit the number of parallel workers
        :type sema: a threading.BoundedSemaphore.
        :return: the list of all HMMReports (derived class depending on the input dataset type)
        :rtype: list of `macsypy.report.HMMReport` object
        """
        with sema:
            hmm_old_path = os.path.join(cfg.previous_run, cfg.hmmer_dir, gene.name + cfg.res_search_suffix)
            _log.info("recover hmm %s" % hmm_old_path)
            hmm_new_path = os.path.join(cfg.working_dir, cfg.hmmer_dir, gene.name + cfg.res_search_suffix)
            shutil.copy(hmm_old_path, hmm_new_path)
            gene.profile.hmm_raw_output = hmm_new_path
            if cfg.db_type == 'gembase':
                report = GembaseHMMReport(gene, hmm_new_path, cfg )
            elif cfg.db_type == 'ordered_replicon':
                report = OrderedHMMReport(gene, hmm_new_path, cfg )
            else:
                report = GeneralHMMReport(gene, hmm_new_path, cfg )
            if report:
                report.extract()
                report.save_extract()
                all_reports.append(report)

    #there is only one instance of gene per name but the same instance can be
    #in all genes several times
    #hmmsearch and extract should be execute only once per run
    #so I uniquify the list of gene
    genes = set(genes)
    _log.debug("start searching genes")

    previous_run = cfg.previous_run
    for gene in genes:
        if previous_run and os.path.exists(os.path.join(previous_run, cfg.hmmer_dir, gene.name + cfg.res_search_suffix)):
            t = threading.Thread(target = recover, args = (gene, all_reports, cfg, sema))
        else:
            t = threading.Thread(target = search, args = (gene, all_reports, sema))
        t.start()

    main_thread = threading.currentThread()   
    while threading.activeCount() > 1:
        for t in threading.enumerate():
            if t is main_thread:
                continue
            t.join(10)

    _log.debug("end searching genes")
    return all_reports
```

### Quick search


Enter search terms or a module, class or function name.

### Navigation

- index
- modules |
- MacSyFinder documentation »
- Module code »

© Copyright 2014, Sophie Abby, Bertrand Néron.
Created using Sphinx 1.1.3.
