# Supplementary material for: MacSyFinder: A Program to Mine Genomes for Molecular Systems with an Application to CRISPR-Cas Systems
Source: PLoS One. 2014 Oct 17;9(10):e110726. doi: 10.1371/journal.pone.0110726 (PMC4201578; doi:10.1371/journal.pone.0110726)
Supplement: Data S1 — The MacSyFinder/MacSyView package (compressed tarball archive). (GZ) [file pone.0110726.s010.gz › macsyfinder-1.0.0-RC3/doc/_build/html/_modules/macsypy/search_systems.html]

macsypy.search\_systems — MacSyFinder 1.0.0-RC3 documentation


### Navigation

- index
- modules |
- MacSyFinder documentation »
- Module code »

# Source code for macsypy.search\_systems

```
# -*- coding: utf-8 -*-

################################################################################
# MacSyFinder - Detection of macromolecular systems in protein datasets        #
#               using systems modelling and similarity search.                 #
# Authors: Sophie Abby, Bertrand Néron                                         #
# Copyright © 2014  Institut Pasteur, Paris.                                   #
# See the COPYRIGHT file for details                                           #
#                                                                              #
# MacsyFinder is distributed under the terms of the GNU General Public License #
# (GPLv3). See the COPYING file for details.                                   #
################################################################################


import logging
import abc
import os.path
from collections import Counter, OrderedDict
import itertools
import operator
import json

from macsypy_error import MacsypyError, SystemDetectionError
from database import RepliconDB
from system import system_bank

_log = logging.getLogger('macsyfinder.' + __name__)

_log_out = logging.getLogger('macsyfinder.out')

[docs]class ClustersHandler(object):
    """
    Deals with sets of clusters found in a dataset. Conceived to store only clusters from a same replicon.
    """

[docs]    def __init__(self):
        """
        :param cfg: The configuration object built from default and user parameters.
        :type cfg: :class:`macsypy.config.Config` 
        """
        self.clusters = []
        self.replicon_name = ""

def add(self, cluster):
        if not self.replicon_name:
            self.replicon_name = cluster.replicon_name
            cluster.save()
            self.clusters.append(cluster)
        elif self.replicon_name == cluster.replicon_name:
            cluster.save()
            self.clusters.append(cluster)
        else:
            msg = "Attempting to add a cluster in a ClustersHandler dedicated to another replicon !"
            for c in self.clusters:
                msg += str(c)
            msg += "To add: {0}".format(cluster)
            _log.critical(msg)
            raise SystemDetectionError(msg)

    def __str__(self):
        to_print = ""
        for cluster in self.clusters:
            to_print += str(cluster)

        return to_print

[docs]    def circularize(self, rep_info, end_hits, systems_to_detect):
        """
        This function takes into account the circularity of the replicon by merging clusters when appropriate (typically at replicon's ends). 
        It has to be called only if the replicon_topology is set to \"circular\".

        :param rep_info: an entry extracted from the :class:`macsypy.database.RepliconDB`
        :type rep_info: a namedTuple "RepliconInfo" :class:`macsypy.database.RepliconInfo`
        :param end_hits: a set of hits at ends of the replicon that were not introduced in clusters, and that might be part of a system overlapping the two "ends" of the replicon
        :type end_hits: a list of :class:`macsypy.report.Hit`
        :param systems_to_detect: the set of systems to detect in this run
        :type systems_to_detect: a list of :class:`macsypy.system.System
        """
        # We assume this function is called when appropriate (i.e. for circular replicons)

        pos_min = rep_info.min
        pos_max = rep_info.max

        check_clust = True
        msg = "\n----------------------------------------\n--- Handling of replicon circularity ---\n"

        if len(self.clusters) >= 1:
            # Here they might be the same !
            clust_first = self.clusters[0]
            clust_last = self.clusters[len(self.clusters) - 1]

            #check_clust = True
            # Case 1: none of the two end hits is part of a cluster => they might form a new cluster together. 
            if len(end_hits) == 2:
                first = end_hits[0]
                second = end_hits[1]
                #if(first.gene.position > second.gene.position): # Genes do not have positions ! But Hits do!
                if(first.position > second.position):
                    tmp = first
                    first = second
                    #second = first
                    second = tmp
                dist = second.position - pos_min + pos_max - first.position 
                if(dist <= max(first.gene.inter_gene_max_space, second.gene.inter_gene_max_space)):
                    # If true, this means that there is no need to circularize other clusters as this cluster already overlaps the "Ori". => check_clust is thus set to False

                    msg += "--- Two hits at both ends of the replicon form a new cluster.\n"

                    new_clust = Cluster(systems_to_detect)
                    new_clust.add(second)
                    new_clust.add(first)
                    new_clust.save()
                    self.clusters.append(new_clust)
                    check_clust = False

            if check_clust:
                for h in end_hits:
                    # Two cases: we are at one end, or the other
                    if h.position > clust_first.hits[0].position:
                        # Case 2: The end hit is at the terminal end of the replicon. Try to cluster it with the 1st stored cluster.
                        #print "The end hit is at the TERMINAL end of the replicon. Test if it must be clustered with the 1st stored cluster."
                        dist_end_hit = clust_first.begin - pos_min + pos_max - h.position # OK?
                        if(dist_end_hit <= max(clust_first.hits[0].gene.inter_gene_max_space, h.gene.inter_gene_max_space)):
                            check_clust = False
                            #print "Cluster them !"

                            msg += "--- A hit is at the TERMINAL end of the replicon, and must be clustered with the 1st stored cluster.\n"

                            new_clust = Cluster(systems_to_detect)
                            new_clust.add(h)
                            for hit_clust in clust_first.hits:
                                new_clust.add(hit_clust)

                            new_clust.save()
                            self.clusters.pop(0)
                            self.clusters.append(new_clust)
                    else:
                        # Case 3: The end hit is at the initial end of the replicon. Try to cluster it with the last stored cluster.
                        #print "The end hit is at the INITIAL end of the replicon. Test if it must be clustered with the last stored cluster."
                        dist_end_hit = h.position - pos_min + pos_max - clust_last.end 
                        if(dist_end_hit <= max(clust_last.hits[len(clust_last.hits) - 1].gene.inter_gene_max_space, h.gene.inter_gene_max_space)):
                            check_clust = False
                            #print "Cluster them !"

                            msg += "--- A hit is at the INITIAL end of the replicon, and must be clustered with the last stored cluster.\n"

                            clust_last.add(h)
                            clust_last.save(True) # Force to re-save the updated cluster

        if check_clust and (len(self.clusters) > 1):
            clust_first = self.clusters[0]
            clust_last = self.clusters[len(self.clusters)-1]

            # Case 4: when putative end hits have been dealt with, now remain cases where clusters are at both ends, and might be fused.                     
            dist_clust = clust_first.begin - pos_min + pos_max - clust_last.end

            if (dist_clust <= max(clust_first.hits[0].gene.inter_gene_max_space, clust_last.hits[len(clust_last.hits) - 1].gene.inter_gene_max_space)):
                # Need to circularize !
                #print "A cluster needs to be \"circularized\" ! "
                msg = "--- Two clusters should be merged into a new cluster \"circularized\" !\n"
                # The "1st" cluster on the replicon is removed, as its hits will be appended to the "last" cluster on the replicon.
                #self.clusters.pop(0) 
                for h in clust_first.hits:
                    clust_last.add(h)
                self.clusters.pop(0) 
                clust_last.save(True) # Force to re-save the updated cluster
                #print clust_last
                msg += str(clust_last)

        msg += "----------------------------------------"
        _log.info(msg)


[docs]class Cluster(object):
    """
    Stores a set of contiguous hits. The Cluster object can have different states regarding 
    its content in different genes' systems: 

      - ineligible: not a cluster to analyze
      - clear: a single system is represented in the cluster
      - ambiguous: several systems are represented in the cluster => might need a disambiguation

    """

[docs]    def __init__(self, systems_to_detect):
        """
        :param systems_to_detect: the list of systems to be detected in this run
        :type systems_to_detect: a list of :class:`macsypy.system.System`
        """
        self.hits = []
        self.systems_to_detect = systems_to_detect # NEW
        self.systems = {}
        self.replicon_name = ""
        self.begin = 0
        self.end = 0
        self._state = ""
        self._putative_system = ""
        self._compatible_systems = [] # NEW!


[docs]    def __len__(self):
        """
        :return: the length of the Cluster, *i.e.*, the number of hits stored in it
        :rtype: integer
        """
        return len(self.hits)


[docs]    def __str__(self):
        """
        print of the Cluster's hits stored in terms of components, and corresponding sequence identifier and positions
        """
        pos = []
        seq_ids = []
        gene_names = []
        for h in self.hits:
            pos.append(h.position)
            seq_ids.append(h.id)
            gene_names.append(h.gene.name)
        clear = '?'
        if self.state == "clear":
            clear = ''
        return "--- Cluster {0} {1} ---\n{2}\n{3}\n{4}".format(self.putative_system,
                                                               clear, 
                                                               seq_ids, 
                                                               gene_names, 
                                                               pos)

@property

[docs]    def state(self):
        """
        :return: the state of the Cluster of hits
        :rtype: string
        """
        return self._state

@property

[docs]    def putative_system(self):
        """
        :return: the name of the putative system represented by the cluster
        :rtype: string
        """
        return self._putative_system

@property

[docs]    def compatible_systems(self):
        """
        :return: the list of the names of compatible systems represented by the cluster
        :rtype: string
        """
        return self._compatible_systems


[docs]    def add(self, hit):
        """
        Add a Hit to a Cluster.
        Hits are always added at the end of the cluster (appended to the list of hits). Thus, 'begin' and 'end' positions of the Cluster are always the position of the 1st and of the last hit respectively.

        :param hit: the Hit to add
        :type hit: a :class:`macsypy.report.Hit`
        :raise: a :class:`macsypy.macsypy_error.SystemDetectionError`
        """
        # need to update cluster bounds
        if len(self.hits) == 0:
            self.begin = hit.get_position()
            self.end = self.begin
            self.replicon_name = hit.replicon_name
            self.hits.append(hit)
        else:
            if(self.replicon_name == hit.replicon_name):
                # To be updated !! make this work also with "circularized" clusters
                # The end position only is updated, as Hits are always appended.
                self.end = hit.get_position()
                self.hits.append(hit)
            else:
                msg = "Attempting to gather in a cluster hits from different replicons ! "
                _log.critical(msg)
                raise SystemDetectionError(msg)


[docs]    def save(self, force=False):
        """
        Check the status of the cluster regarding systems which have hits in it. 
        Update systems represented, and assign a putative system (self._putative_system), which is the system with most hits in the cluster. 
        The systems represented are stored in a dictionary in the self.systems variable. 
        The execution of this function can be forced, even if it has already run for the cluster with the option force=True.
        """
        if not self.putative_system or force:
            # First compute the "Majoritary" system
            systems = {} # Counter of occcurrences of systems in the cluster (keys are systems' names)
            genes = []
            #systems_object={} # Store systems object. # To be replaced by "system_bank"? Yep ! done

            # Version with hits "reference" systems
            for h in self.hits:
                syst = h.system.name
                if not systems.has_key(syst):
                    systems[syst] = 1
                    #systems_object[syst]=h.system
                else:
                    systems[syst] += 1
                if genes.count(h.gene.name) == 0:
                    genes.append(h.gene.name)

            self.systems = systems

            # Useless ?! Or change? 
            max_syst = 0
            tmp_syst_name = ""
            for x, y in systems.iteritems():
                if y >= max_syst:
                    tmp_syst_name = x
                    max_syst = y

            self._putative_system = tmp_syst_name # Remove cause useless?

            # NEW Version with hits all "compatible" systems
            systems_compat = {} # Counter of occcurrences of COMPATIBLE (-extended- list of) systems in the cluster. Keys are systems' names
            for h in self.hits:
                #syst_list=h.gene.get_compatible_systems(self.systems_to_detect) # Need the list of systems (obj!) to be detected... in the cfg?
                # Now exclude forbidden genes from those that define the list of compatible systems
                syst_list = h.gene.get_compatible_systems(self.systems_to_detect, False) # Need the list of systems (obj!) to be detected... in the cfg? # tmp before nope
                #syst_list=h.gene.get_compatible_systems(self.systems_to_detect, True) # Need the list of systems (obj!) to be detected... in the cfg? # tmp before yep
                for syst in syst_list:
                    syst_name = syst.name
                    if not systems_compat.has_key(syst_name):
                        systems_compat[syst_name] = 1
                        #systems_object[syst]=h.system
                    else:
                        systems_compat[syst_name] += 1
                    if genes.count(h.gene.name) == 0:
                        genes.append(h.gene.name)

            #print systems_compat
            # We sort the list of compatible systems per decreasing nb of systems occurrences
            systems_compat = OrderedDict(sorted(systems_compat.items(), reverse = True, key = lambda t: t[1]))
            #print systems_compat
            #print self

            if len(genes) == 1 and self.hits[0].gene.loner == False:
                self._state = "ineligible"
            else:
                # Check for foreign "accessory" genes... Might increase nb of systems predicted in the cluster, even if they are tolerated in the cluster. 
                # Also deal with foreign "exchangeable" genes for the same reasons... NB !! Maybe just not add the system to the list if exchangeable?  
                #if len(systems.keys()) == 1:
                if len(systems_compat.keys()) == 1:
                    self._state = "clear"
                    #syst = systems.keys()[0]
                    syst = systems_compat.keys()[0]
                    #print syst
                    self._putative_system = syst
                    #self._compatible_systems.append(system_bank[syst])
                    # Store only compatible systems that are searched for !!
                    self._compatible_systems.append(syst)
                else:
                    # Check for foreign "accessory" genes regarding the majoritary system... They might increase nb of systems predicted in the cluster artificially, even if they are tolerated in the cluster. For that need to scan again all hits and ask wether they are accessory foreign genes. 
                    def try_system(hits, putative_system, counter_systems_in_clust):
                        """
                        Test if the putative_system is compatible with the systems of hits (counter_systems_in_clust)

                        :param hits: a list of hits
                        :type hits: a list of :class:`macsypy.report.Hit`
                        :param putative_system: the name of a putative system to consider
                        :type putative_system: string
                        :param counter_systems_in_clust: a dictionary with systems occurrences when exploring the hits
                        :type counter_systems_in_clust: a dictionary with systems' names as keys, and counts as values
                        :return: the "state" of the set of hits regarding the putative system
                        :rtype: string

                        """
                        #foreign_accessory = 0
                        auth = 0 # counts nb of hits that are authorized in the putative system
                        #forbid = 0
                        #print "Unclear state with multiple systems to deal with..."
                        #print systems
                        for h in hits:
                            # Exclude the consideration of "forbidden" genes !
                            #if h.gene.is_authorized(system_bank[putative_system], False): # tmp before nope
                            if h.gene.is_authorized(system_bank[putative_system], True): # No! forbidden genes that are defined in system has to be considered!
                                auth += 1
                            #if h.gene.is_forbidden(system_bank[putative_system]):
                            #    forbid +=1
                                                    
                            if auth == len(hits):
                                state = "clear"
                                #print "clear %s"%putative_system
                            else:
                                state = "ambiguous"
                                #print "ambiguous %s"%putative_system

                        """
                        if forbid == 0:
                            if auth == len(hits):
                                state = "clear"
                                #print "clear %s"%putative_system
                            else:
                                state = "ambiguous"
                                #print "ambiguous %s"%putative_system
                        else:
                            state = "ineligible"
                            #print "ineligible %s"%putative_system
                        """    
                        return state

                    # Sort systems to consider by decreasing counts.
                    cluster_compatible_systems = []
                    for putative_system in systems_compat.keys():
                        # Add that it has to be done first from the most rep systems by decreasing order of systems.values.
                        state = try_system(self.hits, putative_system, systems_compat)
                        if state == "clear":
                            #print "BUENO SYSTEMO %s"%putative_system
                            #self._state="clear" 
                            #self._putative_system=putative_system 
                            #break
                            cluster_compatible_systems.append(putative_system)
                        #else:
                        #    self._state="ambiguous" # tmp before nope
                        #    # Aoutch in this case no putative_system?!
                        #    print "YAPABON...%s"%putative_system
                    if len(cluster_compatible_systems) >= 1:
                        #print cluster_compatible_systems
                        self._state = "clear"
                        self._putative_system = cluster_compatible_systems[0]
                        self._compatible_systems = cluster_compatible_systems
                    else:
                        self._state = "ambiguous"
        #print self._compatible_systems


[docs]class SystemNameGenerator(object):
    """
    Creates and stores the names of detected systems. Ensures the uniqueness of the names.
    """
    name_bank = {}

[docs]    def getSystemName(self, replicon, system):
        """
        Generates a unique system name based on the replicon's name and the system's name.

        :param replicon: the replicon name
        :type replicon: string
        :param system: the system name
        :type system: string
        :return: a unique system name
        :rtype: string
        """
        basename = self._computeBasename(replicon, system)
        if basename in self.name_bank:
            self.name_bank[basename] += 1
        else:
            self.name_bank[basename] = 1

        system_name =  basename + str(self.name_bank[basename])
        return system_name


[docs]    def _computeBasename(self, replicon, system):
        """
        Computes the base name to be used for unique name generation

        :param replicon: the replicon name
        :type replicon: string
        :param system: the system name
        :type system: string
        :return: the base name
        :rtype: string
        """
        return "{0}_{1}_".format(replicon, system)

system_name_generator = SystemNameGenerator()

[docs]class SystemOccurence(object):
    """
    This class is instantiated for a specific system that has been asked for detection. It can be filled step by step with hits. 
    A decision can then be made according to the parameters defined *e.g.* quorum of genes. 

    The SystemOccurence object has a "state" parameter, with the possible following values: 
      - "empty" if the SystemOccurence has not yet been filled with genes of the decision rule of the system
      - "no_decision" if the filling process has started but the decision rule has not yet been applied to this occurence
      - "single_locus"
      - "multi_loci"
      - "uncomplete"

    """

[docs]    def __init__(self, system):
        """
        :param system: the system to \"fill\" with hits.
        :type system: :class:`macsypy.system.System` 
        """
        self.system = system
        self.system_name = system.name

        # Variables to be updated during the system detection 
        self.valid_hits = [] # validSystemHit are stored with the "fill_with" function, and ready for extraction in case of a positive detection

        self.loci_positions = [] # list of tuples

        self._state = "empty"
        self.nb_cluster = 0
        self._nb_syst_genes = 0
        self.unique_name = ""

        # System definition
        # Make those attributes non modifiable?
        self.mandatory_genes = {}
        self.exmandatory_genes = {} # List of 'exchanged' mandatory genes

        # New ! Add of a list of "multi_system" genes, fed only from mandatory and accessory genes from the actual system (and not 'exchanged')
        self.multi_syst_genes = {}

        for g in system.mandatory_genes:
            self.mandatory_genes[g.name] = 0
            if g.exchangeable:
                homologs = g.get_homologs()
                analogs = g.get_analogs()
                for ex in homologs + analogs:
                    self.exmandatory_genes[ex.name] = g.name
            if g.multi_system:
                self.multi_syst_genes[g.name] = 0

        self.accessory_genes = {}
        self.exaccessory_genes = {} # List of 'exchanged' accessory genes
        for g in system.accessory_genes:
            self.accessory_genes[g.name] = 0
            if g.exchangeable:
                homologs = g.get_homologs()
                analogs = g.get_analogs()
                for ex in homologs + analogs:
                    self.exaccessory_genes[ex.name] = g.name
            if g.multi_system:
                self.multi_syst_genes[g.name] = 0

        self.forbidden_genes = {}
        self.exforbidden_genes = {} # List of 'exchanged' forbidden genes
        for g in system.forbidden_genes:
            self.forbidden_genes[g.name] = 0
            if g.exchangeable:
                homologs = g.get_homologs()
                analogs = g.get_analogs()
                for ex in homologs + analogs:
                    self.exforbidden_genes[ex.name] = g.name
            # Forbidden genes do not play a role in the system, thus they do not have the multi_system feature
            #if g.multi_system:
            #    self.multi_syst_genes[g.name] = 0


[docs]    def get_gene_ref(self, gene):
        """
        :param gene: the gene to get it's gene reference
        :type gene: :class:`macsypy.gene.Gene`, or :class:`macsypy.gene.Homolog` or :class:`macsypy.gene.Analog` object
        :return: object :class:`macsypy.gene.Gene` or None 
        :rtype: :class:`macsypy.gene.Gene` object or None
        :raise: KeyError if the system does not contain any gene gene.
        """
        return self.system.get_gene_ref(gene)


[docs]    def __str__(self):
        """
        :return: Information of the component content of the SystemOccurence.
        :rtype: string
        """
        out = ""
        if self.mandatory_genes: 
            out += "Mandatory genes: \n"
            for k, g in self.mandatory_genes.iteritems():
                out += "{0}\t{1:d}\n".format(k, g)
        if self.accessory_genes:
            out += "Accessory genes: \n"
            for k, g in self.accessory_genes.iteritems():
                out += "{0}\t{1:d}\n".format(k, g)
        if self.forbidden_genes:
            out += "Forbidden genes: \n"
            for k, g in self.forbidden_genes.iteritems():
                out += "{0}\t{1:d}\n".format(k, g)
        # NEW
        if self.multi_syst_genes:
            out += "Multi_syst genes:\n"
            for k, g in self.multi_syst_genes.iteritems():
                out += "{0}\t{1:d}\n".format(k, g)
        return out


[docs]    def get_gene_counter_output(self, forbid_exclude = False):
        """
        :param forbid_exclude: exclude the forbidden components if set to True. False by default.
        :type forbid_exclude: boolean
        :returns: A dictionary ready for printing in system summary, \
         with genes (mandatory, accessory and forbidden if specified) occurences in the system occurrence.
        """
        out = ""
        out += str(self.mandatory_genes)
        out += "\t{0}".format(self.accessory_genes)
        if not forbid_exclude:
            out += "\t{0}".format(self.forbidden_genes)
        else:
            out += "\t{}"
        return out

@property

[docs]    def state(self):
        """
        :return: the state of the systemOccurrence.
        :rtype: string
        """
        return self._state


[docs]    def get_system_unique_name(self, replicon_name):
        """
        Attributes unique name to the system occurrence with the class :class:`macsypy.search_systems.SystemNameGenerator`.
        Generates the name if not already set. 

        :param replicon_name: the name of the replicon
        :type replicon_name: string
        :return: the unique name of the :class:`macsypy.search_systems.SystemOccurence`
        :rtype: string
        """
        if not self.unique_name:
            self.unique_name = system_name_generator.getSystemName(replicon_name, self.system_name)
        return self.unique_name


[docs]    def get_system_name_unordered(self, suffix="_putative"):
        """
        Attributes a name to the system occurrence for an "unordered" dataset => generating a generic name based on the system name and the suffix given. 

        :param suffix: the suffix to be used for generating the systemOccurrence's name
        :type suffix: string
        :return: a name for a system in an "unordered" dataset to the :class:`macsypy.search_systems.SystemOccurence`
        :rtype: string
        """
        return self.system_name + suffix


[docs]    def compute_system_length(self, rep_info):
        """
        Returns the length of the system, all loci gathered, in terms of protein number (even those not matching any system gene)

        :param rep_info: an entry extracted from the :class:`macsypy.database.RepliconDB`
        :type rep_info: a namedTuple "RepliconInfo" :class:`macsypy.database.RepliconInfo`
        :rtype: integer
        """
        length = 0
        # To be updated to deal with "circular" clusters
        for(begin, end) in self.loci_positions:
            if begin <= end:
                length += (end - begin + 1)
            elif rep_info.topology == "circular":
                locus_length = end - begin + rep_info.max - rep_info.min + 2
                length += locus_length
            else:
                msg = "Inconsistency in locus positions in the case of a linear replicon. The begin position of a locus cannot be higher than the end position. \n"
                msg += "Problem with locus found with positions begin: {0:d} end: {1:d}".format(begin, end)
                _log.critical(msg)
                raise SystemDetectionError(msg)
        return length

@property

[docs]    def nb_syst_genes(self):
        """
        This value is set after a decision was made on the system in :func:`macsypy.search_systems.SystemOccurence:decision_rule`

        :return: the number of mandatory and accessory genes with at least one occurence (number of different accessory genes)
        :rtype: integer
        """
        return self._nb_syst_genes

def compute_nb_syst_genes(self):
        return self.count_genes(self.mandatory_genes) + self.count_genes(self.accessory_genes)

    def compute_nb_syst_genes_tot(self):
        return self.count_genes_tot(self.mandatory_genes) + self.count_genes_tot(self.accessory_genes)

[docs]    def count_genes(self, gene_dict):
        """
        Counts the number of genes with at least one occurrence in a dictionary with a counter of genes. 

        :param gene_dict: a dictionary with gene's names as keys and number of occurrences as values
        :type gene_dict: dict
        :rtype: integer
        """
        total = 0
        for v in gene_dict.values():
            if v > 0:
                total += 1
        return total


[docs]    def count_genes_tot(self, gene_dict):
        """
        Counts the number of matches in a dictionary with a counter of genes, independently of the nb of genes matched.

        :param gene_dict: a dictionary with gene's names as keys and number of occurrences as values
        :type gene_dict: dict
        :rtype: integer
        """
        total = 0
        for v in gene_dict.values():
            total += v
        return total


[docs]    def compute_missing_genes_list(self, gene_dict):
        """
        :param gene_dict: a dictionary with gene's names as keys and number of occurrences as values
        :type gene_dict: dict
        :returns: the list of genes with no occurence in the gene counter. 
        :rtype: list
        """
        missing = []
        for k, v in gene_dict.iteritems():
            if v == 0:
                missing.append(k)
        return missing


[docs]    def count_missing_genes(self, gene_dict):
        """
        Counts the number of genes with no occurence in the gene counter.

        :param gene_dict: a dictionary with gene's names as keys and number of occurrences as values
        :type gene_dict: dict
        :rtype: integer
        """
        return len(self.compute_missing_genes_list(gene_dict))


[docs]    def is_complete(self):
        """
        Test for SystemOccurrence completeness.

        :returns: True if the state of the SystemOccurrence is "single_locus" or "multi_loci", False otherwise.
        :rtype: boolean
        """
        if self.state == "single_locus" or self.state == "multi_loci":
            return True
        else:
            return False


[docs]    def get_summary_header(self):
        """
        Returns a string with the description of the summary returned by self.get_summary()

        :rtype: string
        """
        return "#Replicon_name\tSystem_Id\tReference_system\tSystem_status\tNb_loci\tNb_Ref_mandatory\tNb_Ref_accessory\tNb_Ref_Genes_detected_NR\tNb_Genes_with_match\tSystem_length\tNb_Mandatory_NR\tNb_Accessory_NR\tNb_missing_mandatory\tNb_missing_accessory\tList_missing_mandatory\tList_missing_accessory\tLoci_positions\tOccur_Mandatory\tOccur_Accessory\tOccur_Forbidden"


[docs]    def get_summary(self, replicon_name, rep_info):
        """
        Gives a summary of the system occurrence in terms of gene content and localization.

        :param replicon_name: the name of the replicon
        :type replicon_name: string
        :param rep_info: an entry extracted from the :class:`macsypy.database.RepliconDB`
        :type rep_info: a namedTuple "RepliconInfo" :class:`macsypy.database.RepliconInfo`        
        :return: a tabulated summary of the :class:`macsypy.search_systems.SystemOccurence`
        :rtype: string
        """

        report_str = replicon_name + "\t" + self.get_system_unique_name(replicon_name)
        report_str += "\t{0}".format(self.system_name)
        report_str += "\t{0}".format(self.state)
        # Nb of loci included to fill the system occurrence
        report_str += "\t{0:d}".format(self.nb_cluster)
        # Nb mandatory_genes in the definition of the system
        report_str += "\t{0:d}".format(len(self.mandatory_genes))
         # Nb accessory_genes in the definition of the system                               
        report_str += "\t{0:d}".format(len(self.accessory_genes))
        # Nb syst genes NR
        report_str += "\t{0:d}".format(self.nb_syst_genes)
        # Nb syst genes matched
        report_str += "\t{0:d}".format(self.compute_nb_syst_genes_tot())
        #report_str += "\t%d"%self.compute_system_length() # The total length of the locus in protein number, delimited by hits for profiles of the system.
        # The total length of the locus in protein number, delimited by hits for profiles of the system.
        report_str += "\t{0:d}".format(self.compute_system_length(rep_info))
        # Nb mandatory_genes matched at least once
        report_str += "\t{0:d}".format(self.count_genes(self.mandatory_genes))
        # Nb accessory_genes matched at least once
        report_str += "\t{0:d}".format(self.count_genes(self.accessory_genes))

        missing_mandatory = self.compute_missing_genes_list(self.mandatory_genes)
        missing_accessory = self.compute_missing_genes_list(self.accessory_genes)

        # Nb mandatory_genes with no occurrence in the system
        report_str += "\t{0:d}".format(len(missing_mandatory))
        # Nb accessory_genes with no occurrence in the system
        report_str += "\t{0:d}".format(len(missing_accessory))
        # List of mandatory genes with no occurrence in the system
        report_str += "\t{0}".format(missing_mandatory)
        # List of accessory genes with no occurrence in the system
        report_str += "\t{0}".format(missing_accessory)
        
        # The positions of the loci (begin, end) as delimited by hits for profiles of the system.
        report_str += "\t{0}".format(self.loci_positions)
        # A dico per type of gene 'Mandatory, Accessory, Forbidden' with gene occurrences in the system
        report_str += "\t{0}".format(self.get_gene_counter_output())

        return report_str


[docs]    def get_summary_unordered(self, replicon_name):
        """
        Gives a summary of the system occurrence in terms of gene content only (specific of "unordered" datasets).

        :param replicon_name: the name of the replicon
        :type replicon_name: string
        :return: a tabulated summary of the :class:`macsypy.search_systems.SystemOccurence`
        :rtype: string
        """

        #report_str = replicon_name+"\t"+self.get_system_unique_name(replicon_name)
        # No replicon name for unordered... get it from the config object in future developments... 
        report_str = replicon_name+"\t"+self.get_system_name_unordered()
        report_str += "\t%s"%self.system_name
        report_str += "\t%s"%self.state

        #report_str+="\t%d"%self.nb_cluster # Nb of loci included to fill the system occurrence
        report_str += "\tNone"# No loci in unordered
        report_str += "\t%d"%len(self.mandatory_genes) # Nb mandatory_genes in the definition of the system
        report_str += "\t%d"%len(self.accessory_genes) # Nb accessory_genes in the definition of the system
        report_str += "\t%d"%self.nb_syst_genes # Nb syst genes NR
        report_str += "\t%d"%self.compute_nb_syst_genes_tot() # Nb syst genes matched

        #report_str+="\t%d"%self.compute_system_length(rep_info) # The total length of the locus in protein number, delimited by hits for profiles of the system.
        report_str += "\tNone" # No loci in unordered

        report_str += "\t%d"%self.count_genes(self.mandatory_genes) # Nb mandatory_genes matched at least once
        report_str += "\t%d"%self.count_genes(self.accessory_genes) # Nb accessory_genes matched at least once

        missing_mandatory = self.compute_missing_genes_list(self.mandatory_genes)        
        missing_accessory = self.compute_missing_genes_list(self.accessory_genes)

        report_str += "\t%d"%len(missing_mandatory) # Nb mandatory_genes with no occurrence in the system
        report_str += "\t%d"%len(missing_accessory) # Nb accessory_genes with no occurrence in the system
        report_str += "\t%s"%str(missing_mandatory) # List of mandatory genes with no occurrence in the system
        report_str += "\t%s"%str(missing_accessory) # List of accessory genes with no occurrence in the system

        #report_str+="\t%s"%self.loci_positions # The positions of the loci (begin, end) as delimited by hits for profiles of the system.
        report_str += "\tNone" # No loci in unordered
        report_str += "\t%s"%self.get_gene_counter_output(True) # A dico per type of gene 'Mandatory, Accessory, Forbidden' with gene occurrences in the system

        return report_str


[docs]    def fill_with_cluster(self, cluster):
        """
        Adds hits from a cluster to a system occurence, and check which are their status according to the system definition.
        Set the system occurence state to "no_decision" after calling of this function.

        :param cluster: the set of contiguous genes to treat for :class:`macsypy.search_systems.SystemOccurence` inclusion. 
        :type cluster: :class:`macsypy.search_systems.Cluster`
        """
        included = True
        self._state = "no_decision"
        for hit in cluster.hits:
            # Need to check first that this cluster is eligible for system inclusion
            # Stores hits for system extraction (positions, sequences) when complete.

            if hit.gene.is_mandatory(self.system):
                self.mandatory_genes[hit.gene.name] += 1
                valid_hit = validSystemHit(hit, self.system_name, "mandatory")
                self.valid_hits.append(valid_hit)
                # NEW
                if hit.gene.multi_system:
                    self.multi_syst_genes[hit.gene.name] += 1
            elif hit.gene.is_accessory(self.system):
                self.accessory_genes[hit.gene.name] += 1
                valid_hit = validSystemHit(hit, self.system_name, "accessory")
                self.valid_hits.append(valid_hit)
                # NEW
                if hit.gene.multi_system:
                    self.multi_syst_genes[hit.gene.name] += 1
            elif hit.gene.is_forbidden(self.system):
                self.forbidden_genes[hit.gene.name] += 1
                included = False
            else:
                if hit.gene.name in self.exmandatory_genes.keys():
                    self.mandatory_genes[self.exmandatory_genes[hit.gene.name]] += 1
                    valid_hit = validSystemHit(hit, self.system_name, "mandatory")
                    self.valid_hits.append(valid_hit)
                elif hit.gene.name in self.exaccessory_genes.keys():
                    self.accessory_genes[self.exaccessory_genes[hit.gene.name]] += 1
                    valid_hit = validSystemHit(hit, self.system_name, "accessory")
                    self.valid_hits.append(valid_hit)
                # NEW: exforbidden_genes considered
                elif hit.gene.name in self.exforbidden_genes.keys():
                    self.forbidden_genes[self.exforbidden_genes[hit.gene.name]] += 1
                    valid_hit = validSystemHit(hit, self.system_name, "forbidden")
                    self.valid_hits.append(valid_hit)                    
                else:
                    msg = "Foreign gene {0} in cluster {1}".format(hit.gene.name, self.system_name)
                    #print msg
                    _log.info(msg)

        if included:
            # Update the number of loci included in the system
            self.nb_cluster += 1
            # Update the positions of the system
            self.loci_positions.append((cluster.begin, cluster.end))


[docs]    def fill_with_hits(self, hits, include_forbidden):
        """
        Adds hits to a system occurence, and check what are their status according to the system definition.
        Set the system occurence state to "no_decision" after calling of this function.

        .. note::
            Forbidden genes will only be included if they do belong to the current system (and not to another specified with "system_ref" in the current system's definition). 

        :param hits: a list of Hits to treat for :class:`macsypy.search_systems.SystemOccurence` inclusion.
        :type list of: :class:`macsypy.report.Hit`
        """
        self._state = "no_decision"
        for hit in hits:
            # Need to check first that this cluster is eligible for system inclusion
            # Stores hits for system extraction (positions, sequences) when complete. 

            if hit.gene.is_mandatory(self.system):
                self.mandatory_genes[hit.gene.name] += 1
                valid_hit = validSystemHit(hit, self.system_name, "mandatory")
                self.valid_hits.append(valid_hit)
            elif hit.gene.is_accessory(self.system):
                self.accessory_genes[hit.gene.name] += 1
                valid_hit = validSystemHit(hit, self.system_name, "accessory")
                self.valid_hits.append(valid_hit)
            elif hit.gene.is_forbidden(self.system):
                self.forbidden_genes[hit.gene.name] += 1

                # SO New: now forbidden genes may be included in the reports:
                if include_forbidden:
                    valid_hit = validSystemHit(hit, self.system_name, "forbidden")
                    self.valid_hits.append(valid_hit)
            else:
                if hit.gene.name in self.exmandatory_genes.keys():
                    self.mandatory_genes[self.exmandatory_genes[hit.gene.name]] += 1
                    valid_hit = validSystemHit(hit, self.system_name, "mandatory")
                    self.valid_hits.append(valid_hit)
                elif hit.gene.name in self.exaccessory_genes.keys():
                    self.accessory_genes[self.exaccessory_genes[hit.gene.name]] += 1
                    valid_hit = validSystemHit(hit, self.system_name, "accessory")
                    self.valid_hits.append(valid_hit)
                # NEW: exforbidden_genes considered
                elif hit.gene.name in self.exforbidden_genes.keys():
                    self.forbidden_genes[self.exforbidden_genes[hit.gene.name]] += 1
                    valid_hit = validSystemHit(hit, self.system_name, "forbidden")
                    self.valid_hits.append(valid_hit)
                else:
                    msg = "Foreign gene {0} in cluster {1}".format(hit.gene.name, self.system_name)
                    _log.info(msg)


[docs]    def fill_with_multi_systems_genes(self, multi_systems_hits):
        """
        This function fills the SystemOccurrence with genes putatively coming from other systems (feature "multi_system").
        Those genes are used only if the occurrence of the corresponding gene was not yet filled with a gene from a cluster of the system. 

        :param multi_systems_hits: a list of hits of genes that are "multi_system" which correspond to mandatory or accessory genes from the current system for which to fill a SystemOccurrence 
        :type list of: :class:`macsypy.report.Hit`

        """
        # For each "multi_system" gene missing:
        for g in self.multi_syst_genes:
            if self.multi_syst_genes[g] == 0:
                #multi_systems_hits should be a dico gene.name-wise?
                # We check wether this missing "multi_system" gene was found elsewhere:
                #if g in multi_gene_names in [multi_gene.name for multi_gene in [hit.gene for hit in multi_systems_hits]]:
                if g in [multi_gene.name for multi_gene in [hit.gene for hit in multi_systems_hits]]:
                    # If so, then the SystemOccurrence is filled with this:
                    if g in self.accessory_genes.keys():
                        self.accessory_genes[g] += 1
                        # Add a valid_hit with a special status? e.g "accessory_multi_system"?
                        #self.accessory_genes[hit.gene.name]+=1
                        #valid_hit=validSystemHit(hit, self.system_name, "accessory")
                        #self.valid_hits.append(valid_hit)

                    elif g in self.mandatory_genes.keys():
                        self.mandatory_genes[g] += 1
                        # Add a valid_hit with a special status? e.g "mandatory_multi_system"?
                        #self.mandatory_genes[hit.gene.name]+=1
                        #valid_hit=validSystemHit(hit, self.system_name, "mandatory")
                        #self.valid_hits.append(valid_hit)

                    _log_out.info("Gene {0} supplied from a multi_system gene".format(g))
        #all_hits = [hit for subl in [report.hits for report in all_reports ] for hit in subl]


[docs]    def decision_rule(self):
        """
        This function applies the decision rules for system assessment in terms of quorum:
            - the absence of forbidden genes is checked
            - the minimal number of mandatory genes is checked (\"min_mandatory_genes_required\")
            - the minimal number of genes in the system is checked (\"min_genes_required\")

        When a decision is made, the status (self.status) of the 
        :class:`macsypy.search_systems.SystemOccurence` is set either to:

            - "\single_locus\" when a complete system in the form of a single cluster was found
            - "\multi_loci\" when a complete system in the form of several clusters was found
            - "\uncomplete\" when no system was assessed (quorum not reached)
            - "\empty\" when no gene for this system was found
            - "\exclude\" when no system was assessed (at least one forbidden gene was found)

        :return: a printable message of the output decision with this SystemOccurrence
        :rtype: string
        """
        nb_forbid = self.count_genes(self.forbidden_genes)
        nb_mandat = self.count_genes(self.mandatory_genes)
        nb_accessory = self.count_genes(self.accessory_genes)
        self._nb_syst_genes = self.compute_nb_syst_genes()

        msg = "====> Decision rule for putative system %s:\n" % self.system_name
        msg += str(self)
        msg += "\nnb_forbid : %d\nnb_mandat : %d\nnb_accessory : %d" % (nb_forbid, nb_mandat, nb_accessory)

        if (nb_forbid == 0):
            if (nb_mandat >= self.system.min_mandatory_genes_required) and (self.nb_syst_genes >= self.system.min_genes_required) and (self.nb_syst_genes  >= 1):
                if self.nb_cluster == 1: 
                    self._state = "single_locus"
                else:
                    self._state = "multi_loci"

                msg += "\nComplete \"%s\" system."%self.state
                msg += "\n******************************************\n"
                #print msg
                #_log.info(msg)

            elif self.nb_syst_genes > 0:
                msg += "\nUncomplete system."
                msg += "\n******************************************\n"
                #print msg
                #_log.info(msg)
                self._state = "uncomplete"

            else:
                msg += "\nEmpty system."
                msg += "\n******************************************\n"
                #print msg
                #_log.info(msg)
                self._state = "empty"
        else:
            msg += "\nExclude."
            msg += "\n******************************************\n"
            #print msg
            #_log.info(msg)
            self._state = "exclude"

        return msg


[docs]class validSystemHit(object):
    """
    Encapsulates a :class:`macsypy.report.Hit`
    This class stores a Hit that has been attributed to a detected system. Thus, it also stores:

    - the system,
    - the status of the gene in this system,

    It also aims at storing information for results extraction:

    - system extraction (e.g. genomic positions)
    - sequence extraction
    """

[docs]    def __init__(self, hit, detected_system, gene_status):
        """
        :param hit: a hit to base the validSystemHit on
        :type hit: :class:`macsypy.report.Hit`
        :param detected_system: the name of the predicted System
        :type detected_system: string
        :param gene_status: the "role" of the gene in the predicted system
        :type gene_status: string

        """
        self._hit = hit
        self.predicted_system = detected_system
        self.reference_system = hit.system.name
        self.gene_status = gene_status

def __getattr__(self, attr_name):
        return getattr(self._hit, attr_name)

    def __str__(self):
        return "{id}\t{rpl_name}\t{pos:d}\t{seq_l:d}\t{gene_name}\t{ref_sys}\t{predict_sys}\
\t{g_status}\t{i_eval}\t{score}\t{prof_cov:f}\t{seq_cov:f}\
\t{begin_match:d}\t{end_match:d}\n".format(id = self.id,
                                           rpl_name = self.replicon_name,
                                           pos = self.position,
                                           seq_l = self.seq_length,
                                           gene_name = self.gene.name,
                                           ref_sys = self.reference_system,
                                           predict_sys = self.predicted_system,
                                           g_status = self.gene_status,
                                           i_eval = self.i_eval,
                                           score = self.score,
                                           prof_cov = self.profile_coverage,
                                           seq_cov = self.sequence_coverage,
                                           begin_match = self.begin_match,
                                           end_match = self.end_match)

    def output_system(self, system_name, system_status):
        return "%s\t%s\t%d\t%d\t%s\t%s\t%s\t%s\t%s\t%s\t%s\t%s\t%f\t%f\t%d\t%d\n" % (self.id,
                                                     self.replicon_name,
                                                     self.position,
                                                     self.seq_length,
                                                     self.gene.name,
                                                     self.reference_system,
                                                     self.predicted_system,
                                                     system_name,
                                                     system_status,
                                                     self.gene_status,
                                                     self.i_eval,
                                                     self.score,
                                                     self.profile_coverage,
                                                     self.sequence_coverage,
                                                     self.begin_match,
                                                     self.end_match)

[docs]    def output_system_header(self):
        """
        :return: the header for the output file
        :rtype: string
        """
        return "#Hit_Id\tReplicon_name\tPosition\tSequence_length\tGene\tReference_system\tPredicted_system\tSystem_Id\tSystem_status\tGene_status\ti-evalue\tScore\tProfile_coverage\tSequence_coverage\tBegin_match\tEnd_match\n"

class systemDetectionReport(object):

    __metaclass__ = abc.ABCMeta

    def __init__(self, systems_occurences_list, cfg):
        self._systems_occurences_list = systems_occurences_list
        self.cfg = cfg
        if 'MACSY_DEBUG' in os.environ and os.environ['MACSY_DEBUG']:
            self._indent = 2 #human readable json for debugging purpose
        else:
            self._indent = None #improve performance of txssview
        self.json_file_name = 'results.macsyfinder.json'


    @abc.abstractmethod
    def report_output(self, reportfilename, print_header = False):
        """
        Writes a report of sequences forming the detected systems, with information in their status in the system, 
        their localization on replicons, and statistics on the Hits.

        :param reportfilename: the output file name 
        :type reportfilename: string
        :param print_header: True if the header has to be written. False otherwise
        :type print_header: boolean

        """
        pass

    @abc.abstractmethod
    def summary_output(self, reportfilename, rep_info, print_header = False):
        """
        Writes a report with the summary of systems detected in replicons. For each system, a summary is done including: 

            - the number of mandatory/accessory genes in the reference system (as defined in XML files)
            - the number of mandatory/accessory genes detected
            - the number and list of missing genes
            - the number of loci encoding the system

        :param rep_info: an entry extracted from the :class:`macsypy.database.RepliconDB`
        :type rep_info: a namedTuple "RepliconInfo" :class:`macsypy.database.RepliconInfo`
        :param print_header: True if the header has to be written. False otherwise
        :type print_header: boolean

        """
        pass

    @abc.abstractmethod
    def json_output(self, path, rep_db):
        """
        Generates the report in json format

        :param path: the path to a file where to write the report in json format
        :type path: string
        :param rep_db: the replicon database
        :type rep_db: a class:`macsypy.database.RepliconDB` object
        """
        pass

[docs]class systemDetectionReportOrdered(systemDetectionReport):
    """
    Stores the detected systems to report for each replicon: 
        - by system name, 
        - by state of the systems (single vs multi loci)

    """

[docs]    def __init__(self, replicon_name, systems_occurences_list, cfg):
        """
        :param replicon_name: the name of the replicon
        :type replicon_name: string
        :param systems_occurences_list: the list of system's occurrences to consider
        :type systems_occurences_list: list of :class:`macsypy.search_systems.SystemOccurence`
        """
        super(systemDetectionReportOrdered, self).__init__(systems_occurences_list, cfg)
        self.replicon_name = replicon_name


[docs]    def counter_output(self):
        """
        Builds a counter of systems per replicon, with different "states" separated (single-locus vs multi-loci systems)

        :return: the counter of systems
        :rtype: Counter
        """
        system_textlist = []
        for so in self._systems_occurences_list:
            system_textlist.append(so.system_name + "_" + so.state)

        return Counter(system_textlist)


[docs]    def tabulated_output_header(self, system_occurence_states, system_names):
        """
        Returns a string containing the header of the tabulated output

        :param system_occurence_states: the different forms of detected systems to consider
        :type system_occurence_states: list of string
        :rtype: string
        """
        # Can be done intra-class 
        header = "#Replicon"
        for syst_name in system_names:
            for state in system_occurence_states:
                header += "\t" + syst_name + "_" + state
        header += "\n"
        return header


[docs]    def tabulated_output(self, system_occurence_states, system_names, reportfilename, print_header = False):
        """
        Write a tabulated output with number of detected systems for each replicon.

        :param system_occurence_states: the different forms of detected systems to consider
        :type system_occurence_states: list of string
        :param reportfilename: the output file name 
        :type reportfilename: string
        :param print_header: True if the header has to be written. False otherwise
        :type print_header: boolean
        :rtype: string

        """
        system_counter = self.counter_output()
        _log_out.info(system_counter)
        report_str = self.replicon_name
        for s in system_names:
            for o in system_occurence_states:
                index = s + "_" + str(o)
                if system_counter.has_key(index):
                    report_str += "\t"
                    report_str += str(system_counter[index])
                else:
                    report_str += "\t0"
        report_str += "\n"

        with open(reportfilename, 'a') as _file:
            if print_header:
                _file.write(self.tabulated_output_header(system_occurence_states, system_names))
            _file.write(report_str)


[docs]    def report_output(self, reportfilename, print_header = False):
        """
        Writes a report of sequences forming the detected systems, with information in their status in the system, 
        their localization on replicons, and statistics on the Hits.

        :param reportfilename: the output file name 
        :type reportfilename: string
        :param print_header: True if the header has to be written. False otherwise
        :type print_header: boolean

        """
        report_str = ""
        for so in self._systems_occurences_list:
            so_unique_name = so.get_system_unique_name(self.replicon_name)
            for hit in so.valid_hits:
                if print_header:
                    report_str += hit.output_system_header()
                    print_header = False
                report_str += hit.output_system(so_unique_name, so.state)

        with open(reportfilename, 'a') as _file:
            _file.write(report_str)


[docs]    def summary_output(self, reportfilename, rep_info, print_header = False):
        """
        Writes a report with the summary of systems detected in replicons. For each system, a summary is done including:

            - the number of mandatory/accessory genes in the reference system (as defined in XML files)
            - the number of mandatory/accessory genes detected
            - the number and list of missing genes
            - the number of loci encoding the system

        :param rep_info: an entry extracted from the :class:`macsypy.database.RepliconDB`
        :type rep_info: a namedTuple "RepliconInfo" :class:`macsypy.database.RepliconInfo`
        :param print_header: True if the header has to be written. False otherwise
        :type print_header: boolean

        """
        report_str = ""
        for so in self._systems_occurences_list:
            if print_header:
                report_str += "{0}\n".format(so.get_summary_header())
                print_header = False

            report_str += "{0}\n".format(so.get_summary(self.replicon_name, rep_info))

        with open(reportfilename, 'a') as _file:
            _file.write(report_str)


[docs]    def json_output(self, json_path, json_data):
        """
        """
        with open(json_path, 'w') as _file:
            json.dump(json_data, _file, indent = self._indent)


[docs]    def _match2json(self, valid_hit, so):
        """
        :param valid_hit: the valid hit to transform in to json.
        :type valid_hit: class:`macsypy.search_system.ValidHit` object.
        :param so: the system occurence where the valid hit come from.
        :type so: class:`macsypy.search_system.SystemOccurence.`
        """
        gene = {}
        gene['id'] = valid_hit.id
        gene['position'] = valid_hit.position
        gene['sequence_length'] = valid_hit.seq_length
        gene['system'] = valid_hit.reference_system
        gene['match'] = valid_hit.gene.name
        gene['gene_status'] = valid_hit.gene_status
        gene['i_eval'] = valid_hit.i_eval
        gene['score'] = valid_hit.score
        gene['profile_coverage'] = valid_hit.profile_coverage
        gene['sequence_coverage'] = valid_hit.sequence_coverage
        gene['begin_match'] = valid_hit.begin_match
        gene['end_match'] = valid_hit.end_match
        gene_ref = so.get_gene_ref(valid_hit.gene)
        if gene_ref:
            gene['function'] = gene_ref.name
        return gene

def _gene2json(self, gene_name, sequence_length, position):
        gene = {'id': gene_name,
                'sequence_length' : sequence_length,
                'position': position
                }
        return gene

[docs]    def system_2_json(self, rep_db):
        """
        Generates the report in json format

        :param path: the path to a file where to write the report in json format
        :type path: string
        :param rep_db: the replicon database
        :type rep_db: a class:`macsypy.database.RepliconDB` object
        """
        systems = []
        for so in self._systems_occurences_list:
            system = {}
            repliconName, systemName, occurenceNumber = so.unique_name.split('_')
            occurenceNumber = int(occurenceNumber)
            system['occurenceNumber'] = occurenceNumber
            system['name'] = systemName
            system['id'] = so.unique_name
            system['replicon'] = {}
            system['replicon']['name'] = so.valid_hits[0].replicon_name # Ok, Otherwise the object has a field self.replicon_name
            rep_info = rep_db[system['replicon']['name']]
            system['replicon']['length'] = rep_info.max - rep_info.min + 1
            system['replicon']['topology'] = rep_info.topology
            system['genes'] = []
            if so.valid_hits:
                positions = [s.position for s in so.valid_hits]
                valid_hits = {vh.id: vh for vh in so.valid_hits}
                pos_min = positions[0] - 5
                if pos_min < rep_info.min:
                    if rep_info.topology == 'circular':
                        pos_min = rep_info.max + positions[0] - 5
                    else:
                        pos_min = rep_info.min
                pos_max = positions[-1] + 5
                if pos_max > rep_info.max:
                    if rep_info.topology == 'circular':
                        pos_max = rep_info.max - positions[-1] + 5
                    else:
                        pos_max = rep_info.max
                if pos_min < pos_max:
                    pos_in_bk_2_display = range(pos_min, pos_max + 1)
                else:
                    before_orig = range(pos_min, rep_info.max + 1)
                    after_orig = range(rep_info.min, pos_max + 1)
                    pos_in_bk_2_display = before_orig + after_orig
                pos_in_rep_2_display = [pos - rep_info.min for pos in pos_in_bk_2_display]
                for curr_position in pos_in_rep_2_display:
                    gene_name, gene_lenght = rep_info.genes[curr_position]
                    if self.cfg.db_type == 'gembase':
                        # SO - PB WAS HERE, NAMES WERE WRONG after the 1st replicon. Thus the gene_id is NEVER in the valid_hits.
                        gene_id = "{0}_{1}".format(system['replicon']['name'], gene_name)
                    else:
                        gene_id = gene_name
                    if gene_id in valid_hits:
                        gene = self._match2json(valid_hits[gene_id], so)
                    else:
                        gene = self._gene2json(gene_id, int(gene_lenght), curr_position + rep_info.min)
                    system['genes'].append(gene)

            system['summary'] = {}
            system['summary']['mandatory'] = so.mandatory_genes
            system['summary']['accessory'] = so.accessory_genes
            system['summary']['forbidden'] = so.forbidden_genes
            system['summary']['state'] = so._state
            systems.append(system)
        return systems


[docs]class systemDetectionReportUnordered(systemDetectionReport):
    """
    Stores a report for putative detected systems gathering all hits from a search in an unordered dataset: 
        - by system.

    Mandatory and accessory genes only are reported in the "json" and "report" output, but all hits matching a system component are reported in the "summary".  

    """

[docs]    def __init__(self, systems_occurences_list, cfg):
        """
        :param systems_occurences_list: the list of system's occurrences to consider
        :type systems_occurences_list: list of :class:`macsypy.search_systems.SystemOccurence`
        """
        super(systemDetectionReportUnordered, self).__init__(systems_occurences_list, cfg)


[docs]    def report_output(self, reportfilename, print_header = False):
        """
        Writes a report of sequences forming the detected systems, with information in their status in the system, 
        their localization on replicons, and statistics on the Hits.

        :param reportfilename: the output file name 
        :type reportfilename: string
        :param print_header: True if the header has to be written. False otherwise
        :type print_header: boolean

        """
        report_str = ""
        for so in self._systems_occurences_list:
            #so_unique_name = so.get_system_unique_name(self.replicon_name)
            so_unique_name = so.get_system_name_unordered()
            #so_unique_name = so.system_name+"_putative"
            for hit in so.valid_hits:
                if print_header:
                    report_str += hit.output_system_header()
                    print_header = False
                report_str += hit.output_system(so_unique_name, so.state)

        with open(reportfilename, 'a') as _file:
            _file.write(report_str)


[docs]    def summary_output(self, reportfilename, print_header = False):
        """
        Writes a report with the summary for putative systems in an unordered dataset. For each system, a summary is done including:

            - the number of mandatory/accessory genes in the reference system (as defined in XML files)
            - the number of mandatory/accessory genes detected

        :param reportfilename: the output file name 
        :type reportfilename: string
        :param print_header: True if the header has to be written. False otherwise
        :type print_header: boolean

        """

        report_str = ""
        for so in self._systems_occurences_list:
            if print_header:
                report_str += "{0}\n".format(so.get_summary_header())
                print_header = False

            #report_str+="%s\n"%so.get_summary(self.replicon_name, rep_info)
            # Get a fake "replicon_name" from the config object in future devt.
            report_str += "{0}\n".format(so.get_summary_unordered("Unordered"))

        with open(reportfilename, 'a') as _file:
            _file.write(report_str)


[docs]    def json_output(self, json_path):
        """
        Generates the report in json format

        :param path: the path to a file where to write the report in json format
        :type path: string
        """
        def cmp_so(so, vh_1, vh_2):
            gene_1 = so.get_gene_ref(vh_1.gene)
            if not gene_1:
                gene_1 = vh_1.gene
            gene_2 = so.get_gene_ref(vh_2.gene)
            if not gene_2:
                gene_2 = vh_2.gene
            if gene_1.is_mandatory(so.system) and gene_2.is_mandatory(so.system):
                return cmp(vh_1.gene.name, vh_2.gene.name)
            elif gene_1.is_mandatory(so.system) and gene_2.is_accessory(so.system):
                return -1
            elif gene_1.is_mandatory(so.system) and gene_2.is_forbidden(so.system):
                return -1

            elif gene_1.is_accessory(so.system) and gene_2.is_mandatory(so.system):
                return 1
            elif gene_1.is_accessory(so.system) and gene_2.is_accessory(so.system):
                return cmp(vh_1.gene.name, vh_2.gene.name)
            elif gene_1.is_accessory(so.system) and gene_2.is_forbidden(so.system):
                return -1

            elif gene_1.is_forbidden(so.system) and gene_2.is_mandatory(so.system):
                return 1
            elif gene_1.is_forbidden(so.system) and gene_2.is_accessory(so.system):
                return 1
            elif gene_1.is_forbidden(so.system) and gene_2.is_forbidden(so.system):
                return cmp(vh_1.gene.name, vh_2.gene.name)
            assert False, "problem during hit comparison"


        with open(json_path, 'w') as _file:
            systems = []
            for so in self._systems_occurences_list:
                if not so.unique_name:
                    so.unique_name = so.get_system_name_unordered()
                system = {}
                system['name'] = so.system_name
                system['id'] = so.unique_name
                system['replicon'] = {}
                system['replicon']['name'] = so.valid_hits[0].replicon_name # Ok, Otherwise the object has a field self.replicon_name
                system['genes'] = []
                so.valid_hits.sort(cmp = lambda x, y:cmp_so(so, x, y))
                for valid_hit in so.valid_hits:
                    gene = {}
                    gene['id'] = valid_hit.id
                    gene['position'] = valid_hit.position
                    gene['sequence_length'] = valid_hit.seq_length
                    gene['system'] = valid_hit.reference_system
                    gene['match'] = valid_hit.gene.name
                    gene['gene_status'] = valid_hit.gene_status
                    gene['i_eval'] = valid_hit.i_eval
                    gene['score'] = valid_hit.score
                    gene['profile_coverage'] = valid_hit.profile_coverage
                    gene['sequence_coverage'] = valid_hit.sequence_coverage
                    gene['begin_match'] = valid_hit.begin_match
                    gene['end_match'] = valid_hit.end_match
                    gene_ref = so.get_gene_ref(valid_hit.gene)
                    if gene_ref:
                        gene['function'] = gene_ref.name
                    system['genes'].append(gene)
                system['summary'] = {}
                system['summary']['mandatory'] = so.mandatory_genes
                system['summary']['accessory'] = so.accessory_genes
                system['summary']['forbidden'] = so.forbidden_genes
                system['summary']['state'] = so._state
                systems.append(system)
            json.dump(systems, _file, indent = self._indent)


[docs]def get_compatible_systems(systems_list1, systems_list2):
    """
    Returns the intersection of the two input systems lists.

    :param systems_list1, systems_list2: two lists of systems 
    :type systems_list1, systems_list2: two lists of :class:`macsypy.system.System`
    :return: a list of systems, or an empty list if no common system
    :rtype: a list of :class:`macsypy.system.System`

    """
    inter = []
    for el in systems_list1:
        if el in systems_list2:
            if not el in inter:
                inter.append(el)
    return inter


[docs]def disambiguate_cluster(cluster):
    """
    This disambiguation step is used on clusters with hits for multiple systems (when cluster.state is set to "ambiguous"). 
    It returns a "cleansed" list of clusters, ready to use for system occurence detection (and that are "clear" cases). It: 

    - splits the cluster in two if it seems that two systems are nearby
    - removes single hits that are not forbidden for the "main" system and that are at one end of the current cluster in this case, check that they are not "loners", cause "loners" can be stored.

    :param cluster: the cluster to "disambiguate"
    :type cluster: :class:`macsypy.search_systems.Cluster`

    """
    res_clusters = []
    counter_genes_compat_systems = {}
    _log_out.info("Disambiguation step:")

    cur_cluster = Cluster(cluster.systems_to_detect) # New
    cur_cluster.add(cluster.hits[0])
    # Now more complex, deals with compatible systems also for disambiguation.
    cur_compatible = cluster.hits[0].gene.get_compatible_systems(cluster.systems_to_detect)

    #print cluster.hits[0]
    #print [syst.name for syst in cur_compatible]
    for h in cluster.hits[1:]:
        #compatible_systems = h.gene.get_compatible_systems(cluster.systems_to_detect) # tmp before yep
        compatible_systems = h.gene.get_compatible_systems(cluster.systems_to_detect, False) # tmp before nope
        compat_list = get_compatible_systems(cur_compatible, compatible_systems) # intersection for the two genes to agglomerate

        #print h
        #print "Hit's:"
        #print [syst.name for syst in compatible_systems]
        #print "Inter:"
        #print [syst.name for syst in compat_list]
        if compat_list:
            # The two consecutive genes have at least one common compatible system
            cur_cluster.add(h)
            cur_compatible = compat_list
        else:
            # Deal with "accessory foreign genes", and system attribution when the current gene can be in both aside systems! 
            # Former cluster (now to save) is considered.
            # Check cluster status before storing it or not:
            cur_cluster.save() # Check if it updates compatible systems?? right?
            if cur_cluster.state == "clear":
                # Update counts of compatible systems with the current cluster to store
                #print "\nclear to store: "
                #print cur_cluster
                res_clusters.append(cur_cluster)
                for syst in cur_compatible: # Good list of compatible??
                    if not counter_genes_compat_systems.has_key(syst.name):
                        counter_genes_compat_systems[syst.name] = len(cur_cluster.hits)
                    else:
                        counter_genes_compat_systems[syst.name] += len(cur_cluster.hits)
                    #print counter_genes_compat_systems
            else:
                # Update counts of compatibles systems for all the hits
                #print "\nnope to store: "
                #print cur_cluster
                for h_clust in cur_cluster.hits:
                    #h_compat = h_clust.gene.get_compatible_systems(cluster.systems_to_detect) # tmp before yep
                    h_compat = h_clust.gene.get_compatible_systems(cluster.systems_to_detect, False) # tmp before nope
                    for syst in h_compat:
                        if not counter_genes_compat_systems.has_key(syst.name):
                            counter_genes_compat_systems[syst.name] = 1
                        else:
                            counter_genes_compat_systems[syst.name] += 1

            cur_compatible = compatible_systems
            #print [syst.name for syst in cur_compatible]            
            cur_cluster = Cluster(cluster.systems_to_detect) # NEW
            cur_cluster.add(h)

    cur_cluster.save()         
   
    # Check cluster status before storing it or not:
    if cur_cluster.state == "clear":
        #print "\nclear to store: "
        #print cur_cluster
        res_clusters.append(cur_cluster) 
        for syst in cur_compatible: # Good list of compatible??
            #print syst.name
            if not counter_genes_compat_systems.has_key(syst.name):
                counter_genes_compat_systems[syst.name] = len(cur_cluster.hits)
            else:
                counter_genes_compat_systems[syst.name] += len(cur_cluster.hits)
    else:
        #print "\nnope to store: "
        #print cur_cluster
        for h_clust in cur_cluster.hits:
            #h_compat = h_clust.gene.get_compatible_systems(cluster.systems_to_detect) # tmp before yep
            h_compat = h_clust.gene.get_compatible_systems(cluster.systems_to_detect, False) # tmp before nope
            for syst in h_compat:
                #print syst.name
                if not counter_genes_compat_systems.has_key(syst.name):
                    counter_genes_compat_systems[syst.name] = 1
                else:
                    counter_genes_compat_systems[syst.name] += 1
    #print "\n---Counter compatible systems ! ---\n%s"%str(counter_genes_compat_systems)

    # Now final check: return only sub-clusters that consist in hits from systems represented at a single locus in the cluster to disambiguate.
    real_res = []
    for r in res_clusters:
        #print "\nres_cluster : "
        #print r
        nb_genes = len(r.hits)
        #print nb_genes
        store = True
        for c in r.compatible_systems:
            #print c
            if c in counter_genes_compat_systems:
                if counter_genes_compat_systems[c] != nb_genes:
                    store = False
                if counter_genes_compat_systems[c] == nb_genes:
                    store = True
                    break
            else:
                store = False                    
        if store:
            #print "=> store !"
            real_res.append(r)

    for r in real_res:
        _log_out.info("Store: ")
        _log_out.info(r)

    #return res_clusters
    return real_res


[docs]def analyze_clusters_replicon(clusters, systems, multi_systems_genes):
    """
    Analyzes sets of contiguous hits (clusters) stored in a ClustersHandler for system detection:

    - split clusters if needed
    - delete them if they are not relevant
    - add eventual genes from other systems "multi_system" genes
    - check the QUORUM for each system to detect, *i.e.* mandatory + accessory - forbidden

    Only for \"ordered\" datasets representing a whole replicon.
    Reports systems occurence.

    :param clusters: the set of clusters to analyze
    :type clusters: :class:`macsypy.search_systems.ClustersHandler` 
    :param systems: the set of systems to detect
    :type systems: a list of :class:`macsypy.system.System`
    :param multi_systems_genes: a dictionary with genes that could belong to multiple systems (keys are system names)
    :return: a set of systems occurence filled with hits found in clusters
    :rtype: a list of :class:`macsypy.search_systems.SystemOccurence` 

    """

    # Global Hits collectors, for uncomplete cluster Hits
    systems_occurences_scattered = {}
    systems_occurences_list = []

    syst_dict = {}
    for system in systems:
        syst_dict[system.name] = system
        systems_occurences_scattered[system.name] = SystemOccurence(system)

    for clust in clusters.clusters:
        _log_out.info("\n{0}".format(clust))
        #if clust.state == "clear":
        systems_to_consider = get_compatible_systems([system_bank[s] for s in clust.compatible_systems], clust.systems_to_detect)
        if clust.state == "clear" and len(systems_to_consider) > 0:
            # Local Hits collector
            # Check the putative system belongs to the list of systems to detect !! If it does not, do not go further with this cluster of genes.
            # New! different compatible systems are tested: then update cluster._putative_system w the good one?
            #print clust.compatible_systems
            # Arbitratily, if none of the set of compatible_systems pass the decision rule step, then the 1st system will store a scattered version of this...
            first = True
            #store_scattered=True
            store_scattered = False
            store_clust = None
            #store_so = None # unused_var
            #store_msg = "" # unused_var
            #exclude = False # unused_var
            #for putative_system in clust.compatible_systems:
            for putative_system in [s.name for s in systems_to_consider]:
                _log_out.info("Considering %s: " % putative_system)
                if putative_system in syst_dict.keys():
                    so = SystemOccurence(syst_dict[putative_system])
                    so.fill_with_cluster(clust)
                    # NEW!
                    if putative_system in multi_systems_genes.keys():
                        so.fill_with_multi_systems_genes(multi_systems_genes[putative_system])
                    msg = so.decision_rule()
                    so_state = so.state
                    _log_out.info("-> %s" % so_state)
                    if so_state != "exclude":
                        if so_state != "single_locus":
                            # Store it to pool genes found with genes from other clusters.
                            # Do not do it if the so has a forbidden gene !!!
                            # NEW !! Now do not do this at the 1st try ! only if not complete is stored in the loop ! 
                            if first:
                                store_clust = clust
                                #store_so = so
                                store_scattered = True
                                #store_msg
                            #print "...\nStored for later treatment of scattered systems.\n"
                            #systems_occurences_scattered[putative_system].fill_with_cluster(clust)
                        else:
                            #print so 
                            #print so_state
                            #print "...\nComplete %s %s system stored.\n"%(putative_system, so_state)
                            _log_out.info(msg)
                            systems_occurences_list.append(so)
                            store_scattered = False
                            break
                    #else:
                    #    exclude = True
                    #    #store_scattered=True
            #if store_scattered and not exclude:
            if store_scattered:
                #print store_so
                #print store_msg
                #print "...\nPutative %s locus stored for later treatment of scattered systems.\n"%clust.compatible_systems[0]
                _log_out.info("=> Putative %s locus stored for later treatment of scattered systems."%clust.compatible_systems[0])
                systems_occurences_scattered[clust.compatible_systems[0]].fill_with_cluster(store_clust)

        elif clust.state == "ambiguous":
            # Implement a way to "clean" the clusters. For instance :
            # - split the cluster in two if it seems that two systems are nearby
            # - remove single hits that are not forbidden for the "main" system and that are at one end of the current cluster
            # in this case, check that they are not "loners", cause "loners" can be stored.
            disamb_clusters = disambiguate_cluster(clust)
            # Add those new clusters to the set of clusters to fill system_occurrences?
            for c in disamb_clusters:
                clusters.add(c)
            if disamb_clusters:
                _log_out.info("=> disambiguated cluster(s) stored for later treatment")
            else:
                _log_out.info("=> none kept")

        else:
            _log_out.info("------- next -------")
    _log_out.info("\n\n***************************************************\n******* Report scattered/uncomplete systems *******\n***************************************************\n")
    for system in systems:
        #print systems_occurences[system]

        # So new: Add support for the multi_loci parameter:
        if system.multi_loci:

            so = systems_occurences_scattered[system.name]
            msg = so.decision_rule()
            so_state = so.state
            #print "-- %s --"%so.system_name
            #print so
            _log_out.info(msg)
            if so.is_complete():
                systems_occurences_list.append(so)
    _log_out.info("******************************************\n")

    # Stores results in this list? Or code a new object : systemDetectionReport ? 
    return systems_occurences_list


[docs]def build_clusters(hits, systems_to_detect, rep_info):
    """
    Gets sets of contiguous hits according to the minimal inter_gene_max_space between two genes. Only for \"ordered\" datasets.

    :param hits: a list of Hmmer hits to analyze 
    :type hits: a list of :class:`macsypy.report.Hit`
    :param systems_to_detect: the list of systems to detect 
    :type systems_to_detect: a list of :class:`macsypy.system.System`
    :param cfg: the configuration object built from default and user parameters.
    :type cfg: :class:`macsypy.config.Config`
    :param rep_info: an entry extracted from the :class:`macsypy.database.RepliconDB`
    :type rep_info: a namedTuple "RepliconInfo" :class:`macsypy.database.RepliconInfo`
    :return: a set of clusters and a dictionary with \"multi_system\" genes stored in a system-wise way for further utilization.
    :rtype: :class:`macsypy.search_systems.ClustersHandler`
    """

    _log.debug("Starting cluster detection with build_clusters... ")

    # Deals with different dataset types using Pipeline ?? 
    clusters = ClustersHandler()
    prev = hits[0]
    #cur_cluster = Cluster()
    cur_cluster = Cluster(systems_to_detect)
    positions = []
    loner_state = False

    # New: storage of multi_system genes:
    multi_system_genes_system_wise = {}

    # Before the case where there is a single hit was not treated...
    """
    if len(hits)==1 and prev.gene.loner:
        #print "LONELY HITS LONER..."
        if positions.count(prev.position) == 0:
            cur_cluster.add(prev)
            #clusters.add(cur_cluster)
            # New : Storage of multi_system genes:
            if prev.gene.multi_system:
                if not prev.system.name in multi_system_genes_system_wise.keys():
                    multi_system_genes_system_wise[prev.system.name] = []
                    multi_system_genes_system_wise[prev.system.name].append(prev)

            positions.append(prev.position)
    """

    tmp = ""
    for cur in hits[1:]:

        _log.debug("Hit {0}".format(cur))
        #prev_max_dist = prev.get_syst_inter_gene_max_space()
        #cur_max_dist = cur.get_syst_inter_gene_max_space()
        prev_max_dist = prev.gene.inter_gene_max_space
        cur_max_dist = cur.gene.inter_gene_max_space
        inter_gene = cur.get_position() - prev.get_position() - 1

        tmp = "\n****\n"
        tmp += "prev_max_dist : {0:d}\n".format(prev_max_dist)
        tmp += "cur_max_dist : {0:d}\n".format(cur_max_dist)
        tmp += "Intergene space : {0:d}\n".format(inter_gene)
        tmp += "Cur : {0}".format(cur)
        tmp += "Prev : {0}".format(prev)
        tmp += "Len cluster: {0:d}\n".format(len(cur_cluster))
        #print tmp

        # First condition removes duplicates (hits for the same sequence)
        # the two others takes into account either system1 parameter or system2 parameter

        #smaller_dist = min(prev_max_dist, cur_max_dist)
        if(inter_gene <= prev_max_dist or inter_gene <= cur_max_dist):
        #if(inter_gene <= smaller_dist ):
            #print "zero"
            if positions.count(prev.position) == 0:
                #print "un - ADD prev in cur_cluster"
                cur_cluster.add(prev)
                positions.append(prev.position)
                # New : Storage of multi_system genes:
                if prev.gene.multi_system:
                    if not prev.system.name in multi_system_genes_system_wise.keys():
                        multi_system_genes_system_wise[prev.system.name] = []
                    multi_system_genes_system_wise[prev.system.name].append(prev)

            if positions.count(cur.position) == 0:
                #print "deux - ADD cur in cur_cluster"
                cur_cluster.add(cur)
                positions.append(cur.position)
                # New : Storage of multi_system genes:
                if cur.gene.multi_system:
                    if not cur.system.name in multi_system_genes_system_wise.keys():
                        multi_system_genes_system_wise[cur.system.name] = []
                    multi_system_genes_system_wise[cur.system.name].append(cur)

            if prev.gene.loner:
                #print "trois - loner_state"
                #print "--- PREVLONER %s %s"%(prev.id, prev.gene.name)
                loner_state = True
        else:
            # Storage of the previous cluster
            if len(cur_cluster) > 1:
                #print cur_cluster
                #print "quatre - ADD cur_cluster"
                clusters.add(cur_cluster) # Add an in-depth copy of the object? 
                #print(cur_cluster)

                #cur_cluster = Cluster()
                cur_cluster = Cluster(systems_to_detect)
                loner_state = False

            elif len(cur_cluster) == 1 and loner_state == True: # WTF?
                #print cur_cluster
                #print "cinq - ADD cur_cluster"
                #print "PREVLONER %s %s"%(prev.id, prev.gene.name)
                clusters.add(cur_cluster) # Add an in-depth copy of the object? 

                cur_cluster = Cluster(systems_to_detect)
                loner_state = False

            if prev.gene.loner:
                #print "six - check"
                #print "PREVLONER ?? %s %s"%(prev.id, prev.gene.name)

                if positions.count(prev.position) == 0:
                    #print "six - ADD prev in cur_cluster, ADD cur_cluster"
                    cur_cluster.add(prev)
                    clusters.add(cur_cluster)
                    #print(cur_cluster)

                    # New : Storage of multi_system genes:
                    if prev.gene.multi_system:
                        if not prev.system.name in multi_system_genes_system_wise.keys():
                            multi_system_genes_system_wise[prev.system.name] = []
                        multi_system_genes_system_wise[prev.system.name].append(prev)

                    positions.append(prev.position)
                    loner_state = False
                    cur_cluster = Cluster(systems_to_detect)

            cur_cluster = Cluster(systems_to_detect)

        prev = cur
        #print "Now prev is %s"%(prev.gene.name)

    if len(cur_cluster) > 1 or (len(cur_cluster) == 1 and prev.gene.loner):
        #print "Recap clusters"
        clusters.add(cur_cluster)

    # Deal both with the case of single loner hits, and of last hits that are loners... YES!
    #print len(cur_cluster)
    if len(cur_cluster) == 0 and prev.gene.loner:
        #print "FIFO or LIFO?"
        cur_cluster.add(prev)
        clusters.add(cur_cluster)
        # New : Storage of multi_system genes:
        if prev.gene.multi_system:
            if not prev.system.name in multi_system_genes_system_wise.keys():
                multi_system_genes_system_wise[prev.system.name] = []
                multi_system_genes_system_wise[prev.system.name].append(prev)

    if rep_info.topology == "circular":
        # Need to take into account the possibility of a single gene at both extremity, that should be considered as part of a cluster (and was not with previous steps)! 
        first = hits[0]
        last = hits[-1]
        hitstoconsider=[]
        #_log_out.info("\n*** Check if single hits at ends are to consider for circularization ***\n")
        if positions.count(first.position) == 0:
            hitstoconsider.append(first)
        if positions.count(last.position) == 0:
            hitstoconsider.append(last)

        #for h in hitstoconsider:
        #    _log_out.info(h)
        clusters.circularize(rep_info, hitstoconsider, systems_to_detect)

    return (clusters, multi_system_genes_system_wise)


[docs]def get_best_hits(hits, tosort = False, criterion = "score"):
    """
    Returns from a putatively redundant list of hits, a list of best matching hits.
    Analyzes quorum and co-localization if required for system detection. 
    By default, hits are already sorted by position, and the hit with the best score is kept, then the best i-evalue. Possible criteria are:

    - maximal score (criterion=\"score\")
    - minimal i-evalue (criterion=\"i_eval\")
    - maximal percentage of the profile covered by the alignment with the query sequence (criterion=\"profile_coverage\")

    :param tosort: tells if the hits have to be sorted
    :type tosort: boolean
    :param criterion: the criterion to base the sorting on 
    :type criterion: string
    :return: the list of best matching hits
    :rtype: list of :class:`macsypy.report.Hit`
    :raise: a :class:`macsypy.macsypy_error.MacsypyError`
    """
    if tosort:
        hits = sorted(hits, key = operator.attrgetter('position'))
    best_hits = []

    prev_hit = hits[0]
    prev_pos = prev_hit.get_position()
    for h in hits[1:]:
        pos = h.get_position()
        if pos != prev_pos:
            best_hits.append(prev_hit)
            #print "******* no comp ****"
            #print prev_hit
            #print "******* ****** ****"
            prev_hit = h
            prev_pos = pos
        else:
            #print "******* COMP ****"
            #print h 
            #print prev_hit
            if criterion == "score":
                if prev_hit.score < h.score:
                    prev_hit = h
                # To be tested before adding it !
                #if prev_hit.score == h.score:
                #    print prev_hit
                #    print h
                #    prev_hit = get_best_hits([prev_hit,h], False, i_eval)[0]
            elif criterion == "i_eval":
                if getattr(prev_hit, 'i_eval') > getattr(h, 'i_eval'):
                    prev_hit = h
            elif criterion == "profile_coverage":
                if getattr(prev_hit, 'profile_coverage') < getattr(h, 'profile_coverage'):
                    prev_hit = h
            else:
                raise MacsypyError("The criterion for Hits comparison % does not exist or is not available. \nIt must be either \"score\", \"i_eval\" or \"profile_coverage\"."%criterion)

            #print "BEST"
            #print prev_hit
            #print "******* ****** ****"

    best_hits.append(prev_hit)
    return best_hits


[docs]def search_systems(hits, systems, cfg):
    """
    Runs search of systems from a set of hits. Criteria for system assessment will depend on the kind of input dataset provided: 

      - analyze **quorum and co-localization** for "ordered_replicon" and "gembase" datasets.
      - analyze **quorum only** (and in a limited way) for "unordered_replicon" and "unordered" datasets.

    :param hits: the list of hits for input systems components
    :type hits: list of :class:`macsypy.report.Hit`
    :param systems: the list of systems asked for detection
    :type systems: list of :class:`macsypy.system.System`
    :param cfg: the configuration object
    :type cfg: :class:`macsypy.config.Config`
    """

    tabfilename = os.path.join(cfg.working_dir, 'macsyfinder.tab')
    reportfilename = os.path.join(cfg.working_dir, 'macsyfinder.report')
    summaryfilename = os.path.join(cfg.working_dir, 'macsyfinder.summary')


    # For the headers of the output files: no report so far ! print them in the loop at the 1st round ! 
    # Update to fit only to the states looked for:
    #system_occurences_states = ['single_locus', 'multi_loci']
    system_occurences_states = ['single_locus']
    system_names = []
    multi_loci = False
    for s in systems:
        syst_name = s.name
        system_names.append(syst_name)
        if s.multi_loci:
            multi_loci = True

    if multi_loci:
        system_occurences_states.append('multi_loci')

    # Specify to build_clusters the rep_info (min, max positions,[gene_name,...), and replicon_type... 
    # Test with psae_circular_test.prt: pos_min = 1 , pos_max = 5569
    #RepInfo= namedtuple('RepInfo', ['topology', 'min', 'max'])
    #rep_info=RepInfo("circular", 1, 5569)

    header_print = True
    if cfg.db_type == 'gembase':
        # Construction of the replicon database storing info on replicons: 
        rep_db = RepliconDB(cfg)
        replicons_w_hits=[]
        json_all_systems = []
        # Use of the groupby() function from itertools : allows to group Hits by replicon_name, 
        # and then apply the same build_clusters functions to replicons from "gembase" and "ordered_replicon" types of databases.
        for k, g in itertools.groupby(hits, operator.attrgetter('replicon_name')):
            sub_hits = list(g)
            rep_info = rep_db[k]

            # The following applies to any "replicon"
            (clusters, multi_syst_genes) = build_clusters(sub_hits, systems, rep_info)          
            _log_out.info("\n************************************\n Analyzing clusters for {0} \n************************************".format(k))
            # Make analyze_clusters_replicon return an object systemOccurenceReport?
            # Note: at this stage, ther is no control of which systems are looked for... But systemsOccurrence do not have to be created for systems not searched. 
            # 
            systems_occurences_list = analyze_clusters_replicon(clusters, systems, multi_syst_genes)  

            _log_out.info("******************************************")
            _log_out.info("Building reports for {0}: \n".format(k))
            report = systemDetectionReportOrdered(k, systems_occurences_list, cfg)

            # TO DO: Add replicons with no hits in tabulated_output!!! But where?! No trace of these replicons as replicons are taken from hits. 
            report.tabulated_output(system_occurences_states, system_names, tabfilename, header_print)
            report.report_output(reportfilename, header_print)
            report.summary_output(summaryfilename, rep_info, header_print)

            json_all_systems += report.system_2_json(rep_db)
            _log_out.info("******************************************")
            header_print = False
            # To add replicons with no systems in the 
            replicons_w_hits.append(k)

        json_path = os.path.join(cfg.working_dir, report.json_file_name)
        report.json_output(json_path, json_all_systems)

        _log_out.info("\n--- Replicons with no hits: ---")
        with open(tabfilename, 'a') as _f:
            for replicon in rep_db.replicon_names():
                if not replicon in replicons_w_hits:
                    _log_out.info(replicon)
                    texte = replicon+"\t0"*len(system_names)*len(system_occurences_states)+"\n"
                    #print texte.strip()
                    _f.write(texte)

    elif cfg.db_type == 'ordered_replicon':
        # Basically the same as for 'gembase' (except the loop on replicons)
        rep_db = RepliconDB(cfg)
        rep_info = rep_db[RepliconDB.ordered_replicon_name]

        #(clusters, multi_syst_genes)=build_clusters(hits, rep_info) 
        (clusters, multi_syst_genes)=build_clusters(hits, systems, rep_info) 
        #for syst in multi_syst_genes:
        #    for g in multi_syst_genes[syst]:
        #        print g
        _log_out.info("\n************************************\n Analyzing clusters \n************************************\n")
        #systems_occurences_list = analyze_clusters_replicon(clusters, systems)
        systems_occurences_list = analyze_clusters_replicon(clusters, systems, multi_syst_genes)
        _log_out.info("******************************************")
        #print "Reporting detected systems : \n"
        _log_out.info("Building reports of detected systems\n ")
        report = systemDetectionReportOrdered(RepliconDB.ordered_replicon_name, systems_occurences_list, cfg)
        report.tabulated_output(system_occurences_states, system_names, tabfilename, header_print)
        report.report_output(reportfilename, header_print)
        report.summary_output(summaryfilename, rep_info, header_print)

        json_all_systems = report.system_2_json(rep_db)
        json_path = os.path.join(cfg.working_dir, report.json_file_name)
        report.json_output(json_path, json_all_systems)
        _log_out.info("******************************************")

    elif cfg.db_type == 'unordered_replicon' or cfg.db_type == 'unordered':

        # implement a new function "analyze_cluster" => Fills a systemOccurence per system
        systems_occurences_list = []
        # Hits with best score are first selected. 
        hits = get_best_hits(hits, True)
        # Then system-wise treatment:
        hits = sorted(hits, key = operator.attrgetter('system'))
        for k, g in itertools.groupby(hits, operator.attrgetter('system')):
            # SO new : if we want to include forbidden genes, 
            # we have to get the corresponding list of hits at this point, 
            # even if this is not their original system... 
            # Need to compute the list of forbidden genes from hits for each system... 
            if k in systems:
                # SO new: get the list of forbidden genes... Then have from hits 
                # Should better rewrite this part of the code to have a single process of the hits...
                #forbidden_genes = k.forbidden_genes # unused_var
                forbidden_hits = []
                for h in hits:
                    if h.gene.is_forbidden(k):
                        forbidden_hits.append(h)
                sub_hits = list(g) + forbidden_hits

                so = SystemOccurence(k)
                #resy=so.fill_with_hits(sub_hits) # does not return anything
                #so.fill_with_hits(sub_hits)
                so.fill_with_hits(sub_hits, True) # SO new parameter to say wether forbidden genes should be included or not. 
                _log_out.info("******************************************")
                _log_out.info(k.name)
                _log_out.info("******************************************")
                _log_out.info(so)
                systems_occurences_list.append(so)
        _log_out.info("******************************************")
        _log_out.info("Building reports of detected systems ")
        #report = systemDetectionReportUnordered(systems_occurences_list, systems)
        report = systemDetectionReportUnordered(systems_occurences_list, cfg)
        report.report_output(reportfilename, header_print)
        report.summary_output(summaryfilename, header_print)
        json_path = os.path.join(cfg.working_dir, report.json_file_name)
        report.json_output(json_path)
        _log_out.info("******************************************")

    else:
        raise ValueError("Invalid database type. ")
```

### Quick search


Enter search terms or a module, class or function name.

### Navigation

- index
- modules |
- MacSyFinder documentation »
- Module code »

© Copyright 2014, Sophie Abby, Bertrand Néron.
Created using Sphinx 1.1.3.
