# Supplementary material for: MacSyFinder: A Program to Mine Genomes for Molecular Systems with an Application to CRISPR-Cas Systems
Source: PLoS One. 2014 Oct 17;9(10):e110726. doi: 10.1371/journal.pone.0110726 (PMC4201578; doi:10.1371/journal.pone.0110726)
Supplement: Data S1 — The MacSyFinder/MacSyView package (compressed tarball archive). (GZ) [file pone.0110726.s010.gz › macsyfinder-1.0.0-RC3/doc/_build/html/_modules/macsypy/system.html]

macsypy.system — MacSyFinder 1.0.0-RC3 documentation


### Navigation

- index
- modules |
- MacSyFinder documentation »
- Module code »

# Source code for macsypy.system

```
# -*- coding: utf-8 -*-

################################################################################
# MacSyFinder - Detection of macromolecular systems in protein datasets        #
#               using systems modelling and similarity search.                 #
# Authors: Sophie Abby, Bertrand Néron                                         #
# Copyright © 2014  Institut Pasteur, Paris.                                   #
# See the COPYRIGHT file for details                                           #
#                                                                              #
# MacsyFinder is distributed under the terms of the GNU General Public License #
# (GPLv3). See the COPYING file for details.                                   #
################################################################################


import logging
_log = logging.getLogger('macsyfinder.' + __name__)
from macsypy_error import SystemInconsistencyError

[docs]class SystemBank(object):
    """
    Build and store all Systems objects. Systems must not be instanciated directly.
    This system factory must be used. It ensures there is a unique instance
    of a system for a given system name.
    To get a system, use the method __getitem__ via the "[]". If the System is already cached in the SystemBank, it is returned.
    Otherwise a new system is built, stored and then returned.
    """

    _system_bank = {}

[docs]    def __getitem__(self, name):
        """
        :param name: the name of the system
        :type name: string
        :return: the system corresponding to the name.
         If the system already exists, return it, otherwise build it and return it.
        :rtype: :class:`macsypy.system.System` object
        """
        if name in self._system_bank:
            return self._system_bank[name]
        else:
            raise KeyError(name)


[docs]    def __contains__(self, system):
        """
        Implement the membership test operator
        
        :param system: the system to test
        :type system: :class:`macsypy.system.System` object
        :return: True if the system is in the System factory, False otherwise
        :rtype: boolean
        """
        return system in self._system_bank.values()


[docs]    def __iter__(self):
        """
        Return an iterator object on the systems contained in the bank
        """
        return self._system_bank.itervalues()


[docs]    def __len__(self):
        """
        :return: the number of systems stored in the bank
        :rtype: integer
        """
        return len(self._system_bank)


[docs]    def add_system(self, system):
        """
        :param system: the system to add
        :type system: :class:`macsypy.system.System` object
        :raise: KeyError if a system with the same name is already registered.
        """
        if system in self._system_bank:
            raise KeyError, "a system named %s is already registered in the systems' bank" % system.name
        else:
            self._system_bank[system.name] = system

system_bank = SystemBank()

[docs]class System(object):
    """
    Handle a secretion system.
    """

[docs]    def __init__(self, cfg, name, inter_gene_max_space, min_mandatory_genes_required = None, min_genes_required = None, max_nb_genes = None, multi_loci = False):
        """
        :param cfg: the configuration object
        :type cfg: :class:`macsypy.config.Config` object
        :param name: the name of the system
        :type name: string
        :param inter_gene_max_space: the maximum distance between two genes (**co-localization** parameter)
        :type inter_gene_max_space: integer
        :param min_mandatory_genes_required: the quorum of mandatory genes to define this system
        :type min_mandatory_genes_required: integer
        :param min_genes_required: the quorum of genes to define this system
        :type min_genes_required: integer
        :param max_nb_genes: 
        :type max_nb_genes: integer
        :param multi_loci: 
        :type multi_loci: boolean
        """
        self.cfg = cfg
        self.name = name
        self._inter_gene_max_space = inter_gene_max_space
        self._min_mandatory_genes_required = min_mandatory_genes_required
        self._min_genes_required = min_genes_required
        if self._min_mandatory_genes_required is not None and self._min_genes_required is not None:
            if self._min_genes_required < self._min_mandatory_genes_required:
                raise SystemInconsistencyError("min_genes_required must be greater or equal than min_mandatory_genes_required")
        self._max_nb_genes = max_nb_genes
        self._multi_loci = multi_loci
        self._mandatory_genes = []
        self._accessory_genes = []
        self._forbidden_genes = []

@property

[docs]    def inter_gene_max_space(self):
        """
        :return: set the maximum distance allowed between 2 genes for this system
        :rtype: integer
        """
        cfg_inter_gene_max_space = self.cfg.inter_gene_max_space(self.name)
        if cfg_inter_gene_max_space is not None:
            return cfg_inter_gene_max_space
        return self._inter_gene_max_space

@property

[docs]    def min_mandatory_genes_required(self):
        """
        :return: get the quorum of mandatory genes required for this system
        :rtype: integer
        """
        cfg_min_mandatory_genes_required = self.cfg.min_mandatory_genes_required(self.name)
        if cfg_min_mandatory_genes_required is not None:
            return cfg_min_mandatory_genes_required
        elif self._min_mandatory_genes_required is None:
            return len(self._mandatory_genes)
        else:
            return self._min_mandatory_genes_required

@property

[docs]    def min_genes_required(self):
        """
        :return: get the minimum number of genes to assess for the system presence.
        :rtype: integer
        """
        cfg_min_genes_requireds = self.cfg.min_genes_required(self.name)
        if cfg_min_genes_requireds is not None:
            return cfg_min_genes_requireds
        elif self._min_genes_required is None:
            return len(self._mandatory_genes)
        else:
            return self._min_genes_required

@property

[docs]    def max_nb_genes(self):
        """
        :return: the maximum number of genes to assess the system presence.
        :rtype: int (or None)
        """
        cfg_max_nb_genes = self.cfg.max_nb_genes(self.name)
        if cfg_max_nb_genes:
            return cfg_max_nb_genes
        else:
            return self._max_nb_genes

@property

[docs]    def multi_loci(self):
        """
        :return: True if the system is authorized to be inferred from multiple loci, False otherwise
        :rtype: boolean
        """
        cfg_multi_loci = self.cfg.multi_loci(self.name)
        if cfg_multi_loci:
            return cfg_multi_loci
        else:
            return self._multi_loci


[docs]    def add_mandatory_gene(self, gene):
        """
        Add a gene to the list of mandatory genes

        :param gene: gene that is mandatory for this system
        :type gene: :class:`macsypy.gene.Gene` object
        """
        self._mandatory_genes.append(gene)


[docs]    def add_accessory_gene(self, gene):
        """
        Add a gene to the list of accessory genes

        :param gene: gene that is allowed to be present in this system
        :type gene: :class:`macsypy.gene.Gene` object
        """
        self._accessory_genes.append(gene)


[docs]    def add_forbidden_gene(self, gene):
        """
        Add a gene to the list of forbidden genes

        :param gene: gene that must not be found in this system
        :type gene: :class:`macsypy.genen.Gene` object
        """
        self._forbidden_genes.append(gene)

@property

[docs]    def mandatory_genes(self):
        """
        :return: the list of genes that are mandatory in this secretion system
        :rtype: list of :class:`macsypy.gene.Gene` objects
        """
        return self._mandatory_genes

@property

[docs]    def accessory_genes(self):
        """
        :return: the list of genes that are allowed in this secretion system
        :rtype: list of :class:`macsypy.gene.Gene` objects
        """
        return self._accessory_genes

@property

[docs]    def forbidden_genes(self):
        """
        :return: the list of genes that are forbidden in this secretion system
        :rtype: list of :class:`macsypy.gene.Gene` objects
        """
        return self._forbidden_genes


[docs]    def get_gene(self, gene_name):
        """
        :param gene_name: the name of the gene to get
        :type gene_name: string
        :return: the gene corresponding to gene_name.
        :rtype: a :class:`macsypy.gene.Gene` object.
        :raise: KeyError the system does not contain any gene with name gene_name.
        """
        all_genes = (self.mandatory_genes, self.accessory_genes, self.forbidden_genes)
        for g_list in all_genes:
            for g in g_list:
                if g.name == gene_name:
                    return g
                else:
                    homolgs = g.get_homologs()
                    analogs = g.get_analogs()
                    for ex in homolgs + analogs:
                        if ex.name == gene_name:
                            return ex
        raise KeyError("System {0} does not contain gene {1}".format(self.name, gene_name))


[docs]    def get_gene_ref(self, gene):
        """
        :param gene: the gene to get the gene reference.
        :type gene: a :class:`macsypy.gene.Gene` or macsypy.gene.Homolog` or macsypy.gene.Analog` object.
        :return: the gene reference of the gene if exists (if the gene is an Homolog or an Analog), otherwise return None.
        :rtype: :class:`macsypy.gene.Gene` object or None
        :raise: KeyError if gene is not in the system
        """
        g = self.get_gene(gene.name)
        try:
            return g.gene_ref
        except AttributeError:
            return None
```

### Quick search


Enter search terms or a module, class or function name.

### Navigation

- index
- modules |
- MacSyFinder documentation »
- Module code »

© Copyright 2014, Sophie Abby, Bertrand Néron.
Created using Sphinx 1.1.3.
