# Supplementary material for: MacSyFinder: A Program to Mine Genomes for Molecular Systems with an Application to CRISPR-Cas Systems
Source: PLoS One. 2014 Oct 17;9(10):e110726. doi: 10.1371/journal.pone.0110726 (PMC4201578; doi:10.1371/journal.pone.0110726)
Supplement: Data S1 — The MacSyFinder/MacSyView package (compressed tarball archive). (GZ) [file pone.0110726.s010.gz › macsyfinder-1.0.0-RC3/doc/_build/html/_modules/macsypy/system_parser.html]

macsypy.system\_parser — MacSyFinder 1.0.0-RC3 documentation


### Navigation

- index
- modules |
- MacSyFinder documentation »
- Module code »

# Source code for macsypy.system\_parser

```
# -*- coding: utf-8 -*-

################################################################################
# MacSyFinder - Detection of macromolecular systems in protein datasets        #
#               using systems modelling and similarity search.                 #
# Authors: Sophie Abby, Bertrand Néron                                         #
# Copyright © 2014  Institut Pasteur, Paris.                                   #
# See the COPYRIGHT file for details                                           #
#                                                                              #
# MacsyFinder is distributed under the terms of the GNU General Public License #
# (GPLv3). See the COPYING file for details.                                   #
################################################################################


import os
import xml.etree.ElementTree as ET
import logging
_log = logging.getLogger('macsyfinder.' + __name__)

from system import System
from gene import Gene
from gene import Homolog, Analog
from registries import ProfilesRegistry, DefinitionsRegistry
from macsypy_error import MacsypyError, SystemInconsistencyError

[docs]class SystemParser(object):
    """
    Build a System instance from the corresponding System definition described in the XML file (named after the system's name) found at the dedicated location ("-d" command-line option). 
    """

[docs]    def __init__(self, cfg, system_bank, gene_bank):
        """
        Constructor

        :param cfg: the configuration object of this run
        :type cfg: :class:`macsypy.config.Config` object
        :param system_bank: the system factory
        :type system_bank: :class:`macsypy.system.SystemBank` object
        :param gene_bank: the gene factory
        :type gene_bank: :class:`macsypy.gene.GeneBank` object
        """
        self.cfg = cfg
        self.system_bank = system_bank
        self.gene_bank = gene_bank
        self.profiles_registry = ProfilesRegistry(cfg)
        self.definitions_registry = DefinitionsRegistry(cfg)


[docs]    def system_to_parse(self, sys_2_parse, parsed_systems):
        """
        :param sys_2_parse: the list of systems to parse
        :type sys_2_parse: [string, ...]
        :return: the list of systems' names to parse. Scan the whole chain of 'system_ref' in a recursive way.
        :rtype: [string, ...]
        """
        diff_sys = list(set(parsed_systems.keys())- set(sys_2_parse.keys()))  
        diff_sys_2 = list(set(sys_2_parse.keys()) - set(parsed_systems.keys()))
        diff_sys += diff_sys_2
        #print diff_sys
        if diff_sys == []:
            return sys_2_parse.keys()
        else:            
            for system_name in diff_sys:
                parsed_systems[system_name] = None
                path = os.path.join(self.cfg.def_dir, system_name + ".xml")
                if not os.path.exists(path):
                    raise MacsypyError("%s: No such system definitions" % path)
                try:
                    tree = ET.parse(path)
                    root = tree.getroot()
                    sys_ref = root.findall(".//gene[@system_ref]")
                    for gene_node in sys_ref:
                        sys_2_parse[gene_node.get("system_ref")] = None
                except Exception, err:
                    msg = "unable to parse system definition \"{0}\" : {1}".format(system_name, err)
                    _log.critical(msg)
                    raise MacsypyError(msg)
                    
            return self.system_to_parse(sys_2_parse, parsed_systems)


[docs]    def _create_system(self, system_name, system_node):
        """
        :param system_name: the name of the system to create.\
          This name must match a XML file in the definition directory ("-d" option in the command-line)
        :type param: string
        :param system_node: the node corresponding to the system.
        :type system_node: :class"`ET.ElementTree` object.
        :return: the system corresponding to the name.
        :rtype: :class:`macsypy.system.System` object.
        """
        path = os.path.join(self.cfg.def_dir, system_name + ".xml")
        inter_gene_max_space = system_node.get('inter_gene_max_space')
        if inter_gene_max_space is None:
            msg = "Invalid system definition ({0}): inter_gene_max_space must be defined".format(path)
            _log.critical(msg)
            raise SyntaxError(msg)
        try:
            inter_gene_max_space = int(inter_gene_max_space)
        except ValueError:
            msg = "Invalid system definition ({0}): inter_gene_max_space must be an integer: {1}".format(path, inter_gene_max_space)
            _log.critical(msg)
            raise SyntaxError(msg)
        min_mandatory_genes_required = system_node.get('min_mandatory_genes_required')
        if min_mandatory_genes_required is not None:
            try:
                min_mandatory_genes_required = int(min_mandatory_genes_required)
            except ValueError:
                msg = "Invalid system definition ({0}): min_mandatory_genes_required must be an integer:{1}".format(path, min_mandatory_genes_required)
                _log.critical(msg)
                raise SyntaxError(msg)

        min_genes_required = system_node.get('min_genes_required')
        if min_genes_required is not None:
            try:
                min_genes_required = int(min_genes_required)
            except ValueError:
                msg = "Invalid system definition ({0}): min_genes_required must be an integer: {1}".format(path, min_genes_required)
                _log.critical(msg)
                raise SyntaxError(msg)

        max_nb_genes = system_node.get('max_nb_genes')
        if max_nb_genes is not None:
            try:
                max_nb_genes = int(max_nb_genes)
            except ValueError:
                msg = "Invalid system definition ({0}): max_nb_genes must be an integer: {1}".format(path, max_nb_genes)
                _log.critical(msg)
                raise SyntaxError(msg)

        multi_loci = system_node.get('multi_loci')
        if multi_loci is not None:
            multi_loci = multi_loci.lower() in ("1", "true")
        else:
            multi_loci = False
        system = System(self.cfg,
                        system_name,
                        inter_gene_max_space,
                        min_mandatory_genes_required,
                        min_genes_required,
                        max_nb_genes,
                        multi_loci)
        return system


[docs]    def _create_genes(self, system, system_node):
        """
        Create genes belonging to the systems. Be careful, the returned genes have not their homologs/analogs set yet.

        :param system: the System currently parsing
        :type system: :class:`macsypy.system.System` object
        :param system_node: the element gene
        :type system_node: :class"`ET.ElementTree` object
        :return: a list of the genes belonging to the system.
        :rtype: [:class:`macsypy.gene.Gene`, ...]
        """
        genes = []
        gene_nodes = system_node.findall(".//gene")
        gene_nodes = [gene_node for gene_node in gene_nodes if gene_node.get("system_ref") is None ]
        for node in gene_nodes:
            name = node.get("name")
            if not name:
                msg = "Invalid system definition '{0}': gene without a name".format(system.name)
                _log.error(msg)
                raise SyntaxError(msg)

            attrs = {}
            for attr in ('loner', 'exchangeable', 'multi_system'):
                val = node.get(attr)
                if val in ("1", "true", "True"):
                    val = True
                elif val in (None, "0", "false", "False"):
                    val = False
                attrs[attr] = val
            inter_gene_max_space = node.get("inter_gene_max_space")
            try:
                inter_gene_max_space = int(inter_gene_max_space)
            except ValueError:
                msg = "Invalid system definition '{0}': inter_gene_max_space must be an integer: %s".format(system.name, inter_gene_max_space)
                _log.critical(msg)
                raise SyntaxError(msg)
            except TypeError:#if None
                pass
            else:
                attrs['inter_gene_max_space'] = inter_gene_max_space
            genes.append(Gene(self.cfg, name, system, self.profiles_registry, **attrs))
        return genes


[docs]    def _fill(self, system, system_node):
        """
        Fill the system with genes found in this system definition. Add homologs to the genes if necessary.
        
        :param system: the system to fill
        :type system: :class:`macsypy.system.System` object
        :param system_node: the "node" in the XML hierarchy corresponding to the system
        :type system_node: :class"`ET.ElementTree` object
        """
        genes_nodes = system_node.findall("gene")
        for gene_node in genes_nodes:
            presence = gene_node.get("presence")
            if not presence:
                msg = "Invalid system definition '{0}': gene without presence".format(system.name)
                _log.error(msg)
                raise SyntaxError(msg)
            gene_name = gene_node.get('name')
            gene = self.gene_bank[gene_name]
            for homolog_node in gene_node.findall("homologs/gene"):
                gene.add_homolog(self._parse_homolog(homolog_node, gene, system))
            for analog_node in gene_node.findall("analogs/gene"):
                gene.add_analog(self._parse_analog(analog_node, gene, system))
            if presence == 'mandatory':
                system.add_mandatory_gene(gene)
            elif presence == 'accessory':
                system.add_accessory_gene(gene)
            elif presence == 'forbidden':
                system.add_forbidden_gene(gene)
            else:
                msg = "Invalid system '{0}' definition: presence value must be either [mandatory, accessory, forbidden] not {1}".format(system.name,
                                                                                                                                      presence)
                _log.error(msg)
                raise SyntaxError(msg)


[docs]    def _parse_homolog(self, node, gene_ref, curr_system):
        """
        Parse a xml element gene and build the corresponding object

        :param node: a "node" corresponding to the gene element in the XML hierarchy
        :type node: :class:`xml.etree.ElementTree.Element` object.
        :param gene_ref: the gene which this gene is homolog to
        :type gene_ref: class:`macsypy.gene.Gene` object
        :return: the gene object corresponding to the node
        :rtype: :class:`macsypy.gene.Homolog` object 
        """
        name = node.get("name")
        if not name:
            msg = "Invalid system definition: gene without name"
            _log.error(msg)
            raise SyntaxError(msg)
        aligned = node.get("aligned")
        if aligned in ("1", "true", "True"):
            aligned = True
        elif aligned in (None, "0", "false", "False"):
            aligned = False
        else:
            msg = 'Invalid system definition \'{0}\': invalid value for an attribute of gene \'{1}\': \'{2}\' allowed values are "1", "true", "True", "0", "false", "False"'.format(curr_system.name,
                                                                                                                                                                        aligned,
                                                                                                                                                                        name)
            _log.error(msg)
            raise SyntaxError(msg)
        try:
            gene = self.gene_bank[name]
        except KeyError:
            msg = "Invalid system definition '{0}': The gene '{1}' described as homolog of '{2}' in system '{3}' is not in the \"GeneBank\" gene factory".format(curr_system.name,
                                                                                                                                                         name, 
                                                                                                                                                         gene_ref.name, 
                                                                                                                                                         curr_system.name)
            _log.critical(msg)
            raise SystemInconsistencyError(msg)
        system_ref = node.get("system_ref")       
        #if system_ref != gene.system.name:     
        if system_ref != None and system_ref != gene.system.name:
            msg = "Inconsistency in systems definitions: the gene '{0}' described as homolog of '{1}' with system_ref '{2}' has an other system in bank ({3})".format(name, 
                                                                                                                                                            gene_ref.name, 
                                                                                                                                                            system_ref, 
                                                                                                                                                            gene.system.name)
            _log.critical(msg)
            raise SystemInconsistencyError(msg)
        homolog = Homolog(gene, gene_ref, aligned)
        for homolog_node in node.findall("homologs/gene"):
            h2 = self._parse_homolog(homolog_node , gene, curr_system)
            homolog.add_homolog(h2)
        return homolog


[docs]    def _parse_analog(self, node, gene_ref, curr_system):
        """
        Parse a xml element gene and build the corresponding object

        :param node: a "node" corresponding to the gene element in the XML hierarchy
        :type node: :class:`xml.etree.ElementTree.Element` object.
        :param gene_ref: the gene which this gene is homolog to
        :type gene_ref: class:`macsypy.gene.Gene` object.
        :return: the gene object corresponding to the node
        :rtype: :class:`macsypy.gene.Analog` object 
        """
        name = node.get("name")
        if not name:
            msg = "Invalid system definition '{0}': gene without name".format(curr_system.name)
            _log.error(msg)
            raise SyntaxError(msg)
        try:
            gene = self.gene_bank[name]
        except KeyError:
            msg = "The gene '{0}' described as analog of '{1}' in system '{2}' is not in the \"GeneBank\" gene factory".format(name, 
                                                                                                                         gene_ref.name, 
                                                                                                                         curr_system.name)
            _log.critical(msg)
            raise SystemInconsistencyError(msg)
        system_ref = node.get("system_ref")       
        if system_ref != None and system_ref != gene.system.name:
            msg = "Inconsistency in systems definitions: the gene '{0}' described as analog of '{1}' with system_ref '{2}' has an other system in bank ({3})" % (name, 
                                                                                                                                                           gene_ref.name, 
                                                                                                                                                           system_ref, 
                                                                                                                                                           gene.system.name)
            _log.critical(msg)
            raise SystemInconsistencyError(msg)
        analog = Analog(gene, gene_ref)
        for analog_node in node.findall("analogs/gene"):
            h2 = self._parse_analog(analog_node , gene, curr_system)
            analog.add_analog(h2)
        return analog


[docs]    def check_consistency(self, systems):
        """
        Check the consistency of the co-localization features between the different values given as an input: 
        between XML definitions, configuration file, and command-line options.
        
        :param systems: the list of systems to check
        :type systems: list of `class:txssnalib.system.System` object
        :raise: :class:`macsypy.macsypy_error.SystemInconsistencyError` if one test fails

        (see `feature <https://projets.pasteur.fr/issues/1850>`_)
          
        In the different possible situations, different requirements need to be fulfilled ("mandatory_genes" and "accessory_genes" consist of lists of genes defined as such in the system definition):
          
          - **If:** min_mandatory_genes_required = None  ; min_genes_required = None
          - **Then:** min_mandatory_genes_required = min_genes_required = len(mandatory_genes)
          
          *always True by Systems design*

          - **If:** min_mandatory_genes_required = value  ; min_genes_required = None
          - **Then:** min_mandatory_genes_required <= len(mandatory_genes) 
          - AND min_genes_required = min_mandatory_genes_required
          
          *always True by design*

          - **If:** min_mandatory_genes_required =  None ; min_genes_required = Value
          - **Then:** min_mandatory_genes_required = len(mandatory_genes)
          - AND min_genes_required >= min_mandatory_genes_required 
          - AND min_genes_required <= len(mandatory_genes+accessory_genes)
          
          *to be checked*

          - **If:** min_mandatory_genes_required =  Value ; min_genes_required = Value
          - **Then:** min_genes_required <= len(accessory_genes+mandatory_genes)
          - AND min_genes_required >= min_mandatory_genes_required
          - AND min_mandatory_genes_required <= len(mandatory_genes)
           
          *to be checked*   
                 
        """
        for system in systems:
            len_accessory_genes = len(system.accessory_genes)
            len_mandatory_genes = len(system.mandatory_genes)
            if not (system.min_genes_required <= (len_accessory_genes + len_mandatory_genes)) :
                msg = "system '{0}' is not consistent: min_genes_required {1:d} must be lesser or equal than the number of \"accessory\" and \"mandatory\" components in the system: {2:d}".format(system.name, 
                                                                                                                                                                                                 system.min_genes_required, 
                                                                                                                                                                                                 len_accessory_genes + len_mandatory_genes)
                _log.critical(msg)
                raise SystemInconsistencyError(msg)

            if not (system.min_mandatory_genes_required <= len_mandatory_genes):
                msg = "system '{0}' is not consistent: min_mandatory_genes_required {1:d} must be lesser or equal than the number of \"mandatory\" components in the system: {2:d}".format(system.name, 
                                                                                                                                                                                         system.min_mandatory_genes_required, 
                                                                                                                                                                                         len_mandatory_genes)
                _log.critical(msg)
                raise SystemInconsistencyError(msg)
            if not(system.min_mandatory_genes_required <= system.min_genes_required):
                msg = "system '{0}' is not consistent: min_mandatory_genes_required {1:d} must be lesser or equal than min_genes_required {2:d}".format(system.name, 
                                                                                                                                               system.min_mandatory_genes_required, 
                                                                                                                                               system.min_genes_required)
                _log.critical(msg)
                raise SystemInconsistencyError(msg)


[docs]    def parse(self, systems_2_detect):
        """
        Parse systems definition in XML format to build the corresponding system objects,\
         and add them to the system factory after checking its consistency.
        To get the system ask it to system_bank
        :param systems_2_detect: a list with the names of the systems to parse
        :type systems_2_detect: list of string
        """
        #systems_2_parse = self.system_to_parse(systems_2_detect)  # une ouverture fermeture de fichier /systeme
        parsed_systems = {}
        systems_2_detect_dict = {}
        for s in systems_2_detect:
            systems_2_detect_dict[s] = None
            
        systems_2_parse = self.system_to_parse(systems_2_detect_dict, parsed_systems)  # une ouverture fermeture de fichier /systeme
        msg = "\nSystem(s) to parse (recursive inclusion of 'system_ref'):"
        for s in systems_2_parse:
            msg += "\n\t-%s"%s
        _log.info(msg)
        
        for system_name in systems_2_parse:
            path = self.definitions_registry.get(system_name)
            if path is None:
                raise MacsypyError("%s: No such system definitions" % path)
            tree = ET.parse(path)
            system_node = tree.getroot()
            sys = self._create_system(system_name, system_node)  # une ouverture par fichier
            self.system_bank.add_system(sys)
            genes = self._create_genes(sys, system_node)
            for g in genes:
                self.gene_bank.add_gene(g)
        #for system_name in systems_2_detect: #une ouverture par fichier # OLD version: only systems to detect were filled appropriately
        for system_name in systems_2_parse: #une ouverture par fichier. # Now, all systems related (e.g. via system_ref) to the one to detect are filled appropriately. 
            system = self.system_bank[system_name]
            path = os.path.join(self.cfg.def_dir, system_name + ".xml")
            tree = ET.parse(path)
            system_node = tree.getroot()
            self._fill(system, system_node)
        system_2_check = [self.system_bank[s] for s in systems_2_detect]
        self.check_consistency(system_2_check)
```

### Quick search


Enter search terms or a module, class or function name.

### Navigation

- index
- modules |
- MacSyFinder documentation »
- Module code »

© Copyright 2014, Sophie Abby, Bertrand Néron.
Created using Sphinx 1.1.3.
